# Improved annotation through genome-scale metabolic modeling of *Aspergillus oryzae*

**DOI:** 10.1186/1471-2164-9-245

**Published:** 2008-05-23

**Authors:** Wanwipa Vongsangnak, Peter Olsen, Kim Hansen, Steen Krogsgaard, Jens Nielsen

**Affiliations:** 1 Department of Systems Biology, Technical University of Denmark, DK-2800 Lyngby, Denmark; 2 Novozymes A/S, DK-2880 Bagsværd, Denmark; 3 Department of Chemical and Biological Engineering, Chalmers University of Technology, SE-412 96 Gothenburg, Sweden

## Abstract

**Background:**

Since ancient times the filamentous fungus *Aspergillus oryzae *has been used in the fermentation industry for the production of fermented sauces and the production of industrial enzymes. Recently, the genome sequence of *A. oryzae *with 12,074 annotated genes was released but the number of hypothetical proteins accounted for more than 50% of the annotated genes. Considering the industrial importance of this fungus, it is therefore valuable to improve the annotation and further integrate genomic information with biochemical and physiological information available for this microorganism and other related fungi. Here we proposed the gene prediction by construction of an *A. oryzae *Expressed Sequence Tag (EST) library, sequencing and assembly. We enhanced the function assignment by our developed annotation strategy. The resulting better annotation was used to reconstruct the metabolic network leading to a genome scale metabolic model of *A. oryzae*.

**Results:**

Our assembled EST sequences we identified 1,046 newly predicted genes in the *A. oryzae *genome. Furthermore, it was possible to assign putative protein functions to 398 of the newly predicted genes. Noteworthy, our annotation strategy resulted in assignment of new putative functions to 1,469 hypothetical proteins already present in the *A. oryzae *genome database. Using the substantially improved annotated genome we reconstructed the metabolic network of *A. oryzae*. This network contains 729 enzymes, 1,314 enzyme-encoding genes, 1,073 metabolites and 1,846 (1,053 unique) biochemical reactions. The metabolic reactions are compartmentalized into the cytosol, the mitochondria, the peroxisome and the extracellular space. Transport steps between the compartments and the extracellular space represent 281 reactions, of which 161 are unique. The metabolic model was validated and shown to correctly describe the phenotypic behavior of *A. oryzae *grown on different carbon sources.

**Conclusion:**

A much enhanced annotation of the *A. oryzae *genome was performed and a genome-scale metabolic model of *A. oryzae *was reconstructed. The model accurately predicted the growth and biomass yield on different carbon sources. The model serves as an important resource for gaining further insight into our understanding of *A. oryzae *physiology.

## Background

*A. oryzae *is a member of the diverse group of aspergilli that includes species that are important microbial cell factories, as well as species that are human and plant pathogens [1]. *A. oryzae *has been used safely in the fermentation industry for hundreds of years in the production of soy sauce, miso and sake. Today *A. oryzae *is also used for production of a wide range of different fungal enzymes such as α-amylase, glucoamylase, lipase and protease and it is regarded as an ideal host for the synthesis of proteins of eukaryotic origin [1]. In the post genome-sequencing era, various high-throughput technologies have been developed to characterize biological systems on the genome-scale [2]. Discovering new biological knowledge from high-throughput biological data and assigning biological functions to all the proteins encoded by the genome is, however, challenging and allowing systems level investigations of microbial cell factory. For fungi, several genome-sequencing and annotation projects have been presented, including *Saccharomyces cerevisiae *[3], *A. nidulans *[4], *A. fumigatus *[5], and *A. niger *[6,7]. Recently, genome sequence of *A. oryzae *by Machida and his coworkers has been published [8]. Based on their sequence annotation using gene-finding software tools such as ALN [9], GlimmerM [10] and GeneDecoder [11], this analysis 12,074 genes encoding proteins were predicted to be present in the genome [8]. Despite this prediction many genes had not been assigned a definite function, and of the 12,074 genes, more than 50% were annotated as hypothetical proteins. Hence, there are clearly opportunities for refining the gene prediction and improving the annotation. However, the present one dimensional data does not allow for complete annotation of all genes and it would therefore be interesting and potentially fruitful to use integrative biological tools in the process of improving the annotation of fungal genomes [12]. In this process reconstruction of a genome-scale metabolic model is a good starting point as it allows for integration of various types of data. Nowadays, there are several open sources of fungal metabolic models, such as for *S. cerevisiae *[13], *A. nidulans *[14], *A. niger *[15] and a model for the central carbon metabolism of *A. niger *[16]. These models currently are prominent as one of the most promising approaches to achieve an *in silico *prediction of cellular function in terms of physiology [17].

The aim of this study is to improve the annotation of the genome sequence of *A. oryzae *and further integrate enhanced annotated data to construct a genome-scale metabolic model of *A. oryzae*. The first *A. oryzae *EST library, sequencing and assembly were performed in order to improve gene prediction. Then functional assignment was done by our developed annotation strategy and a combination of different bioinformatics tools and databases. The bioinformatics tools used were BLAST [18], HMMER [19], and PSI-BLAST [20]. Several databases used were namely the *A. oryzae *genome database [21], the EST database of *A. flavus *[22], the *A. nidulans *genome database [23], the *A. fumigatus *genome database [24], the *S. cerevisiae *genome database [25], the Pfam protein families database [26], the COG database [27], and the Non-Redundant (NR) protein database [28]. Subsequently, manual inspection was through in order to achieve a solid annotation for enzyme functions that were needed for reconstruction of the metabolic network. Based on the improved annotated genome, the genome-scale metabolic network was reconstructed. The network was built by comparison with other related metabolic models, namely models for *S. cerevisiae *[13], *A. nidulans *[14], and *A. niger *[15,16], and biochemical pathway databases, literature, as well as experimental evidence for the presence of specific pathways. The biomass composition was taken from the literature, whereas, maintenance and growth-associated ATP consumption rates were estimated based on literature data on yields and growth rates. Finally, Flux Balance Analysis (FBA) was used to predict the flux distributions in the metabolic network, and the biomass yields as well as growth rates on different carbon sources were estimated to validate the metabolic model of *A. oryzae*.

## Results and Discussion

### Gene discovery and validation

**Figure 1:**
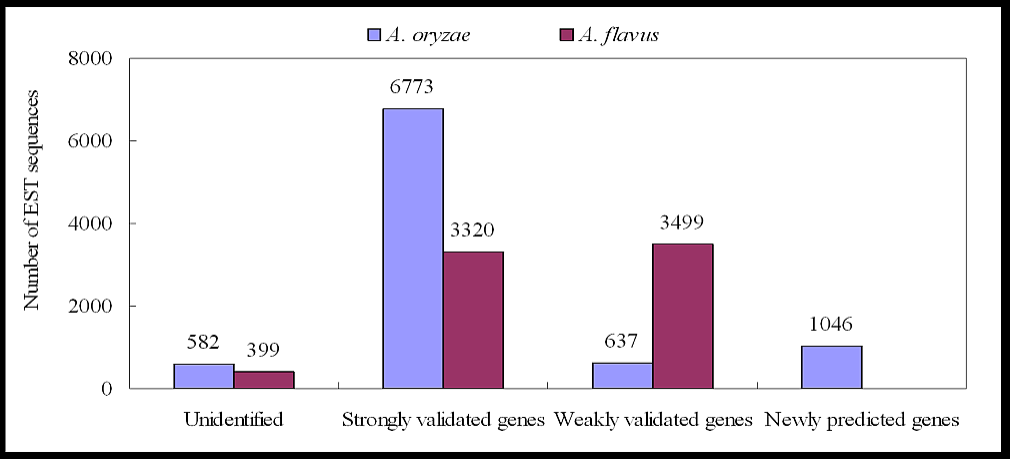
**Gene discovery and validation of existing genes**. The bars show the number of new genes discovered and the number of existing gene validated by our assembled EST sequences of *A. oryzae *and EST data of *A. flavus *[22].

The assembled EST sequences of *A. oryzae *were achieved from this study (see Additional file 1) where were deposited into Genbank database under accession numbers "EY424375–433412". Within our assembled EST data analysis of *A. oryzae*, we found 9,038 EST contig sequences with a GC content of 51.2% and an average EST length of 738 base pairs (bps). Based on analysis of sequences obtained from Machida and coworkers [8], the *A. oryzae *genome consists of eight chromosomes containing 37.2 Megabases (Mb) with a GC content of 48.2% and 12,074 annotated genes. According to the described strategy implemented for gene finding (See Methods), the 9,038 EST sequences were searched against the 12,074 previously identified genes [8] in the sequenced genome using various search parameters to create lists of predicted genes with different match stringencies. Using the criteria described in the Methods, many dissimilar sequences between the EST sequences and previously identified gene sequences of *A. oryzae *[8] were found. This suggests the presence of many newly predicted genes. Interestingly, approximately 12% (1,046 out of the 9,038 EST sequences) were categorized as newly predicted genes in the genome. Many homolog sequences were also found strongly validating previously identified genes [8], with approximately 75% of the total EST sequences (6,773 out of the 9,038 EST sequences) matching earlier identified genes (See Figure 1). To confirm that all the EST sequences do existed in the *A. oryzae *genome, the 9,038 EST sequences were searched by BLASTN [18] against the complete genome, and the results showed that only 20 EST sequences could not be found to be present in the genome. Therefore, this suggests that the assembled EST data of *A. oryzae *had very high quality and showed an excellent success rate for gene discovery and validation, even though approximately 13% (1,219 out of the 9,038 EST sequences) could not be used to predict genes, because 6% (582 out of the 9,038 EST sequences) were too short and about 7% (637 out of the 9,038 EST sequences) were too weakly validated in the original gene list using a conservative cut-off. In another attempt to predict new genes in *A. oryzae *genome, *A. flavus *EST contigs stored in the TIGR public database [22] were also used because *A. flavus *and *A. oryzae *are very closely related [29]. Also, there is a high degree of DNA homology between the two organisms (e.g. aflatoxin cluster > 96%) [29]. *A. flavus *EST library contained 7,218 sequences with a GC content of 49.7% and an average EST length of 636.2 bps. Using these *A. flavus *EST sequences to search against the genes in our new gene list for the *A. oryzae *genome, no new genes were predicted but 3,320 genes in the *A. oryzae *genome were validated by EST sequences (see Figure 1). Based on all the results of the gene finding a total of 13,120 protein-encoding genes were identified in the *A. oryzae *genome. This total number of genes derives from 12,074 previously annotated genes by Machida et al and 1,046 newly predicted genes from our assembled EST library.

### Identification of protein functions by pairwise comparison

In order to assign protein functions to the 13,120 predicted genes, sequence alignment analysis by pairwise comparison between *A. oryzae *and closely related fungi was performed. These fungi included *A. nidulans*, *A. fumigatus *and *S. cerevisiae*. Table 1 shows some genome characteristics of the related fungi in comparison with *A. oryzae*. Initially pairwise comparison was done by similarity searching of the protein sequences of *A. oryzae *against the protein sequences of other related fungi as described in the Methods. With a chosen threshold of the alignment length (bps) and identity (%), a list of putative protein functions was created. The results are summarized in Table 1. Pairwise comparison shows that *A. fumigatus *has 6,274 homologs with *A. oryzae *sequences. It is the highest number of sequence homologs and this indicates the highest percentage (88%) of the homologs obtained between the three species tested. This result is consistent with the fact that *A. oryzae *and *A. fumigatus *are the phylogenetically closest species of those evaluated [4,30]. Upon completion of the similarity searching, the results suggest that 7,161 genes in *A. oryzae *could be assigned as orthologous genes from the three fungi used for comparison. Of these 7,161 protein sequences, 5,836 sequences were assigned putative protein functions for *A. oryzae*. These functions were mainly obtained from *A. fumigatus *(Table 1). The remaining 1,325 sequences that have homologs in the three other fungi could not been assigned any function yet, and they are therefore classified as hypothetical proteins. The putative functions annotated here were classified using biological process (BP) type from the Gene Ontology (GO) database [31]. The genes and functions that have biological process terms involved in metabolism, including both biosynthesis and catabolism, were extracted and used for metabolic network reconstruction. The results of this process show that the *A. oryzae *genome contains 1,924 genes (15% of the 13,120 total genes) encoding 1,070 different protein functions involved in metabolism.

**Table 1:** Comparison of genome characteristics and function assignments between *A. oryzae *and other related fungi

**Genome characteristics**				
**Features**	***ANI*^1^**	***AFU*^2^**	***SC*^3^**	***AO*^4^**

Genome size (Mb)	30.1	29.4	12.1	37.2
Number of chromosomes	8	8	16	8
Number of total predicted genes	10,701	10,267	5,869	13,120

**Function assignments**				

**Pairwise comparison**	***ANI *and *AO***	***AFU *and*AO***	***SC *and*AO***	***AO***

Number of protein sequence homologs	6,095	6,274	1,794	7,161
Percentage of sequence homologs	85	88	25	100
Number of assigned putative functions	837	5,482	1,731	5,836
Percentage of assigned putative functions	14	93	30	100
Number of predicted genes involved in metabolism	567	1,556	837	1,924
Number of putative functions involved in metabolism	377	1,132	495	1,070

*ANI*^1^: Data were obtained from *A. nidulans *genome database [23]

*AFU*^2^: Data were obtained from *A. fumigatus *genome database [24]

*SC*^3^: Data were obtained from *S. cerevisiae *genome database [25]

*AO*^4^: Data were obtained from *A. oryzae *genome database [21]. Notably, total predicted genes were achieved from both database [21] and our EST sequence analysis.

### Metabolic pathway mapping

The metabolic models for *S. cerevisiae *[13], *A. nidulans *[14], and *A. niger *[15,16] were combined to generate an initial reaction list for the construction of the *A. oryzae *metabolic network. Duplicated reactions were removed resulting in a list of 1,924 genes and 1,070 functions involved in metabolism. For each enzyme function involved in this reaction list it was searched in the above generated list of metabolic proteins present in *A. oryzae*. If an enzyme name matched, then the enzyme-encoding genes, enzyme functions and Enzyme Commission (EC) numbers of *A. oryzae *were selected and mapped onto this reaction list. Hereafter a classification system was established to divide reactions in the whole metabolic network of *A. oryzae *into 7 main metabolic pathways: carbohydrate metabolism, energy metabolism, amino acid metabolism, nucleotide metabolism, lipid metabolism, cofactor metabolism and secondary metabolism. It is hereby found that the highest number of enzyme-encoding genes is involved in carbohydrate metabolism, which is consistent with the fact that *A. oryzae *has the ability to use a wide range of carbohydrate substrates. For amino acid and lipid metabolisms, many enzyme-encoding genes were also found. A lower number of enzyme-encoding genes were found in nucleotide, cofactor and energy metabolisms. The lowest number of enzyme-encoding genes was found in secondary metabolism. In fact, the *A. oryzae *genome contains a lot of enzyme-encoding genes involved in secondary metabolism [29], but most of these genes are without EC numbers and could therefore not be mapped onto the metabolic network. The hereby resulting metabolic network contains several gaps, which means that there are metabolic reactions without corresponding enzymes.

### Filling gaps in the metabolic network using an integrated bioinformatics tool

**Figure 2:**
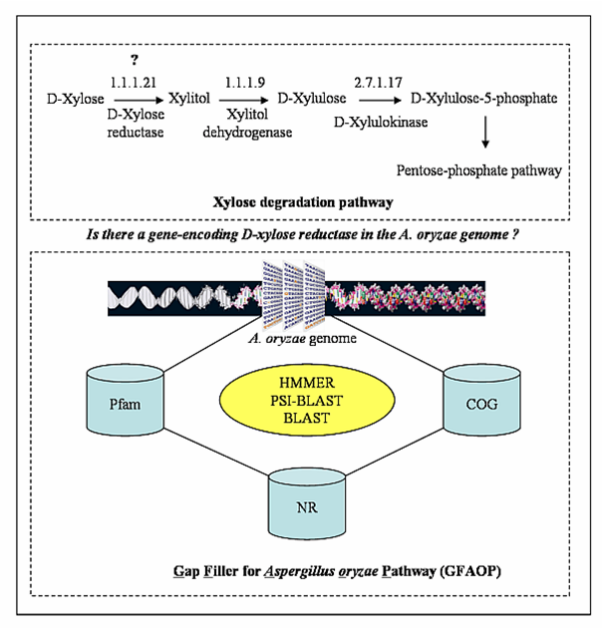
**Filling gap by integrated bioinformatics approach**. A diagram of the integrated bioinformatics tools used for filling the gaps in the metabolic network. A missing enzyme of D-xylose reductase in xylose degradation pathway is used as an example to illustrate the gap filling process.

In order to identify genes encoding more enzyme functions and hereby reduce the number of gaps in the metabolic network, an integrated bioinformatics tool was developed and used to identify these missing enzymes. This tool called "Gap Filler for *Aspergillus oryzae *Pathway (GFAOP)" was developed in- house by combining different bioinformatics tools (i.e. BLAST [18], HMMER [19], and PSI-BLAST [20]) and databases (i.e. *A. oryzae *genome [21], Pfam [26], COG [27], and NR [28]). GFAOP is similar to the McConkey searching algorithm which has been used for enzyme identification in eukaryote genomes [32]. The method is also related to Osterman's method for the identification of bacterial genes encoding metabolic functions [33]. An overview of GFAOP is shown in Figure 2. First, the tool was validated by searching for 441 known protein functions in *A. oryzae *using the information from the genome database [21]. The tool confirmed 100% accuracy of the prediction. This tool was then used to search for functional activity related to missing enzyme (Gap) in the metabolic reaction. To illustrate this approach, one of the missing enzymes ("D-xylose reductase" (EC: 1.1.1.21)) in the pathway of xylose degradation of *A. oryzae *is selected as an example. To answer the question of whether there is a gene encoding D-xylose reductase in *A. oryzae*. GFAOP was applied as follows. First the HMMER program generates a Hidden Markov Model (HMM) profile of this enzyme (D-xylose reductase) from the protein families databases (such as Pfam or COG). Second, a consensus sequence is generated. Third, the consensus sequence is searched against the *A. oryzae *genome by a PSI-BLAST [20]. Sequences where the hit has suitable statistical significance values are selected and extracted for protein function assignment by searching against the NR protein database [28] using BLAST [18] to verify its probable function.

The result clearly shows that there is a high probability for that the gene called "AO090003000859" encode D-xylose reductase. Based on searching of this gene in the *A. oryzae *genome database [21], the gene name AO090003000859 is only reported for general prediction and poorly characterized functions. Moreover, the exploration in other databases such as the Genbank, this gene name is only showed to have a region encoding aldo/keto reductase family proteins, but there is no evidence on the specific function of the gene. As a result from using GFAOP, the missing enzyme of D-xylose reductase is entered into the pathway. Our method results in an improved annotation of the genome using the context of the metabolic network. An iterative process was done for filling all the gaps in the whole metabolic network. Ultimately, 210 gaps in the metabolic network were closed using GFAOP. These gaps distributed with 86 gaps in lipid metabolism, 31 gaps in secondary metabolism, 34 gaps in amino acid metabolism, 23 gaps in nucleotide metabolism, 17 gaps in carbohydrate metabolism, 10 gaps in cofactor metabolism, and 9 gaps in energy metabolism.

### Characteristics of the improved annotation and reconstructed metabolic network

**Figure 3:**
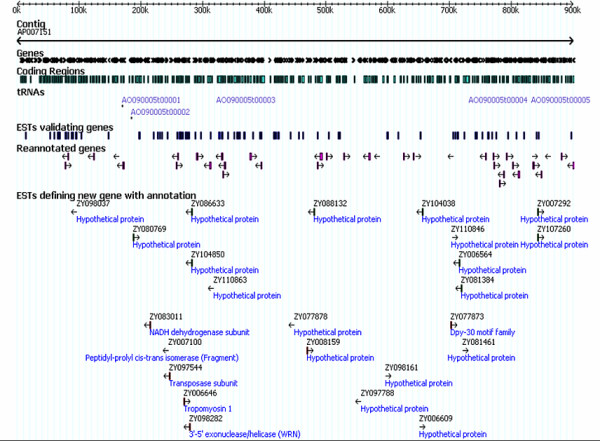
**Gene and EST mapping on the *A. oryzae *genome**. An example of how we map genes and ESTs on the AP007151 contig, a part of chromosome 1 of the *A. oryzae *genome. Along this contig, we mapped EST sequences defining new genes with annotation, EST sequences validating genes, and also re-annotated genes.

The annotation process resulted in the improved annotated data shown in Table 2 where the data are compared with values in the *A. oryzae *genome database by Machida et al [21]. The results show that the number of improved annotated genes is 13,120 which are higher than the number of genes in the database [21]. Of these improved annotated data, the predicted genes and the putative functions are distributed into different groups. The first group contains new putative protein functions assigned to newly predicted genes, and it contains 398 new putative protein functions that are divided into 154 metabolic functions and 244 other functional groups. The second group contains hypothetical proteins assigned to newly predicted genes and it contains 648 hypothetical proteins. The third group is new putative protein functions assigned to proteins previously annotated as hypothetical proteins, and this group comprises 1,469 proteins of which 562 proteins have metabolic functions. The final group contains genes that is found to have the same putative protein function as previously reported in the database [21]. In total the hereby annotated genome of *A. oryzae *contains 5,391 protein functions of which 3,178 have metabolic functions. Even though the genome still contains 5,214 hypothetical proteins this is less than the 6,683 hypothetical proteins currently reported in the database [21], and our work therefore resulted in a substantial improvement of the genome annotation. An enhanced annotated data were mapped on the *A. oryzae *genome by using the Perl Scalable Vector Graphics (SVG) Module V2.33 [34]. Figure 3 shows an example of gene and EST mapping on the contig of AP007151 which is a part of chromosome 1 of the *A. oryzae *genome. The complete genomic map is available as Additional file 2. The list of all ESTs and genes contained on the genomic map is presented as Additional file 3.

**Table 2:** Statistical characteristics of improved annotation and metabolic reconstruction.

**Characteristics of improved annotation**	**Improved annotated data **	**Database**
Total protein-encoding genes	13,120	12,074
New putative protein functions to newly predicted genes	398	-
Metabolic functions	154	-
Other functional groups	244	-
Hypothetical proteins to newly predicted genes	648	-
New putative protein functions to previously hypothetical proteins	1,469	-
Metabolic functions	562	-
Other functional groups	907	-
Same putative protein functions	5,391	5,391
Metabolic functions	3,178	3,178
Other functional groups	2,213	2,213
Hypothetical proteins	5,214	6,683

**Characteristics of network **	***A. oryzae***	***A. nidulans***

Enzymes-encoding genes	1,314	666
Enzymes	729	466
Metabolites	1,073	733
Biochemical reactions	1,846 (1,053 Unique)	1,090 (676 Unique)
Cytosol	832	551
Mtochondria	172	103
Glyoxysome	-	5
Peroxisome	19	-
Extracellular	30	17
Transport reactions	281 (161 Unique)	118 (113 Unique)
Reactions with gene assignments	173 (53 Unique)	15 (12 Unique)
Reactions without gene assignments	108 (108 Unique)	103 (101 Unique)

An improved annotated data is compared with genome database of *A. oryzae *[21]. The reconstructed metabolic network of *A. oryzae *is compared with the reconstructed metabolic network of *A. nidulans *[14]

**Figure 4:**
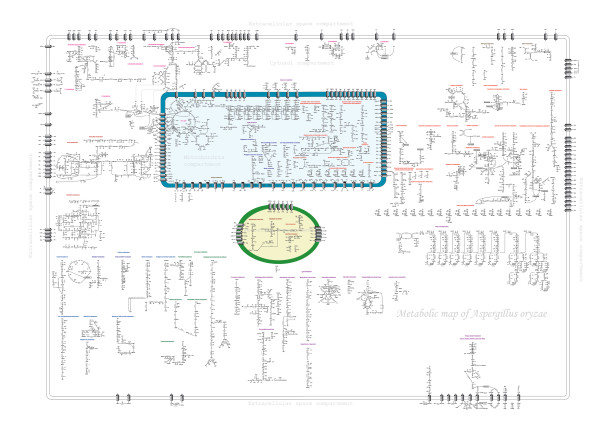
**Overall metabolic map of *A. oryzae***. A full size of metabolic map of *A. oryzae *is viewed in Additional file 5.

As previously mentioned the improved annotation resulted in a final reconstructed metabolic network that contains 729 enzymes, 1,846 (1,053 unique) biochemical reactions and 1,073 metabolites (Table 2). The large number of isoenzymes (indicated by the difference between total biochemical reactions and unique biochemical reactions) points to a very high degree of flexibility in the metabolic network of *A. oryzae*. The 1,053 unique biochemical reactions are distributed into 832 cytosolic, 172 mitochondrial, 19 perosixomal, and 30 extracellular reactions. There are 281 (161 unique) reactions that function as transport processes, and of these 173 (53 unique) are included on the basis of gene assignments whereas there are no annotated genes for 108 of the transport reactions. All the genes and functions involved in metabolism were inspected manually. A total of 1,314 genes without duplication represented as enzyme-encoding genes are included in the reconstructed network. This corresponded to about 10% of the 13,120 total predicted genes of *A. oryzae*. For model comparison, the metabolic network of *A. nidulans *[14] was chosen, and it shows that the metabolism of *A. oryzae *is much larger than that of *A. nidulans *as it contains a higher number of genes, enzymes, metabolites and reactions (see Table 2). A list of the reactions in the reconstructed metabolic network that comprised the genes, EC numbers and enzymes was hereby obtained (see Additional file 4). To illustrate a whole network, overall metabolic map of *A. oryzae *was drawn as shown in Figure 4 (also see in Additional file 5 for full size) to represent a valuable link between genes, enzymes, metabolic reactions and metabolites. The complete metabolite list (with full name) is also given as Additional file 4.

### Biomass growth simulation

Using the reconstructed metabolic network, a stoichiometric model was developed and subsequently used to simulate growth. A list of the reactions that comprise the stoichiometric model is presented as Additional file 4. To describe growth, biomass production is regarded as a drain of macromolecules and building blocks required to produce cellular components. The demands on each of these compounds are estimated based on the biomass composition. No drain of free metabolites or dilution of the metabolite pool due to biomass growth is considered [35]. The cellular composition considered for *A. oryzae *is based on the contents of the main biomass components of *A. oryzae *[36] as shown in Table 3 (see also Additional file 4 for the original data used to perform this analysis). In addition, concerning the biomass composition, the only parameters that have to be estimated are key energetic parameters: ATP requirement for non-growth associated purposes (mATP), ATP requirement for synthesis of biomass from macromolecules (K_ATP_) and the operational P/O ratio. These parameters can not be determined independently, but if one of the parameters is known the others can be estimated from experimental data. The operational P/O ratio was assumed to be 1.5 [35], and mATP (mmol/gDW) was estimated to be 1.9 and K_ATP _(mmol/gDW) was estimated by fitting model simulation with experimental data obtained at a specific growth rate of 0.1 h^-1 ^[36] with glucose as the sole carbon source. The value of K_ATP _was hereby estimated to be 49 mmoles ATP/g DW.

**Table 3:** Biomass composition in the metabolic model of *A. oryzae*

**Biomass component**	**Average molecular weight^1 ^[g/mol]**	**Content^2 ^[g/100 g DW]**		**Stoichiometric coefficient^4 ^[mmol/g DW]**
			***Normalized***^3^	
Proteins	134.58	40	47.1	3.50075
Carbohydrates	-	28	33	-
Glycogen	666.6	0.1	0.1	0.00212
Chitin	203.2	7	8.3	0.40759
Glucan	162.1	20.8	24.6	1.51453
RNA	341.9	5.3	6.2	0.18259
DNA	332.3	0.8	0.9	0.02836
Lipids	-	6.8	8	-
Triacylglycerol	954.96	2.12	2.49	0.02617
Free fatty acid	301.31	0.35	0.41	0.01365
Phosphatidylethanolamine	782.5	0.97	1.14	0.01468
Phosphatidylcholine	834.8	2.38	2.8	0.03356
Phosphatidylserine	827.3	0.4	0.47	0.00564
Phosphatidylamine	755.24	0.58	0.68	0.00903
D-Mannitol	182.2	3.3	3.9	0.21333
Glycerol	92.1	0.7	0.8	0.08952
Ash	-	15.1	-	-

^1^Average molecular weights (units: g/mol of monomers in polymer)

^2^For growth on glucose, using ammonia as the nitrogen source and for a specific growth rate of 0.1 h^-1^

^3^Without considering ash

^4^In the equation representing biomass formation (units: mmol of monomers in polymer/g DW)

### Assessment of model validation of *A. oryzae*

**Figure 5:**
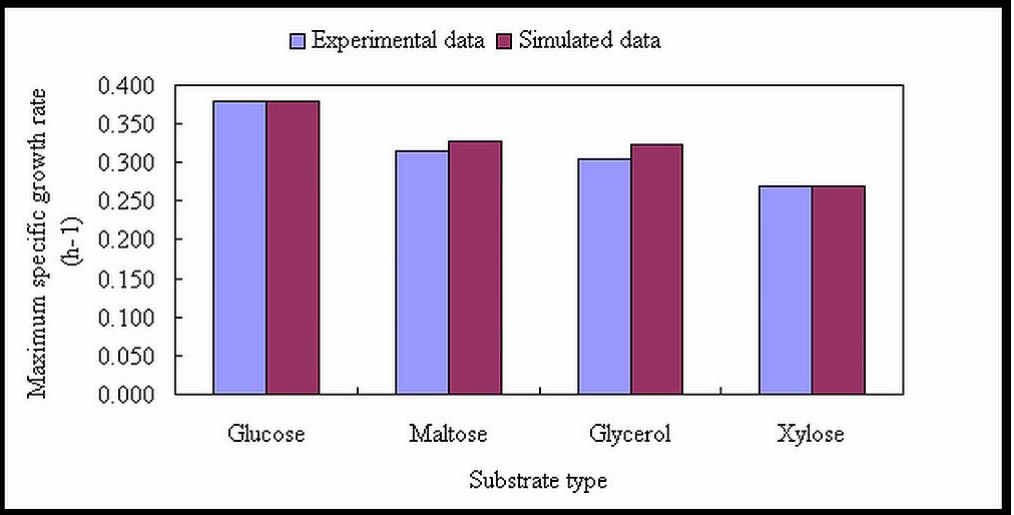
**Model validation by experimental data**. Comparison of the maximum specific growth rate (h^-1^) between simulated data and experimental data. The experimental data were obtained from batch fermentation.

**Figure 6:**
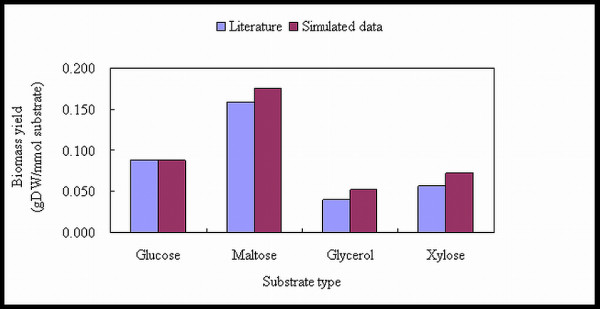
**Model validation by literature**. Comparison of biomass yield (gDW/mmol substrate) obtained by model simulation data and data from the literature [37, 38]. The biomass yield was obtained from chemostat fermentation.

The model was evaluated by simulating *A. oryzae *cell growth on different carbon sources and comparison of the simulated data to the experimentally determined growth rate and biomass yield from literature data [37,38]. For each carbon source the substrate uptake rate was estimated from measurements of the substrate concentration in the medium, and this value is used as input to the model. From this input the flux distributions corresponding to optimal growth are calculated by maximizing the flux of the reaction leading to biomass. The validation results are shown in Figure 5 and Figure 6. From the results, Figure 5 indicates that the model can accurately predict the maximum specific growth rate (h^-1^) during batch cultivations on different carbon sources (when the uptake rate of the carbon source is given as input). The accuracy is on average about 98% of the experimentally determined value. Figure 6 also shows that the model can accurately predict the biomass yield (gDW/mmol substrate) during chemostat cultivations on different carbon sources. The small deviation can be explained by kinetic or genetic regulation within the metabolism, which is not accounted for in the model [17].

## Conclusion

A strategy for the improved annotation of the genome sequence of *A. oryzae *was developed. Using our assembled EST library, 1,046 EST sequences (about 12% of 9,038 EST sequences) were discovered as newly predicted genes and about 75% (6,773 of 9,038 EST sequences) were used to validate previously annotated genes. This indicates that the developed annotation strategy is a very useful approach for gene prediction. Applying a combination of various bioinformatics tools and databases, this annotation strategy was successfully applied for function assignment of genes. A high number of newly predicted genes were assigned with 398 new putative functions, and with new putative functions to 1,469 proteins previously annotated as hypothetical proteins. Therefore our analysis results in a substantially reduced number of hypothetical proteins. In particular, more enzyme-encoding genes could be assigned functions and this led to filling of 210 missing enzymes in the metabolic network. Applying the enhanced annotated genome, biochemical pathway databases, other related metabolic models, and the literature, a metabolic network was reconstructed. The network contains 729 enzymes, 1,314 enzyme-encoding genes (10% of 13,120 total predicted genes), 1,073 metabolites and 1,846 (1,053 unique) biochemical reactions. The 1,053 unique reactions are distributed into different compartments, with 831 reactions located in the cytosol, 173 reactions located in the mitochondria, 19 reactions located in the perosixome, and 30 reactions located in the extracellular space. Transport reactions between the different compartments and the extracellular space represents 281 (161 unique) reactions. This metabolic network was formulated to a stoichiometric model. The model was applied for Flux Balance Analysis (FBA) to obtain the flux distributions corresponding to maximized growth. A physiological study on different carbon sources of *A. oryzae *was performed to validate the genome-scale model, and the model is found to accurately predict the maximum specific growth rate and the biomass yield on different carbon sources. This indicates that the *A. oryzae *metabolic model is able to simulate the phenotypic behavior and the model will hereby serve as an important resource for gaining further insight into our understanding of the important cell factory *A. oryzae*.

## Methods

**Figure 7:**
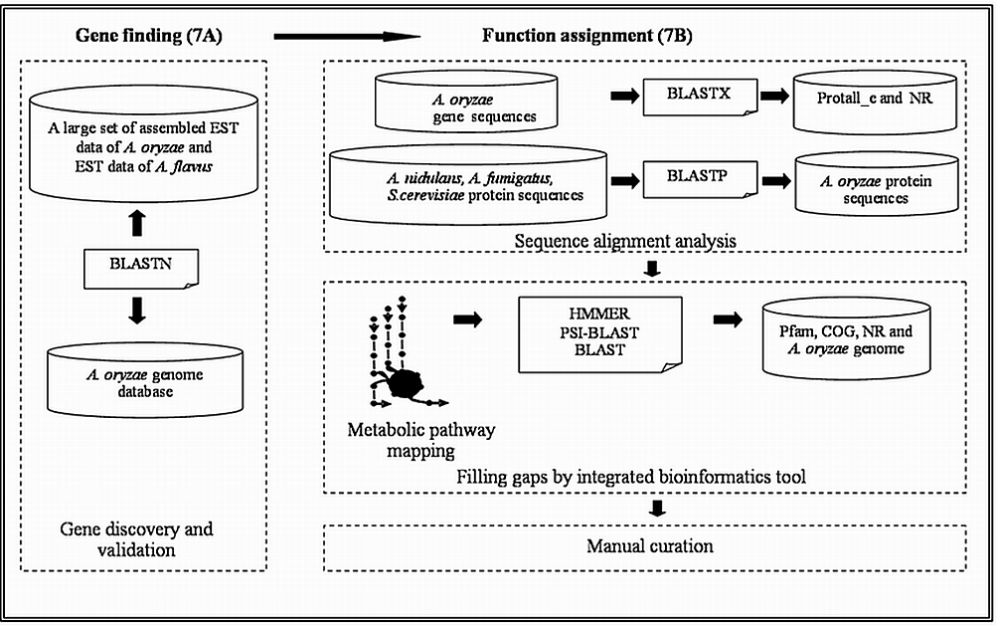
**Overview of annotation process of *A. oryzae *genome**. Illustration of the annotation process, which is divided into two steps, namely gene finding (Figure 7A) and function assignment (Figure 7B).

An overview of the approach employed here for improved genome annotation of *A. oryzae *is depicted in Figure 7. For gene discovery and validation, we constructed EST library and performed sequencing as well as assembly as described in following section.

### EST library construction

The EST sequences of *A. oryzae *strain A1560 were constructed from a normalized library and an un-normalized library. The normalized library was constructed by inserting cDNA of *A. oryzae *in pCMV-Sport6 plasmids between the *Mlu*I and the *Not*I sites (Vector – *Not*I – poly A (3' of insert) – 5' of insert – *Mlu*I – *Sal*I- vector). The plasmids were amplified in *Escherichia coli *EMDH10B-TONA (a recA strain). The un-normalized library was made by inserting cDNA of *A. oryzae *between the *EcoR1 *and *NotI *sites in the vector pYES2. The plasmids were amplified in *E. coli *DH10B.

### EST sequencing and assembly

The EST sequences were generated by sequencing on ABI 377 and ABI 3700 instruments from Applied Biosystems using BigDye terminators version 1 and 2. In total 23,072 EST sequences were produced. Quality clipping, vector removal, *E. coli *contamination removal and assembly were done with the phredPhrap package [39]. The sequences were assembled into 9,038 EST contigs.

### Genome annotation process

The strategy of gene finding as shown in Figure 7A was carried out based on our assembled EST sequences of *A. oryzae *(see Additional file 1, also available online in Genbank database under accession number "EY424375–433412") together with public EST data of *A. flavus *[22]. Our assembled EST data of *A. oryzae *were compared to the genes previously identified [8] in the genome of *A. oryzae *strain RIB 40 by BLASTN [18]. The purpose of this comparison was to validate genes that were already annotated and to discover new genes that had not been annotated by Machida et al [8]. The 9,038 EST sequences were classified into four categories as outlined in Additional file 3 and described as follows. All sequences shorter than 300 bases were discarded from the analysis. If the length of an EST sequence was over 500 bps and the highest ranking hit had a score lower than 50 bits, then the EST sequence was categorized as a sequence that served as a newly predicted gene. If the length of the EST sequence was over 300 bps and the highest ranking hit had a score over 100 bits, then the EST sequence was categorized as validating an earlier identified gene [8]. If the highest ranking hit had a score lower than 100 bits, the EST sequence was classified as weakly validating a gene [8]. In the effort to predict new genes in the *A. oryzae *genome, *A. flavus *EST data from the TIGR database [22] was also used. The cut-off for gene discovery and validation was selected to be the identical as with our assembled EST data of *A. oryzae*. After performing gene finding, assignment of protein function was done. The main principle was performed based on sequence alignment analysis, metabolic pathway mapping, filling the gaps by integrated bioinformatics tool and lastly manual curation. The sequence alignment was done to assign putative function to newly predicted genes by BLASTX [18]. The newly predicted gene was searched against the NR protein database [28] and Protall_e protein database [Unpublished]. The assignment of putative protein function was transferred if the alignment length of the highest ranking hit was over 50 amino acids and the identity over 25%. The sequence alignment was done through pairwise comparison of protein sequences by BLASTP [18] between *A. oryzae *and other related fungi (i.e. *A. nidulans *strain FGSC-A4, *A. fumigatus *strain Af293, *S. cerevisiae *strain S288c) as shown in Figure 7B. The criteria for similarity searching were alignment length (bps) and identity (%), with the parameters depending on the type of fungus used for the comparison [40]. An estimated suitable cut-off for *S. cerevisiae *was an alignment length above 100 bps and an identity higher than 40%. For other related *Aspergillus *species, the cut-off was an alignment length above 200 bps and an identity higher than 40%. All cut-off values were determined by using sequences with known protein functions. After finishing the annotation process, the metabolic network of *A. oryzae *was reconstructed. At the beginning, an initial metabolic reaction list for *A. oryzae *was constructed by combination of *S. cerevisiae *[13], *A. nidulans *[14], and *A. niger *[15,16] metabolic models. In addition, data collection from metabolic pathway databases, such as KEGG [41] and BioCyc [42], of other organisms was integrated into this reaction list. The improved annotated genomic data (i.e. enzyme-encoding genes, enzyme functions, and EC numbers) were then mapped into the reaction list. In order to visualize all the metabolic reactions, overall metabolic map was drawn (see Figure 4 and Additional file 5 for full size). The improved annotated data were placed onto this map. At the end, gaps that existed in the metabolic network were then filled using an integrated bioinformatics tool that allowed for automatic searching for specific enzyme functions. Finally, manual curation of the model was done for finalizing the reconstruction process.

### Metabolic network reconstruction

The metabolic network reconstruction aimed at representing the whole metabolism of *A. oryzae*, which consists of primary catabolism of carbohydrates, biosynthesis of amino acids, nucleotides, lipids, cofactors and production of Gibbs free energy required for biosynthesis, as well as of secondary metabolism. Combination of different types of information was essential to carry out a solid reconstruction. Information was collected from the improved annotated data of *A. oryzae*, biochemical pathways, publications on specific enzymes, online protein databases (e.g. Swiss-Prot database [43]) and also literature. In addition, there was physiological evidence for the presence of a reaction or pathway in *A. oryzae*, e.g. when there was information of presence of a specific enzyme activity or presence of a pathway involved in consumption of a given substrate or formation of a given metabolic product, then the underlying reaction was added to the model, even if there was no annotated gene supporting the presence of the reaction. In the processes of stoichiometry for cofactors as well as the information on reversibility or irreversibility for each reaction, these were verified and added as information into the reconstructed network. Different cellular compartments were considered and consequently biochemical reactions were distributed into four different compartments: the extracellular space, the cytosol, the mitochondria, and the peroxisome [44]. Identification of localization of each biochemical reaction was analyzed according to enzyme localization, which was performed by applying protein localization predictors. Herein, pTARGET [45] and CELLO [46] were selected to predict sub-cellular protein localization because they contain databases of known eukaryotic protein localizations. If there is no information on localization of a biochemical reaction or its corresponding enzyme, then by default this reaction was considered to occur in the cytosol. In addition, the reconstructed metabolic network included transport steps between the different intracellular compartments and between the cell and the environment.

### Modeling and simulation based Flux Balance Analysis (FBA)

After the metabolic network was reconstructed, this was transformed into a mathematical framework to perform Flux Balance Analysis (FBA) [47]. This approach is based on conservation of mass under steady-state conditions. This conversion requires stoichiometry of metabolic pathways, metabolic demands and a few specific parameters. An optimal flux distribution can be obtained within the feasible region by using linear programming [48]. A reaction is selected as an objective function that is to be maximized or minimized. For physiologically meaningful results, the objective functions must be defined as the ability to produce the required components of cellular biomass for a specified uptake rate of a selected carbon source. By maximizing the flux towards biomass formation, a flux is obtained for each reaction in the metabolic network.

### Model validation of *A. oryzae *by physiological study on different carbon sources

Model validation is an important step in the reconstruction process. In this study, the model was validated by simulating the rate of biomass formation on different carbon sources in batch experiments. Here the uptake rate of the carbon source was given as input to the simulations. Different carbon sources namely glucose (C6), maltose (C12), glycerol (C3) and xylose (C5), which were selected as they result in widely different physiological responses and parameters. The strain used for generating these data was *A. oryzae *wild type strain A1560, which was obtained from Novozymes A/S, Denmark. Three biological replicates were done for each carbon source. The fermentations were performed using an in-house fermenter with a working volume of 1.2 L, and operated at 34°C and pH was kept constant at 6 by adding 10% of H_3_PO_4 _or 10% NH_3 _solution. The aeration flow rate was set at 1.2 L/min. The stirrer speed was controlled at 800 rpm for the first 4 hrs and later increased to 1100 rpm. The dissolved oxygen tension was initially calibrated at 100%. The concentrations of oxygen and carbon dioxide in the exhaust gas were measured by a gas analyzer (Magnos 4G for O_2_, Uras 3G for CO_2_, Hartmann & Braun, Germany). Biomass dry weight measurements were done as follows: A sample was filtered using nitrocellulose filters (pore size 0.45 μm, Munktell, Sweden), and the filter cake was therefore dried at 110°C overnight. Hereafter the filter was placed in a dessicator overnight, and subsequently, weighed. In addition, the extracellular concentration of sugars, organic acids, and polyols were measured by using high-performance liquid-chromatography (HPLC) on an Aminex HPX-87H, 300 mm*7.8 mm column. The column was kept at 45°C and eluted at 0.6 ml/min with 5 mM H_2_SO_4_.

## Authors' contributions

WV carried out the improved annotation, performed the genome scale modeling and wrote the manuscript. PO performed the EST sequencing project, participated in the EST and gene mapping and supervised the bioinformatics work. KH supervised the fermentation process. SK provided the EST and gene mapping and supervised the bioinformatics work. JN supervised the whole work and assisted in manuscript preparation. All authors read and approved the final manuscript.

## Supplementary Material

Additional file 1 The assembled EST sequences of *A. oryzae*. The FASTA file provides that 9038 assembled EST sequences. The title name of each sequence indicates dbEST ID, User ID and Genbank accession number. Click here for file

Additional file 2 EST and gene mapping on *A. oryzae *genome. The ZIP file provides that 48 separated files with Perl Scalable Vector Graphics. Click here for file

Additional file 3 Improved annotated data in *A. oryzae *genome. This file is created to extract gene and EST list existed on genomic map (Additional file 2). Click here for file

Additional file 4 A reconstructed metabolic network of *A. oryzae*. A list of all metabolic reactions is shown with EC numbers, enzyme names, gene names and annotation methods. Besides, a list of metabolite abbreviations and biomass compositions are shown also. Click here for file

Additional file 5 Metabolic map of *A. oryzae *in Scalable Vector Graphics. EC numbers are shown for all reactions in the map. A list of abbreviations for the metabolite-names is available in Additional file 4. Click here for file
