# Supplementary material for: Improved annotation through genome-scale metabolic modeling of Aspergillus oryzae
Source: BMC Genomics. 2008 May 23;9:245. doi: 10.1186/1471-2164-9-245 (PMC2413144; doi:10.1186/1471-2164-9-245)
Supplement: Additional file 5 — Metabolic map of A. oryzae in Scalable Vector Graphics. EC numbers are shown for all reactions in the map. A list of abbreviations for the metabolite-names is available in Additional file 4. [file 1471-2164-9-245-S5.pdf]

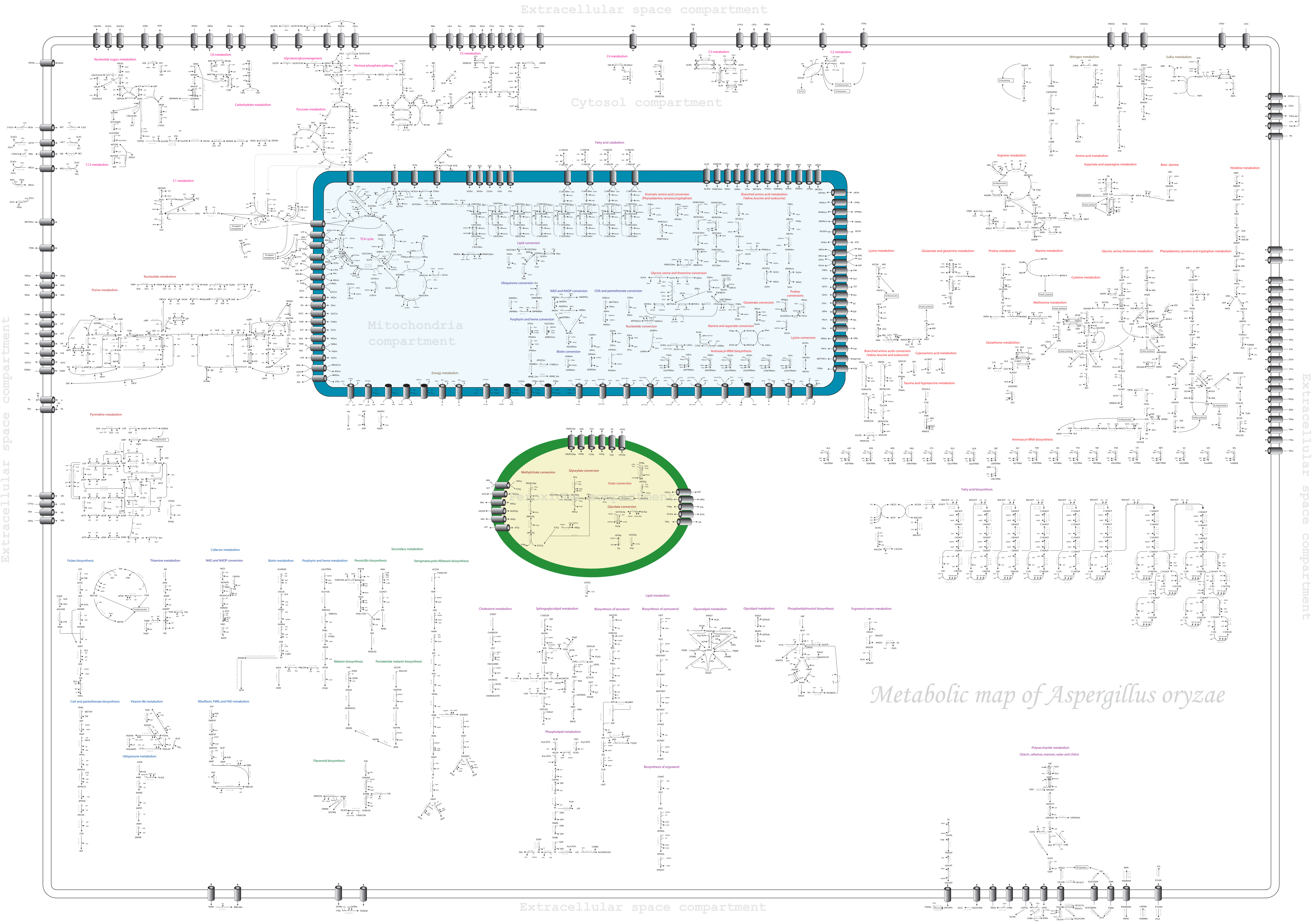
Extracellular space compartment
Cytosol compartment
Mitochondria compartment
Extracellular space compartment
Extracellular space compartment
Extracellular space compartment
Nucleotide sugars metabolism
C6 metabolism
Glycolysis/gluconeogenesis
Pentose phosphate pathway
C5 metabolism
C4 metabolism
C3 metabolism
C2 metabolism
Nitrogen metabolism
Sulfur metabolism
Carbohydrate metabolism
Pyruvate metabolism
C12 metabolism
C1 metabolism
Fatty acid catabolism
Arginine metabolism
Amino acid metabolism
Aspartate and asparagine metabolism
Beta- alanine
Histidine metabolism
TCA cycle
Lipid conversion
Aromatic amino acid conversion (Phenylalanine, tyrosine, tryptophan)
Branched amino acid metabolism (Valine, leucine and isoleucine)
Nucleotide metabolism
Purine metabolism
Ubiquinone conversion
NAD and NADP conversion
COA and pantothenate conversion
Glycine, serine and threonine conversion
Glutamate conversion
Proline conversions
Porphyrin and heme conversion
Nucleotide conversion
Alanine and aspartate conversion
Lysine conversion
Biotin conversion
Aminoacyl-tRNA biosynthesis
Energy metabolism
Lysine metabolism
Glutamate and glutamine metabolism
Proline metabolism
Alanine metabolism
Glycine, serine, threonine metabolism
Phenylalanine, tyrosine and tryptophan metabolism
Cysteine metabolism
Methionine metabolism
Glutathione metabolism
Branched amino acids conversion (Valine, leucine and isoleucine)
Cyanoamino acid metabolism
Taurine and hypotaurine metabolism
Aminoacyl-tRNA biosynthesis
Pyrimidine metabolism
Methylcitrate conversion
Glyoxylate conversion
Urate conversion
Glycolate conversion
Fatty acid biosynthesis
Cofactor metabolism
Secondary metabolism
Folate biosynthesis
Thiamine metabolism
NAD and NADP conversion
Biotin metabolism
Porphyrin and heme metabolism
Penicillin biosynthesis
Sterigmatocystin/Aflatoxin biosynthesis
Lipid metabolism
Cholesterol metabolism
Sphingoglycolipid metabolism
Biosynthesis of lanosterol
Biosynthesis of zymosterol
Glycerolipid metabolism
Glycolipid metabolism
Phosphatidylinositol biosynthesis
Ergosterol esters metabolism
Melanin biosynthesis
Pentaketide melanin biosynthesis
CoA and pantothenate biosynthesis
Vitamin B6 metabolism
Riboflavin, FMN and FAD metabolism
Ubiquinone metabolism
Flavonoid biosynthesis
Phospholipid metabolism
Biosynthesis of ergosterol
Polysaccharide metabolism
(Starch, cellulose, mannan, xylan and chitin)
Metabolic map of Aspergillus oryzae
