# Supplementary material for: Improved annotation through genome-scale metabolic modeling of Aspergillus oryzae
Source: BMC Genomics. 2008 May 23;9:245. doi: 10.1186/1471-2164-9-245 (PMC2413144; doi:10.1186/1471-2164-9-245)
Supplement: Additional file 4 — A reconstructed metabolic network of A. oryzae. A list of all metabolic reactions is shown with EC numbers, enzyme names, gene names and annotation methods. Besides, a list of metabolite abbreviations and biomass compositions are shown also. [file 1471-2164-9-245-S4.pdf]

A reconstructed metabolic network of *Aspergillus oryzae*

Additional file 4

List of Supplementary Tables

1. A reconstructed metabolic network of *A. oryzae* 3
2. List of metabolite abbreviations in *A. oryzae* model 97
3. Macromolecular composition (Biomass formation) of *A. oryzae* 118
4. Protein composition of *A. oryzae* 119
5. DNA composition of *A. oryzae* 120
6. RNA composition of *A. oryzae* 121
7. Lipid composition of *A. oryzae* 122
8. Triacylglycerol composition of *A. oryzae* 123
9. Phospholipid composition of *A. oryzae*. 124
10. Free fatty acid composition of *A. oryzae* 125

References 126

Suppl. Table 1: A reconstructed metabolic network of *A. oryzae*

Description: Suppl. Table 1 contains the reconstructed metabolic network of *A. oryzae*. The details are described by gene name (Column1), enzyme name (Column 2), EC number (Column3) and biochemical reaction (Column4). The last column named "Annotation method" shows which strategy used for improved annotation in each gene list. Abbreviations of the names of the metabolites are found in Suppl. Table 2.

Suppl. Table 1 A reconstructed metabolic network of *A. oryzae*.

Gene name	Enzyme name	EC number	Biochemical reaction	Annotation method
**# 1. Carbohydrate metabolism**				
**# Glucose conversion**				
No_Gene	Spontaneous conversion	No_EC	DGLCe <-> 1/3 GLCe + 2/3 bDGLCe	NONE
No_Gene	Spontaneous conversion	No_EC	DGLC <-> 1/3 GLC + 2/3 bDGLC	NONE
AO090020000042	Aldose 1-epimerase	5.1.3.3	GLC <-> bDGLC	BLASTP
AO090012000809	Aldose 1-epimerase	5.1.3.3	GLC <-> bDGLC	BLASTP
AO090023000671	Aldose 1-epimerase	5.1.3.3	GLC <-> bDGLC	BLASTP
**# Glycolysis/Gluconeogenesis**				
AO090001000710	Hexokinase	2.7.1.1	ATP + GLC -> ADP + G6P	BLASTP
AO090005000083	Hexokinase	2.7.1.1	ATP + GLC -> ADP + G6P	BLASTP
AO090012000259	Hexokinase	2.7.1.1	ATP + GLC -> ADP + G6P	BLASTP
AO090003001508	Hexokinase	2.7.1.1	ATP + GLC -> ADP + G6P	BLASTP
AO090005000003	Hexokinase	2.7.1.1	ATP + GLC -> ADP + G6P	BLASTP
AO090120000109	Glucokinase	2.7.1.2	ATP + bDGLC -> ADP + bDG6P	BLASTP
AO090113000120	Glucose-6-phosphatase	3.1.3.9	G6P + H2O -> GLC + PI	BLASTP
AO090113000120	Glucose-6-phosphatase	3.1.3.9	bDG6P + H2O -> bDGLC + PI	BLASTP
AO090011000659	Glucose-6-phosphate isomerase	5.3.1.9	G6P <-> F6P	BLASTP
AO090011000659	Glucose-6-phosphate isomerase	5.3.1.9	bDG6P <-> F6P	BLASTP
AO090011000659	Glucose-6-phosphate isomerase	5.3.1.9	G6P <-> bDG6P	BLASTP
AO090003001390	Phosphofructokinase	2.7.1.11	ATP + F6P -> ADP + FDP	BLASTP
AO090003000725	Fructose-bisphosphate aldolase	4.1.2.13	FDP <-> T3P2 + T3P1	BLASTP
AO090010000514	Fructose-bisphosphate aldolase	4.1.2.13	FDP <-> T3P2 + T3P1	BLASTP
AO090026000543	Triosephosphate isomerase	5.3.1.1	T3P2 <-> T3P1	BLASTP
AO090120000493	Triosephosphate isomerase	5.3.1.1	T3P2 <-> T3P1	BLASTP
AO090003001322	Glyceraldehyde-3-phosphate dehydrogenase	1.2.1.12	T3P1 + PI + NAD <-> 13PDG + NADH	BLASTP
AO090020000265	Glyceraldehyde-3-phosphate dehydrogenase	1.2.1.12	T3P1 + PI + NAD <-> 13PDG + NADH	BLASTP
AO090011000414	Glyceraldehyde-3-phosphate dehydrogenase	1.2.1.12	T3P1 + PI + NAD <-> 13PDG + NADH	BLASTP
AO090038000395	Phosphoglycerate kinase	2.7.2.3	ADP + 13PDG <-> ATP + 3PG	BLASTP
AO090005001300	Phosphoglycerate mutase	5.4.2.1	3PG <-> 2PG	BLASTP

Gene name	Enzyme name	EC number	Biochemical reaction	Annotation method
AO090120000145	Phosphoglycerate mutase	5.4.2.1	3PG <-> 2PG	BLASTP
AO090011000152	Phosphoglycerate mutase	5.4.2.1	3PG <-> 2PG	BLASTP
ZY077820	Phosphoglycerate mutase	5.4.2.1	3PG <-> 2PG	EST
AO090003000055	Enolase	4.2.1.11	2PG <-> PEP + H2O	BLASTP
AO090005001556	Pyruvate kinase	2.7.1.40	ADP + PEP -> ATP + PYR	BLASTP
AO090003001089	Fructose-1,6-bisphosphatase	3.1.3.11	FDP + H2O -> F6P + PI	BLASTP
AO090003000174	Phosphoenolpyruvate carboxykinase (ATP)	4.1.1.49	ATP + OA -> ADP + PEP + CO2	BLASTP
AO090010000668	NADP-dependent alcohol dehydrogenase	1.1.1.2	ETH + NADP <-> ACAL + NADPH	BLASTP
AO090005001358	NADP-dependent alcohol dehydrogenase	1.1.1.2	ETH + NADP <-> ACAL + NADPH	BLASTP
AO090023000460	NADP-dependent alcohol dehydrogenase	1.1.1.2	ETH + NADP <-> ACAL + NADPH	BLASTP
AO090003000751	NADP-dependent alcohol dehydrogenase	1.1.1.2	ETH + NADP <-> ACAL + NADPH	BLASTP
AO090009000634	NAD-dependent alcohol dehydrogenase	1.1.1.1	ETH + NAD <-> ACAL + NADH	BLASTP
AO090005000125	NAD-dependent alcohol dehydrogenase	1.1.1.1	ETH + NAD <-> ACAL + NADH	BLASTP
AO090012000375	NAD-dependent alcohol dehydrogenase	1.1.1.1	ETH + NAD <-> ACAL + NADH	BLASTP
AO090038000108	NAD-dependent alcohol dehydrogenase	1.1.1.1	ETH + NAD <-> ACAL + NADH	BLASTP
AO090003001407	NAD-dependent alcohol dehydrogenase	1.1.1.1	ETH + NAD <-> ACAL + NADH	BLASTP
AO090009000634	NAD-dependent alcohol dehydrogenase	1.1.1.1	ETHm + NADm <-> ACALm + NADHm	BLASTP
AO090005000125	NAD-dependent alcohol dehydrogenase	1.1.1.1	ETHm + NADm <-> ACALm + NADHm	BLASTP
AO090012000375	NAD-dependent alcohol dehydrogenase	1.1.1.1	ETHm + NADm <-> ACALm + NADHm	BLASTP
AO090038000108	NAD-dependent alcohol dehydrogenase	1.1.1.1	ETHm + NADm <-> ACALm + NADHm	BLASTP
AO090003001407	NAD-dependent alcohol dehydrogenase	1.1.1.1	ETHm + NADm <-> ACALm + NADHm	BLASTP
AO090003000290/ AO090012000948/ AO090124000079/ AO090005000436/ AO090005001450/ AO090011000486	Pyruvate dehydrogenase complex	1.2.4.1/ 2.3.1.12/ 1.8.1.4	PYRm + COAm + NADm -> ACCOAm + CO2m + NADHm	BLASTP

Gene name	Enzyme name	EC number	Biochemical reaction	Annotation method
**# Pentose-phosphate pathway**				
AO090005001427	Glucose-6-phosphate 1-dehydrogenase	1.1.1.49	G6P + NADP -> D6PGL + NADPH	BLASTP
AO090005000789	6-phosphogluconolactonase	3.1.1.31	D6PGL + H2O -> D6PGC	BLASTP
AO090003000121	Phosphogluconate dehydrogenase (decarboxylating)	1.1.1.44	D6PGC + NADP -> RL5P + CO2 + NADPH	BLASTP
AO090001000547	Phosphogluconate dehydrogenase (decarboxylating)	1.1.1.44	D6PGC + NADP -> RL5P + CO2 + NADPH	BLASTP
AO090012000303	Ribulose-phosphate 3-epimerase	5.1.3.1	RL5P <-> XUL5P	BLASTP
AO090023000209	Ribose 5-phosphate isomerase	5.3.1.6	RL5P <-> R5P	BLASTP
AO090012000526	Transketolase	2.2.1.1	R5P + XUL5P <-> S7P + T3P1	BLASTP
AO090023000345	Transketolase	2.2.1.1	R5P + XUL5P <-> S7P + T3P1	BLASTP
AO090001000322	Transketolase	2.2.1.1	R5P + XUL5P <-> S7P + T3P1	BLASTP
AO090012000526	Transketolase	2.2.1.1	E4P + XUL5P <-> F6P + T3P1	BLASTP
AO090023000345	Transketolase	2.2.1.1	E4P + XUL5P <-> F6P + T3P1	BLASTP
AO090001000322	Transketolase	2.2.1.1	E4P + XUL5P <-> F6P + T3P1	BLASTP
AO090020000520	Transaldolase	2.2.1.2	S7P + T3P1 <-> E4P + F6P	BLASTP
AO090003000625	Xylulose-5-phosphate phosphoketolase	4.1.2.9	XUL5P + PI <-> ACTP + T3P1 + H2O	BLASTP
GAP	Phosphopentomutase	5.4.2.7	DR1P -> DEORIPI	NONE
AO090138000062	Deoxyribose-phosphate aldolase	4.1.2.4	DEORIPI <-> T3P1 + ACAL	BLASTP
AO090020000341	Deoxyribose-phosphate aldolase	4.1.2.4	DEORIPI <-> T3P1 + ACAL	BLASTP
AO090038000092	Ribokinase	2.7.1.15	ATP + RIB -> ADP + R5P	BLASTP
AO090102000368	Ribokinase	2.7.1.15	ATP + RIB -> ADP + R5P	BLASTP
AO090003001003	Gluconokinase	2.7.1.12	GLCNT + ATP -> D6PGC + ADP	BLASTP
AO090003000746	Phosphoglucomutase	5.4.2.2	R5P <-> R1P	BLASTP
AO090011000487	Phosphoglucomutase	5.4.2.2	R5P <-> R1P	BLASTP
AO090001000101	Phosphoglucomutase	5.4.2.2	R5P <-> R1P	BLASTP
AO090012000711	Fructose-6-phosphate phosphoketolase	4.1.2.22	F6P + PI -> H2O + ACTP + E4P	GFAOP
AO090003000625	Fructose-6-phosphate phosphoketolase	4.1.2.22	F6P + PI -> H2O + ACTP + E4P	GFAOP

Gene name	Enzyme name	EC number	Biochemical reaction	Annotation method
**# Tricarboxylic acid cycle and glyoxylate/Dicarboxylate metabolism**				
AO090010000170	Citrate synthase	2.3.3.1	ACCOAm + H2Om + OAm <-> CITm + COAm	BLASTP
AO090012000318	Citrate synthase	2.3.3.1	ACCOAm + H2Om + OAm <-> CITm + COAm	BLASTP
AO090102000627	Citrate synthase	2.3.3.1	ACCOAm + H2Om + OAm <-> CITm + COAm	BLASTP
AO090005000045	Aconitate hydratase	4.2.1.3	CITm <-> ACOm + H2Om	BLASTP
AO090001000484	Aconitate hydratase	4.2.1.3	CITm <-> ACOm + H2Om	BLASTP
AO090001000642	Aconitate hydratase	4.2.1.3	CITm <-> ACOm + H2Om	BLASTP
AO090003000415	Aconitate hydratase	4.2.1.3	CITm <-> ACOm + H2Om	BLASTP
ZY085753	Aconitate hydratase	4.2.1.3	CITm <-> ACOm + H2Om	EST
AO090005000045	Aconitate hydratase	4.2.1.3	ACOm + H2Om <-> ICITm	BLASTP
AO090001000484	Aconitate hydratase	4.2.1.3	ACOm + H2Om <-> ICITm	BLASTP
AO090001000484	Aconitate hydratase	4.2.1.3	ACOm + H2Om <-> ICITm	BLASTP
AO090003000415	Aconitate hydratase	4.2.1.3	ACOm + H2Om <-> ICITm	BLASTP
ZY085753	Aconitate hydratase	4.2.1.3	ACOm + H2Om <-> ICITm	EST
AO090003000008/ AO090012000629	NAD-dependent isocitrate dehydrogenase	1.1.1.41	ICITm + NADm -> AKGm + CO2m + NADHm	BLASTP
AO090005001404	NADP-dependent isocitrate dehydrogenase	1.1.1.42	ICITm + NADPm -> AKGm + CO2m + NADPHm	BLASTP
AO090005001404	NADP-dependent isocitrate dehydrogenase	1.1.1.42	ICIT + NADP -> AKG + CO2 + NADPH	BLASTP
AO090005001404	NADP-dependent isocitrate dehydrogenase	1.1.1.42	ICITp + NADPp -> AKGp + CO2p + NADPHp	BLASTP
AO090003001055/ AO090005001170/ AO090005001171/ AO090005001173/ AO090005001174/ AO090020000008/ AO090011000486	Dihydrolipoamide S-succinyltransferase complex	1.2.4.2/ 2.3.1.61/ 1.8.1.4	AKGm + NADm + COAm -> SUCCOAm + CO2m + NADHm + Hm	BLASTP
AO090003000943	Succinyl-CoA:alpha-ketoacid-CoA transferase	2.8.3.5	SUCCOAm + ACTACm -> SUCCm + AACCOAm	GFAOP
AO090009000195	Succinyl-CoA:alpha-ketoacid-CoA transferase	2.8.3.5	SUCCOAm + ACTACm -> SUCCm + AACCOAm	GFAOP
AO090124000071	Succinyl-CoA:alpha-ketoacid-CoA transferase	2.8.3.5	SUCCOAm + ACTACm -> SUCCm + AACCOAm	GFAOP

Gene name	Enzyme name	EC number	Biochemical reaction	Annotation method
AO090009000644	Succinate-CoA ligase (GDP-forming)	6.2.1.4	GDPm + PIm + SUCCOAm <-> GTPm + SUCCm + COAm	BLASTP
AO090038000330/	Succinate-CoA ligase (ADP-forming)	6.2.1.5	ADPm + PIm + SUCCOAm <-> ATPm + SUCCm + COAm	BLASTP
AO090206000040/<br>AO090023000354<br>AO090020000415/<br>AO090020000596/<br>AO090010000505/<br>ZY029275	Succinate dehydrogenase (flavoprotein subunit)	1.3.5.1	SUCCm + Qm <-> FUMm + QH2m	EST, BLASTP
AO090003000831	Fumarate reductase (flavoprotein subunit)	1.3.99.1	FUM + FADH2m -> SUCC + FADm	BLASTP
AO090005000592	Fumarate reductase (flavoprotein subunit)	1.3.99.1	FUMm + FADH2m -> SUCCm + FADm	BLASTP
AO090120000133	Fumarate hydratase	4.2.1.2	FUMm + H2Om <-> MALm	BLASTP
AO090005000438	NAD-dependent malate dehydrogenase	1.1.1.37	MALm + NADm <-> OAm + NADHm	BLASTP
AO090701000013	NAD-dependent malate dehydrogenase	1.1.1.37	MAL + NAD <-> OA + NADH	BLASTP
AO090009000285	Citrate lyase	4.1.3.6	CIT <-> AC + OA	GFAOP
AO090023000205/<br>AO090023000206	ATP citrate synthase	2.3.3.8	ATP + CIT + COA -> ADP + PI + ACCOA + OA	BLASTP
AO090009000557	Malate synthase	2.3.3.9	ACCOAp + H2Op + GLXp -> MALp + COAp	BLASTP
AO090009000219	Isocitrate lyase	4.1.3.1	ICITp -> GLXp + SUCCp	BLASTP
AO090701000233	Carboxyphosphonoenolpyruvate phosphonomutase	3.7.1.1	OA + H2O -> OXAL + AC	GFAOP
AO090701000316	Carboxyphosphonoenolpyruvate phosphonomutase	3.7.1.1	OA + H2O -> OXAL + AC	GFAOP
AO090005001508	Carboxyphosphonoenolpyruvate phosphonomutase	3.7.1.1	OA + H2O -> OXAL + AC	GFAOP
AO090005000078	Oxalate decarboxylase	4.1.1.2	OXAL -> FOR + CO2	BLASTP
AO090010000631	Oxalate decarboxylase	4.1.1.2	OXAL -> FOR + CO2	BLASTP
AO090011000368	Glyoxylate reductase	1.1.1.79	GLYCOLAp + NADPp -> GLXp + NADPHp	BLASTP
AO090003001032	Glyoxylate reductase	1.1.1.79	GLYCOLAp + NADPp -> GLXp + NADPHp	BLASTP
AO090023000508	Glyoxylate reductase	1.1.1.79	GLYCOLAp + NADPp -> GLXp + NADPHp	BLASTP
AO090010000085	Glycolate oxidase	1.1.3.15	GLYCOLAp + O2p -> GLXp + H2O2p	GFAOP
AO090010000623	Glycolate oxidase	1.1.3.15	GLYCOLAp + O2p -> GLXp + H2O2p	GFAOP
AO090138000044	Glycolate oxidase	1.1.3.15	GLYCOLAp + O2p -> GLXp + H2O2p	GFAOP
AO090026000735	Glycolate oxidase	1.1.3.15	GLYCOLAp + O2p -> GLXp + H2O2p	GFAOP
GAP	Glycolaldehyde dehydrogenase	1.2.1.21	GLALp + NADp + H2Op <-> GLYCOLAp + NADHp	NONE

Gene name	Enzyme name	EC number	Biochemical reaction	Annotation method
AO090012000231	Hydroxypyruvate reductase	1.1.1.29	Gp + NADp <-> HPYRp + NADHp	GFAOP
AO090003000929	Tartrate dehydrogenase	1.1.1.93	TAR + NAD <-> OXGLY + NADH	BLASTP
AO090020000210	Tartrate dehydrogenase	1.1.1.93	TAR + NAD <-> OXGLY + NADH	BLASTP
GAP	Oxaloglycolate reductase (decarboxylating)	1.1.1.92	OXGLY + NAD -> G + CO2 + NADH	NONE
**# Pyruvate metabolism**				
AO090003000661	Pyruvate decarboxylase	4.1.1.1	PYR -> ACAL + CO2	BLASTP
AO090012000752	Pyruvate decarboxylase	4.1.1.1	PYR -> ACAL + CO2	BLASTP
AO090010000261	Pyruvate decarboxylase	4.1.1.1	PYR -> ACAL + CO2	BLASTP
AO090011000272	Pyruvate decarboxylase	4.1.1.1	PYR -> ACAL + CO2	BLASTP
AO090124000047	Pyruvate decarboxylase	4.1.1.1	PYR -> ACAL + CO2	BLASTP
AO090701000373	S-(hydroxymethyl)glutathione dehydrogenase	1.1.1.284	HYGTA + NAD -> FGT + NADH	BLASTP
GAP	S-(hydroxymethyl)glutathione synthase	4.4.1.22	FALD + RGT <-> HYGTA	NONE
ZY097848	Methylglyoxal synthase	4.2.3.3	T3P2 -> MTHGXL + PI	EST
AO090010000424	NADP-dependent methylglyoxal reductase	1.1.1.283	MTHGXL + NADPH <-> LACAL + NADP	BLASTP
AO090026000741	Lactaldehyde dehydrogenase	1.2.1.22	LACAL + NAD + H2O -> LAC + NADH	BLASTP
AO090023000697	Lactaldehyde dehydrogenase	1.2.1.22	LACAL + NAD + H2O -> LAC + NADH	BLASTP
AO090023000697	Lactaldehyde dehydrogenase	1.2.1.22	LACALm + NADm + H2Om -> LACm + NADHm	BLASTP
AO090003000140	Lactoylglutathione lyase	4.4.1.5	RGT + MTHGXL <-> LGT	BLASTP
AO090026000258	Lactoylglutathione lyase	4.4.1.5	RGT + MTHGXL <-> LGT	BLASTP
AO090120000408	Hydroxyacylglutathione hydrolase	3.1.2.6	LGT + H2O -> LAC + RGT	BLASTP
AO090003001112	Acetyl-coenzyme A synthetase	6.2.1.1	ATP + AC + COA -> AMP + PPI + ACCOA	BLASTP
AO090003001112	Acetyl-coenzyme A synthetase	6.2.1.1	ATPp + ACp + COAp -> AMPp + PPIp + ACCOAp	BLASTP
AO090003001112	Acetyl-coenzyme A synthetase	6.2.1.1	ATPm + ACm + COAm -> AMPm + PPIm + ACCOAm	BLASTP
AO090023000467	NAD-dependent aldehyde dehydrogenase	1.2.1.3	ACAL + NAD + H2O -> NADH + AC	BLASTP
AO090009000417	NAD-dependent aldehyde dehydrogenase	1.2.1.3	ACALm + NADm + H2Om -> NADHm + ACm	BLASTP
AO090009000417	NAD-dependent aldehyde dehydrogenase	1.2.1.3	ACAL + NAD + H2O -> NADH + AC	BLASTP
AO090001000162	NAD-dependent aldehyde dehydrogenase	1.2.1.3	ACAL + NAD + H2O -> NADH + AC	BLASTP
ZY087055	NAD-dependent aldehyde dehydrogenase	1.2.1.3	ACAL + NAD + H2O -> NADH + AC	EST
AO090009000222	NADP-dependent aldehyde dehydrogenase	1.2.1.5	ACAL + NADP + H2O -> AC + NADPH	BLASTP
AO090005000588	Acetyl-CoA hydrolase	3.1.2.1	ACCOA + H2O -> COA + AC	BLASTP
AO090005000588	Acetyl-CoA hydrolase	3.1.2.1	ACCOAm + H2Om -> COAm + ACm	BLASTP

Gene name	Enzyme name	EC number	Biochemical reaction	Annotation method
AO090005000277	Esterase	3.1.2.12	FGT + H2O <-> RGT + FOR	BLASTP
AO090701000046/ AO090010000579	NAD-dependent formate dehydrogenase	1.2.1.2	FOR + NAD -> CO2 + NADH	BLASTP
AO090701000046/ AO090010000579	NAD-dependent formate dehydrogenase	1.2.1.2	FORm + NADm -> CO2m + NADHm	BLASTP
AO090005000125	NAD-dependent alcohol dehydrogenase	1.1.1.1	METHOL + NAD <-> FALD + NADH	BLASTP
AO090012000375	NAD-dependent alcohol dehydrogenase	1.1.1.1	METHOL + NAD <-> FALD + NADH	BLASTP
AO090009000634	NAD-dependent alcohol dehydrogenase	1.1.1.1	METHOL + NAD <-> FALD + NADH	BLASTP
AO090038000108	NAD-dependent alcohol dehydrogenase	1.1.1.1	METHOL + NAD <-> FALD + NADH	BLASTP
AO090003001407	NAD-dependent alcohol dehydrogenase	1.1.1.1	METHOL + NAD <-> FALD + NADH	BLASTP
AO090023000801	Pyruvate carboxylase	6.4.1.1	ATP + PYR + HCO3 -> ADP + PI + OA	BLASTP
AO090701000666	Pyruvate carboxylase	6.4.1.1	ATPm + PYRm + HCO3m -> ADPm + PIm + OAm	BLASTP
AO090011000876	NADP-dependent malic enzyme	1.1.1.40	MALm + NADPm -> PYRm + CO2m + NADPHm	BLASTP
AO090038000621	NADP-dependent malic enzyme	1.1.1.40	MAL + NADP -> PYR + CO2 + NADPH	BLASTP
AO090038000621	NADP-dependent malic enzyme	1.1.1.38	MAL + NAD -> PYR + CO2 + NADH	BLASTP
AO090003000624	Acetate kinase	2.7.2.1	ADPm + ACTPm <-> ATPm + ACm	BLASTP
AO090023000577	D-lactate dehydrogenase	1.1.1.28	PYR + NADH <-> LAC + NAD	BLASTP
AO090023000871	L-lactate dehydrogenase	1.1.1.27	LLAC + NAD -> PYR + NADH	BLASTP
AO090102000252	Lactic acid dehydrogenase	1.1.2.3	2 FERIm + LLACm -> PYRm + 2 FEROm	BLASTP
AO090003001006	D-Lactate dehydrogenase (cytochrome)	1.1.2.4	2 FERIm + LACm -> PYRm + 2 FEROm	BLASTP
AO090010000369	D-Lactate dehydrogenase (cytochrome)	1.1.2.4	2 FERIm + LACm -> PYRm + 2 FEROm	BLASTP
AO090038000632	D-Lactate dehydrogenase (cytochrome)	1.1.2.4	2 FERIm + LACm -> PYRm + 2 FEROm	BLASTP
AO090020000315	D-Lactate dehydrogenase (cytochrome)	1.1.2.4	2 FERIm + LACm -> PYRm + 2 FEROm	BLASTP
**# Pentose and glucuronate interconversions**				
AO090005000972	UDP-glucose dehydrogenase	1.1.1.22	UDPG + 2 NAD + H2O -> UDPGE + 2 NADH	BLASTP
AO090020000680	UDP-glucuronosyltransferase	2.4.1.17	UDPGE + H2O -> UDP + GLUCRE	GFAOP
AO090038000009	Beta-glucuronidase	3.2.1.31	GLUCRE + H2O -> GLUCN + ETH	GFAOP
AO090026000127	Alpha-glucuronidase	3.2.1.139	GLUCRE + H2O -> GLUCN + ETH	GFAOP
GAP	Glucuronate isomerase	5.3.1.12	GLUCN <-> FRUTN	NONE
AO090011000230	D-mannonate oxidoreductase	1.1.1.57	FRUTN + NADH <-> MANOE + NAD	GFAOP
GAP	Mannonate dehydratase	4.2.1.8	MANOE <-> DEXG + H2O	NONE

Gene name	Enzyme name	EC number	Biochemical reaction	Annotation method
AO090011000374	2-deoxy-D-gluconate 3-dehydrogenase	1.1.1.125	DEXG + NAD <-> DEHXG + NADH	GFAOP
AO090003000277	2-deoxy-D-gluconate 3-dehydrogenase	1.1.1.125	DEXG + NAD <-> DEHXG + NADH	GFAOP
**# Propanoate and butanoate metabolism**				
AO090011000917	Propanoyl CoA synthase	6.2.1.17	PROPm + ATPm + COAm -> AMPm + PPIm + PROPCOAm	BLASTP
GAP	Propionyl-CoA carboxylase	6.4.1.3	ATP + PROPCOA + HCO3 -> ADP + PI + MMCOA	NONE
AO090701000665	Methylmalonyl-CoA decarboxylase	4.1.1.41	MMCOA -> PROPCOA + CO2	BLASTP
GAP	1-aminocyclopropane-1-carboxylate synthase	4.4.1.14	SAM -> ACPC + 5MTA	NONE
AO090701000360	1-aminocyclopropane-1-carboxylate deaminase	3.5.99.7	ACPC + H2O -> OBUT + NH3	BLASTP
AO090009000568	Methylcitrate synthase	2.3.3.5	PROPCOAp + H2Op + OAp -> 2MCITp + COAp	BLASTP
AO090010000155	Methylcitrate dehydratase	4.2.1.79	2MCITp <-> 2MACOp + H2Op	GFAOP
GAP	Methylisocitrate dehydratase	4.2.1.99	2MACOp + H2Op <-> 2MICITp	NONE
AO090120000179	Methylisocitrate lyase	4.1.3.30	2MICITp <-> PYRp + SUCCp	GFAOP
AO090001000376	Acetoacetyl-CoA synthase	6.2.1.16	ATP + ACTAC + COA -> AMP + PPI + AACCOA	BLASTP
AO090005000672	Acetoacetyl-CoA synthase	6.2.1.16	ATP + ACTAC + COA -> AMP + PPI + AACCOA	BLASTP
AO090005001248	Acyl-CoA dehydrogenase	1.3.99.2	CRONYLCOAm + FADm -> C40COAm + FADH2m	GFAOP
AO090206000053	3-hydroxybutyryl-CoA dehydrogenase	1.1.1.157	C4HCOAm + NADPm <-> AACCOAm + NADPHm	BLASTP
**# Inositol phosphate metabolism**				
AO090003001009	Phosphatidylinositol phospholipase C	4.6.1.13	PINS -> MYOCYPI + DAGLY	BLASTP
AO090701000359	Myo-inositol-phosphate synthase	5.5.1.4	G6P -> MI1P	BLASTP
AO090124000088	Inositol monophosphatase	3.1.3.25	MI1P + H2O -> MYOI + PI	GFAOP
AO090038000262	Inositol monophosphatase	3.1.3.25	MI1P + H2O -> MYOI + PI	GFAOP
AO090206000077	Inositol monophosphatase	3.1.3.25	MI1P + H2O -> MYOI + PI	GFAOP
AO090011000613	Myo-inositol oxygenase	1.13.99.1	MYOI + O2 -> GLUCN	BLASTP
AO090010000202	Phytase	3.1.3.8	PYTE -> MYOI + 6 PI	BLASTP
AO090023000692	Phytase	3.1.3.8	PYTE -> MYOI + 6 PI	BLASTP
AO090023000448	Phytase	3.1.3.8	PYTE -> MYOI + 6 PI	BLASTP
AO090023000481	Phytase	3.1.3.8	PYTE -> MYOI + 6 PI	BLASTP
AO090023000764	Phosphoinositide phosphatase	3.1.3.36	D45PI + H2O -> PINS4P + PI	BLASTP

Gene name	Enzyme name	EC number	Biochemical reaction	Annotation method
AO090102000272	Inositol polyphosphate phosphatase	3.1.3.56	TPI + H2O -> MYOBISPI + PI	GFAOP
GAP	Inositol-1,4-bisphosphate 1-phosphatase	3.1.3.57	MYOBISPI + H2O -> MYOI + PI	NONE
AO090011000611	Myo-inositol 2-dehydrogenase	1.1.1.18	MYOI + NAD -> KEMYOI + NADH	BLASTP
AO090011000612	Myo-inositol 2-dehydrogenase	1.1.1.18	MYOI + NAD -> KEMYOI + NADH	BLASTP
**# L-Arabinose/Arabitol and D-Xylose/D,L-Xylulose/Xylitol metabolism**				
AO090012000287	L-xylulose reductase	1.1.1.10	LXUL + NADPH <-> XOL + NADP	BLASTP
AO090020000558	L-xylulose reductase	1.1.1.10	LXUL + NADPH <-> XOL + NADP	BLASTP
AO090103000206	L-xylulose reductase	1.1.1.10	LXUL + NADPH <-> XOL + NADP	BLASTP
AO090020000635	Xylitol dehydrogenase	1.1.1.9	XUL + NADH <-> XOL + NAD	GFAOP
AO090020000676	Xylitol dehydrogenase	1.1.1.9	XUL + NADH <-> XOL + NAD	GFAOP
AO090103000393	Xylitol dehydrogenase	1.1.1.9	XUL + NADH <-> XOL + NAD	GFAOP
AO090003000859	Xylose reductase	1.1.1.21	XYL + NADPH <-> XOL + NADP	GFAOP
AO090023000264	Xylose reductase	1.1.1.21	XYL + NADPH <-> XOL + NADP	GFAOP
AO090009000575	D-arabinitol 4-dehydrogenase	1.1.1.11	AOL + NAD <-> XUL + NADH	BLASTP
AO090005001078	D-arabinitol 4-dehydrogenase	1.1.1.11	AOL + NAD <-> XUL + NADH	BLASTP
AO090023000346	D-arabinitol 4-dehydrogenase	1.1.1.11	AOL + NAD <-> XUL + NADH	BLASTP
AO090009000575	L-arabinitol 4-dehydrogenase	1.1.1.12	LAOL + NAD <-> LXUL + NADH	BLASTP
AO090005001078	L-arabinitol 4-dehydrogenase	1.1.1.12	LAOL + NAD <-> LXUL + NADH	BLASTP
AO090023000346	L-arabinitol 4-dehydrogenase	1.1.1.12	LAOL + NAD <-> LXUL + NADH	BLASTP
AO090012000869	Aldehyde reductase	1.1.1.21	ARAB + NADPH -> AOL + NADP	GFAOP
AO090023000264	Aldehyde reductase	1.1.1.21	ARAB + NADPH -> AOL + NADP	GFAOP
AO090023000394	Aldehyde reductase	1.1.1.21	ARAB + NADPH -> AOL + NADP	GFAOP
AO090020000624	Aldehyde reductase	1.1.1.21	ARAB + NADPH -> AOL + NADP	GFAOP
AO090012000869	Aldehyde reductase	1.1.1.21	LARAB + NADPH -> LAOL + NADP	BLASTP
AO090023000264	Aldehyde reductase	1.1.1.21	LARAB + NADPH -> LAOL + NADP	BLASTP
AO090023000394	Aldehyde reductase	1.1.1.21	LARAB + NADPH -> LAOL + NADP	BLASTP
AO090020000624	Aldehyde reductase	1.1.1.21	LARAB + NADPH -> LAOL + NADP	BLASTP
AO090011000614	D-Arabinose 1-dehydrogenase (NAD(P))	1.1.1.117	ARAB + NAD -> ARABLAC + NADH	BLASTP
AO090009000563	D-Arabinose 1-dehydrogenase (NAD(P))	1.1.1.117	ARAB + NADP -> ARABLAC + NADPH	BLASTP
AO090020000603	D-xylulose kinase	2.7.1.17	ATP + XUL -> ADP + XUL5P	BLASTP
AO090012000526	Formaldehyde transketolase	2.2.1.3	XUL5P + FALD <-> T3P1 + GLYN	BLASTP

Gene name	Enzyme name	EC number	Biochemical reaction	Annotation method
AO090023000345	Formaldehyde transketolase	2.2.1.3	XUL5P + FALD <-> T3P1 + GLYN	BLASTP
AO090001000322	Formaldehyde transketolase	2.2.1.3	XUL5P + FALD <-> T3P1 + GLYN	BLASTP
AO090701000508	Formaldehyde dehydrogenase	1.2.1.46	FALD + NAD + H2O -> FOR + NADH	BLASTP
AO090308000002	Formaldehyde dehydrogenase	1.2.1.46	FALD + NAD + H2O -> FOR + NADH	BLASTP
AO090020000646	D-ribulokinase	2.7.1.47	ATP + RL -> ADP + RL5P	BLASTP
AO090206000019	D-ribulokinase	2.7.1.47	ATP + RL -> ADP + RL5P	BLASTP
AO090020000646	Ribulokinase	2.7.1.16	ATP + LRL -> ADP + LRL5P	BLASTP
AO090206000019	Ribulokinase	2.7.1.16	ATP + LRL -> ADP + LRL5P	BLASTP
AO090009000063	L-ribulose-5-phosphate 4-epimerase	5.1.3.4	LRL5P <-> XUL5P	GFAOP
AO090026000216	L-ribulose-5-phosphate 4-epimerase	5.1.3.4	LRL5P <-> XUL5P	GFAOP
AO090701000461	L-ribulose-5-phosphate 4-epimerase	5.1.3.4	LRL5P <-> XUL5P	GFAOP
**# Gluconic acid/Gluconate metabolism**				
GAP	Glucose dehydrogenase	1.1.99.10	DGLC + FADm -> GLCN15LAC + FADH2m	NONE
AO090005000449	Glucose oxidase	1.1.3.4	bDGLC + O2 <-> GLCN15LAC + H2O2	BLASTP
AO090103000214	Glucose oxidase	1.1.3.4	bDGLCe + O2e <-> GLCN15LACe + H2O2e	BLASTP
AO090124000002	Gluconolactonase	3.1.1.17	GLCN15LACe + H2Oe -> GLCNTe	BLASTP
AO090701000487	Gluconolactonase	3.1.1.17	GLCN15LACe + H2Oe -> GLCNTe	BLASTP
AO090124000002	Gluconolactonase	3.1.1.17	GLCN15LAC + H2O -> GLCNT	BLASTP
AO090701000487	Gluconolactonase	3.1.1.17	GLCN15LAC + H2O -> GLCNT	BLASTP
GAP	1,4-lactonase	3.1.1.25	GALN14LAC + H2O <-> GALNT	NONE
**# Galactose/Galactitol metabolism**				
AO090012000869	Aldehyde reductase	1.1.1.21	GLAC + NADPH <-> GALOL + NADP	BLASTP
AO090020000624	Aldehyde reductase	1.1.1.21	GLAC + NADPH <-> GALOL + NADP	BLASTP
AO090023000264	Aldehyde reductase	1.1.1.21	GLAC + NADPH <-> GALOL + NADP	BLASTP
AO090023000394	Aldehyde reductase	1.1.1.21	GLAC + NADPH <-> GALOL + NADP	BLASTP
AO090003000570	Galactokinase	2.7.1.6	ATP + GLAC -> ADP + GAL1P	BLASTP
AO090026000490	UTP-galactose-1-phosphate uridylyltransferase	2.7.7.10	UTP + GAL1P <-> PPI + UDPGAL	BLASTP
AO090005001490	UDP-glucose 4-epimerase	5.1.3.2	UDPGAL <-> UDPG	BLASTP
AO090010000463	UDP-glucose 4-epimerase	5.1.3.2	UDPGAL <-> UDPG	BLASTP
AO090003001495	UDP-glucose 4-epimerase	5.1.3.2	UDPGAL <-> UDPG	BLASTP
AO090038000558	UDP glucose pyrophosphorylase	2.7.7.9	G1P + UTP <-> UDPG + PPI	BLASTP

Gene name	Enzyme name	EC number	Biochemical reaction	Annotation method
AO090003000746	Phosphoglucomutase	5.4.2.2	G6P <-> G1P	BLASTP
AO090011000487	Phosphoglucomutase	5.4.2.2	G6P <-> G1P	BLASTP
AO090001000101	Phosphoglucomutase	5.4.2.2	G6P <-> G1P	BLASTP
AO090026000490	UDP-galactose-1-phosphate uridylyltransferase	2.7.7.12	UDPG + GAL1P <-> G1P + UDPGAL	BLASTP
AO090011000063	Alpha-galactosidase	3.2.1.22	MELI + H2O -> GLC + GLAC	GFAOP
AO090010000684	Alpha-galactosidase	3.2.1.22	MELI + H2O -> GLC + GLAC	GFAOP
AO090003001305	Alpha-galactosidase	3.2.1.22	MELIe + H2Oe -> GLCe + GLACe	BLASTP
AO090023000151	Alpha-galactosidase	3.2.1.22	MELIe + H2Oe -> GLCe + GLACe	BLASTP
AO090005000217	Alpha-galactosidase	3.2.1.22	MELIe + H2Oe -> GLCe + GLACe	BLASTP
AO090701000400	Beta-fructofuranosidase	3.2.1.26	SUCe + H2Oe -> GLCe + FRUe	BLASTP
AO090701000400	Beta-fructofuranosidase	3.2.1.26	RAFe + H2Oe -> FRUe + MELIe	BLASTP
AO090308000021	Galactose oxidase	1.1.3.9	GLAC + O2 -> GALNT + H2O2	BLASTP
AO090011000020	Galactose oxidase	1.1.3.9	GLAC + O2 -> GALNT + H2O2	BLASTP
AO090009000065	Tagatose-bisphosphate aldolase	4.1.2.40	TDP <-> T3P2 + T3P1	BLASTP
AO090009000324	Tagatose-bisphosphate aldolase	4.1.2.40	TDP <-> T3P2 + T3P1	BLASTP
AO090701000462	Tagatose-bisphosphate aldolase	4.1.2.40	TDP <-> T3P2 + T3P1	BLASTP
AO090003001390	Phosphofructokinase	2.7.1.11	T6P + ATP <-> TDP + ADP	BLASTP
GAP	Tagatose kinase	2.7.1.101	TGE + ATP <-> T6P + ADP	NONE
**# Galactonic acid/Galactonate metabolism**				
AO090102000225	Galactose 1-dehydrogenase	1.1.1.48	GLAC + NAD -> GALN14LAC + NADH	BLASTP
AO090020000363	Galactose 1-dehydrogenase	1.1.1.48	GLAC + NAD -> GALN14LAC + NADH	BLASTP
AO090003000276	Galatonate dehydratase	4.2.1.6	GALNT <-> 2D3DGALT + H2O	BLASTP
AO090001000258	Galatonate dehydratase	4.2.1.6	GALNT <-> 2D3DGALT + H2O	BLASTP
**# Mannose/Mannitol, Fructose and Sorbose/Sorbitol metabolism**				
AO090001000619	D-Sorbitol dehydrogenase (acceptor)	1.1.99.21	SOR + NADH -> SOT + NAD	BLASTP
AO090011000454	Mannose-6-phosphate isomerase	5.3.1.8	MAN6P <-> F6P	BLASTP
AO090012000554	Mannose-6-phosphate isomerase	5.3.1.8	MAN6P <-> F6P	BLASTP
AO090023000719	Mannose-6-phosphate isomerase	5.3.1.8	MAN6P <-> F6P	BLASTP
GAP	Mannitol-1-phosphatase	3.1.3.22	MNT1P + H2O -> MNT + PI	NONE
AO090001000710	Hexokinase	2.7.1.1	ATP + FRU -> ADP + F6P	BLASTP

Gene name	Enzyme name	EC number	Biochemical reaction	Annotation method
AO090003001508	Hexokinase	2.7.1.1	ATP + FRU -> ADP + F6P	BLASTP
AO090005000003	Hexokinase	2.7.1.1	ATP + FRU -> ADP + F6P	BLASTP
AO090005000083	Hexokinase	2.7.1.1	ATP + FRU -> ADP + F6P	BLASTP
AO090012000259	Hexokinase	2.7.1.1	ATP + FRU -> ADP + F6P	BLASTP
AO090011000576	Mannitol-1-phosphate dehydrogenase	1.1.1.17	MNT1P + NAD <-> F6P + NADH	BLASTP
AO090011000230	Mannitol 2-dehydrogenase	1.1.1.67	MNT + NAD <-> FRU + NADH	GFAOP
AO090012000976	6-phosphofructo-2-kinase	2.7.1.105	ATP + F6P -> ADP + F26P	BLASTP
AO090701000027	6-phosphofructo-2-kinase	2.7.1.105	ATP + F6P -> ADP + F26P	BLASTP
AO090005000444	Fructose-2,6-bisphosphatase	3.1.3.46	F26P + H2O -> F6P + PI	BLASTP
AO090003000030	Phosphomannomutase	5.4.2.8	MAN1P <-> MAN6P	BLASTP
AO090003001069	Mannose-1-phosphate guanylyltransferase	2.7.7.13	GTP + MAN1P -> PPI + GDPMAN	BLASTP
AO090003000189	GDP-mannose pyrophosphorylase	2.7.7.22	GDP + MAN1P <-> PI + GDPMAN	BLASTP
AO090011000515	UDP-glucose:glycoprotein glucosyltransferase	2.4.1.-	GDPMAN -> MANNAN	BLASTP
AO090038000631	Sorbitol dehydrogenase	1.1.1.14	SOT + NAD -> FRU + NADH	GFAOP
AO090020000647	Sorbitol dehydrogenase	1.1.1.14	SOT + NAD -> FRU + NADH	GFAOP
AO090701000411	Sorbitol dehydrogenase	1.1.1.14	SOT + NAD -> FRU + NADH	GFAOP
AO090011000515	UDP-glucose:glycoprotein glucosyltransferase	2.4.1.-	GDPMAN -> 14MNAN	BLASTP
AO090038000444	Endo-1,4-beta-mannosidase	3.2.1.78	14MNAN -> MAN	BLASTP
AO090010000122	Endo-1,4-beta-mannosidase	3.2.1.78	14MNAN -> MAN	BLASTP
AO090001000710	Hexokinase	2.7.1.1	ATP + MAN -> ADP + MAN6P	BLASTP
AO090003001508	Hexokinase	2.7.1.1	ATP + MAN -> ADP + MAN6P	BLASTP
AO090005000003	Hexokinase	2.7.1.1	ATP + MAN -> ADP + MAN6P	BLASTP
AO090005000083	Hexokinase	2.7.1.1	ATP + MAN -> ADP + MAN6P	BLASTP
AO090012000259	Hexokinase	2.7.1.1	ATP + MAN -> ADP + MAN6P	BLASTP
**# Disaccharide metabolism (Trehalose, Maltose, Lactose)**				
AO090003000417/ AO090026000820/ AO090020000035/ AO090005001531/ AO090005001530/ AO090102000159/ AO090026000819	Alpha, alpha -trehalose-phosphate synthase (UDP-forming)	2.4.1.15	UDPG + G6P -> UDP + TRE6P	BLASTP

Gene name	Enzyme name	EC number	Biochemical reaction	Annotation method
AO090020000035	Trehalose-phosphatase	3.1.3.12	TRE6P + H2O -> TRE + PI	BLASTP
AO090009000215	Trehalase	3.2.1.28	TRE + H2O -> 2 DGLC	BLASTP
AO090120000052	Trehalase	3.2.1.28	TREe + H2Oe -> 2 DGLCe	BLASTP
AO090103000417	Trehalose synthase	5.4.99.16	TRE <-> MLT	BLASTP
AO090023000288	Alpha-glucosidase	3.2.1.20	MLT + H2O -> 2 GLC	GFAOP
AO090003001209	Alpha-glucosidase	3.2.1.20	MLTe + H2Oe -> 2 GLCe	BLASTP
AO090038000471	Alpha-glucosidase	3.2.1.20	MLTe + H2Oe -> 2 GLCe	BLASTP
AO090005001084	Alpha-glucosidase	3.2.1.20	MLT + H2O -> 2 GLC	GFAOP
AO090103000129	Alpha-glucosidase	3.2.1.20	MLT + H2O -> 2 GLC	GFAOP
AO090038000234	Alpha-glucosidase	3.2.1.20	MLT + H2O -> 2 GLC	BLASTP
AO090012000445	Beta-galactosidase	3.2.1.23	LACTe + H2Oe -> GLCe + GLACe	BLASTP
AO090701000770	Beta-galactosidase	3.2.1.23	LACTe + H2Oe -> GLCe + GLACe	BLASTP
AO090012000745	Beta-galactosidase	3.2.1.23	LACTe + H2Oe -> GLCe + GLACe	BLASTP
AO090003000042	Beta-galactosidase	3.2.1.23	LACTe + H2Oe -> GLCe + GLACe	BLASTP
AO090120000158	Beta-galactosidase	3.2.1.23	LACTe + H2Oe -> GLCe + GLACe	BLASTP
AO090001000259	Beta-galactosidase	3.2.1.23	LACT + H2O -> GLC + GLAC	BLASTP
AO090012000389	Beta-galactosidase	3.2.1.23	LACT + H2O -> GLC + GLAC	BLASTP
**# Polysaccharide metabolism (Starch, Cellulose, Chitin, and Xylan)**				
AO090038000595	UDP-N-acetylglucosamine pyrophosphorylase	2.7.7.23	UTP + NAGA1P <-> PPI + UDPNAG	BLASTP
AO090010000463	UDP-glucose 4-epimerase	5.1.3.7	UDPNAG <-> UDPNAGA	BLASTP
AO090005001490	UDP-glucose 4-epimerase	5.1.3.7	UDPNAG <-> UDPNAGA	BLASTP
AO090009000174/ AO090009000686	1,3-beta-glucan synthase	2.4.1.34	UDPG -> UDP + 13GLUCAN	BLASTP
AO090005001553	Endoglucanase	3.2.1.4	CELLUe -> 13GLUCANe	BLASTP
AO090011000715	Endoglucanase	3.2.1.4	CELLUe -> 13GLUCANe	BLASTP
AO090001000221	Endoglucanase	3.2.1.4	CELLUe -> 13GLUCANe	BLASTP
AO090003000905	Endoglucanase	3.2.1.4	CELLUe -> 13GLUCANe	BLASTP
AO090026000102	Endoglucanase	3.2.1.4	CELLUe -> 13GLUCANe	BLASTP
AO090005001553	Endoglucanase	3.2.1.4	CELLOBe -> 13GLUCANe	BLASTP
AO090011000715	Endoglucanase	3.2.1.4	CELLOBe -> 13GLUCANe	BLASTP

Gene name	Enzyme name	EC number	Biochemical reaction	Annotation method
AO090001000221	Endoglucanase	3.2.1.4	CELLOBe -> 13GLUCANe	BLASTP
AO090003000905	Endoglucanase	3.2.1.4	CELLOBe -> 13GLUCANe	BLASTP
AO090026000102	Endoglucanase	3.2.1.4	CELLOBe -> 13GLUCANe	BLASTP
AO090012000941	Cellulose 1,4-beta-cellobiosidase	3.2.1.91	CELLUe + H2Oe -> CELLOBe + DGLCe + CELLOTe	BLASTP
AO090038000439	Cellulose 1,4-beta-cellobiosidase	3.2.1.91	CELLUe + H2Oe -> CELLOBe + DGLCe + CELLOTe	BLASTP
AO090001000348	Cellulose 1,4-beta-cellobiosidase	3.2.1.91	CELLUe + H2Oe -> CELLOBe + DGLCe + CELLOTe	BLASTP
AO090010000314	Cellulose 1,4-beta-cellobiosidase	3.2.1.91	CELLUe + H2Oe -> CELLOBe + DGLCe + CELLOTe	BLASTP
AO090003000497	Beta-glucosidase	3.2.1.21	CELLOB -> bDGLC	BLASTP
AO090010000034	Beta-glucosidase	3.2.1.21	CELLOB -> bDGLC	BLASTP
AO090011000140	Beta-glucosidase	3.2.1.21	CELLOBe -> bDGLCe	BLASTP
AO090001000544	Beta-glucosidase	3.2.1.21	CELLOB -> bDGLC	BLASTP
AO090009000554	Beta-glucosidase	3.2.1.21	CELLOB -> bDGLC	BLASTP
AO090166000090	Beta-glucosidase	3.2.1.21	CELLOB -> bDGLC	BLASTP
AO090701000244	Beta-glucosidase	3.2.1.21	CELLOB -> bDGLC	BLASTP
AO090701000274	Beta-glucosidase	3.2.1.21	CELLOB -> bDGLC	BLASTP
AO090113000148	Beta-glucosidase	3.2.1.21	CELLOB -> bDGLC	BLASTP
AO090038000223	Beta-glucosidase	3.2.1.21	CELLOB -> bDGLC	BLASTP
AO090038000425	Beta-glucosidase	3.2.1.21	CELLOB -> bDGLC	BLASTP
AO090012000135	Beta-glucosidase	3.2.1.21	CELLOBe -> bDGLCe	BLASTP
AO090009000356	Beta-glucosidase	3.2.1.21	CELLOBe -> bDGLCe	BLASTP
AO090003001511	Beta-glucosidase	3.2.1.21	CELLOBe -> bDGLCe	BLASTP
AO090120000075	Beta-glucosidase	3.2.1.21	CELLOBe -> bDGLCe	BLASTP
AO090701000841	Beta-glucosidase	3.2.1.21	CELLOBe -> bDGLCe	BLASTP
AO090102000032	Cellobiose dehydrogenase	1.1.99.18	CELLOBe <-> CB15LCTe	GFAOP
AO090102000058	Cellobiose dehydrogenase	1.1.99.18	CELLOBe <-> CB15LCTe	GFAOP
AO090010000757	Cellobiose dehydrogenase	1.1.99.18	CELLOB <-> CB15LCT	BLASTP
AO090026000220	Cellobiose dehydrogenase	1.1.99.18	CELLOB <-> CB15LCT	BLASTP
AO090113000054	Cellobiose dehydrogenase	1.1.99.18	CELLOB <-> CB15LCT	BLASTP
AO090010000746	Glucoamylase	3.2.1.3	GLYCOGENe -> GLCe	BLASTP

Gene name	Enzyme name	EC number	Biochemical reaction	Annotation method
AO090003000321	Glucoamylase	3.2.1.3	GLYCOGENe -> GLCe	BLASTP
AO090010000746	Glucoamylase	3.2.1.3	STARe -> GLCe	BLASTP
AO090003000321	Glucoamylase	3.2.1.3	STARe -> GLCe	BLASTP
AO090102000361	Glycogen synthase	2.4.1.11	UDPG -> UDP + GLYNIN	BLASTP
AO090010000483	1,4-alpha-glucan branching enzyme	2.4.1.18	GLYNIN -> GLYCOGEN	BLASTP
AO090012000601	Glycogen phosphorylase	2.4.1.1	GLYCOGEN + PI -> G1P + GLYCODEX	BLASTP
AO090005000884	Amylo-1,6-glucosidase	3.2.1.33	GLYCODEX + H2O -> MLTOSE + DGLYCODEX	BLASTP
AO090012000601	Glycogen phosphorylase	2.4.1.1	MLTOSE + PI -> G1P + MLTIOSE	BLASTP
AO090023000288	Alpha-glucosidase	3.2.1.20	MLTIOSE + H2O -> MLT + GLC	BLASTP
AO090003001209	Alpha-glucosidase	3.2.1.20	MLTIOSE + H2O -> MLT + GLC	BLASTP
AO090038000471	Alpha-glucosidase	3.2.1.20	MLTIOSE + H2O -> MLT + GLC	BLASTP
AO090005001084	Alpha-glucosidase	3.2.1.20	MLTIOSE + H2O -> MLT + GLC	BLASTP
AO090103000129	Alpha-glucosidase	3.2.1.20	MLTIOSE + H2O -> MLT + GLC	BLASTP
AO090038000234	Alpha-glucosidase	3.2.1.20	MLTIOSE + H2O -> MLT + GLC	BLASTP
AO090005000884	4-alpha-glucanotransferase	2.4.1.25	MLT + MLTIOSE -> MLTOSE + bDGLC	BLASTP
AO090102000139	Exo-polygalacturonase	3.2.1.67	PTATEe + H2Oe -> GALUNTe	BLASTP
GAP	D-Galacturonate reductase	1.1.1.19	GALUNT + NADPH <-> LGALNT + NADP	NONE
GAP	L-Galactonate dehydratase	4.2.1.-	LGALNT -> H2O + TR3DHT	NONE
GAP	L-threo-3-deoxy-hexulosonate aldolase	4.1.2.-	TR3DHT <-> PYR + LGLYAL	NONE
GAP	Glyceraldehyde reductase	1.1.1.21	LGLYAL + NADPH -> GL + NADP	NONE
AO090103000019	Beta-N-acetylglucosaminidase	3.2.1.52	CHIB + H2O -> NAG	BLASTP
AO090005000639	Beta-N-acetylglucosaminidase	3.2.1.52	CHIBe + H2Oe -> NAGe	BLASTP
AO090023000367	Chitinase	3.2.1.14	CHIT + H2O -> CHIB	BLASTP
AO090020000207	Chitinase	3.2.1.14	CHIT + H2O -> CHIB	BLASTP
AO090102000586	Chitinase	3.2.1.14	CHIT + H2O -> CHIB	BLASTP
AO090020000231	Chitinase	3.2.1.14	CHIT + H2O -> CHIB	BLASTP
AO090102000563	Chitinase	3.2.1.14	CHIT + H2O -> CHIB	BLASTP
AO090102000591	Chitinase	3.2.1.14	CHIT + H2O -> CHIB	BLASTP
AO090003000464	Chitinase	3.2.1.14	CHIT + H2O -> CHIB	BLASTP
AO090005000815	Chitinase	3.2.1.14	CHIT + H2O -> CHIB	BLASTP
AO090103000218	Chitinase	3.2.1.14	CHIT + H2O -> CHIB	BLASTP

Gene name	Enzyme name	EC number	Biochemical reaction	Annotation method
AO090003000680	Chitinase	3.2.1.14	CHITe + H2Oe -> CHIBe	BLASTP
AO090012000041	Chitinase	3.2.1.14	CHITe + H2Oe -> CHIBe	BLASTP
AO090103000180	Chitinase	3.2.1.14	CHITe + H2Oe -> CHIBe	BLASTP
AO090023000367	Chitinase	3.2.1.14	CHIT + H2O -> NAG	BLASTP
AO090020000207	Chitinase	3.2.1.14	CHIT + H2O -> NAG	BLASTP
AO090102000586	Chitinase	3.2.1.14	CHIT + H2O -> NAG	BLASTP
AO090020000231	Chitinase	3.2.1.14	CHIT + H2O -> NAG	BLASTP
AO090102000563	Chitinase	3.2.1.14	CHIT + H2O -> NAG	BLASTP
AO090102000591	Chitinase	3.2.1.14	CHIT + H2O -> NAG	BLASTP
AO090003000464	Chitinase	3.2.1.14	CHIT + H2O -> NAG	BLASTP
AO090005000815	Chitinase	3.2.1.14	CHIT + H2O -> NAG	BLASTP
AO090103000218	Chitinase	3.2.1.14	CHIT + H2O -> NAG	BLASTP
AO090003000680	Chitinase	3.2.1.14	CHITe + H2Oe -> NAGe	BLASTP
AO090012000041	Chitinase	3.2.1.14	CHITe + H2Oe -> NAGe	BLASTP
AO090103000180	Chitinase	3.2.1.14	CHITe + H2Oe -> NAGe	BLASTP
AO090102000400	N-acetylglucosamine kinase	2.7.1.59	NAG -> NAGA6P	GFAOP
AO090103000023	N-acetylglucosamine-6-phosphate deacetylase	3.5.1.25	NAGA6P+ H2O -> GA6P + AC	BLASTP
AO090103000020	Glucosamine-6-phosphate deaminase	3.5.99.6	GA6P + H2O -> F6P + NH3	BLASTP
AO090001000710	Hexokinase	2.7.1.1	ATP + GLCN -> ADP + GA6P	BLASTP
AO090003001508	Hexokinase	2.7.1.1	ATP + GLCN -> ADP + GA6P	BLASTP
AO090005000003	Hexokinase	2.7.1.1	ATP + GLCN -> ADP + GA6P	BLASTP
AO090005000083	Hexokinase	2.7.1.1	ATP + GLCN -> ADP + GA6P	BLASTP
AO090012000259	Hexokinase	2.7.1.1	ATP + GLCN -> ADP + GA6P	BLASTP
AO090005001370	Chitin synthase	2.4.1.16	UDPNAG -> UDP + CHIT	BLASTP
AO090011000449	Chitin synthase	2.4.1.16	UDPNAG -> UDP + CHIT	BLASTP
AO090012000084	Chitin synthase	2.4.1.16	UDPNAG -> UDP + CHIT	BLASTP
AO090206000079	Chitin synthase	2.4.1.16	UDPNAG -> UDP + CHIT	BLASTP
AO090701000589	Chitin synthase	2.4.1.16	UDPNAG -> UDP + CHIT	BLASTP
AO090005001370	Chitin synthase	2.4.1.16	UDPNAG -> UDP + CHIT	BLASTP

Gene name	Enzyme name	EC number	Biochemical reaction	Annotation method
AO090005001371	Chitin synthase	2.4.1.16	UDPNAG -> UDP + CHIT	BLASTP
AO090113000128	Chitin synthase	2.4.1.16	UDPNAG -> UDP + CHIT	BLASTP
AO090005000579	Chitin synthase	2.4.1.16	UDPNAG -> UDP + CHIT	BLASTP
AO090026000212	Chitin synthase	2.4.1.16	UDPNAG -> UDP + CHIT	BLASTP
AO090026000321	Chitin synthase	2.4.1.16	UDPNAG -> UDP + CHIT	BLASTP
AO090026000323	Chitin synthase	2.4.1.16	UDPNAG -> UDP + CHIT	BLASTP
ZY029184	Chitin synthase	2.4.1.16	UDPNAG -> UDP + CHIT	EST
AO090023000239	Chitin deacetylase	3.5.1.41	CHITe + H2Oe -> CHITOe + ACe	BLASTP
AO090023000238	Chitin deacetylase	3.5.1.41	CHIT + H2O -> CHITO + AC	BLASTP
AO090003001241	Chitin deacetylase	3.5.1.41	CHIT + H2O -> CHITO + AC	BLASTP
AO090011000027	Chitosanase	3.2.1.132	CHITO -> GLCN	BLASTP
AO090020000697	Chitosanase	3.2.1.132	CHITO -> GLCN	BLASTP
AO090113000063	Chitosanase	3.2.1.132	CHITOe -> GLCNe	BLASTP
AO090003000990	Exo-beta-1,3-glucanase	3.2.1.58	13GLUCAN -> GLC	BLASTP
AO090023000365	Exo-beta-1,3-glucanase	3.2.1.58	13GLUCANe -> GLCe	BLASTP
AO090020000144	Exo-beta-1,3-glucanase	3.2.1.58	13GLUCANe -> GLCe	BLASTP
AO090001000604	Exo-beta-1,3-glucanase	3.2.1.58	13GLUCANe -> GLCe	BLASTP
AO090011000362	Exo-beta-1,3-glucanase	3.2.1.58	13GLUCANe -> GLCe	BLASTP
AO090003001500	Alpha-1,3-glucan synthase	2.4.1.183	UDPG -> UDP + 13GLUCAN	BLASTP
AO090026000523	Alpha-1,3-glucan synthase	2.4.1.183	UDPG -> UDP + 13GLUCAN	BLASTP
AO090010000106	Alpha-1,3-glucan synthase	2.4.1.183	UDPG -> UDP + 13GLUCAN	BLASTP
AO090003001498	Alpha-amylase	3.2.1.1	STARe -> GLCe	BLASTP
AO090023000944	Alpha-amylase	3.2.1.1	STARe -> GLCe	BLASTP
AO090120000196	Alpha-amylase	3.2.1.1	STARe -> GLCe	BLASTP
AO090120000263	Alpha-amylase	3.2.1.1	STARe -> GLCe	BLASTP
AO090005001193	Alpha-amylase	3.2.1.1	STAR -> GLC	BLASTP
AO090003001497	Alpha-amylase	3.2.1.1	STAR -> GLC	BLASTP
AO090003001498	Alpha-amylase	3.2.1.1	GLYCOGENe -> GLCe	BLASTP
AO090023000944	Alpha-amylase	3.2.1.1	GLYCOGENe -> GLCe	BLASTP
AO090120000196	Alpha-amylase	3.2.1.1	GLYCOGENe -> GLCe	BLASTP
AO090120000263	Alpha-amylase	3.2.1.1	GLYCOGENe -> GLCe	BLASTP

Gene name	Enzyme name	EC number	Biochemical reaction	Annotation method
AO090005001193	Alpha-amylase	3.2.1.1	GLYCOGEN -> GLC	BLASTP
AO090003001497	Alpha-amylase	3.2.1.1	GLYCOGEN -> GLC	BLASTP
AO090003000476	Mannosyl-oligosaccharide 1,2-alpha-mannosidase	3.2.1.113	MANNAN -> MAN	BLASTP
AO090009000178	Mannosyl-oligosaccharide 1,2-alpha-mannosidase	3.2.1.113	MANNAN -> MAN	BLASTP
AO090003000057	Mannosyl-oligosaccharide 1,2-alpha-mannosidase	3.2.1.113	MANNAN -> MAN	BLASTP
AO090005001458	Alpha-mannosidase	3.2.1.24	MANNAN -> MAN	BLASTP
AO090003001225	Alpha-mannosidase	3.2.1.24	MANNAN -> MAN	BLASTP
AO090001000556	Beta-mannosidase	3.2.1.25	MANNAN -> MAN	BLASTP
AO090005000740	Beta-mannosidase	3.2.1.25	MANNAN -> MAN	BLASTP
AO090010000208	Beta-mannosidase	3.2.1.25	MANNANe -> MANe	BLASTP
AO090023000401	Endo-polygalacturonase	3.2.1.15	PTATEe -> GALUNTe	BLASTP
AO090023000161	Endo-polygalacturonase	3.2.1.15	PTATEe -> GALUNTe	BLASTP
AO090005000186	Endo-polygalacturonase	3.2.1.15	PTATEe -> GALUNTe	BLASTP
AO090023000001	Alpha-L-arabinofuranosidase	3.2.1.55	ARABINe -> LARABe	BLASTP
AO090124000023	Alpha-L-arabinofuranosidase	3.2.1.55	ARABINe -> LARABe	BLASTP
AO090012000298	Alpha-L-arabinofuranosidase	3.2.1.55	ARABINe -> LARABe	BLASTP
AO090020000712	Alpha-L-arabinofuranosidase	3.2.1.55	ARABINe -> LARABe	BLASTP
AO090005000476	Endo-1,5-alpha-L-arabinanase	3.2.1.99	ARABINe -> LARABe	BLASTP
AO090138000055	Endo-1,5-alpha-L-arabinanase	3.2.1.99	ARABINe -> LARABe	BLASTP
AO090701000481	Endo-1,5-alpha-L-arabinanase	3.2.1.99	ARABINe -> LARABe	BLASTP
AO090005000986	Beta-xylosidase	3.2.1.37	XYLANe -> XYLe	GFAOP
AO090103000120	Beta-xylosidase	3.2.1.37	XYLANe -> XYLe	GFAOP
AO090005000337	Beta-xylosidase	3.2.1.37	XYLAN -> XYL	GFAOP
AO090005000698	Beta-xylosidase	3.2.1.37	XYLAN -> XYL	GFAOP
AO090103000326	Endo-1,4-beta-xylanase	3.2.1.8	XYLANe -> XYLe	GFAOP
AO090701000887	Endo-1,4-beta-xylanase	3.2.1.8	XYLANe -> XYLe	GFAOP
AO090001000111	Endo-1,4-beta-xylanase	3.2.1.8	XYLANe -> XYLe	GFAOP
AO090120000026	Endo-1,4-beta-xylanase	3.2.1.8	XYLANe -> XYLe	GFAOP
AO090103000423	Endo-1,4-beta-xylanase	3.2.1.8	XYLANe -> XYLe	GFAOP
AO090001000208	Endo-1,4-beta-xylanase	3.2.1.8	XYLANe -> XYLe	GFAOP

Gene name	Enzyme name	EC number	Biochemical reaction	Annotation method
**# 2. Energy metabolism**				
**# Oxidative phosphorylation**				
ZY104986	Polyphosphate kinase	2.7.4.1	ATP + 3 PI <-> ADP + 2 PPI	EST
AO090005001437	Inorganic pyrophosphatase	3.6.1.1	PPI -> 2 PI	BLASTP
AO090003001115/<br>AO090011000782/<br>AO090001000553/<br>AO090026000229/<br>AO090001000661/<br>AO090011000578/<br>AO090003001313/<br>AO090102000645/<br>AO090012000402/<br>AO090011000502/<br>AO090103000199/<br>AO090672000005/<br>AO090001000459/<br>AO090001000300/<br>AO090003000400/<br>ZY006639/<br>ZY081278/<br>ZY083531/<br>ZY087692	NADH dehydrogenase (ubiquinone)	1.6.5.3	NADHm + Qm + 4 H_PO_m -> NADm + QH2m + 4 H_PO	EST, BLASTP
AO090003001115/<br>AO090011000782/<br>AO090001000553/<br>AO090026000229/<br>AO090001000661/<br>AO090011000578/<br>AO090003001313/<br>AO090102000645/<br>AO090012000402/<br>AO090011000502/	NADH dehydrogenase (ubiquinone)	1.6.5.3	NADH + Qm -> QH2m + NAD	EST, BLASTP

Gene name	Enzyme name	EC number	Biochemical reaction	Annotation method
AO090103000199/ AO090672000005/ AO090001000459/ AO090001000300/ AO090003000400/ ZY006639/ ZY081278/ ZY083531/ ZY087692				
AO090003001115/ AO090011000782/ AO090001000553/ AO090026000229/ AO090001000661/ AO090011000578/ AO090003001313/ AO090102000645/ AO090012000402/ AO090011000502/ AO090103000199/ AO090672000005/ AO090001000459/ AO090001000300/ AO090003000400/ AO090001000657/ AO090011000358/ ZY083011	NADH dehydrogenase (FAD-containing subunit)	1.6.99.3	NADH + FADm -> NAD + FADH2m	EST, BLASTP
AO090023000520	NADPH-cytochrome p-450 reductase	1.6.2.4	NADPH + 2 FERIm -> NADP + 2 FEROm	BLASTP
AO090011000910	NADPH-cytochrome p-450 reductase	1.6.2.4	NADPH + 2 FERIm -> NADP + 2 FEROm	BLASTP
AO090005000842/ AO090020000403/ AO090102000565	H+-exporting ATPase	3.6.3.6	ATPm + H2Om + 3.87 H_PO_m -> ADPm + PIm + 3.87 H_PO	BLASTP

Gene name	Enzyme name	EC number	Biochemical reaction	Annotation method
AO090038000088/ AO090005000141	H+/K+-exchanging ATPase	3.6.3.10	ATPm + H2Om + 3.87 H_PO_m + K -> ADPm + PIm + 3.87 H_PO + Km	BLASTP
AO090011000813/ AO090120000088/ AO090003001200/ AO090701000148	Phospholipid-translocating ATPase	3.6.3.1	ATPm + H2Om + PEm -> ADPm + PIm + PE	BLASTP
AO090011000813/ AO090120000088/ AO090003001200/ AO090701000148	Phospholipid-translocating ATPase	3.6.3.1	ATPm + H2Om + PSm -> ADPm + PIm + PS	BLASTP
AO090011000813/ AO090120000088/ AO090003001200/ AO090701000148	Phospholipid-translocating ATPase	3.6.3.1	ATPm + H2Om + PGm -> ADPm + PIm + PG	BLASTP
AO090011000813/ AO090120000088/ AO090003001200/ AO090701000148	Phospholipid-translocating ATPase	3.6.3.1	ATPm + H2Om + PAm -> ADPm + PIm + PA	BLASTP
AO090009000330/ AO090012000848	Cu2+-exporting ATPase	3.6.3.4	ATPm + H2Om + 3.87 H_PO_m + Cu -> ADPm + PIm + 3.87 H_PO + Cum	BLASTP
AO090023000590/ AO090009000591	Na+-exporting ATPase	3.6.3.7	ATPm + H2Om + 3.87 H_PO_m + Na -> ADPm + PIm + 3.87 H_PO + Nam	BLASTP
AO090001000706/ AO090003000051/ AO090701000406/ AO090038000322/ AO090005000799/ AO090003000614	Ca2+-transporting ATPase	3.6.3.8	ATPm + H2Om + 3.87 H_PO_m + Ca -> ADPm + PIm + 3.87 H_PO + Cam	BLASTP
AO090003000460	NAD(P)H oxidase	1.6.3.1	NADPH + O2 -> NADP + H2O2	BLASTP
AO090003000460	NADPH dehydrogenase (Quinone)	1.6.99.6	NADPH + Qm -> QH2m + NADP	BLASTP
AO090003000545	Electron-transferring-flavoprotein dehydrogenase	1.5.5.1	FADH2m + Qm -> FADm + QH2m	BLASTP

Gene name	Enzyme name	EC number	Biochemical reaction	Annotation method
AO090010000475/<br>AO090102000625/<br>ZY007427	Ubiquinol-cytochrome c reductase	1.10.2.2	QH2m + 2 FERIm + 4 H_PO_m -> Qm + 2 FEROm + 4 H_PO	EST, BLASTP
AO090010000481/<br>AO090026000651/<br>AO090026000421/<br>ZY007107/<br>ZY007619/<br>ZY029203/<br>ZY029342	Cytochrome c oxidase	1.9.3.12	FEROm + 0.5 O2m + 4 H_PO_m -> 2 FERIm + H2Om + 4 H_PO	EST, BLASTP
AO090005000617/<br>AO090005000749/<br>AO090005000604/<br>AO090026000372/<br>AO090010000482/<br>AO090701000168/<br>AO090206000115/<br>AO090010000482/<br>AO090003000607/<br>AO090023000599/<br>ZY036719	F-type ATPase complex	3.6.3.14	ADPm + PIm + 3.87 H_PO -> ATPm + H2Om + 3.87 H_PO_m	EST, BLASTP
AO090102000349/<br>AO090038000334/<br>AO090026000283/<br>AO090120000313/<br>AO090120000091/<br>AO090003001090/<br>AO090012000797/<br>AO090026000517/<br>AO090038000579/<br>AO090038000352/<br>AO090012000274/<br>ZY036719	V-type ATPase complex	3.6.3.14	ADPm + PIm + 3.87 H_PO -> ATPm + H2Om + 3.87 H_PO_m	EST, BLASTP

Gene name	Enzyme name	EC number	Biochemical reaction	Annotation method
AO090009000405	Mitochondrial ADP/ATP carrier proteins	ATP translocase	ADP + PI + ATPm + H2Om + H_PO -> ADPm + PIm + ATP + H2O + H_PO_m	BLASTP
**# Sulfur metabolism**				
AO090038000333	Adenylylsulfate kinase	2.7.1.25	APS + ATP -> ADP + PAPS	BLASTP
AO090020000347	3'-phosphoadenosine-5'-phosphosulfate reductase	1.8.4.8	PAPS + RTHIO -> OTHIO + H2SO3 + PAP	BLASTP
AO090012000271	Sulfite reductase (NADPH)	1.8.1.2	H2SO3 + 3 NADPH -> H2S + 3 NADP + 3 H2O	BLASTP
AO090005001313	Sulfite reductase (NADPH)	1.8.1.2	H2SO3 + 3 NADPH -> H2S + 3 NADP + 3 H2O	BLASTP
AO090001000571	Sulfite reductase (NADPH)	1.8.1.2	H2SO3 + 3 NADPH -> H2S + 3 NADP + 3 H2O	BLASTP
AO090102000277	Sulfite oxidase	1.8.3.1	H2SO3 + O2 + H2O -> SLF + H2O2	BLASTP
AO090020000232	Sulfite oxidase	1.8.3.1	H2SO3 + O2 + H2O -> SLF + H2O2	BLASTP
**# Nitrogen metabolism**				
AO090012000624	Nitrite reductase	1.7.1.4	HNO2 + 3 NADPH -> NH4OH + 3 NADP + H2O	BLASTP
AO090012000626	Nitrate reductase (NADPH)	1.7.1.3	HNO3 + NADPH -> HNO2 + NADP + H2O	BLASTP
AO090003001472	Nitrate reductase (NADH)	1.7.1.1	HNO3 + NADH -> HNO2 + NAD + H2O	BLASTP
AO090003001473	Nitrate reductase (NADH)	1.7.1.1	HNO3 + NADH -> HNO2 + NAD + H2O	BLASTP
AO090005001159	Urea amidolyase	6.3.4.6	ATP + UREA + CO2 + H2O -> ADP + PI + UREAC	BLASTP
AO090010000596	Allophanate hydrolase	3.5.1.54	UREAC -> 2 NH3 + 2 CO2	GFAOP
AO090011000504	Allantoinase	3.5.2.5	ATN <-> ATT	BLASTP
AO090005001321	Allantoicase	3.5.3.4	ATTp <-> UGCp + UREAp	BLASTP
AO090005000694	Ureidoglycolate hydrolase	4.3.2.3	UGCp -> GLXp + UREAp	BLASTP
AO090003000879	Urease	3.5.1.5	UREA + H2O -> CO2 + 2 NH3	GFAOP
AO090020000571	Nitrilase	3.5.5.1	NITE -> CARBO + NH3	BLASTP
AO090003000470	Nitrilase	3.5.5.1	NITE -> CARBO + NH3	BLASTP
AO090020000385	Nitrilase	3.5.5.1	NITE -> CARBO + NH3	BLASTP
AO090010000658	2-nitropropane dioxygenase	1.13.11.32	2 NITROPRO + O2 -> 2 ACET + 2 HNO2	BLASTP
AO090011000650	2-nitropropane dioxygenase	1.13.11.32	2 NITROPRO + O2 -> 2 ACET + 2 HNO2	BLASTP
AO090023000783	2-nitropropane dioxygenase	1.13.11.32	2 NITROPRO + O2 -> 2 ACET + 2 HNO2	BLASTP
AO090103000286	2-nitropropane dioxygenase	1.13.11.32	2 NITROPRO + O2 -> 2 ACET + 2 HNO2	BLASTP
AO090701000520	2-nitropropane dioxygenase	1.13.11.32	2 NITROPRO + O2 -> 2 ACET + 2 HNO2	BLASTP
AO090038000173	2-nitropropane dioxygenase	1.13.11.32	2 NITROPRO + O2 -> 2 ACET + 2 HNO2	BLASTP

Gene name	Enzyme name	EC number	Biochemical reaction	Annotation method
No_Gene	Chemical conversion	No_EC	NH3 + H2O <-> NH4OH	BLASTP
AO090010000582	Carbonic anhydrase	4.2.1.1	CO2 + H2O -> H + HCO3	BLASTP
AO090102000190	Cyanate lyase	4.2.1.104	CYNE + H2O -> CABM	BLASTP
No_Gene	Spontaneous conversion	No_EC	CABM -> CO2 + NH3	BLASTP
AO090010000710	Methylaspartate ammonia-lyase	4.3.1.2	TRMAS -> MESC + NH3	BLASTP
GAP	Methylaspartate mutase	5.4.99.1	GLU -> TRMAS	NONE
GAP	Mesaconate hydratase	4.2.1.34	MESC + H2O -> CML	NONE
GAP	Citramalate lyase	4.1.3.22	CML -> AC + PYR	NONE
**# 3. Amino acid metabolism**				
**# Alanine/aspartate and asparagine metabolism**				
AO090011000679	Aspartate transaminase	2.6.1.1	OAm + GLUm <-> ASPm + AKGm	BLASTP
AO090003001171	Aspartate transaminase	2.6.1.1	OAm + GLUm <-> ASPm + AKGm	BLASTP
AO090120000135	Aspartate transaminase	2.6.1.1	OAm + GLUm <-> ASPm + AKGm	BLASTP
AO090120000135	Aspartate transaminase	2.6.1.1	OA + GLU <-> ASP + AKG	BLASTP
AO090003000931	Aspartate transaminase	2.6.1.1	OA + GLU <-> ASP + AKG	BLASTP
AO090012000899	Aspartate transaminase	2.6.1.1	OA + GLU <-> ASP + AKG	BLASTP
AO090003000164	Alanine aminotransferase	2.6.1.2	PYR + GLU <-> AKG + ALA	BLASTP
AO090003000164	Alanine aminotransferase	2.6.1.2	PYRm + GLUm <-> AKGm + ALAm	BLASTP
AO090020000619	Cysteine desulfurase	2.8.1.7	CYSm + ECYSm -> ALAm + ESULFCYSm	GFAOP
AO090026000714	D-aspartate oxidase	1.4.3.1	DASP + H2O + O2 -> OA + NH3 + H2O2	BLASTP
AO090005000816	Asparaginase	3.5.1.1	ASN -> ASP + NH3	BLASTP
AO090003000216	Asparaginase	3.5.1.1	ASN -> ASP + NH3	BLASTP
AO090023000896	Asparagine synthase (glutamine-hydrolysing)	6.3.5.4	ASP + ATP + GLN -> GLU + ASN + AMP + PPI	BLASTP
AO090103000353	Asparagine synthase (glutamine-hydrolysing)	6.3.5.4	ASP + ATP + GLN -> GLU + ASN + AMP + PPI	BLASTP
AO090120000180	Asparagine synthase (glutamine-hydrolysing)	6.3.5.4	ASP + ATP + GLN -> GLU + ASN + AMP + PPI	BLASTP
AO090009000460	Asparagine synthetase	6.3.1.1	ATP + ASP + NH3 -> AMP + PPI + ASN	BLASTP
AO090020000314	Aspartate-semialdehyde dehydrogenase	1.2.1.11	BASP + NADPH -> NADP + PI + ASPSA	BLASTP
AO090009000702	Aspartokinase	2.7.2.4	ASP + ATP -> ADP + BASP	BLASTP
AO090026000404	Carnitine O-acyltransferase	2.3.1.7	C120COA + CAR <-> C120CAR + COA	BLASTP
AO090001000295	Carnitine O-acyltransferase	2.3.1.7	C120COAm + CARm <-> C120CARm + COAm	BLASTP
AO090026000404	Carnitine O-acyltransferase	2.3.1.7	C120COAm + CARm <-> C120CARm + COAm	BLASTP

Gene name	Enzyme name	EC number	Biochemical reaction	Annotation method
AO090026000404	Carnitine O-acyltransferase	2.3.1.7	C140COA + CAR <-> C140CAR + COA	BLASTP
AO090001000295	Carnitine O-acyltransferase	2.3.1.7	C140COAm + CARm <-> C140CARm + COAm	BLASTP
AO090026000404	Carnitine O-acyltransferase	2.3.1.7	C140COAm + CARm <-> C140CARm + COAm	BLASTP
AO090026000404	Carnitine O-acyltransferase	2.3.1.7	C160COA + CAR <-> C160CAR + COA	BLASTP
AO090001000295	Carnitine O-acyltransferase	2.3.1.7	C160COAm + CARm <-> C160CARm + COAm	BLASTP
AO090026000404	Carnitine O-acyltransferase	2.3.1.7	C160COAm + CARm <-> C160CARm + COAm	BLASTP
AO090026000404	Carnitine O-acyltransferase	2.3.1.7	C180COA + CAR <-> C180CAR + COA	BLASTP
AO090001000295	Carnitine O-acyltransferase	2.3.1.7	C180COAm + CARm <-> C180CARm + COAm	BLASTP
AO090026000404	Carnitine O-acyltransferase	2.3.1.7	C180COAm + CARm <-> C180CARm + COAm	BLASTP
AO090010000640	L-amino-acid oxidase	1.4.3.2	ASP + H2O + O2 -> OA + NH3 + H2O2	BLASTP
AO090010000405	L-amino-acid oxidase	1.4.3.2	ASP + H2O + O2 -> OA + NH3 + H2O2	BLASTP
AO090103000001	Alanyl-tRNA synthetase	6.1.1.7	ATP + ALA + TRNA -> AMP + PPI + LAlaTRNA	BLASTP
AO090005000838	Aspartyl-tRNA synthetase	6.1.1.12	ATP + ASP + TRNA -> AMP + PPI + ASPTRNA	BLASTP
AO090005000838	Aspartyl-tRNA synthetase	6.1.1.12	ATPm + ASPm + TRNAm -> AMPm + PPIm + ASPTRNAm	BLASTP
AO090026000603	Aspartyl-tRNA synthetase	6.1.1.12	ATP + ASP + TRNA -> AMP + PPI + ASPTRNA	BLASTP
AO090023000726	Aspartyl-tRNA synthetase	6.1.1.12	ATP + ASP + TRNA -> AMP + PPI + ASPTRNA	BLASTP
AO090005001286	Asparaginyl-tRNA synthetase	6.1.1.22	ATPm + ASNm + TRNAm -> AMPm + PPIm + ASNTRNAm	BLASTP
AO090001000680	Asparaginyl-tRNA synthetase	6.1.1.22	ATP + ASN + TRNA -> AMP + PPI + ASNTRNA	BLASTP
ZY110993	Alanine racemase	5.1.1.1	DALA <-> ALA	EST
**# Arginine and proline metabolism**				
AO090102000577	S-adenosylmethionine decarboxylase	4.1.1.50	SAM <-> DSAM + CO2	BLASTP
AO090012000528	Spermidine synthase	2.5.1.16	PTRSC + DSAM -> SPRMD + 5MTA	BLASTP
AO090012000528	Spermine synthase	2.5.1.22	DSAM + SPRMD -> 5MTA + SPRM	BLASTP
AO090003001350	Deoxyhypusine synthase	1.5.99.6	SPRMD + Qm -> DAPRP + QH2m	BLASTP
AO090701000729	N-acetylglutamate synthase	2.3.1.1	GLUm + ACCOAm -> COAm + NAGLUm	BLASTP
AO090026000498	Acetylglutamate kinase	2.7.2.8	NAGLUm + ATPm -> ADPm + NAGLUPm	BLASTP
AO090026000149	Acetylglutamate kinase	2.7.2.8	NAGLUm + ATPm -> ADPm + NAGLUPm	BLASTP
ZY111420	Acetylglutamate kinase	2.7.2.8	NAGLUm + ATPm -> ADPm + NAGLUPm	EST
ZY086694	Acetylglutamate kinase	2.7.2.8	NAGLUm + ATPm -> ADPm + NAGLUPm	EST

Gene name	Enzyme name	EC number	Biochemical reaction	Annotation method
Chr 4: SC012 (1076984-1077358)	N-acetyl-γ-glutamyl-phosphate reductase	1.2.1.38	NAGLUPm + NADPHm -> NADPm + PIm + NAGLUSm	GFAOP
AO090026000394	Acetylornithine aminotransferase	2.6.1.11	NAGLUSm + GLUm -> AKGm + NAORNm	BLASTP
AO090005001109	Acetylornithine deacetylase	3.5.1.16	NAORN + H2O -> AC + ORN	GFAOP
AO090005001108	Acetylornithine deacetylase	3.5.1.16	NAORN + H2O -> AC + ORN	GFAOP
AO090003000058	Acetylornithine deacetylase	3.5.1.16	NAORN + H2O -> AC + ORN	GFAOP
AO090023000856	Ornithine carbamoyltransferase	2.1.3.3	ORNm + CAPm -> CITRm + PIm	BLASTP
AO090023000395	Argininosuccinate synthase	6.3.4.5	CITR + ASP + ATP <-> AMP + PPI + ARGSUCC	BLASTP
AO090020000418	Argininosuccinate lyase	4.3.2.1	ARGSUCC <-> FUM + ARG	BLASTP
AO090003000697	Arginase	3.5.3.1	ARG -> ORN + UREA	BLASTP
AO090011000557	Arginase	3.5.3.1	ARG -> ORN + UREA	BLASTP
GAP	Arginine decarboxylase	4.1.1.19	ARG -> AGMT + CO2	NONE
AO090001000694/ AO090120000455/ AO090701000422	Agmatinase	3.5.3.11	AGMT + H2O -> PTRSC + UREA	BLASTP
AO090011000935	Glutamate 5-kinase	2.7.2.11	GLU + ATP -> ADP + GLUP	BLASTP
AO090011000955	Gamma-glutamyl phosphate reductase	1.2.1.41	GLUP + NADPH -> NADP + PI + GLUGSAL	BLASTP
No_Gene	Spontaneous conversion	No_EC	GLUGSALm <-> P5Cm + H2Om	NONE
No_Gene	Spontaneous conversion	No_EC	GLUGSAL <-> P5C + H2O	NONE
AO090001000550	Proline dehydrogenase	1.5.99.8	PROm + FADm -> P5Cm + FADH2m	BLASTP
AO090003000761	Proline hydroxylase	1.14.11.2	PRO + AKG + O2 -> HPRO + SUCC + CO2	BLASTP
AO090001000549	Delta-1-Pyrroline-5-carboxylate dehydrogenase	1.5.1.12	HPROm + NADm -> PHCm + NADHm	BLASTP
AO090206000121	Delta-1-Pyrroline-5-carboxylate dehydrogenase	1.5.1.12	HPROm + NADm -> PHCm + NADHm	BLASTP
AO090005000037	Delta-1-Pyrroline-5-carboxylate dehydrogenase	1.5.1.12	HPROm + NADm -> PHCm + NADHm	BLASTP
AO090012000606	Delta-1-Pyrroline-5-carboxylate dehydrogenase	1.5.1.12	HPROm + NADm -> PHCm + NADHm	BLASTP
AO090001000549	Delta-1-Pyrroline-5-carboxylate dehydrogenase	1.5.1.12	GLUGSALm + NADPm -> NADPHm + GLUm	BLASTP
AO090206000121	Delta-1-Pyrroline-5-carboxylate dehydrogenase	1.5.1.12	GLUGSALm + NADPm -> NADPHm + GLUm	BLASTP
AO090005000037	Delta-1-Pyrroline-5-carboxylate dehydrogenase	1.5.1.12	GLUGSALm + NADPm -> NADPHm + GLUm	BLASTP
AO090012000606	Delta-1-Pyrroline-5-carboxylate dehydrogenase	1.5.1.12	GLUGSALm + NADPm -> NADPHm + GLUm	BLASTP
AO090012000119	Pyrroline-5-carboxylate reductase	1.5.1.2	P5C + NADPH -> PRO + NADP	BLASTP

Gene name	Enzyme name	EC number	Biochemical reaction	Annotation method
AO090120000046	Pyrroline-5-carboxylate reductase	1.5.1.2	P5C + NADPH -> PRO + NADP	BLASTP
AO090005000035	Pyrroline-5-carboxylate reductase	1.5.1.2	P5C + NADPH -> PRO + NADP	BLASTP
AO090206000124	Pyrroline-5-carboxylate reductase	1.5.1.2	P5C + NADPH -> PRO + NADP	BLASTP
AO090012000119	Pyrroline-5-carboxylate reductase	1.5.1.2	PHC + NADPH -> HPRO + NADP	BLASTP
AO090120000046	Pyrroline-5-carboxylate reductase	1.5.1.2	PHC + NADPH -> HPRO + NADP	BLASTP
AO090005000035	Pyrroline-5-carboxylate reductase	1.5.1.2	PHC + NADPH -> HPRO + NADP	BLASTP
AO090206000124	Pyrroline-5-carboxylate reductase	1.5.1.2	PHC + NADPH -> HPRO + NADP	BLASTP
AO090012000119	Pyrroline-5-carboxylate reductase	1.5.1.2	PHC + NADH -> HPRO + NAD	BLASTP
AO090120000046	Pyrroline-5-carboxylate reductase	1.5.1.2	PHC + NADH -> HPRO + NAD	BLASTP
AO090005000035	Pyrroline-5-carboxylate reductase	1.5.1.2	PHC + NADH -> HPRO + NAD	BLASTP
AO090206000124	Pyrroline-5-carboxylate reductase	1.5.1.2	PHC + NADH -> HPRO + NAD	BLASTP
AO090010000012	Proline racemase	5.1.1.4	PRO -> DPRO	BLASTP
AO090010000067	Proline racemase	5.1.1.4	PRO -> DPRO	BLASTP
AO090103000370	Proline racemase	5.1.1.4	PRO -> DPRO	BLASTP
AO090701000729	Glutamate N-acetyltransferase	2.3.1.35	NAORNm + GLUm -> ORNm + NAGLUm	NONE
AO090023000546	Ornithine aminotransferase	2.6.1.13	ORN + AKG -> GLUGSAL + GLU	BLASTP
AO090023000628	Ornithine aminotransferase	2.6.1.13	ORN + AKG -> GLUGSAL + GLU	BLASTP
AO090023000771	Ornithine decarboxylase	4.1.1.17	ORN -> PTRSC + CO2	BLASTP
AO090038000189	Ornithine decarboxylase	4.1.1.17	ORN -> PTRSC + CO2	BLASTP
AO090026000097	Ornithine decarboxylase	4.1.1.17	ORNm -> PTRSCm + CO2m	GFAOP
AO090026000380	Ornithine decarboxylase	4.1.1.17	ORN -> PTRSC + CO2	BLASTP
AO090701000513	Ornithine decarboxylase	4.1.1.17	ORNm -> PTRSCm + CO2m	GFAOP
AO090138000170	Nitric-oxide synthase	1.14.13.39	ARG + NADPH + H + O2 -> CITR + NO + NADP	BLASTP
AO090026000140	Arginyl-tRNA synthetase	6.1.1.19	ATP + ARG + TRNA -> AMP + PPI + ALTRNA	BLASTP
AO090012000200	Prolyl-tRNA synthetase	6.1.1.15	ATP + PRO + TRNA -> AMP + PPI + LPROTRNA	BLASTP
AO090038000342	Prolyl-tRNA synthetase	6.1.1.15	ATP + PRO + TRNA -> AMP + PPI + LPROTRNA	BLASTP

Gene name	Enzyme name	EC number	Biochemical reaction	Annotation method
**# Cysteine metabolism**				
AO090120000210	Homoserine O-acetyltransferase	2.3.1.30	SER + ACCOA -> COA + ASER	BLASTP
AO090701000235	Homoserine O-acetyltransferase	2.3.1.30	SER + ACCOA -> COA + ASER	BLASTP
AO090020000347	3'-phosphoadenosine-5'-phosphosulfate reductase	1.8.4.8	PAPS + RTHIO -> OTHIO + H2SO3 + PAP	BLASTP
AO090005000626	Cysteine synthase	2.5.1.47	ASERm + H2Sm -> ACm + CYSm	BLASTP
AO090102000276	Cysteine synthase	2.5.1.47	ASERm + H2Sm -> ACm + CYSm	BLASTP
AO090011000336	Cysteine synthase	2.5.1.47	ASER + H2S -> AC + CYS	BLASTP
AO090001000584	3'(2',5')-bisphosphate nucleotidase	3.1.3.7	PAP -> AMP + PI	GFAOP
AO090206000077	3'(2',5')-bisphosphate nucleotidase	3.1.3.7	PAP -> AMP + PI	GFAOP
AO090011000904	3'(2',5')-bisphosphate nucleotidase	3.1.3.7	PAP -> AMP + PI	GFAOP
AO090011000009	Cysteine dioxygenase	1.13.11.20	CYS + O2 -> 3SULALA	GFAOP
AO090020000659	Cysteine dioxygenase	1.13.11.20	CYS + O2 -> 3SULALA	GFAOP
AO090009000384	Cysteine dioxygenase	1.13.11.20	CYS + O2 -> 3SULALA	GFAOP
AO090005000162	Sulfhydryl oxidase	1.8.3.2	2 CYS + O2 -> CYST + H2O	BLASTP
AO090005000642	sulfate adenylyltransferase (ADP)	2.7.7.5	ADP + SLF <-> PI + APS	BLASTP
AO090020000349	ATP sulphurylase	2.7.7.4	SLF + ATP -> PPI + APS	BLASTP
AO090009000342	L-serine dehydratase	4.3.1.17	SER -> PYR + NH3	BLASTP
AO090023000790	L-serine dehydratase	4.3.1.17	SER -> PYR + NH3	BLASTP
AO090023000163	Cysteinyl-tRNA synthetase	6.1.1.16	ATP + CYS + TRNA -> AMP + PPI + LCysTRNA	BLASTP
**# Glutamate and glutamine metabolism**				
AO090103000342	Glutamate decarboxylase	4.1.1.15	GLU -> GABA + CO2	BLASTP
AO090005000539	Glutamate decarboxylase	4.1.1.15	GLU -> GABA + CO2	BLASTP
AO090003000666	Glutamate decarboxylase	4.1.1.15	GLU -> GABA + CO2	BLASTP
AO090005000704	Glutamate decarboxylase	4.1.1.15	GLU -> GABA + CO2	BLASTP
AO090009000135	Succinate-semialdehyde dehydrogenase	1.2.1.16	SUCCSAL + NAD + H2O -> SUCC + NADH	BLASTP
AO090023000754	Succinate-semialdehyde dehydrogenase	1.2.1.16	SUCCSAL + NAD + H2O -> SUCC + NADH	BLASTP
AO090005001383	Succinate-semialdehyde dehydrogenase	1.2.1.16	SUCCSAL + NAD + H2O -> SUCC + NADH	BLASTP
AO090020000216	Succinate-semialdehyde dehydrogenase	1.2.1.16	SUCCSAL + NAD + H2O -> SUCC + NADH	BLASTP
AO090009000135	Succinate-semialdehyde dehydrogenase	1.2.1.16	SUCCSAL + NADP -> SUCC + NADPH	BLASTP
AO090023000754	Succinate-semialdehyde dehydrogenase	1.2.1.16	SUCCSAL + NADP -> SUCC + NADPH	BLASTP
AO090005001383	Succinate-semialdehyde dehydrogenase	1.2.1.16	SUCCSAL + NADP -> SUCC + NADPH	BLASTP

Gene name	Enzyme name	EC number	Biochemical reaction	Annotation method
AO090020000216	Succinate-semialdehyde dehydrogenase	1.2.1.16	SUCCSAL + NADP -> SUCC + NADPH	BLASTP
AO090012000988	NADPH-dependent glutamate synthase	1.4.1.13	AKG + GLN + NADPH -> NADP + 2 GLU	BLASTP
AO090001000717	NAD-dependent glutamate dehydrogenase	1.4.1.2	GLU + NAD <-> AKG + NH3 + NADH	BLASTP
AO090001000717	NAD-dependent glutamate dehydrogenase	1.4.1.2	GLUm + NADm <-> AKGm + NH3m + NADHm	BLASTP
AO090023000923	Glutamate dehydrogenase	1.4.1.4	GLU + NADP + H2O <-> AKG + NH3 + NADPH	BLASTP
AO090023000579	Glutamate dehydrogenase	1.4.1.4	GLU + NADP + H2O <-> AKG + NH3 + NADPH	BLASTP
AO090120000132	Glucosamine-phosphate N-acetyltransferase	2.3.1.4	ACCOA + GA6P <-> COA + NAGA6P	GFAOP
AO090023000785	Glucosamine-phosphate N-acetyltransferase	2.3.1.4	ACCOA + GA6P <-> COA + NAGA6P	GFAOP
AO090001000429	N-acetylglucosamine-phosphate mutase	5.4.2.3	NAGA6P <-> NAGA1P	BLASTP
AO090701000206	4-aminobutyrate aminotransferase	2.6.1.19	GABAm + AKGm -> SUCCSALm + GLUm	BLASTP
AO090009000269	Glutamine synthetase	6.3.1.2	GLU + NH3 + ATP -> GLN + ADP + PI	BLASTP
AO090009000558	Glutamine synthetase	6.3.1.2	GLU + NH3 + ATP -> GLN + ADP + PI	BLASTP
AO090026000090	Glutamine synthetase	6.3.1.2	GLU + NH3 + ATP -> GLN + ADP + PI	BLASTP
AO090011000308	Glutamine synthetase	6.3.1.2	GLU + NH3 + ATP -> GLN + ADP + PI	BLASTP
AO090020000289	Glutaminase	3.5.1.2	GLN -> GLU + NH3	BLASTP
AO090003000638	Glutaminase	3.5.1.2	GLN -> GLU + NH3	BLASTP
AO090001000625	Glutaminase	3.5.1.2	GLN -> GLU + NH3	BLASTP
AO090001000626	Glutaminase	3.5.1.2	GLN -> GLU + NH3	BLASTP
AO090003000003	Glucosamine-fructose-6-phosphate aminotransferase	2.6.1.16	GLN + F6P -> GLU + GA6P	BLASTP
AO090003001475	Glucosamine-fructose-6-phosphate aminotransferase	2.6.1.16	GLN + F6P -> GLU + GA6P	BLASTP
AO090102000234	Glutamyl-tRNA synthetase	6.1.1.17	ATPm + GLUm + TRNAm -> AMPm + PPIm + LGLUTRNAm	BLASTP
AO090102000566	Glutamyl-tRNA synthetase	6.1.1.17	ATP + GLU + TRNA -> AMP + PPI + LGLUTRNA	BLASTP
AO090701000597	Glutaminyl-tRNA synthetase	6.1.1.18	ATP + GLN + TRNA -> AMP + PPI + LGLNTRNA	BLASTP
AO090003000698	Glutaminyl-tRNA synthetase	6.1.1.18	ATPm + GLNm + TRNAm -> AMPm + PPIm + LGLNTRNAm	BLASTP

Gene name	Enzyme name	EC number	Biochemical reaction	Annotation method
**# Glycine, serine and threonine metabolism**				
AO090020000670	Phosphoglycerate dehydrogenase	1.1.1.95	3PG + NAD -> NADH + PHP	BLASTP
AO090009000711	Phosphoglycerate dehydrogenase	1.1.1.95	3PG + NAD -> NADH + PHP	BLASTP
AO090020000668	Phosphoglycerate dehydrogenase	1.1.1.95	3PG + NAD -> NADH + PHP	BLASTP
AO090001000641	Phosphoglycerate dehydrogenase	1.1.1.95	3PG + NAD -> NADH + PHP	BLASTP
ZY083916	Phosphoglycerate dehydrogenase	1.1.1.95	3PG + NAD -> NADH + PHP	EST
ZY007726	Phosphoglycerate dehydrogenase	1.1.1.95	3PG + NAD -> NADH + PHP	EST
AO090023000099	Phosphoserine aminotransferase	2.6.1.52	PHP + GLU -> AKG + 3PSER	GFAOP
AO090009000301	Phosphoserine phosphatase	3.1.3.3	3PSER -> PI + SER	BLASTP
AO090020000345	Phosphoserine phosphatase	3.1.3.3	3PSER -> PI + SER	BLASTP
AO090308000017	Phosphoserine phosphatase	3.1.3.3	3PSER -> PI + SER	BLASTP
AO090012000926	Alanine-glyoxylate aminotransferase	2.6.1.44	ALAm + GLXm <-> PYRm + GLYm	BLASTP
AO090308000018	Alanine-glyoxylate aminotransferase	2.6.1.44	ALA + GLX <-> PYR + GLY	GFAOP
AO090020000641	Alanine-glyoxylate aminotransferase	2.6.1.44	ALA + GLX <-> PYR + GLY	GFAOP
AO090003000721	Homoserine dehydrogenase	1.1.1.3	ASPSA + NADPH -> NADP + HSER	BLASTP
AO090009000136	Homoserine dehydrogenase	1.1.1.3	ASPSA + NADPH -> NADP + HSER	BLASTP
AO090003000721	Homoserine dehydrogenase	1.1.1.3	ASPSA + NADH -> NAD + HSER	BLASTP
AO090009000136	Homoserine dehydrogenase	1.1.1.3	ASPSA + NADH -> NAD + HSER	BLASTP
AO090009000682	Homoserine kinase	2.7.1.39	HSER + ATP -> ADP + PHSER	BLASTP
AO090038000224	Threonine synthase	4.2.3.1	PHSER + H2O -> PI + THR	BLASTP
AO090005000497	Threonine aldolase	4.1.2.5	THR -> GLY + ACAL	BLASTP
AO090012000408	Threonine aldolase	4.1.2.5	THR -> GLY + ACAL	BLASTP
AO090011000351	Glycine dehydrogenase	1.4.4.2	GLYm + LIPOm <-> SAPm + CO2m	BLASTP
AO090038000338	Aminomethyltransferase	2.1.2.10	GLYm + THFm + NADm -> METTHFm + NADHm + CO2m + NH3m	BLASTP
AO090038000338	Aminomethyltransferase	2.1.2.10	GLY + THF + NAD -> METTHF + NADH + CO2 + NH3	BLASTP
AO090012000211	Threonine dehydrogenase	1.1.1.103	THR + NAD -> AMOXOBU + NADH	BLASTP
AO090023000523	Threonine dehydrogenase	1.1.1.103	THR + NAD -> AMOXOBU + NADH	BLASTP

Gene name	Enzyme name	EC number	Biochemical reaction	Annotation method
No_Gene	Spontaneous conversion	No_EC	AMOXOBU -> AMAC + CO2	BLASTP
AO090003000775	Amine oxidase	1.4.3.4	AMACm + H2Om + O2m -> MTHGXLm + NH3m + H2O2m	BLASTP
AO090113000186	Amine oxidase	1.4.3.4	AMACm + H2Om + O2m -> MTHGXLm + NH3m + H2O2m	BLASTP
AO090103000118	Amine oxidase	1.4.3.4	AMACm + H2Om + O2m -> MTHGXLm + NH3m + H2O2m	BLASTP
AO090023000744	Amine oxidase	1.4.3.4	AMACm + H2Om + O2m -> MTHGXLm + NH3m + H2O2m	BLASTP
AO090166000103	Amine oxidase	1.4.3.4	AMACm + H2Om + O2m -> MTHGXLm + NH3m + H2O2m	BLASTP
AO090011000247	Amine oxidase	1.4.3.4	AMACm + H2Om + O2m -> MTHGXLm + NH3m + H2O2m	BLASTP
AO090009000288	Amine oxidase	1.4.3.4	AMACm + H2Om + O2m -> MTHGXLm + NH3m + H2O2m	BLASTP
AO090103000401	Copper amine oxidase	1.4.3.6	AMAC + H2O + O2 -> MTHGXL + NH3 + H2O2	BLASTP
AO090003000356	Copper amine oxidase	1.4.3.6	AMAC + H2O + O2 -> MTHGXL + NH3 + H2O2	BLASTP
AO090005000103	Copper amine oxidase	1.4.3.6	AMAC + H2O + O2 -> MTHGXL + NH3 + H2O2	BLASTP
AO090701000307	Copper amine oxidase	1.4.3.6	AMAC + H2O + O2 -> MTHGXL + NH3 + H2O2	BLASTP
AO090138000079	Copper amine oxidase	1.4.3.6	AMAC + H2O + O2 -> MTHGXL + NH3 + H2O2	BLASTP
AO090701000490	Copper amine oxidase	1.4.3.6	AMAC + H2O + O2 -> MTHGXL + NH3 + H2O2	BLASTP
AO090011000229	Copper amine oxidase	1.4.3.6	AMAC + H2O + O2 -> MTHGXL + NH3 + H2O2	BLASTP
AO090120000309	Choline dehydrogenase	1.1.99.1	CHO + FADm -> BETALD + FADH2m	BLASTP
AO090103000021	Betaine aldehyde dehydrogenase	1.2.1.8	BETALD + NAD + H2O -> GLYBET + NADH	BLASTP
AO090701000172	Betaine aldehyde dehydrogenase	1.2.1.8	BETALD + NAD + H2O -> GLYBET + NADH	BLASTP
GAP	Betaine—homocysteine S-methyltransferase	2.1.1.5	GLYBET + HCYS -> DIMEGLY + MET	NONE
AO090023000142	N,N-dimethylglycine oxidase	1.5.99.2	DIMEGLYm + FADm + H2Om -> SARCm + FALDm + FADH2m	BLASTP
AO090038000193/ AO090003000547	Sarcosine oxidase	1.5.3.1	SARC + H2O + O2 -> GLY + FALD + H2O2	BLASTP

Gene name	Enzyme name	EC number	Biochemical reaction	Annotation method
AO090011000931	Cystathionine beta-synthase	4.2.1.22	SER + HCYS -> LLCT	BLASTP
AO090038000174	Threonine dehydratase	4.3.1.19	THR -> OBUT + NH3	GFAOP
AO090023000756	Threonine dehydratase	4.3.1.19	THRm -> OBUTm + NH3m	BLASTP
AO090005000706	Seryl-tRNA synthetase	6.1.1.11	ATP + SER + TRNA -> AMP + PPI + LSERTRNA	BLASTP
AO090009000712	Seryl-tRNA synthetase	6.1.1.11	ATP + SER + TRNA -> AMP + PPI + LSERTRNA	BLASTP
AO090009000672	Glycyl-tRNA synthetase	6.1.1.14	ATP + GLY + TRNA -> AMP + PPI + GLYTRNA	BLASTP
AO090005001088	Threonyl tRNA synthetase	6.1.1.3	ATPm + THRm + TRNAm -> AMPm + PPIm + LTHETRNAm	BLASTP
AO090009000201	Threonyl tRNA synthetase	6.1.1.3	ATP + THR + TRNA -> AMP + PPI + LTHETRNA	BLASTP
ZY140875	Selenocysteine synthase	2.9.1.1	LSERTRNA + SNPI -> LSECTRNA + H2O + PI	EST
**# Histidine metabolism**				
AO090001000740	Glutamine amidotransferase:cyclase	2.4.2.-	PRLP + GLN -> GLU + AICAR + DIMGP	BLASTP
AO090005000719/ AO090120000326/ AO090001000559/ AO090005000352	HNRNP arginine N-methyltransferase	2.1.1.-	SAM + HIS -> SAH + MHIS	BLASTP
AO090005000694	Ureidoglycolate hydrolase	3.5.3.19	UGC <-> GLX + 2 NH3 + CO2	BLASTP
AO090009000641	5-proFAR isomerase	5.3.1.16	PRFP -> PRLP	BLASTP
AO090005000146	Histidinol dehydrogenase	3.6.1.31	PRBATP -> PPI + PRBAMP	BLASTP
AO090005000146	Histidinol dehydrogenase	3.5.4.19	PRBAMP -> PRFP	BLASTP
AO090701000070	Imidazoleglycerol-phosphate dehydratase	4.2.1.19	DIMGP -> IMACP	BLASTP
AO090012000450	Histidinol-phosphate aminotransferase	2.6.1.9	IMACP + GLU -> AKG + HISOLP	BLASTP
AO090206000105	Histidinol-phosphatase	3.1.3.15	HISOLP -> PI + HISOL	BLASTP
AO090010000720	Histidinol-phosphatase	3.1.3.15	HISOLP -> PI + HISOL	BLASTP
AO090005000146	Histidinol dehydrogenase	1.1.1.23	HISOL + 2 NAD -> HIS + 2 NADH	BLASTP
AO090005000135	ATP phosphoribosyltransferase	2.4.2.17	PRPP + ATP -> PPI + PRBATP	BLASTP
GAP	Histidine ammonia-lyase	4.3.1.3	HIS -> NH3 + UROCA	NONE
GAP	Urocanate hydratase	4.2.1.49	UROCA + H2O -> IMIPRO	NONE
AO090001000184	Imidazolonepropionase	3.5.2.7	IMIPRO + H2O -> FORGLU	GFAOP
AO090003000932	Imidazolonepropionase	3.5.2.7	IMIPRO + H2O -> FORGLU	GFAOP
GAP	Formimidoylglutamase	3.5.3.8	FORGLU + H2O -> GLU + FORMIE	NONE

Gene name	Enzyme name	EC number	Biochemical reaction	Annotation method
AO090009000295	Histidyl-tRNA synthetase	6.1.1.21	ATP + HIS + TRNA -> AMP + PPI + HHTRNA	BLASTP
**# Valine, leucine and isoleucine metabolism (Branched-chain amino acids)**				
AO090023000977/ AO090011000598/ AO090005000936/ AO090011000044/ AO090011000598/ AO090023000123	Branched chain amino acid aminotransferase	2.6.1.42	AKGm + ILEm <-> OMVALm + GLUm	BLASTP
AO090023000977/ AO090011000598/ AO090005000936/ AO090011000044/ AO090011000598/ AO090023000123	Branched chain amino acid aminotransferase	2.6.1.42	AKGm + VALm <-> OIVALm + GLUm	BLASTP
AO090023000977/ AO090011000598/ AO090005000936/ AO090011000044/ AO090011000598/ AO090023000123	Branched chain amino acid aminotransferase	2.6.1.42	AKGm + LEUm <-> OICAPm + GLUm	BLASTP
AO090023000854/ AO090003000571/ AO090166000076/ ZY098280	Acetolactate synthase	2.2.1.6	OBUTm + PYRm -> ABUTm + CO2m	EST, GFAOP
AO090023000854/ AO090003000571/ AO090166000076/ ZY098280	Acetolactate synthase	2.2.1.6	2 PYRm -> CO2m + ACLACm	EST, GFAOP

Gene name	Enzyme name	EC number	Biochemical reaction	Annotation method
AO090701000557	Ketol-acid reductoisomerase	1.1.1.86	ACLACm + NADPHm -> NADPm + DHVALm	BLASTP
AO090701000557	Ketol-acid reductoisomerase	1.1.1.86	ABUTm + NADPHm -> NADPm + DHMVAm	BLASTP
AO090010000053	Dihydroxy-acid dehydratase	4.2.1.9	DHVALm -> OIVALm	GFAOP
AO090102000231	Dihydroxy-acid dehydratase	4.2.1.9	DHVALm -> OIVALm	GFAOP
AO090009000414	Dihydroxy-acid dehydratase	4.2.1.9	DHVALm -> OIVALm	GFAOP
AO090012000890	Dihydroxy-acid dehydratase	4.2.1.9	DHVALm -> OIVALm	GFAOP
AO090023000127	Dihydroxy-acid dehydratase	4.2.1.9	DHVALm -> OIVALm	GFAOP
AO090010000053	Dihydroxy-acid dehydratase	4.2.1.9	DHMVAm -> OMVALm	GFAOP
AO090102000231	Dihydroxy-acid dehydratase	4.2.1.9	DHMVAm -> OMVALm	GFAOP
AO090009000414	Dihydroxy-acid dehydratase	4.2.1.9	DHMVAm -> OMVALm	GFAOP
AO090012000890	Dihydroxy-acid dehydratase	4.2.1.9	DHMVAm -> OMVALm	GFAOP
AO090023000127	Dihydroxy-acid dehydratase	4.2.1.9	DHMVAm -> OMVALm	GFAOP
AO090005001230	2-isopropylmalate synthase	2.3.3.13	ACCOAm + OIVALm -> COAm + IPPMALm	BLASTP
AO090026000524	3-isopropylmalate dehydratase	4.2.1.33	CBHCAP <-> IPPMAL	BLASTP
AO090026000524	3-isopropylmalate dehydratase	4.2.1.33	PPMAL <-> IPPMAL	BLASTP
AO090005001122	3-isopropylmalate dehydrogenase	1.1.1.85	IPPMALm + NADm -> NADHm + OICAPm + CO2m	BLASTP
AO090103000328	3-isopropylmalate dehydrogenase	1.1.1.85	IPPMALm + NADm -> NADHm + OICAPm + CO2m	BLASTP
AO090001000555	2-oxoisovalerate dehydrogenase complex	1.2.4.4/1.8.1.4/ 2.3.1.168	OIVAL + NAD + COA ->ISOBUCOA + CO2 + NADH	BLASTP
AO090001000555	2-oxoisovalerate dehydrogenase complex	1.2.4.4/1.8.1.4/ 2.3.1.168	OMVAL + NAD + COA -> METBUCOA + CO2 + NADH	BLASTP
AO090001000555	2-oxoisovalerate dehydrogenase complex	1.2.4.4/1.8.1.4/ 2.3.1.168	OICAP + NAD + COA -> ISOVACOA + CO2 + NADH	BLASTP
AO090020000493	Isovaleryl-CoA dehydrogenase	1.3.99.10	ISOVACOAm + FADm -> MCRCOAm + FADH2m	BLASTP
AO090020000495/ AO090020000492	Methylcrotonoyl-CoA carboxylase	6.4.1.4	ATPm + MCRCOAm + HCO3m -> ADPm + PIm + MGCOAm	BLASTP

Gene name	Enzyme name	EC number	Biochemical reaction	Annotation method
AO090003000703	Methylglutaconyl-CoA hydratase	4.2.1.18	H2Om + MGCOAm <-> H3MCOAm	BLASTP
AO090026000392	3-hydroxymethyl-3-methylglutaryl-Coenzyme A lyase	4.1.3.4	H3MCOAm + COAm -> ACTACm + ACCOAm	GFAOP
AO090038000541	3-hydroxymethyl-3-methylglutaryl-Coenzyme A lyase	4.1.3.4	H3MCOAm + COAm -> ACTACm + ACCOAm	GFAOP
AO090005001248	Acyl-CoA dehydrogenase	1.3.99.2	ISOBUCOAm + FADm -> MCRCOAm + FADH2m	BLASTP
AO090011000575	Enoyl-CoA hydratase	4.2.1.17	MCRCOAm + H2Om -> HYISOCOAm	BLASTP
AO090120000413	3-hydroxyisobutyryl-CoA hydrolase	3.1.2.4	HYISOCOAm + H2Om -> HYISORATEm + COAm	BLASTP
AO090023000518	3-hydroxyisobutyrate dehydrogenase	1.1.1.31	HYISORATEm + NADm <-> MMSHYm + NADHm	BLASTP
AO090009000314	Methylmalonate-semialdehyde dehydrogenase	1.2.1.27	MMSHYm + COAm + NADm -> PROPCOAm + CO2m + NADHm	BLASTP
AO090005001248	Acyl-CoA dehydrogenase	1.3.99.2	METBUCOA + FADm -> TGLCOA + FADH2m	BLASTP
AO090011000575	Enoyl-CoA hydratase	4.2.1.17	TGLCOA + H2O -> METBYCOA	GFAOP
AO090001000629/ AO090026000443/ AO090009000113/ AO090026000411/ AO090103000436	3-hydroxyacyl-CoA dehydrogenase	1.1.1.35	METBYCOA + NAD <-> MCECOA + NADH	BLASTP
AO090005000090	3-ketoacyl-CoA thiolase	2.3.1.16	MCECOA + COA -> ACCOA + PROPCOA	BLASTP
AO090003001121	3-ketoacyl-CoA thiolase	2.3.1.16	MCECOA + COA -> ACCOA + PROPCOA	BLASTP
AO090026000515	3-ketoacyl-CoA thiolase	2.3.1.16	MCECOA + COA -> ACCOA + PROPCOA	BLASTP
AO090012000715	3-ketoacyl-CoA thiolase	2.3.1.16	MCECOA + COA -> ACCOA + PROPCOA	BLASTP
AO090012000505	Isoleucyl-tRNA synthetase	6.1.1.5	ATP + ILE + TRNA -> AMP + PPI + LILEUTRNA	BLASTP
AO090010000218	Isoleucyl-tRNA synthetase	6.1.1.5	ATPm + ILEm + TRNAm -> AMPm + PPIm + LILEUTRNAm	BLASTP
AO090005000667	Valyl-tRNA synthetase	6.1.1.9	ATP + VAL + TRNA -> AMP + PPI + LValTRNA	BLASTP
AO090003000376	Leucyl-tRNA synthetase	6.1.1.4	ATP + LEU + TRNA -> AMP + PPI + LLeuTRNA	BLASTP
AO090701000047	Leucyl-tRNA synthetase	6.1.1.4	ATP + LEU + TRNA -> AMP + PPI + LLeuTRNA	BLASTP

Gene name	Enzyme name	EC number	Biochemical reaction	Annotation method
**# Lysine metabolism**				
AO090003001165	Homocitrate synthase	2.3.3.14	ACCOA + H2O + AKG -> HCIT + COA	BLASTP
AO090124000027	Homocitrate synthase	2.3.3.14	ACCOA + H2O + AKG -> HCIT + COA	BLASTP
AO090701000175	Methylcitrate dehydratase	4.2.1.79	HCIT <-> HACN	BLASTP
AO090701000175	Methylcitrate dehydratase	4.2.1.79	HCITm <-> HACNm	BLASTP
AO090010000161	Methylcitrate dehydratase	4.2.1.79	HCIT <-> HACN	BLASTP
AO090701000041	Homoaconitase	4.2.1.36	HACNm <-> HICITm	BLASTP
AO090003001097	Alpha-aminoadipate reductase	1.2.1.31	AMA + NADPH + ATP -> AMASA + NADP + AMP + PPI	BLASTP
AO090003001086	Saccharopine dehydrogenase (NADP, L-glutamate-forming)	1.5.1.10	GLU + AMASA + NADPH <-> SACP + NADP	BLASTP
AO090005001561	Homoisocitrate dehydrogenase	1.1.1.87	HICIT + NAD <-> AKA + CO2 + NADH	BLASTP
AO090026000563	2-aminoadipate transaminase	2.6.1.39	AKA + GLU <-> AMA + AKG	GFAOP
AO090011000361	2-aminoadipate transaminase	2.6.1.39	AKA + GLU <-> AMA + AKG	GFAOP
AO090003000728	Saccharopine dehydrogenase (NAD+, L-lysine forming)	1.5.1.7	SACP + NAD <-> LYS + AKG + NADH	GFAOP
AO090003000729	Saccharopine dehydrogenase (NAD+, L-lysine forming)	1.5.1.7	SACP + NAD <-> LYS + AKG + NADH	GFAOP
AO090001000233	Saccharopine dehydrogenase (NAD+, L-lysine forming)	1.5.1.7	SACP + NAD <-> LYS + AKG + NADH	GFAOP
AO090003001442	Dihydrodipicolinate synthase	4.2.1.52	ASPSA + PYR -> DIDIPC + 2 H2O	BLASTP
AO090003000186	Lysyl-tRNA synthetase	6.1.1.6	ATP + LYS + TRNA -> AMP + PPI + LLTRNA	BLASTP
AO090003000867	Lysyl-tRNA synthetase	6.1.1.6	ATP + LYS + TRNA -> AMP + PPI + LLTRNA	BLASTP
**# Methionine metabolism**				
AO090020000019	Cystathionine gamma-synthase	2.5.1.48	PHSER + CYS -> LLCT + SUCC	BLASTP
AO090003001113	Cystathionine gamma-synthase	2.5.1.48	PHSER + CYS -> LLCT + SUCC	BLASTP
AO090038000528	Cystathionine beta-lyase	4.4.1.8	LLCT + H2O -> HCYS + PYR + NH3	BLASTP
AO090012000886	Cystathionine beta-lyase	4.4.1.8	LLCT + H2O -> HCYS + PYR + NH3	BLASTP
AO090023000150	Cystathionine beta-lyase	4.4.1.8	LLCT + H2O -> HCYS + PYR + NH3	BLASTP
AO090120000144	Cystathionine beta-lyase	4.4.1.8	LLCT + H2O -> HCYS + PYR + NH3	BLASTP

Gene name	Enzyme name	EC number	Biochemical reaction	Annotation method
AO090023000837	5-methyltetrahydropteroyltriglutamate—homocysteine S-methyltransferase	2.1.1.14	HCYS + MTHPTGLU -> THPTGLU + MET	BLASTP
AO090003000768	5-methyltetrahydropteroyltriglutamate—homocysteine S-methyltransferase	2.1.1.14	HCYS + MTHPTGLU -> THPTGLU + MET	BLASTP
AO090003000768	Methionine synthase	2.1.1.13	HCYS + MTHF -> THF + MET	BLASTP
AO090023000837	Methionine synthase	2.1.1.13	HCYS + MTHF -> THF + MET	BLASTP
AO090038000357	S-adenosylmethionine synthetase	2.5.1.6	MET + ATP -> PPI + PI + SAM	BLASTP
AO090701000299	S-adenosylmethionine synthetase	2.5.1.6	MET + ATP -> PPI + PI + SAM	BLASTP
AO090038000417	Adenosylhomocysteinase	3.3.1.1	SAH -> HCYS + ADN	BLASTP
AO090005001181	Homoserine O-acetyltransferase	2.3.1.31	ACCOA + HSER <-> COA + OAHSER	BLASTP
AO090120000210	Homoserine O-acetyltransferase	2.3.1.31	ACCOA + HSER <-> COA + OAHSER	BLASTP
AO090701000235	Homoserine O-acetyltransferase	2.3.1.31	ACCOA + HSER <-> COA + OAHSER	BLASTP
AO090005000626	Cysteine synthase	2.5.1.47	ASERm + H2Sm -> ACm + CYSm	BLASTP
AO090102000276	Cysteine synthase	2.5.1.47	ASERm + H2Sm -> ACm + CYSm	BLASTP
AO090011000336	Cysteine synthase	2.5.1.47	ASER + H2S -> AC + CYS	BLASTP
AO090102000630	O-acetylhomoserine (thiol)-lyase	2.5.1.49	OAHSER + H2S -> HCYS + AC	BLASTP
AO090020000019	Cystathionine gamma-synthase	2.5.1.48	CYS + OSLHSER -> SUCC + LLCT	BLASTP
AO090003001113	Cystathionine gamma-synthase	2.5.1.48	CYS + OSLHSER -> SUCC + LLCT	BLASTP
AO090012000475	Diphthine synthase	2.1.1.98	SAM + CALH -> SAH + DPTH	BLASTP
AO090103000051	Cystathionine gamma-lyase	4.4.1.1	LLCT -> CYS + NH3 + OBUT	BLASTP
AO090011000323	Cystathionine gamma-lyase	4.4.1.1	LLCT -> CYS + NH3 + OBUT	BLASTP
AO090010000103	Homocysteine S-methyltransferase	2.1.1.10	SAM + HCYS -> SAH + MET	GFAOP
AO090001000546	S-methyl-5'-thioadenosine phosphorylase	2.4.2.28	5MTA + PI -> 5THR1P + AD	BLASTP
AO090023000789	Methionyl-tRNA synthetase	6.1.1.10	ATP + MET + TRNA -> AMP + PPI + LMETTRNA	BLASTP
**# Phenylalanine, tyrosine and tryptophan biosynthesis (Aromatic amino acids)**				
AO090005000886	Phospho-2-dehydro-3-deoxyheptonate aldolase	2.5.1.54	E4P + PEP -> PI + 3DDAH7P	BLASTP
AO090005000086	Phospho-2-dehydro-3-deoxyheptonate aldolase	2.5.1.54	E4P + PEP -> PI + 3DDAH7P	BLASTP
AO090001000214	Phospho-2-dehydro-3-deoxyheptonate aldolase	2.5.1.54	E4P + PEP -> PI + 3DDAH7P	BLASTP
AO090023000682	Phospho-2-dehydro-3-deoxyheptonate aldolase	2.5.1.54	E4P + PEP -> PI + 3DDAH7P	BLASTP

Gene name	Enzyme name	EC number	Biochemical reaction	Annotation method
AO090012000502	3-dehydroquinate synthase	4.2.3.4	3DDAH7P -> DQT + PI	BLASTP
AO090038000261	3-dehydroquinate dehydratase	4.2.1.10	DQT -> DHSK	BLASTP
AO090012000502	3-dehydroquinate dehydratase	4.2.1.10	DQT -> DHSK	BLASTP
AO090012000502	Shikimate dehydrogenase	1.1.1.25	DHSK + NADPH -> SME + NADP	BLASTP
AO090038000263	Quinate dehydrogenase	1.1.1.24	QT + NAD <-> DQT + NADH	BLASTP
AO090012000502	Shikimate kinase	2.7.1.71	SME + ATP -> ADP + SME3P	BLASTP
AO090012000502	3-phosphoshikimate 1-carboxyvinyltransferase	2.5.1.19	SME3P + PEP -> 3PSME + PI	BLASTP
AO090003000081	Chorismate synthase	4.2.3.5	3PSME -> PI + CHOR	BLASTP
AO090003000371/ AO090012000581/ AO090026000117	Anthranilate synthase	4.1.3.27	CHOR + GLN -> GLU + PYR + AN	GFAOP
AO090003001011	Anthranilate phosphoribosyltransferase	2.4.2.18	AN + PRPP -> PPI + NPRAN	BLASTP
AO090012000581	Phosphoribosylanthranilate isomerase	5.3.1.24	NPRAN -> CPAD5P	GFAOP
AO090012000581	Indoleglycerol phosphate synthase	4.1.1.48	CPAD5P -> CO2 + IGP	GFAOP
AO090005001315/ AO090023000874/ AO090026000284	Tryptophan synthetase	4.2.1.20	IGP + SER -> T3P1 + TRP	BLASTP
AO090020000618	Tryptophan 2,3-dioxygenase	1.13.11.11	TRP + O2 -> FKYN	BLASTP
AO090003001248	Tryptophan 2,3-dioxygenase	1.13.11.11	TRP + O2 -> FKYN	BLASTP
AO090038000057	Aromatic-L-amino-acid decarboxylase	4.1.1.28	TRP -> TRPM + CO2	GFAOP
AO090038000010	Aromatic-L-amino-acid decarboxylase	4.1.1.28	TRP -> TRPM + CO2	GFAOP
ZY093903	Aromatic-L-amino-acid decarboxylase	4.1.1.28	TRP -> TRPM + CO2	EST
ZY087560	Aromatic-L-amino-acid decarboxylase	4.1.1.28	TRP -> TRPM + CO2	EST
AO090011000472	Formamidase	3.5.1.9	FKYN -> FOR + KYN	BLASTP
AO090011000602	Kynureninase	3.7.1.3	KYN -> ALA + AN	BLASTP
AO090003001247	Kynureninase	3.7.1.3	KYN -> ALA + AN	BLASTP
AO090005001567	Kynurenine 3-monooxygenase	1.14.13.9	KYNm + NADPHm + O2m -> HKYNm + NADPm	BLASTP
AO090003001247	Kynureninase	3.7.1.3	HKYN -> HAN + ALA	BLASTP
AO090011000602	Kynureninase	3.7.1.3	HKYN -> HAN + ALA	BLASTP
AO090023000314	3-Hydroxyanthranilate 3,4-dioxygenase	1.13.11.6	HAN + O2 -> CMUSA	BLASTP

Gene name	Enzyme name	EC number	Biochemical reaction	Annotation method
AO090120000370	2-amino-3-carboxymuconate-6-semialdehyde decarboxylase	4.1.1.45	CMUSA -> CO2 + AM6SA	BLASTP
AO090005000682	2-amino-3-carboxymuconate-6-semialdehyde decarboxylase	4.1.1.45	CMUSA -> CO2 + AM6SA	BLASTP
AO090102000067	2-amino-3-carboxymuconate-6-semialdehyde decarboxylase	4.1.1.45	CMUSA -> CO2 + AM6SA	BLASTP
GAP	2-Aminomuconate-reductase	1.5.1.-	AMUCO + NADPH -> AKA + NADP + NH3	NONE
AO090120000438	Chorismate mutase	5.4.99.5	CHOR -> PHEN	BLASTP
AO090011000595	Prephenate dehydrogenase	1.3.1.12	PHEN + NAD -> 4HPP + CO2 + NADH	BLASTP
AO090011000595	Prephenate dehydrogenase	1.3.1.13	PHEN + NADP -> 4HPP + CO2 + NADPH	BLASTP
AO090009000693	Prephenate dehydratase	4.2.1.51	PHEN -> CO2 + PHPYR	BLASTP
AO090026000245	Aromatic-amino-acid transaminase	2.6.1.57	PHPYR + GLU <-> AKG + PHE	BLASTP
AO090020000444	Aromatic-amino-acid transaminase	2.6.1.57	PHPYR + GLU <-> AKG + PHE	BLASTP
AO090102000498	Aromatic-amino-acid transaminase	2.6.1.57	PHPYR + GLU <-> AKG + PHE	BLASTP
AO090120000135	Aspartate transaminase	2.6.1.1	4HPP + GLU <-> AKG + TYR	BLASTP
GAP	Aminomuconate-semialdehyde dehydrogenase	1.2.1.32	AM6SA + NAD -> AMUCO + NADH	NONE
AO090003000208	4-hydroxyphenylpyruvate dioxygenase	1.13.11.27	4HPP + O2 -> HOMOGEN + CO2	BLASTP
AO090038000266	4-hydroxyphenylpyruvate dioxygenase	1.13.11.27	4HPP + O2 -> HOMOGEN + CO2	BLASTP
AO090103000429	4-hydroxyphenylpyruvate dioxygenase	1.13.11.27	4HPP + O2 -> HOMOGEN + CO2	BLASTP
AO090001000548	4-hydroxyphenylpyruvate dioxygenase	1.13.11.27	4HPP + O2 -> HOMOGEN + CO2	BLASTP
AO090103000492	4-hydroxyphenylpyruvate dioxygenase	1.13.11.27	4HPP + O2 -> HOMOGEN + CO2	BLASTP
AO090003000210	Homogentisate 1,2-dioxygenase	1.13.11.5	HOMOGEN + O2 -> MACAC	BLASTP
AO090012000156	Homogentisate 1,2-dioxygenase	1.13.11.5	HOMOGEN + O2 -> MACAC	BLASTP
AO090003000212	Maleylacetoacetate isomerase	5.2.1.2	MACAC -> FUACAC	BLASTP
AO090003000211	Fumarylacetoacetate hydrolase	3.7.1.2	FUACAC -> FUM + ACTAC	BLASTP
AO090701000601	Phenylalanine ammonia-lyase	4.3.1.5	PHE -> CINNAM + NH3	GFAOP
AO090011000788	Phenylalanine ammonia-lyase	4.3.1.5	PHE -> CINNAM + NH3	GFAOP
AO090026000586	Phenylalanine ammonia-lyase	4.3.1.5	PHE -> CINNAM + NH3	GFAOP
AO090005000532	Phenylalanine ammonia-lyase	4.3.1.5	PHE -> CINNAM + NH3	GFAOP
AO090010000722	Peroxidase	1.11.1.7	PHEm + H2O2m -> PADm + 2 H2Om	BLASTP

Gene name	Enzyme name	EC number	Biochemical reaction	Annotation method
AO090038000171	Acetamidase	3.5.1.4	PAD -> PAC + NH3	BLASTP
AO090003000129	Acetamidase	3.5.1.4	PAD -> PAC + NH3	BLASTP
AO090701000035	Acetamidase	3.5.1.4	PAD -> PAC + NH3	BLASTP
AO090038000537	Acetamidase	3.5.1.4	PAD -> PAC + NH3	BLASTP
AO090038000171	Acetamidase	3.5.1.4	IAD -> IAC + NH3	BLASTP
AO090003000129	Acetamidase	3.5.1.4	IAD -> IAC + NH3	BLASTP
AO090701000035	Acetamidase	3.5.1.4	IAD -> IAC + NH3	BLASTP
AO090038000537	Acetamidase	3.5.1.4	IAD -> IAC + NH3	BLASTP
AO090012000770	Diamine transaminase	2.6.1.29	SPRMD + ACCOA -> ASPERMD + COA	BLASTP
AO090009000545	Polyamine oxidase	1.5.3.11	ASPERMD + O2 -> APRUT + APROA + H2O2	BLASTP
AO090010000340	Polyamine oxidase	1.5.3.11	ASPERMD + O2 -> APRUT + APROA + H2O2	BLASTP
AO090009000545	Polyamine oxidase	1.5.3.11	APRUT + O2 -> GABAL + APROA + H2O2	BLASTP
AO090010000340	Polyamine oxidase	1.5.3.11	APRUT + O2 -> GABAL + APROA + H2O2	BLASTP
AO090003001103	Kynurenine aminotransferase	2.6.1.7	KYN + AKG -> APEBU + GLU	BLASTP
AO090026000525	Aryl-alcohol dehydrogenase	1.1.1.90	PHACAL + NADH <-> PHEETHAL + NAD	BLASTP
AO090113000193	Aryl-alcohol dehydrogenase	1.1.1.90	PHACALm + NADHm <-> PHEETHALm + NADm	BLASTP
AO090138000198	Aryl-alcohol dehydrogenase	1.1.1.90	PHACALm + NADHm <-> PHEETHALm + NADm	BLASTP
AO090011000472	Formamidase	3.5.1.49	FORMIE + H2O -> FOR + NH3	BLASTP
AO090003000380	Catalase	1.11.1.6	2 H2O2p -> 2 H2Op + O2p	GFAOP
AO090011000540	Catalase	1.11.1.6	2 H2O2p -> 2 H2Op + O2p	GFAOP
AO090020000389	Catalase	1.11.1.6	2 H2O2p -> 2 H2Op + O2p	GFAOP
AO090113000153	Catalase	1.11.1.6	2 H2O2p -> 2 H2Op + O2p	GFAOP
AO090113000153	Catalase	1.11.1.6	2 H2O2m -> 2 H2Om + O2m	BLASTP
AO090010000722	Catalase	1.11.1.6	2 H2O2m -> 2 H2Om + O2m	BLASTP
AO090120000068	Catalase	1.11.1.6	2 H2O2 -> 2 H2O + O2	BLASTP
AO090701000158	Catalase	1.11.1.6	2 H2O2 -> 2 H2O + O2	BLASTP
AO090120000068	Catalase	1.11.1.6	2 H2O2e -> 2 H2Oe + O2e	BLASTP
AO090701000158	Catalase	1.11.1.6	2 H2O2e -> 2 H2Oe + O2e	BLASTP
AO090003001055	2-oxoglutarate dehydrogenase	1.2.4.2	AKA -> GLTCOA + CO2	BLASTP
AO090020000074	Glutaryl-CoA dehydrogenase	1.3.99.7	GLTCOAm + FADm -> CRONYLCOAm + CO2m + FADH2m	BLASTP

Gene name	Enzyme name	EC number	Biochemical reaction	Annotation method
AO090124000024	Tyrosyl-tRNA synthetase	6.1.1.1	ATP + TYR + TRNA -> AMP + PPI+ LTyrTRNA	BLASTP
AO090023000727	Tyrosyl-tRNA synthetase	6.1.1.1	ATPm + TYRm + TRNAm -> AMPm + PPIm+ LTyrTRNAm	BLASTP
AO090012000399	Tryptophan-tRNA synthetase	6.1.1.2	ATP + TRP + TRNA -> AMP + PPI + TRPTRNA	BLASTP
AO090009000378/ AO090026000265/ AO090120000226	Phenylalanyl-tRNA synthetase	6.1.1.20	ATP + PHE + TRNA -> AMP + PPI + LPheTRNA	BLASTP
**# Beta-alanine metabolism**				
AO090001000162	NAD-dependent aldehyde dehydrogenase	1.2.1.3	GABAL + NAD -> GABA + NADH	BLASTP
AO090023000467	NAD-dependent aldehyde dehydrogenase	1.2.1.3	GABAL + NAD -> GABA + NADH	BLASTP
AO090009000417	NAD-dependent aldehyde dehydrogenase	1.2.1.3	GABALm + NADm -> GABAm + NADHm	BLASTP
AO090009000417	NAD-dependent aldehyde dehydrogenase	1.2.1.3	GABAL + NAD -> GABA + NADH	BLASTP
ZY087055	NAD-dependent aldehyde dehydrogenase	1.2.1.3	GABAL + NAD -> GABA + NADH	EST
AO090001000162	NAD-dependent aldehyde dehydrogenase	1.2.1.3	LACAL + NAD <-> LLAC + NADH	BLASTP
AO090023000467	NAD-dependent aldehyde dehydrogenase	1.2.1.3	LACAL + NAD <-> LLAC + NADH	BLASTP
AO090009000417	NAD-dependent aldehyde dehydrogenase	1.2.1.3	LACALm + NADm <-> LLACm + NADHm	BLASTP
AO090009000417	NAD-dependent aldehyde dehydrogenase	1.2.1.3	LACAL + NAD <-> LLAC + NADH	BLASTP
ZY087055	NAD-dependent aldehyde dehydrogenase	1.2.1.3	LACAL + NAD <-> LLAC + NADH	EST
GAP	Dihydropyrimidine dehydrogenase (NADP)	1.3.1.2	URA + NADPH -> DIHURA + NADP	NONE
AO090009000580	Dihydropyrimidinase	3.5.2.2	DIHURA + H2O -> UREIPRO	GFAOP
AO090026000066	Dihydropyrimidinase	3.5.2.2	DIHURA + H2O -> UREIPRO	GFAOP
AO090003000384	Beta-alanine synthase	3.5.1.6	UREIPRO -> bALA + NH3 + CO2	GFAOP
**# Cyanoamino acid metabolism**				
AO090020000571	Nitrilase	3.5.5.1	ACYBUT -> GLU + NH3	BLASTP
AO090003000470	Nitrilase	3.5.5.1	ACYBUT -> GLU + NH3	BLASTP
AO090020000385	Nitrilase	3.5.5.1	ACYBUT -> GLU + NH3	BLASTP
AO090020000571	Nitrilase	3.5.5.1	APROP -> ALA + NH3	BLASTP
AO090003000470	Nitrilase	3.5.5.1	APROP -> ALA + NH3	BLASTP
AO090020000385	Nitrilase	3.5.5.1	APROP -> ALA + NH3	BLASTP

Gene name	Enzyme name	EC number	Biochemical reaction	Annotation method
**# Taurine and hypotaurine metabolism**				
No_Gene	Spontaneous conversion	No_EC	3SULALA -> CYSE	BLASTP
AO090103000342	Glutamate decarboxylase	4.1.1.15	CYSE -> TARE + CO2	BLASTP
AO090005000539	Glutamate decarboxylase	4.1.1.15	CYSE -> TARE + CO2	BLASTP
AO090003000666	Glutamate decarboxylase	4.1.1.15	CYSE -> TARE + CO2	BLASTP
AO090005000704	Glutamate decarboxylase	4.1.1.15	CYSE -> TARE + CO2	BLASTP
AO090009000076	Alpha-ketoglutarate-dependent taurine dioxygenase	1.14.11.17	TARE + AKG + O2 -> H2S + AMIACE + SUCC + CO2	BLASTP
AO090003000525	Alpha-ketoglutarate-dependent taurine dioxygenase	1.14.11.17	TARE + AKG + O2 -> H2S + AMIACE + SUCC + CO2	BLASTP
AO090023000531	Alpha-ketoglutarate-dependent taurine dioxygenase	1.14.11.17	TARE + AKG + O2 -> H2S + AMIACE + SUCC + CO2	BLASTP
AO090005001496	Alpha-ketoglutarate-dependent taurine dioxygenase	1.14.11.17	TARE + AKG + O2 -> H2S + AMIACE + SUCC + CO2	BLASTP
**# Glutathione biosynthesis**				
AO090012000764	Gamma-Glutamylcysteine synthetase	6.3.2.2	CYS + GLU + ATP -> GC + PI + ADP	BLASTP
AO090701000193	Glutathione synthetase	6.3.2.3	GLY + GC + ATP -> RGT + PI + ADP	GFAOP
AO090138000051	Glutathione synthetase	6.3.2.3	GLY + GC + ATP -> RGT + PI + ADP	GFAOP
AO090005000169	Gamma-glutamyltranspeptidase	2.3.2.2	RGT + ALA -> CGLY + ALAGLY	BLASTP
AO090009000211	Gamma-glutamyltranspeptidase	2.3.2.2	RGT + ALA -> CGLY + ALAGLY	BLASTP
AO090023000537	Gamma-glutamyltranspeptidase	2.3.2.2	RGT + ALA -> CGLY + ALAGLY	BLASTP
AO090113000029	Gamma-glutamyltranspeptidase	2.3.2.2	RGT + ALA -> CGLY + ALAGLY	BLASTP
GAP	Gamma-glutamylcyclotransferase	2.3.2.4	ALAGLY -> 5OXOPRO + ALA	NONE
GAP	Cysteinylglycinase aminopeptidase	3.4.11.2	CGLY + H2O -> GLY + CYS	NONE
AO090103000443	5-oxo-L-prolinase	3.5.2.9	ATP + 5OXOPRO + 2 H2O -> ADP + PI + GLU	BLASTP
AO090103000420	5-oxo-L-prolinase	3.5.2.9	ATP + 5OXOPRO + 2 H2O -> ADP + PI + GLU	BLASTP
AO090005000015	5-oxo-L-prolinase	3.5.2.9	ATP + 5OXOPRO + 2 H2O -> ADP + PI + GLU	BLASTP
AO090005000956	5-oxo-L-prolinase	3.5.2.9	ATP + 5OXOPRO + 2 H2O -> ADP + PI + GLU	BLASTP
AO090005000739	Glutathione peroxidase	1.11.1.9	2 RGT + H2O2 -> OGT + 2 H2O	BLASTP
AO090005001092	Glutathione reductase	1.6.4.2	NADPH + OGT -> NADP + RGT	BLASTP
AO090003000631	Glutathione S-transferase	2.5.1.18	RX + RGT -> HX + OGT	BLASTP
AO090005000973	Glutathione S-transferase	2.5.1.18	RX + RGT -> HX + OGT	BLASTP
AO090103000149	Glutathione S-transferase	2.5.1.18	RX + RGT -> HX + OGT	BLASTP
AO090103000485	Glutathione S-transferase	2.5.1.18	RX + RGT -> HX + OGT	BLASTP

Gene name	Enzyme name	EC number	Biochemical reaction	Annotation method
AO090102000005	Glutathione S-transferase	2.5.1.18	RX + RGT -> HX + OGT	BLASTP
AO090003001039	Glutathione S-transferase	2.5.1.18	RX + RGT -> HX + OGT	BLASTP
AO090103000134	Glutathione S-transferase	2.5.1.18	RX + RGT -> HX + OGT	BLASTP
AO090102000478	Glutathione S-transferase	2.5.1.18	RX + RGT -> HX + OGT	BLASTP
AO090012000378	Glutathione S-transferase	2.5.1.18	RX + RGT -> HX + OGT	BLASTP
AO090012000112	Glutathione S-transferase	2.5.1.18	RX + RGT -> HX + OGT	BLASTP
AO090023000353	Glutathione S-transferase	2.5.1.18	RX + RGT -> HX + OGT	BLASTP
ZY087096	Glutathione S-transferase	2.5.1.18	RX + RGT -> HX + OGT	EST
AO090005001092	Glutathione-disulfide reductase	1.8.1.7	NADPH + OGT -> NADP + 2 RGT	BLASTP
**# 4. Nucleotide metabolism**				
**# Purine Metabolism**				
AO090003001133	Ribose-phosphate pyrophosphokinase	2.7.6.1	R5P + ATP <-> PRPP + AMP	BLASTP
AO090005000432	Ribose-phosphate pyrophosphokinase	2.7.6.1	R5P + ATP <-> PRPP + AMP	BLASTP
AO090012000798	Ribose-phosphate pyrophosphokinase	2.7.6.1	R5P + ATP <-> PRPP + AMP	BLASTP
AO090001000512	Adenylate cyclase	4.6.1.1	ATP -> cAMP + PPI	BLASTP
AO090009000333	Phosphoribosyl-aminoimidazole carboxylase	4.1.1.21	CAIR <-> AIR + CO2	BLASTP
AO090020000395	Phosphoribosylaminoimidazole-succinocarboxamide synthase	6.3.2.6	CAIR + ATP + ASP <-> ADP + PI + SAICAR	BLASTP
AO090026000450	Adenylosuccinate lyase	4.3.2.2	SAICAR <-> FUM + AICAR	BLASTP
AO090103000467	Adenylosuccinate lyase	4.3.2.2	SAICAR <-> FUM + AICAR	BLASTP
AO090701000066	Phosphoribosylformylglycinamidine cyclo-ligase	6.3.3.1	FGAM + ATP -> ADP + PI + AIR	BLASTP
AO090102000389	Phosphoribosylformylglycinamidine synthase	6.3.5.3	FGAR + ATP + GLN -> GLU + ADP + PI + FGAM	BLASTP
AO090011000545	5-phosphoribosylglycinamide formyltransferase	2.1.2.2	GAR + FTHF -> THF + FGAR	GFAOP
AO090701000066	Phosphoribosylamine--glycine ligase	6.3.4.13	PRAM + ATP + GLY <-> ADP + PI + GAR	BLASTP
AO090701000173	Amidophosphoribosyltransferase	2.4.2.14	PRPP + GLN -> PPI + GLU + PRAM	BLASTP
AO090023000806	Phosphoribosylaminoimidazolecarboxamide formyltransferase	2.1.2.3	AICAR + FTHF <-> THF + PRFICA	BLASTP
AO090023000806	IMP cyclohydrolase	3.5.4.10	PRFICA <-> IMP	BLASTP

Gene name	Enzyme name	EC number	Biochemical reaction	Annotation method
AO090005001150	Adenylosuccinate synthetase	6.3.4.4	IMP + GTP + ASP -> GDP + PI + ASUC	BLASTP
AO090023000395	Argininosuccinate synthase	6.3.4.4	IMP + GTP + ASP -> GDP + PI + ASUC	BLASTP
AO090026000450	Adenylosuccinate lyase	4.3.2.2	ASUC <-> FUM + AMP	BLASTP
AO090103000467	Adenylosuccinate lyase	4.3.2.2	ASUC <-> FUM + AMP	BLASTP
AO090026000141	GMP synthase	6.3.5.2	XMP + ATP + GLN -> GLU + AMP + PPI + GMP	BLASTP
AO090011000307	GMP synthase	6.3.5.2	XMP + ATP + GLN -> GLU + AMP + PPI + GMP	BLASTP
AO090005001643	Guanylate kinase	2.7.4.8	GMP + ATP <-> GDP + ADP	BLASTP
AO090005001643	Guanylate kinase	2.7.4.8	DGMP + ATP <-> DGDP + ADP	BLASTP
AO090005001643	Guanylate kinase	2.7.4.8	GMP + DATP <-> GDP + DADP	BLASTP
AO090010000778	Adenosine monophosphate deaminase	3.5.4.6	AMP + H2O -> IMP + NH3	BLASTP
AO090003000819	3'5'-cyclic nucleotide phosphodiesterase	3.1.4.17	cAMP -> AMP	BLASTP
AO090003000820	3'5'-cyclic nucleotide phosphodiesterase	3.1.4.17	cAMP -> AMP	BLASTP
AO090005001243	3'5'-cyclic nucleotide phosphodiesterase	3.1.4.17	cAMP -> AMP	BLASTP
AO090003000819	3'5'-cyclic nucleotide phosphodiesterase	3.1.4.17	cdAMP -> DAMP	BLASTP
AO090003000820	3'5'-cyclic nucleotide phosphodiesterase	3.1.4.17	cdAMP -> DAMP	BLASTP
AO090005001243	3'5'-cyclic nucleotide phosphodiesterase	3.1.4.17	cdAMP -> DAMP	BLASTP
AO090003000819	3'5'-cyclic nucleotide phosphodiesterase	3.1.4.17	cIMP -> IMP	BLASTP
AO090003000820	3'5'-cyclic nucleotide phosphodiesterase	3.1.4.17	cIMP -> IMP	BLASTP
AO090005001243	3'5'-cyclic nucleotide phosphodiesterase	3.1.4.17	cIMP -> IMP	BLASTP
AO090003000819	3'5'-cyclic nucleotide phosphodiesterase	3.1.4.17	cGMP -> GMP	BLASTP
AO090003000820	3'5'-cyclic nucleotide phosphodiesterase	3.1.4.17	cGMP -> GMP	BLASTP
AO090005001243	3'5'-cyclic nucleotide phosphodiesterase	3.1.4.17	cGMP -> GMP	BLASTP
AO090003000819	3'5'-cyclic nucleotide phosphodiesterase	3.1.4.17	cCMP -> CMP	BLASTP
AO090003000820	3'5'-cyclic nucleotide phosphodiesterase	3.1.4.17	cCMP -> CMP	BLASTP
AO090005001243	3'5'-cyclic nucleotide phosphodiesterase	3.1.4.17	cCMP -> CMP	BLASTP
AO090701000748	ADP-ribose diphosphatase	3.6.1.13	ADPRIB + H2O -> AMP + R5P	BLASTP
AO090138000061	ADP-ribose diphosphatase	3.6.1.13	ADPRIB + H2O -> AMP + R5P	BLASTP
AO090010000228	Inosine triphosphate pyrophosphatase	3.6.1.19	ITP + H2O -> IMP + PPI	BLASTP
AO090010000228	Inosine triphosphate pyrophosphatase	3.6.1.19	GTP + H2O -> GMP + PPI	GFAOP
AO090010000228	Inosine triphosphate pyrophosphatase	3.6.1.19	DGTP + H2O -> DGMP + PPI	BLASTP

Gene name	Enzyme name	EC number	Biochemical reaction	Annotation method
AO090011000350	Guanine deaminase	3.5.4.3	GN + H2O -> XAN + NH3	BLASTP
AO090023000256	Guanine deaminase	3.5.4.3	GN + H2O -> XAN + NH3	BLASTP
GAP	GTP diphosphokinase	2.7.6.5	ATP+ GTP -> AMP + pppGpp	NONE
AO090102000550	Exopolyphosphatase	3.6.1.11	pppGpp + H2O <-> ppGpp + PI	BLASTP
GAP	Guanosine-3',5'-bis(diphosphate) 3'-diphosphatase	3.1.7.2	ppGpp + H2O <-> GDP + PPI	NONE
AO090003001099	Xanthine dehydrogenase	1.17.1.4	XANp + NADp + H2Op -> URATEp + NADHp	BLASTP
AO090003001099	Xanthine oxidase	1.17.3.2	XANp + H2Op + O2p -> URATEp + H2O2p	BLASTP
AO090011000588	Urate oxidase	1.7.3.3	URATEp + O2p + H2Op -> HIURTEp + H2O2p	BLASTP
No_Gene	Hydroxyisourate hydrolase (Spontaneous conversion)	3.5.2.17	HIURTE + H2O -> ATN + CO2	BLASTP
AO090001000480	IMP dehydrogenase	1.1.1.205	IMP + NAD -> NADH + XMP	BLASTP
AO090001000481	IMP dehydrogenase	1.1.1.205	IMP + NAD -> NADH + XMP	BLASTP
AO090120000224	IMP dehydrogenase	1.1.1.205	IMP + NAD -> NADH + XMP	BLASTP
**# Pyrimidine metabolism**				
AO090023000483/ AO090011000630	Aspartate-carbamoyltransferase	2.1.3.2	CAP + ASP -> CAASP + PI	BLASTP
AO090005001052	Dihydroorotase	3.5.2.3	CAASP <-> DOROA	GFAOP
AO090026000542	Dihydroorotate oxidase	1.3.3.1	DOROAm + O2m <-> H2O2m + OROAm	BLASTP
AO090026000521	Orotate phosphoribosyltransferase	2.4.2.10	OROA + PRPP <-> PPI + OMP	BLASTP
AO090011000868	Orotidine 5'-phosphate decarboxylase	4.1.1.23	OMP -> CO2 + UMP	BLASTP
AO090026000815	Uridylate kinase	2.7.4.22	ATP + UMP <-> ADP + UDP	BLASTP
AO090009000714	Uracil phosphoribosyltransferase	2.4.2.9	URA + PRPP -> UMP + PPI	BLASTP
AO090102000246	Uracil phosphoribosyltransferase	2.4.2.9	URA + PRPP -> UMP + PPI	BLASTP
AO090009000714	Cytosine deaminase	3.5.4.1	CYTS + H2O -> URA + NH3	BLASTP
AO090003000802	Cytosine deaminase	3.5.4.1	CYTS + H2O -> URA + NH3	BLASTP
AO090001000654	Uridine kinase	2.7.1.48	URI + GTP <-> UMP + GDP	BLASTP
AO090001000654	Uridine kinase	2.7.1.48	CYTD + GTP -> GDP + CMP	BLASTP
AO090001000654	Uridine kinase	2.7.1.48	URI + ATP -> ADP + UMP	BLASTP
GAP	Thymidine phosphorylase	2.4.2.4	DU + PI <-> URA + DR1P	NONE

Gene name	Enzyme name	EC number	Biochemical reaction	Annotation method
GAP	Thymidine phosphorylase	2.4.2.4	DT + PI <-> THY + DR1P	NONE
AO090026000800	Cytidine deaminase	3.5.4.5	CYTD + H2O -> URI + NH3	BLASTP
AO090026000800	Cytidine deaminase	3.5.4.5	DC -> NH3 + DU	GFAOP
ZY007112	Cytidine deaminase	3.5.4.5	CYTD + H2O -> URI + NH3	EST
ZY007112	Cytidine deaminase	3.5.4.5	DC -> NH3 + DU	EST
AO090009000289	Thioredoxin reductase	1.8.1.9	OTHIO + NADPH -> NADP + RTHIO	BLASTP
AO090009000289	Thioredoxin reductase	1.8.1.9	OTHIOm + NADPHm -> NADPm + RTHIOm	BLASTP
AO090005000780	dUTPase	3.6.1.23	DUTP + H2O -> PPI + DUMP	BLASTP
AO090206000066	Thymidylate synthase	2.1.1.45	DUMP + METTHF -> DHF + DTMP	BLASTP
AO090026000606	CTP synthase	6.3.4.2	UTP + GLN + ATP -> GLU + CTP + ADP + PI	BLASTP
AO090026000606	CTP synthase	6.3.4.2	ATP + UTP + NH3 -> ADP + PI + CTP	BLASTP
AO090009000691	Pseudouridylate synthase	4.2.1.70	URA + R5P <-> PURI5P	BLASTP
AO090120000348	Pseudouridylate synthase	4.2.1.70	URA + R5P <-> PURI5P	BLASTP
AO090011000855	Pseudouridylate synthase	4.2.1.70	URA + R5P <-> PURI5P	BLASTP
AO090001000741	Deoxycytidylate deaminase	3.5.4.12	DCMP + H2O -> DUMP + NH3	BLASTP
AO090001000742	Deoxycytidylate deaminase	3.5.4.12	DCMP + H2O -> DUMP + NH3	BLASTP
AO090009000391	Uridine nucleosidase	3.2.2.3	URI + H2O -> URA + RIB	GFAOP
AO090023000483	Carbamoyl-phosphate synthase (glutamine-hydrolysing)	6.3.5.5	GLN + 2 ATP + CO2 -> GLU + CAP + 2 ADP + PI	BLASTP
AO090701000214/ AO090010000199/ AO090011000630	Carbamoyl-phosphate synthase (glutamine-hydrolysing)	6.3.5.5	GLNm + 2 ATPm + CO2m -> GLUm + CAPm + 2 ADPm + PIm	BLASTP
AO090010000228	Inosine triphosphate pyrophosphatase	3.6.1.19	UTP + H2O -> UMP + PPI	BLASTP
AO090010000228	Inosine triphosphate pyrophosphatase	3.6.1.19	DUTP + H2O -> DUMP + PPI	BLASTP
AO090206000067	Queuine tRNA-ribosyltransferase	2.1.1.45	DUMPm + METTHFm -> DHFm + DTMPm	BLASTP
**# Nucleotide salvage pathway**				
AO090038000610	Adenine phosphoribosyltransferase	2.4.2.7	AD + PRPP -> PPI + AMP	BLASTP
AO090011000781	Adenosine deaminase	3.5.4.4	ADN -> INS + NH3	BLASTP
AO090012000559	Adenosine deaminase	3.5.4.4	ADN -> INS + NH3	BLASTP

Gene name	Enzyme name	EC number	Biochemical reaction	Annotation method
AO090012000959	Adenosine deaminase	3.5.4.4	ADN -> INS + NH3	BLASTP
AO090001000546	Purine-nucleoside phosphorylase	2.4.2.1	DIN + PI <-> HYXN + DR1P	GFAOP
AO090001000546	Purine-nucleoside phosphorylase	2.4.2.1	DA + PI <-> AD + DR1P	GFAOP
AO090001000546	Purine-nucleoside phosphorylase	2.4.2.1	DG + PI <-> GN + DR1P	GFAOP
AO090001000546	Purine-nucleoside phosphorylase	2.4.2.1	HYXN + R1P <-> INS + PI	GFAOP
AO090001000546	Purine-nucleoside phosphorylase	2.4.2.1	AD + R1P <-> PI + ADN	GFAOP
AO090001000546	Purine-nucleoside phosphorylase	2.4.2.1	XAN + R1P <-> PI + XTSINE	GFAOP
AO090001000546	Purine-nucleoside phosphorylase	2.4.2.1	GSN + PI <-> GN + R1P	GFAOP
AO090206000037	Xanthine-guanine phosphoribosyltransferase	2.4.2.22	XAN + PRPP -> XMP + PPI	BLASTP
AO090038000241	Purine nucleosidase	3.2.2.1	GSN -> GN + RIB	BLASTP
AO090038000241	Purine nucleosidase	3.2.2.1	ADN -> AD + RIB	BLASTP
AO090009000617	Adenosine kinase	2.7.1.20	ADN + ATP -> AMP + ADP	BLASTP
AO090012001004	Adenylate kinase	2.7.4.3	ATP + AMP <-> 2 ADP	BLASTP
AO090012001004	Adenylate kinase	2.7.4.3	GTP + AMP <-> ADP + GDP	BLASTP
AO090012001004	Adenylate kinase	2.7.4.3	ITP + AMP <-> ADP + IDP	BLASTP
AO090005000758	Adenylate kinase	2.7.4.3	ATPm + AMPm <-> 2 ADPm	BLASTP
AO090005000758	Adenylate kinase	2.7.4.3	GTPm + AMPm <-> ADPm + GDPm	BLASTP
AO090005000758	Adenylate kinase	2.7.4.3	ITPm + AMPm <-> ADPm + IDPm	BLASTP
AO090102000558	Nucleoside diphosphate kinase	2.7.4.6	UDP + ATP <-> UTP + ADP	GFAOP
AO090102000558	Nucleoside diphosphate kinase	2.7.4.6	CDP + ATP <-> CTP + ADP	GFAOP
AO090102000558	Nucleoside diphosphate kinase	2.7.4.6	DGDP + ATP <-> DGTP + ADP	GFAOP
AO090102000558	Nucleoside diphosphate kinase	2.7.4.6	DUDP + ATP <-> DUTP + ADP	GFAOP
AO090102000558	Nucleoside diphosphate kinase	2.7.4.6	DCDP + ATP <-> DCTP + ADP	GFAOP
AO090102000558	Nucleoside diphosphate kinase	2.7.4.6	DTDP + ATP <-> DTTP + ADP	GFAOP
AO090102000558	Nucleoside diphosphate kinase	2.7.4.6	DADP + ATP <-> DATP + ADP	GFAOP
AO090102000558	Nucleoside diphosphate kinase	2.7.4.6	GDP + ATP <-> GTP + ADP	GFAOP
AO090102000558	Nucleoside diphosphate kinase	2.7.4.6	IDP + ATP <-> ITP + ADP	GFAOP
AO090012001004	Deoxy-adenylate kinase	2.7.4.11	DAMP + ATP <-> DADP + ADP	GFAOP
AO090005000758	Deoxy-adenylate kinase	2.7.4.11	DAMP + ATP <-> DADP + ADP	GFAOP

Gene name	Enzyme name	EC number	Biochemical reaction	Annotation method
AO090012001004	Adenylate kinase	2.7.4.3	ATP + AMP <-> 2 ADP	BLASTP
AO090005000758	Adenylate kinase	2.7.4.3	ATP + AMP <-> 2 ADP	BLASTP
AO090011000781	Adenosine deaminase	3.5.4.2	AD -> NH3 + HYXN	BLASTP
AO090009000617	Adenosine kinase	2.7.1.73	INS + ATP -> IMP + ADP	GFAOP
AO090009000617	Adenosine kinase	2.7.1.73	GSN + ATP -> GMP + ADP	GFAOP
AO090206000037	Xanthine-guanine phosphoribosyltransferase	2.4.2.8	HYXN + PRPP -> PPI + IMP	BLASTP
AO090206000037	Xanthine-guanine phosphoribosyltransferase	2.4.2.8	GN + PRPP -> PPI + GMP	BLASTP
GAP	Pyrimidine-5'-nucleotide nucleosidase	3.2.2.10	CMP -> CYTS + R5P	NONE
AO090103000444	5'-nucleotidase	3.1.3.5	DUMP -> DU + PI	BLASTP
AO090103000444	5'-nucleotidase	3.1.3.5	DTMP -> DT + PI	BLASTP
AO090103000444	5'-nucleotidase	3.1.3.5	DAMP -> DA + PI	BLASTP
AO090103000444	5'-nucleotidase	3.1.3.5	DGMP -> DG + PI	BLASTP
AO090103000444	5'-nucleotidase	3.1.3.5	DCMP -> DC + PI	BLASTP
AO090103000444	5'-nucleotidase	3.1.3.5	CMP -> CYTD + PI	BLASTP
AO090103000444	5'-nucleotidase	3.1.3.5	AMP -> PI + ADN	BLASTP
AO090103000444	5'-nucleotidase	3.1.3.5	GMP -> PI + GSN	BLASTP
AO090103000444	5'-nucleotidase	3.1.3.5	IMP -> PI + INS	BLASTP
AO090103000444	5'-nucleotidase	3.1.3.5	XMP -> PI + XTSINE	BLASTP
AO090103000444	5'-nucleotidase	3.1.3.5	UMP -> PI + URI	BLASTP
AO090023000916	Ribonucleoside-diphosphate reductase	1.17.4.1	ADP + RTHIO -> DADP + OTHIO	BLASTP
AO090023000916	Ribonucleoside-diphosphate reductase	1.17.4.1	GDP + RTHIO -> DGDP + OTHIO	BLASTP
AO090023000916	Ribonucleoside-diphosphate reductase	1.17.4.1	CDP + RTHIO -> DCDP + OTHIO	BLASTP
AO090023000916	Ribonucleoside-diphosphate reductase	1.17.4.1	UDP + RTHIO -> DUDP + OTHIO	BLASTP
AO090120000352	Ribonucleoside-triphosphate reductase	1.17.4.2	ATP + RTHIO -> DATP + OTHIO	BLASTP
AO090120000352	Ribonucleoside-triphosphate reductase	1.17.4.2	GTP + RTHIO -> DGTP + OTHIO	BLASTP
AO090120000352	Ribonucleoside-triphosphate reductase	1.17.4.2	CTP + RTHIO -> DCTP + OTHIO	BLASTP
AO090120000352	Ribonucleoside-triphosphate reductase	1.17.4.2	UTP + RTHIO -> DUTP + OTHIO	BLASTP
AO090001000329	Nucleoside diphosphatase	3.6.1.6	UDP + H2O -> UMP + PI	BLASTP
AO090026000750	Nucleoside diphosphatase	3.6.1.6	UDP + H2O -> UMP + PI	BLASTP
AO090001000329	Nucleoside diphosphatase	3.6.1.6	GDP + H2O -> GMP + PI	BLASTP
AO090026000750	Nucleoside diphosphatase	3.6.1.6	GDP + H2O -> GMP + PI	BLASTP

Gene name	Enzyme name	EC number	Biochemical reaction	Annotation method
AO090102000555	Thymidylate kinase	2.7.4.9	DTMP + ATP <-> ADP + DTDP	BLASTP
AO090026000815	Uridylate kinase	2.7.4.14	DCMP + ATP <-> ADP + DCDP	BLASTP
AO090026000815	Uridylate kinase	2.7.4.14	CMP + ATP <-> ADP + CDP	BLASTP
AO090026000815	Uridylate kinase	2.7.4.14	UMP + ATP <-> ADP + UDP	BLASTP
**# 5. Lipid metabolism**				
**# Fatty acid biosynthesis**				
AO090124000084	Acetyl coenzyme A-acyl-carrier-protein transacylase	2.3.1.38	ACCOA + ACP -> ACACP + COA	BLASTP
AO090011000040	Acetyl coenzyme A-acyl-carrier-protein transacylase	2.3.1.38	ACCOA + ACP -> ACACP + COA	BLASTP
AO090011000838/ ZY098176	Acetyl-CoA carboxylase	6.4.1.2	CBCCP + ACCOA <-> BCCP + MALCOA	EST, GFAOP
AO090672000003	Malonyl CoA-acyl carrier protein transacylase	2.3.1.39	MALCOA + ACP <-> MALACP + COA	GFAOP
AO090124000084	Malonyl CoA-acyl carrier protein transacylase	2.3.1.39	MALCOA + ACP <-> MALACP + COA	GFAOP
AO090011000040	Malonyl CoA-acyl carrier protein transacylase	2.3.1.39	MALCOA + ACP <-> MALACP + COA	GFAOP
AO090011000838	Biotin carboxylase	6.3.4.14	ATP + BCCP + CO2 -> ADP + PI + CBCCP	BLASTP
AO090001000695	3-oxoacyl-[acyl-carrier-protein] synthase I	2.3.1.41	ACACP + MALACP -> AACACP + CO2 + ACP	BLASTP
AO090023000924/ AO090023000082/ AO090003000520/ AO090005000145/ AO090010000429/ AO090011000370	3-oxoacyl-[acyl-carrier-protein] reductase	1.1.1.100	AACACP + NADPH <-> C4HACP + NADP	BLASTP
AO090124000084	Crotonoyl-[acyl-carrier-protein] hydratase	4.2.1.58	C4HACP <-> C4DACP + H2O	BLASTP
AO090011000040	Crotonoyl-[acyl-carrier-protein] hydratase	4.2.1.58	C4HACP <-> C4DACP + H2O	BLASTP
AO090124000084	Enoyl-[acyl-carrier-protein] reductase (NADH)	1.3.1.9	C4DACP + NADPH <-> C40ACP + NADP	BLASTP
AO090011000040	Enoyl-[acyl-carrier-protein] reductase (NADH)	1.3.1.9	C4DACP + NADPH <-> C40ACP + NADP	BLASTP

Gene name	Enzyme name	EC number	Biochemical reaction	Annotation method
AO090010000107/<br>AO090010000171/<br>AO090026000012/<br>AO090124000083/<br>AO090010000156/<br>AO090011000040/<br>AO090124000084/<br>AO090011000046	Fatty-acid synthase	2.3.1.85	ACACP + MALACP + 2 NADPH -> C40ACP + ACP + 2 NADP + H2O + CO2	BLASTP
AO090001000695	3-oxoacyl-[acyl-carrier-protein] synthase I	2.3.1.41	C40ACP + MALACP -> C6OACP + CO2 + ACP	BLASTP
AO090023000924/<br>AO090023000082/<br>AO090003000520/<br>AO090005000145/<br>AO090010000429/<br>AO090011000370	3-oxoacyl-[acyl-carrier-protein] reductase	1.1.1.100	C6OACP + NADPH <-> C6HACP + NADP	BLASTP
AO090124000084	Crotonoyl-[acyl-carrier-protein] hydratase	4.2.1.58	C6HACP <-> C6DACP + H2O	BLASTP
AO090011000040	Crotonoyl-[acyl-carrier-protein] hydratase	4.2.1.58	C6HACP <-> C6DACP + H2O	BLASTP
AO090124000084	Enoyl-[acyl-carrier-protein] reductase (NADH)	1.3.1.9	C6DACP + NADPH <-> C60ACP + NADP	BLASTP
AO090011000040	Enoyl-[acyl-carrier-protein] reductase (NADH)	1.3.1.9	C6DACP + NADPH <-> C60ACP + NADP	BLASTP
AO090010000107/<br>AO090010000171/<br>AO090026000012/<br>AO090124000083/<br>AO090010000156/<br>AO090011000040/<br>AO090124000084/<br>AO090011000046	Fatty-acid synthase	2.3.1.85	C40ACP + MALACP + 2 NADPH -> C60ACP + ACP + 2 NADP + H2O + CO2	BLASTP

Gene name	Enzyme name	EC number	Biochemical reaction	Annotation method
AO090001000695	3-oxoacyl-[acyl-carrier-protein] synthase I	2.3.1.41	C60ACP + MALACP -> C8OACP + CO2 + ACP	BLASTP
AO090023000924/ AO090023000082/ AO090003000520/ AO090005000145/ AO090010000429/ AO090011000370	3-oxoacyl-[acyl-carrier-protein] reductase	1.1.1.100	C8OACP + NADPH <-> C8HACP + NADP	BLASTP
AO090124000084	Crotonoyl-[acyl-carrier-protein] hydratase	4.2.1.58	C8HACP <-> C8DACP + H2O	BLASTP
AO090011000040	Crotonoyl-[acyl-carrier-protein] hydratase	4.2.1.58	C8HACP <-> C8DACP + H2O	BLASTP
AO090124000084	Enoyl-[acyl-carrier-protein] reductase (NADH)	1.3.1.9	C8DACP + NADPH <-> C80ACP + NADP	BLASTP
AO090011000040	Enoyl-[acyl-carrier-protein] reductase (NADH)	1.3.1.9	C8DACP + NADPH <-> C80ACP + NADP	BLASTP
AO090010000107/ AO090010000171/ AO090026000012/ AO090124000083/ AO090010000156/ AO090011000040/ AO090124000084/ AO090011000046	Fatty-acid synthase	2.3.1.85	C60ACP + MALACP + 2 NADPH -> C80ACP + ACP + 2 NADP + H2O + CO2	BLASTP
AO090001000695	3-oxoacyl-[acyl-carrier-protein] synthase I	2.3.1.41	C80ACP + MALACP -> C10OACP + CO2 + ACP	BLASTP
AO090023000924/ AO090023000082/ AO090003000520/ AO090005000145/ AO090010000429/ AO090011000370	3-oxoacyl-[acyl-carrier-protein] reductase	1.1.1.100	C10OACP + NADPH <-> C10HACP + NADP	BLASTP
AO090124000084	3-hydroxydecanoyl-[acyl-carrier-protein] dehydratase	4.2.1.60	C10HACP <-> C10DACP + H2O	BLASTP
AO090011000040	3-hydroxydecanoyl-[acyl-carrier-protein] dehydratase	4.2.1.60	C10HACP <-> C10DACP + H2O	BLASTP
AO090124000084	Enoyl-[acyl-carrier-protein] reductase (NADH)	1.3.1.9	C10DACP + NADPH <-> C100ACP + NADP	BLASTP
AO090011000040	Enoyl-[acyl-carrier-protein] reductase (NADH)	1.3.1.9	C10DACP + NADPH <-> C100ACP + NADP	BLASTP

Gene name	Enzyme name	EC number	Biochemical reaction	Annotation method
AO090010000107/ AO090010000171/ AO090026000012/ AO090124000083/ AO090010000156/ AO090011000040/ AO090124000084/ AO090011000046	Fatty-acid synthase	2.3.1.85	C80ACP + MALACP + 2 NADPH -> C100ACP + ACP + 2 NADP + H2O + CO2	BLASTP
AO090001000695	3-oxoacyl-[acyl-carrier-protein] synthase I	2.3.1.41	C100ACP + MALACP -> C12OACP + CO2 + ACP	BLASTP
AO090023000924/ AO090023000082/ AO090003000520/ AO090005000145/ AO090010000429/ AO090011000370	3-oxoacyl-[acyl-carrier-protein] reductase	1.1.1.100	C12OACP + NADPH <-> C12HACP + NADP	BLASTP
AO090124000084	3-hydroxypalmitoyl-[acyl-carrier-protein] dehydratase	4.2.1.61	C12HACP <-> C12DACP + H2O	BLASTP
AO090011000040	3-hydroxypalmitoyl-[acyl-carrier-protein] dehydratase	4.2.1.61	C12HACP <-> C12DACP + H2O	BLASTP
AO090124000084	Enoyl-[acyl-carrier-protein] reductase (NADH)	1.3.1.9	C12DACP + NADPH <-> C120ACP + NADP	BLASTP
AO090011000040	Enoyl-[acyl-carrier-protein] reductase (NADH)	1.3.1.9	C12DACP + NADPH <-> C120ACP + NADP	BLASTP
AO090010000107/ AO090010000171/ AO090026000012/ AO090124000083/ AO090010000156/ AO090011000040/ AO090124000084/ AO090011000046	Fatty-acid synthase	2.3.1.85	C100ACP + MALACP + 2 NADPH -> C120ACP + ACP + 2 NADP + H2O + CO2	BLASTP
AO090001000695	3-oxoacyl-[acyl-carrier-protein] synthase I	2.3.1.41	C120ACP + MALACP -> C14OACP + CO2 + ACP	BLASTP

Gene name	Enzyme name	EC number	Biochemical reaction	Annotation method
AO090023000924/ AO090023000082/ AO090003000520/ AO090005000145/ AO090010000429/ AO090011000370	3-oxoacyl-[acyl-carrier-protein] reductase	1.1.1.100	C14OACP + NADPH <-> C14HACP + NADP	BLASTP
AO090124000084	3-hydroxypalmitoyl-[acyl-carrier-protein] dehydratase	4.2.1.61	C14HACP <-> C14DACP + H2O	BLASTP
AO090011000040	3-hydroxypalmitoyl-[acyl-carrier-protein] dehydratase	4.2.1.61	C14HACP <-> C14DACP + H2O	BLASTP
AO090124000084	Enoyl-[acyl-carrier-protein] reductase (NADH)	1.3.1.9	C14DACP + NADPH <-> C140ACP + NADP	BLASTP
AO090011000040	Enoyl-[acyl-carrier-protein] reductase (NADH)	1.3.1.9	C14DACP + NADPH <-> C140ACP + NADP	BLASTP
AO090010000107/ AO090010000171/ AO090026000012/ AO090124000083/ AO090010000156/ AO090011000040/ AO090124000084/ AO090011000046	Fatty-acid synthase	2.3.1.85	C120ACP + MALACP + 2 NADPH -> C140ACP + ACP + 2 NADP + H2O + CO2	BLASTP
AO090001000695	3-oxoacyl-[acyl-carrier-protein] synthase I	2.3.1.41	C140ACP + MALACP -> C16OACP + CO2 + ACP	BLASTP
AO090023000924/ AO090023000082/ AO090003000520/ AO090005000145/ AO090010000429/ AO090011000370	3-oxoacyl-[acyl-carrier-protein] reductase	1.1.1.100	C16OACP + NADPH <-> C16HACP + NADP	BLASTP
AO090124000084	3-hydroxypalmitoyl-[acyl-carrier-protein] dehydratase	4.2.1.61	C16HACP <-> C16DACP + H2O	BLASTP
AO090011000040	3-hydroxypalmitoyl-[acyl-carrier-protein] dehydratase	4.2.1.61	C16HACP <-> C16DACP + H2O	BLASTP
AO090124000084	Enoyl-[acyl-carrier-protein] reductase (NADH)	1.3.1.9	C16DACP + NADPH <-> C160ACP + NADP	BLASTP
AO090011000040	Enoyl-[acyl-carrier-protein] reductase (NADH)	1.3.1.9	C16DACP + NADPH <-> C160ACP + NADP	BLASTP

Gene name	Enzyme name	EC number	Biochemical reaction	Annotation method
AO090010000107/ AO090010000171/ AO090026000012/ AO090124000083/ AO090010000156/ AO090011000040/ AO090124000084/ AO090011000046	Fatty-acid synthase	2.3.1.85	C140ACP + MALACP + 2 NADPH -> C160ACP + ACP + 2 NADP + H2O + CO2	BLASTP
AO090001000695	3-oxoacyl-[acyl-carrier-protein] synthase II	2.3.1.179	C160ACP + MALACP -> C18OACP + CO2 + ACP	BLASTP
AO090023000924/ AO090023000082/ AO090003000520/ AO090005000145/ AO090010000429/ AO090011000370	3-oxoacyl-[acyl-carrier-protein] reductase	1.1.1.100	C18OACP + NADPH <-> C18HACP + NADP	BLASTP
AO090124000084	3-hydroxypalmitoyl-[acyl-carrier-protein] dehydratase	4.2.1.61	C18HACP <-> C18DACP + H2O	BLASTP
AO090011000040	3-hydroxypalmitoyl-[acyl-carrier-protein] dehydratase	4.2.1.61	C18HACP <-> C18DACP + H2O	BLASTP
AO090124000084	Enoyl-[acyl-carrier-protein] reductase (NADH)	1.3.1.9	C18DACP + NADPH <-> C180ACP + NADP	BLASTP
AO090011000040	Enoyl-[acyl-carrier-protein] reductase (NADH)	1.3.1.9	C18DACP + NADPH <-> C180ACP + NADP	BLASTP
AO090010000107/ AO090010000171/ AO090026000012/ AO090124000083/ AO090010000156/ AO090011000040/ AO090124000084/ AO090011000046	Fatty-acid synthase	2.3.1.85	C160ACP + MALACP + 2 NADPH -> C180ACP + ACP + 2 NADP + H2O + CO2	BLASTP
AO090005000456	Stearoyl-CoA 9-desaturase	1.14.19.1	C180ACP + NADH + O2 -> C181ACP + NAD + 2 H2O	BLASTP
AO090102000339	Stearoyl-CoA 9-desaturase	1.14.19.1	C180ACP + NADH + O2 -> C181ACP + NAD + 2 H2O	BLASTP
AO090026000799	Stearoyl-CoA 9-desaturase	1.14.19.1	C180ACP + NADH + O2 -> C181ACP + NAD + 2 H2O	BLASTP

Gene name	Enzyme name	EC number	Biochemical reaction	Annotation method
AO090103000283	Stearoyl-CoA 9-desaturase	1.14.19.1	C180ACP + NADH + O2 -> C181ACP + NAD + 2 H2O	BLASTP
AO090001000224	Oleate delta-12 desaturase	1.14.19.3	C181ACP + NADH + O2 -> C182ACP + NAD + 2 H2O	BLASTP
AO090010000714	Oleate delta-12 desaturase	1.14.19.3	C181ACP + NADH + O2 -> C182ACP + NAD + 2 H2O	BLASTP
GAP	Stearoyl-CoA 15-desaturase	1.14.19.-	C182ACP + NADH + O2 -> C183ACP + NAD + 2 H2O	NONE
AO090005000456	Stearoyl-CoA 9-desaturase	1.14.19.1	C160ACP + NADH + O2 -> C161ACP + NAD + 2 H2O	BLASTP
AO090102000339	Stearoyl-CoA 9-desaturase	1.14.19.1	C160ACP + NADH + O2 -> C161ACP + NAD + 2 H2O	BLASTP
AO090026000799	Stearoyl-CoA 9-desaturase	1.14.19.1	C160ACP + NADH + O2 -> C161ACP + NAD + 2 H2O	BLASTP
AO090103000283	Stearoyl-CoA 9-desaturase	1.14.19.1	C160ACP + NADH + O2 -> C161ACP + NAD + 2 H2O	BLASTP
AO090001000224	Oleate delta-12 desaturase	1.14.19.3	C161ACP + NADH + O2 -> C162ACP + NAD + 2 H2O	BLASTP
AO090010000714	Oleate delta-12 desaturase	1.14.19.3	C161ACP + NADH + O2 -> C162ACP + NAD + 2 H2O	BLASTP
AO090124000084	Acyl-[acyl-carrier-protein] hydrolase	3.1.2.14	C40ACP + H2O -> C40 + ACP	BLASTP
AO090011000040	Acyl-[acyl-carrier-protein] hydrolase	3.1.2.14	C40ACP + H2O -> C40 + ACP	BLASTP
AO090124000084	Acyl-[acyl-carrier-protein] hydrolase	3.1.2.14	C60ACP + H2O -> C60 + ACP	BLASTP
AO090011000040	Acyl-[acyl-carrier-protein] hydrolase	3.1.2.14	C60ACP + H2O -> C60 + ACP	BLASTP
AO090124000084	Acyl-[acyl-carrier-protein] hydrolase	3.1.2.14	C80ACP + H2O -> C80 + ACP	BLASTP
AO090011000040	Acyl-[acyl-carrier-protein] hydrolase	3.1.2.14	C80ACP + H2O -> C80 + ACP	BLASTP
AO090124000084	Acyl-[acyl-carrier-protein] hydrolase	3.1.2.14	C100ACP + H2O -> C100 + ACP	BLASTP
AO090011000040	Acyl-[acyl-carrier-protein] hydrolase	3.1.2.14	C100ACP + H2O -> C100 + ACP	BLASTP
AO090124000084	Acyl-[acyl-carrier-protein] hydrolase	3.1.2.14	C120ACP + H2O -> C120 + ACP	BLASTP
AO090011000040	Acyl-[acyl-carrier-protein] hydrolase	3.1.2.14	C120ACP + H2O -> C120 + ACP	BLASTP
AO090124000084	Acyl-[acyl-carrier-protein] hydrolase	3.1.2.14	C140ACP + H2O -> C140 + ACP	BLASTP
AO090011000040	Acyl-[acyl-carrier-protein] hydrolase	3.1.2.14	C140ACP + H2O -> C140 + ACP	BLASTP
AO090124000084	Acyl-[acyl-carrier-protein] hydrolase	3.1.2.14	C160ACP + H2O -> C160 + ACP	BLASTP
AO090011000040	Acyl-[acyl-carrier-protein] hydrolase	3.1.2.14	C160ACP + H2O -> C160 + ACP	BLASTP
AO090124000084	Acyl-[acyl-carrier-protein] hydrolase	3.1.2.14	C161ACP + H2O -> C161 + ACP	BLASTP
AO090011000040	Acyl-[acyl-carrier-protein] hydrolase	3.1.2.14	C161ACP + H2O -> C161 + ACP	BLASTP
AO090124000084	Acyl-[acyl-carrier-protein] hydrolase	3.1.2.14	C162ACP + H2O -> C162 + ACP	BLASTP
AO090011000040	Acyl-[acyl-carrier-protein] hydrolase	3.1.2.14	C162ACP + H2O -> C162 + ACP	BLASTP
AO090124000084	Acyl-[acyl-carrier-protein] hydrolase	3.1.2.14	C180ACP + H2O -> C180 + ACP	BLASTP
AO090011000040	Acyl-[acyl-carrier-protein] hydrolase	3.1.2.14	C180ACP + H2O -> C180 + ACP	BLASTP

Gene name	Enzyme name	EC number	Biochemical reaction	Annotation method
AO090124000084	Acyl-[acyl-carrier-protein] hydrolase	3.1.2.14	C181ACP + H2O -> C181 + ACP	BLASTP
AO090011000040	Acyl-[acyl-carrier-protein] hydrolase	3.1.2.14	C181ACP + H2O -> C181 + ACP	BLASTP
AO090124000084	Acyl-[acyl-carrier-protein] hydrolase	3.1.2.14	C182ACP + H2O -> C182 + ACP	BLASTP
AO090011000040	Acyl-[acyl-carrier-protein] hydrolase	3.1.2.14	C182ACP + H2O -> C182 + ACP	BLASTP
AO090124000084	Acyl-[acyl-carrier-protein] hydrolase	3.1.2.14	C183ACP + H2O -> C183 + ACP	BLASTP
AO090011000040	Acyl-[acyl-carrier-protein] hydrolase	3.1.2.14	C183ACP + H2O -> C183 + ACP	BLASTP
**# Fatty acid metabolism**				
AO090102000633	Long-chain-fatty-acid-CoA ligase	6.2.1.3	C40 + COA + ATP -> C40COA + H2O + PPI + AMP	BLASTP
AO090102000634	Long-chain-fatty-acid-CoA ligase	6.2.1.3	C40 + COA + ATP -> C40COA + H2O + PPI + AMP	BLASTP
AO090011000642	Long-chain-fatty-acid-CoA ligase	6.2.1.3	C40 + COA + ATP -> C40COA + H2O + PPI + AMP	BLASTP
AO090023000529	Long-chain-fatty-acid-CoA ligase	6.2.1.3	C40 + COA + ATP -> C40COA + H2O + PPI + AMP	BLASTP
AO090038000487	Long-chain-fatty-acid-CoA ligase	6.2.1.3	C40 + COA + ATP -> C40COA + H2O + PPI + AMP	BLASTP
AO090102000633	Long-chain-fatty-acid-CoA ligase	6.2.1.3	C60 + COA + ATP -> C60COA + H2O + PPI + AMP	BLASTP
AO090102000634	Long-chain-fatty-acid-CoA ligase	6.2.1.3	C60 + COA + ATP -> C60COA + H2O + PPI + AMP	BLASTP
AO090011000642	Long-chain-fatty-acid-CoA ligase	6.2.1.3	C60 + COA + ATP -> C60COA + H2O + PPI + AMP	BLASTP
AO090023000529	Long-chain-fatty-acid-CoA ligase	6.2.1.3	C60 + COA + ATP -> C60COA + H2O + PPI + AMP	BLASTP
AO090038000487	Long-chain-fatty-acid-CoA ligase	6.2.1.3	C60 + COA + ATP -> C60COA + H2O + PPI + AMP	BLASTP
AO090102000633	Long-chain-fatty-acid-CoA ligase	6.2.1.3	C80 + COA + ATP -> C80COA + H2O + PPI + AMP	BLASTP
AO090102000634	Long-chain-fatty-acid-CoA ligase	6.2.1.3	C80 + COA + ATP -> C80COA + H2O + PPI + AMP	BLASTP
AO090011000642	Long-chain-fatty-acid-CoA ligase	6.2.1.3	C80 + COA + ATP -> C80COA + H2O + PPI + AMP	BLASTP
AO090023000529	Long-chain-fatty-acid-CoA ligase	6.2.1.3	C80 + COA + ATP -> C80COA + H2O + PPI + AMP	BLASTP
AO090038000487	Long-chain-fatty-acid-CoA ligase	6.2.1.3	C80 + COA + ATP -> C80COA + H2O + PPI + AMP	BLASTP
AO090102000633	Long-chain-fatty-acid-CoA ligase	6.2.1.3	C100 + COA + ATP -> C100COA + H2O + PPI + AMP	BLASTP
AO090102000634	Long-chain-fatty-acid-CoA ligase	6.2.1.3	C100 + COA + ATP -> C100COA + H2O + PPI + AMP	BLASTP
AO090011000642	Long-chain-fatty-acid-CoA ligase	6.2.1.3	C100 + COA + ATP -> C100COA + H2O + PPI + AMP	BLASTP
AO090023000529	Long-chain-fatty-acid-CoA ligase	6.2.1.3	C100 + COA + ATP -> C100COA + H2O + PPI + AMP	BLASTP
AO090038000487	Long-chain-fatty-acid-CoA ligase	6.2.1.3	C100 + COA + ATP -> C100COA + H2O + PPI + AMP	BLASTP
AO090102000633	Long-chain-fatty-acid-CoA ligase	6.2.1.3	C120 + COA + ATP -> C120COA + H2O + PPI + AMP	BLASTP
AO090102000634	Long-chain-fatty-acid-CoA ligase	6.2.1.3	C120 + COA + ATP -> C120COA + H2O + PPI + AMP	BLASTP
AO090011000642	Long-chain-fatty-acid-CoA ligase	6.2.1.3	C120 + COA + ATP -> C120COA + H2O + PPI + AMP	BLASTP
AO090023000529	Long-chain-fatty-acid-CoA ligase	6.2.1.3	C120 + COA + ATP -> C120COA + H2O + PPI + AMP	BLASTP

Gene name	Enzyme name	EC number	Biochemical reaction	Annotation method
AO090038000487	Long-chain-fatty-acid-CoA ligase	6.2.1.3	C120 + COA + ATP -> C120COA + H2O + PPI + AMP	BLASTP
AO090102000633	Long-chain-fatty-acid-CoA ligase	6.2.1.3	C140 + COA + ATP -> C140COA + H2O + PPI + AMP	BLASTP
AO090102000634	Long-chain-fatty-acid-CoA ligase	6.2.1.3	C140 + COA + ATP -> C140COA + H2O + PPI + AMP	BLASTP
AO090011000642	Long-chain-fatty-acid-CoA ligase	6.2.1.3	C140 + COA + ATP -> C140COA + H2O + PPI + AMP	BLASTP
AO090023000529	Long-chain-fatty-acid-CoA ligase	6.2.1.3	C140 + COA + ATP -> C140COA + H2O + PPI + AMP	BLASTP
AO090038000487	Long-chain-fatty-acid-CoA ligase	6.2.1.3	C140 + COA + ATP -> C140COA + H2O + PPI + AMP	BLASTP
AO090102000633	Long-chain-fatty-acid-CoA ligase	6.2.1.3	C160 + COA + ATP -> C160COA + H2O + PPI + AMP	BLASTP
AO090102000634	Long-chain-fatty-acid-CoA ligase	6.2.1.3	C160 + COA + ATP -> C160COA + H2O + PPI + AMP	BLASTP
AO090011000642	Long-chain-fatty-acid-CoA ligase	6.2.1.3	C160 + COA + ATP -> C160COA + H2O + PPI + AMP	BLASTP
AO090023000529	Long-chain-fatty-acid-CoA ligase	6.2.1.3	C160 + COA + ATP -> C160COA + H2O + PPI + AMP	BLASTP
AO090038000487	Long-chain-fatty-acid-CoA ligase	6.2.1.3	C160 + COA + ATP -> C160COA + H2O + PPI + AMP	BLASTP
AO090102000633	Long-chain-fatty-acid-CoA ligase	6.2.1.3	C161 + COA + ATP -> C161COA + H2O + PPI + AMP	BLASTP
AO090102000634	Long-chain-fatty-acid-CoA ligase	6.2.1.3	C161 + COA + ATP -> C161COA + H2O + PPI + AMP	BLASTP
AO090011000642	Long-chain-fatty-acid-CoA ligase	6.2.1.3	C161 + COA + ATP -> C161COA + H2O + PPI + AMP	BLASTP
AO090023000529	Long-chain-fatty-acid-CoA ligase	6.2.1.3	C161 + COA + ATP -> C161COA + H2O + PPI + AMP	BLASTP
AO090038000487	Long-chain-fatty-acid-CoA ligase	6.2.1.3	C161 + COA + ATP -> C161COA + H2O + PPI + AMP	BLASTP
AO090102000633	Long-chain-fatty-acid-CoA ligase	6.2.1.3	C162 + COA + ATP -> C162COA + H2O + PPI + AMP	BLASTP
AO090102000634	Long-chain-fatty-acid-CoA ligase	6.2.1.3	C162 + COA + ATP -> C162COA + H2O + PPI + AMP	BLASTP
AO090011000642	Long-chain-fatty-acid-CoA ligase	6.2.1.3	C162 + COA + ATP -> C162COA + H2O + PPI + AMP	BLASTP
AO090023000529	Long-chain-fatty-acid-CoA ligase	6.2.1.3	C162 + COA + ATP -> C162COA + H2O + PPI + AMP	BLASTP
AO090038000487	Long-chain-fatty-acid-CoA ligase	6.2.1.3	C162 + COA + ATP -> C162COA + H2O + PPI + AMP	BLASTP
AO090102000633	Long-chain-fatty-acid-CoA ligase	6.2.1.3	C180 + COA + ATP -> C180COA + H2O + PPI + AMP	BLASTP
AO090102000634	Long-chain-fatty-acid-CoA ligase	6.2.1.3	C180 + COA + ATP -> C180COA + H2O + PPI + AMP	BLASTP
AO090011000642	Long-chain-fatty-acid-CoA ligase	6.2.1.3	C180 + COA + ATP -> C180COA + H2O + PPI + AMP	BLASTP
AO090023000529	Long-chain-fatty-acid-CoA ligase	6.2.1.3	C180 + COA + ATP -> C180COA + H2O + PPI + AMP	BLASTP
AO090038000487	Long-chain-fatty-acid-CoA ligase	6.2.1.3	C180 + COA + ATP -> C180COA + H2O + PPI + AMP	BLASTP
AO090102000633	Long-chain-fatty-acid-CoA ligase	6.2.1.3	C181 + COA + ATP -> C181COA + H2O + PPI + AMP	BLASTP
AO090102000634	Long-chain-fatty-acid-CoA ligase	6.2.1.3	C181 + COA + ATP -> C181COA + H2O + PPI + AMP	BLASTP
AO090011000642	Long-chain-fatty-acid-CoA ligase	6.2.1.3	C181 + COA + ATP -> C181COA + H2O + PPI + AMP	BLASTP
AO090023000529	Long-chain-fatty-acid-CoA ligase	6.2.1.3	C181 + COA + ATP -> C181COA + H2O + PPI + AMP	BLASTP
AO090038000487	Long-chain-fatty-acid-CoA ligase	6.2.1.3	C181 + COA + ATP -> C181COA + H2O + PPI + AMP	BLASTP

Gene name	Enzyme name	EC number	Biochemical reaction	Annotation method
AO090102000633	Long-chain-fatty-acid-CoA ligase	6.2.1.3	C182 + COA + ATP -> C182COA + H2O + PPI + AMP	BLASTP
AO090102000634	Long-chain-fatty-acid-CoA ligase	6.2.1.3	C182 + COA + ATP -> C182COA + H2O + PPI + AMP	BLASTP
AO090011000642	Long-chain-fatty-acid-CoA ligase	6.2.1.3	C182 + COA + ATP -> C182COA + H2O + PPI + AMP	BLASTP
AO090023000529	Long-chain-fatty-acid-CoA ligase	6.2.1.3	C182 + COA + ATP -> C182COA + H2O + PPI + AMP	BLASTP
AO090038000487	Long-chain-fatty-acid-CoA ligase	6.2.1.3	C182 + COA + ATP -> C182COA + H2O + PPI + AMP	BLASTP
AO090102000633	Long-chain-fatty-acid-CoA ligase	6.2.1.3	C183 + COA + ATP -> C183COA + H2O + PPI + AMP	BLASTP
AO090102000634	Long-chain-fatty-acid-CoA ligase	6.2.1.3	C183 + COA + ATP -> C183COA + H2O + PPI + AMP	BLASTP
AO090011000642	Long-chain-fatty-acid-CoA ligase	6.2.1.3	C183 + COA + ATP -> C183COA + H2O + PPI + AMP	BLASTP
AO090023000529	Long-chain-fatty-acid-CoA ligase	6.2.1.3	C183 + COA + ATP -> C183COA + H2O + PPI + AMP	BLASTP
AO090038000487	Long-chain-fatty-acid-CoA ligase	6.2.1.3	C183 + COA + ATP -> C183COA + H2O + PPI + AMP	BLASTP
AO090005000482	Very-long-chain acyl-CoA dehydrogenase	1.3.99.13	C180COAm + FADm -> C18DCOAm + FADH2m	BLASTP
AO090005000521/ AO090009000596/ AO090102000172/ AO090012000150/ AO090005000494/ AO090023000711/ ZY077810	Acyl-CoA dehydrogenase	1.3.99.3	C180COAm + FADm -> C18DCOAm + FADH2m	EST, BLASTP
AO090005000479	Acyl-coenzyme A oxidase	1.3.3.6	C180COAm + O2m -> C18DCOAm + H2O2m	GFAOP
AO090011000575	Enoyl-CoA hydratase	4.2.1.17	C18DCOAm + H2Om <-> C18HCOAm	GFAOP
AO090001000629/ AO090026000443/ AO090009000113/ AO090026000411/ AO090103000436	3-hydroxyacyl-CoA dehydrogenase	1.1.1.35	C18HCOAm + NADm <-> C18OCOAm + NADHm	BLASTP
AO090003001121/ AO090005000090/ AO090026000515/ AO090012000715	Acetyl-CoA C-acyltransferase	2.3.1.16	C18OCOAm + COAm <-> ACCOAm + C160COAm	BLASTP
AO090005000482	Very-long-chain acyl-CoA dehydrogenase	1.3.99.13	C160COAm + FADm -> C16DCOAm + FADH2m	BLASTP

Gene name	Enzyme name	EC number	Biochemical reaction	Annotation method
AO090005000521/ AO090009000596/ AO090102000172/ AO090012000150/ AO090005000494/ AO090023000711/ ZY077810	Acyl-CoA dehydrogenase	1.3.99.3	C160COAm + FADm -> C16DCOAm + FADH2m	EST, BLASTP
AO090005000479	Acyl-coenzyme A oxidase	1.3.3.6	C160COAm + O2m -> C16DCOAm + H2O2m	GFAOP
AO090011000575	Enoyl-CoA hydratase	4.2.1.17	C16DCOAm + H2Om <-> C16HCOAm	GFAOP
AO090001000629/ AO090026000443/ AO090009000113/ AO090026000411/ AO090103000436	3-hydroxyacyl-CoA dehydrogenase	1.1.1.35	C16HCOAm + NADm <-> C16OCOAm + NADHm	BLASTP
AO090003001121/ AO090005000090/ AO090026000515/ AO090012000715	Acetyl-CoA C-acyltransferase	2.3.1.16	C16OCOAm + COAm <-> ACCOAm + C140COAm	BLASTP
AO090005000482	Very-long-chain acyl-CoA dehydrogenase	1.3.99.13	C140COAm + FADm -> C14DCOAm + FADH2m	BLASTP
AO090005000521/ AO090009000596/ AO090102000172/ AO090012000150/ AO090005000494/ AO090023000711/ ZY077810	Acyl-CoA dehydrogenase	1.3.99.3	C140COAm + FADm -> C14DCOAm + FADH2m	EST, BLASTP
AO090005000479	Acyl-coenzyme A oxidase	1.3.3.6	C140COAm + O2m -> C14DCOAm + H2O2m	GFAOP
AO090011000575	Enoyl-CoA hydratase	4.2.1.17	C14DCOAm + H2Om <-> C14HCOAm	GFAOP
AO090001000629/ AO090026000443/ AO090009000113/ AO090026000411/ AO090103000436	3-hydroxyacyl-CoA dehydrogenase	1.1.1.35	C14HCOAm + NADm <-> C14OCOAm + NADHm	BLASTP

Gene name	Enzyme name	EC number	Biochemical reaction	Annotation method
AO090003001121/ AO090005000090/ AO090026000515/ AO090012000715	Acetyl-CoA C-acyltransferase	2.3.1.16	C14OCOAm + COAm <-> ACCOAm + C120COAm	BLASTP
AO090005000482	Very-long-chain acyl-CoA dehydrogenase	1.3.99.13	C120COAm + FADm -> C12DCOAm + FADH2m	BLASTP
AO090005000521/ AO090009000596/ AO090102000172/ AO090012000150/ AO090005000494/ AO090023000711/ ZY077810	Acyl-CoA dehydrogenase	1.3.99.3	C120COAm + FADm -> C12DCOAm + FADH2m	EST, BLASTP
AO090005000479	Acyl-coenzyme A oxidase	1.3.3.6	C120COAm + O2m -> C12DCOAm + H2O2m	GFAOP
AO090011000575	Enoyl-CoA hydratase	4.2.1.17	C12DCOAm + H2Om <-> C12HCOAm	GFAOP
AO090001000629/ AO090026000443/ AO090009000113/ AO090026000411/ AO090103000436	3-hydroxyacyl-CoA dehydrogenase	1.1.1.35	C12HCOAm + NADm <-> C12OCOAm + NADHm	BLASTP
AO090003001121/ AO090005000090/ AO090026000515/ AO090012000715	Acetyl-CoA C-acyltransferase	2.3.1.16	C12OCOAm + COAm <-> ACCOAm + C100COAm	BLASTP
AO090005000482	Very-long-chain acyl-CoA dehydrogenase	1.3.99.13	C100COAm + FADm -> C10DCOAm + FADH2m	BLASTP
AO090005000521/ AO090009000596/ AO090102000172/ AO090012000150/ AO090005000494/ AO090023000711/ ZY077810	Acyl-CoA dehydrogenase	1.3.99.3	C100COAm + FADm -> C10DCOAm + FADH2m	EST, BLASTP
AO090005000479	Acyl-coenzyme A oxidase	1.3.3.6	C100COAm + O2m -> C10DCOAm + H2O2m	GFAOP
AO090011000575	Enoyl-CoA hydratase	4.2.1.17	C10DCOAm + H2Om <-> C10HCOAm	GFAOP

Gene name	Enzyme name	EC number	Biochemical reaction	Annotation method
AO090001000629/ AO090026000443/ AO090009000113/ AO090026000411/ AO090103000436	3-hydroxyacyl-CoA dehydrogenase	1.1.1.35	C10HCOAm + NADm <-> C10OCOAm + NADHm	BLASTP
AO090003001121/ AO090005000090/ AO090026000515/ AO090012000715	Acetyl-CoA C-acyltransferase	2.3.1.16	C10OCOAm + COAm <-> ACCOAm + C80COAm	BLASTP
AO090005000482	Very-long-chain acyl-CoA dehydrogenase	1.3.99.13	C80COAm + FADm -> C8DCOAm + FADH2m	BLASTP
AO090005000521/ AO090009000596/ AO090102000172/ AO090012000150/ AO090005000494/ AO090023000711/ ZY077810	Acyl-CoA dehydrogenase	1.3.99.3	C80COAm + FADm -> C8DCOAm + FADH2m	EST, BLASTP
AO090005000479	Acyl-coenzyme A oxidase	1.3.3.6	C80COAm + O2m -> C8DCOAm + H2O2m	GFAOP
AO090011000575	Enoyl-CoA hydratase	4.2.1.17	C8DCOAm + H2Om <-> C8HCOAm	GFAOP
AO090001000629/ AO090026000443/ AO090009000113/ AO090026000411/ AO090103000436	3-hydroxyacyl-CoA dehydrogenase	1.1.1.35	C8HCOAm + NADm <-> C8OCOAm + NADHm	BLASTP
AO090003001121/ AO090005000090/ AO090026000515/ AO090012000715	Acetyl-CoA C-acyltransferase	2.3.1.16	C8OCOAm + COAm <-> ACCOAm + C60COAm	BLASTP
AO090103000106/ ZY077810	Acyl-CoA dehydrogenase	1.3.99.3	C60COAm + FADm -> C6DCOAm + FADH2m	EST, GFAOP
AO090005000479	Acyl-coenzyme A oxidase	1.3.3.6	C60COAm + O2m -> C6DCOAm + H2O2m	GFAOP
AO090011000575	Enoyl-CoA hydratase	4.2.1.17	C6DCOAm + H2Om <-> C6HCOAm	GFAOP

Gene name	Enzyme name	EC number	Biochemical reaction	Annotation method
AO090001000629/<br>AO090026000443/<br>AO090009000113/<br>AO090026000411/<br>AO090103000436	3-hydroxyacyl-CoA dehydrogenase	1.1.1.35	C6HCOAm + NADm <-> C6OCOAm + NADHm	BLASTP
AO090003001121/<br>AO090005000090/<br>AO090026000515/<br>AO090012000715	Acetyl-CoA C-acyltransferase	2.3.1.16	C6OCOAm + COAm <-> ACCOAm + C40COAm	BLASTP
AO090103000106/<br>ZY077810	Acyl-CoA dehydrogenase	1.3.99.3	C40COAm + FADm -> C4DCOAm + FADH2m	EST, GFAOP
AO090005000479	Acyl-coenzyme A oxidase	1.3.3.6	C40COAm + O2m -> C4DCOAm + H2O2m	GFAOP
AO090011000575	Enoyl-CoA hydratase	4.2.1.17	C4DCOAm + H2Om <-> C4HCOAm	GFAOP
AO090001000629/<br>AO090026000443/<br>AO090009000113/<br>AO090026000411/<br>AO090103000436	3-hydroxyacyl-CoA dehydrogenase	1.1.1.35	C4HCOAm + NADm <-> AACCOAm + NADHm	BLASTP
AO090003001121/<br>AO090005000090/<br>AO090026000515/<br>AO090012000715	Acetyl-CoA C-acyltransferase	2.3.1.16	AACCOAm + COAm <-> 2 ACCOAm	BLASTP
**# Glycerol metabolism**				
AO090120000396	Dihydroxyacetone kinase	2.7.1.29	GLYN + ATP -> T3P2 + ADP	BLASTP
AO090005000207	Glycerol dehydrogenase	1.1.1.6	GL + NAD -> GLYN + NADH	BLASTP
AO090010000381	Glycerol dehydrogenase	1.1.1.6	GL + NAD -> GLYN + NADH	BLASTP
AO090011000614	Glycerol dehydrogenase	1.1.1.6	GL + NAD -> GLYN + NADH	BLASTP
AO090038000442	Glycerol dehydrogenase	1.1.1.6	GL + NAD -> GLYN + NADH	BLASTP
AO090003001045	Glycerol dehydrogenase (NADP)	1.1.1.72	GLYAL + NADPH -> GL + NADP	BLASTP
AO090009000563	Glycerol dehydrogenase (NADP)	1.1.1.72	GLYAL + NADPH -> GL + NADP	BLASTP
AO090003001045	Glycerol dehydrogenase (NADP)	1.1.1.72	GLYN + NADPH -> GL + NADP	BLASTP
AO090009000563	Glycerol dehydrogenase (NADP)	1.1.1.72	GLYN + NADPH -> GL + NADP	BLASTP
AO090001000447	Alcohol oxidase	1.1.3.13	GL + O2 -> GLYAL + H2O2	GFAOP

Gene name	Enzyme name	EC number	Biochemical reaction	Annotation method
AO090001000230	Alcohol oxidase	1.1.3.13	GL + O2 -> GLYAL + H2O2	GFAOP
AO090001000447	Alcohol oxidase	1.1.3.13	GL + O2 -> GLYN + H2O2	GFAOP
AO090001000230	Alcohol oxidase	1.1.3.13	GL + O2 -> GLYN + H2O2	GFAOP
AO090005000125	NAD-dependent alcohol dehydrogenase	1.1.1.1	GL + NAD <-> GLYAL + NADH	BLASTP
AO090012000375	NAD-dependent alcohol dehydrogenase	1.1.1.1	GL + NAD <-> GLYAL + NADH	BLASTP
AO090009000634	NAD-dependent alcohol dehydrogenase	1.1.1.1	GL + NAD <-> GLYAL + NADH	BLASTP
AO090038000108	NAD-dependent alcohol dehydrogenase	1.1.1.1	GL + NAD <-> GLYAL + NADH	BLASTP
AO090003001407	NAD-dependent alcohol dehydrogenase	1.1.1.1	GL + NAD <-> GLYAL + NADH	BLASTP
AO090010000668	NADP-dependent alcohol dehydrogenase	1.1.1.2	GL + NADP <-> GLYAL + NADPH	BLASTP
AO090005001358	NADP-dependent alcohol dehydrogenase	1.1.1.2	GL + NADP <-> GLYAL + NADPH	BLASTP
AO090023000460	NADP-dependent alcohol dehydrogenase	1.1.1.2	GL + NADP <-> GLYAL + NADPH	BLASTP
AO090003000751	NADP-dependent alcohol dehydrogenase	1.1.1.2	GL + NADP <-> GLYAL + NADPH	BLASTP
AO090003001067	Glycerol kinase	2.7.1.30	GL + ATP -> GL3P + ADP	BLASTP
AO090001000509	Glycerol kinase	2.7.1.30	GL + ATP -> GL3P + ADP	BLASTP
AO090005001646	Glycerol-3-phosphate dehydrogenase	1.1.99.5	GL3P + FADm -> T3P2 + FADH2m	BLASTP
AO090005000038	Glycerol-3-phosphate dehydrogenase	1.1.99.5	GL3P + FADm -> T3P2 + FADH2m	BLASTP
AO090011000879	Glycerol 3-phosphate dehydrogenase	1.1.1.8	T3P2 + NADH -> GL3P + NAD	BLASTP
AO090005000883	Glycerol 3-phosphate dehydrogenase	1.1.1.8	T3P2 + NADH -> GL3P + NAD	BLASTP
AO090038000367	Glycerol 3-phosphatase	3.1.3.21	GL3P + H2O -> GL + PI	BLASTP
**# Sterol metabolism**				
AO090010000204	Squalene synthase	2.5.1.21	2 FPP + NADPH -> NADP + SQL + 2 PI	BLASTP
AO090701000685	Squalene monooxygenase	1.14.99.7	SQL + O2 + NADP -> S23E + NADPH	BLASTP
**# Ergosterol esters metabolism**				
AO090102000611	Oxidosqualene:lanosterol cyclase	5.4.99.7	S23E -> LNST	BLASTP
AO090020000194	Oxidosqualene:lanosterol cyclase	5.4.99.7	S23E -> LNST	BLASTP
AO090166000068	Oxidosqualene:lanosterol cyclase	5.4.99.7	S23E -> LNST	BLASTP
AO090003000205	Sterol 14-demethylase	1.14.13.70	LNST + FADH2m + O2 -> IGST + FADm	BLASTP
AO090020000357	Sterol 14-demethylase	1.14.13.70	LNST + FADH2m + O2 -> IGST + FADm	BLASTP
AO090026000842	Sterol 14-demethylase	1.14.13.70	LNST + FADH2m + O2 -> IGST + FADm	BLASTP

Gene name	Enzyme name	EC number	Biochemical reaction	Annotation method
AO090009000362	C-14 sterol reductase	1.3.1.70	IGST + NADPH -> DMZYMST + NADP	BLASTP
AO090023000728	C-14 sterol reductase	1.3.1.70	IGST + NADPH -> DMZYMST + NADP	BLASTP
AO090005000286	C-4 methyl sterol oxidase	1.14.13.72	3 O2 + DMZYMST -> IMZYMST	BLASTP
AO090010000667	C-4 methyl sterol oxidase	1.14.13.72	3 O2 + DMZYMST -> IMZYMST	BLASTP
AO090206000001	C-4 methyl sterol oxidase	1.14.13.72	3 O2 + DMZYMST -> IMZYMST	BLASTP
AO090120000187	C-3 sterol dehydrogenase	1.1.1.170	IMZYMST -> IIMZYMST + CO2	BLASTP
AO090003001070	C-3 sterol keto reductase	1.1.1.270	IIMZYMST + NADPH -> MZYMST + NADP	BLASTP
AO090005000286	C-4 sterol methyl oxidase	1.14.13.72	3 O2 + MZYMST -> IZYMST	BLASTP
AO090010000667	C-4 sterol methyl oxidase	1.14.13.72	3 O2 + MZYMST -> IZYMST	BLASTP
AO090206000001	C-4 sterol methyl oxidase	1.14.13.72	3 O2 + MZYMST -> IZYMST	BLASTP
AO090120000187	C-3 sterol dehydrogenase	1.1.1.170	IZYMST -> IIZYMST + CO2	BLASTP
AO090003001070	3-ketosteroid reductase	1.1.1.270	IIZYMST + NADPH -> ZYMST + NADP	BLASTP
AO090011000289	Sterol 24-c-methyltransferase	2.1.1.41	ZYMST + SAM -> FEST + SAH	BLASTP
AO090012000932	Sterol 24-c-methyltransferase	2.1.1.41	ZYMST + SAM -> FEST + SAH	BLASTP
AO090003000904	C-8 sterol isomerase	5.-.-.-	FEST -> EPST	BLASTP
AO090005000286	C-4 methyl sterol oxidase	1.14.13.72	EPST + O2 + NADPH -> NADP + ERTROL	BLASTP
AO090010000667	C-4 methyl sterol oxidase	1.14.13.72	EPST + O2 + NADPH -> NADP + ERTROL	BLASTP
AO090206000001	C-4 methyl sterol oxidase	1.14.13.72	EPST + O2 + NADPH -> NADP + ERTROL	BLASTP
AO090003000954	Sterol C-22 desaturase	1.14.14.-	ERTROL + O2 + NADPH -> NADP + ERTEOL	BLASTP
AO090012000966	c-24(28) sterol reductase	1.3.1.71	ERTEOL + NADPH -> ERGOST + NADP	BLASTP
AO090026000130	c-24(28) sterol reductase	1.3.1.71	ERTEOL + NADPH -> ERGOST + NADP	BLASTP
AO090005000559	c-24(28) sterol reductase	1.3.1.71	ERTEOL + NADPH -> ERGOST + NADP	BLASTP
GAP	Diacylglycerol—sterol O-acyltransferase	2.3.1.73	DAGLY + ERGOST -> MAGLY + ERGOSE	NONE
AO090020000490	Triglyceride lipase-cholesterol esterase	3.1.1.13	ERGOSE + H2O -> ERGOST + 0.2720 C160 + 0.0051 C161 + 0.0304 C180 + 0.1534 C181 + 0.5573 C182 + 0.0404 C183	BLASTP
GAP	Acylglycerol lipase	3.1.1.23	MAGLY + H2O -> GL + 0.2720 C160 + 0.0051 C161 + 0.0304 C180 + 0.1534 C181 + 0.5573 C182 + 0.0404 C183	NONE

Gene name	Enzyme name	EC number	Biochemical reaction	Annotation method
**# Mevalonate pathway**				
AO090103000406	Acetyl-CoA-acetyltransferase	2.3.1.9	2 ACCOAm <-> COAm + AACCOAm	BLASTP
AO090103000012	Acetyl-CoA-acetyltransferase	2.3.1.9	2 ACCOAp <-> COAp + AACCOAp	BLASTP
AO090023000421	Acetyl-CoA-acetyltransferase	2.3.1.9	2 ACCOA <-> COA + AACCOA	BLASTP
AO090003000611	Hydroxymethylglutaryl-CoA synthase	2.3.3.10	H3MCOA + COA <-> ACCOA + AACCOA	BLASTP
AO090010000487	Hydroxymethylglutaryl-CoA synthase	2.3.3.10	H3MCOA + COA <-> ACCOA + AACCOA	BLASTP
AO090103000311	HMG-CoA reductase	1.1.1.34	MVL + COA + 2 NADP <-> H3MCOA + 2 NADPH	BLASTP
AO090120000217	HMG-CoA reductase	1.1.1.34	MVL + COA + 2 NADP <-> H3MCOA + 2 NADPH	BLASTP
AO090023000793	Mevalonate kinase	2.7.1.36	ATP + MVL -> ADP + PMVL	BLASTP
AO090010000471	Phosphomevalonate kinase	2.7.4.2	ATP + PMVL -> ADP + PPMVL	BLASTP
AO090023000862	Diphosphomevalonate decarboxylase	4.1.1.33	ATP + PPMVL -> ADP + PI + IPPP + CO2	BLASTP
AO090023000500	Isopentenyl-diphosphate delta-isomerase	5.3.3.2	IPPP <-> DMPP	BLASTP
AO090023000391	Isopentenyl-diphosphate delta-isomerase	5.3.3.2	IPPP <-> DMPP	BLASTP
AO090102000358	Farnesyl-pyrophosphate synthetase	2.5.1.1	DMPP + IPPP -> GPP + PPI	BLASTP
AO090102000358	Geranyltranstransferase	2.5.1.10	GPP + IPPP -> FPP + PPI	BLASTP
AO090001000268	Geranylgeranylpyrophosphate synthetase	2.5.1.29	FPP + IPPP -> PPI + GGPP	BLASTP
AO090012000573	Geranylgeranylpyrophosphate synthetase	2.5.1.29	FPP + IPPP -> PPI + GGPP	BLASTP
AO090023000070	Geranylgeranylpyrophosphate synthetase	2.5.1.29	FPP + IPPP -> PPI + GGPP	BLASTP
AO090038000495	Geranylgeranylpyrophosphate synthetase	2.5.1.29	FPP + IPPP -> PPI + GGPP	BLASTP
AO090113000170	Geranylgeranylpyrophosphate synthetase	2.5.1.29	FPP + IPPP -> PPI + GGPP	BLASTP
AO090120000064	Geranylgeranylpyrophosphate synthetase	2.5.1.29	FPP + IPPP -> PPI + GGPP	BLASTP
AO090701000221	Geranylgeranylpyrophosphate synthetase	2.5.1.29	FPP + IPPP -> PPI + GGPP	BLASTP
AO090009000093	Geranylgeranylpyrophosphate synthetase	2.5.1.29	FPP + IPPP -> PPI + GGPP	BLASTP
AO090012000686	Geranylgeranylpyrophosphate synthetase	2.5.1.29	FPP + IPPP -> PPI + GGPP	BLASTP
ZY088142	Di-trans,poly-cis-decaprenylcistransferase	2.5.1.31	FPP + IPPP -> PPI + TTGGP	EST
**# Cholesterol Metabolism**				
AO090005001216	Cholestenol delta-isomerase	5.3.3.5	ZYMST -> CHORESTA	BLASTP
AO090026000084	Cholestenol delta-isomerase	5.3.3.5	ZYMST -> CHORESTA	BLASTP
ZY097910	Cholestenol delta-isomerase	5.3.3.5	ZYMST -> CHORESTA	EST
GAP	Delta-24-sterol reductase	1.3.1.72	CHORESTA + NADPH -> LTST + NADP	NONE

Gene name	Enzyme name	EC number	Biochemical reaction	Annotation method
AO090005001107	Sterol delta 5,6-desaturase	1.3.3.2	LTST + NADH + O2 -> 7DECHORES + NAD + H2O	BLASTP
AO090103000138	Sterol delta 5,6-desaturase	1.3.3.2	LTST + NADH + O2 -> 7DECHORES + NAD + H2O	BLASTP
AO090701000022	Sterol delta 5,6-desaturase	1.3.3.2	LTST + NADH + O2 -> 7DECHORES + NAD + H2O	BLASTP
AO090003000792	7-dehydrocholesterol reductase	1.3.1.21	7DECHORES + NADPH -> CHOREOL + NADP	GFAOP
AO090009000480	Sterol o-acyltransferase	2.3.1.26	ACOA + CHOREOL -> COA + CHOREOLESTR	BLASTP
**# Phospholipid metabolism**				
AO090003001084	Glycerol-3-phosphate acyltransferase	2.3.1.15	GL3P + 0.5856 C160ACP + 0.0144 C161ACP + 0.0543 C180ACP + 0.4261 C181ACP + 1.9754 C182ACP + 0.0744 C183ACP -> AGL3P + ACP	BLASTP
AO090701000835	Glycerol-3-phosphate acyltransferase	2.3.1.15	GL3P + 0.5856 C160ACP + 0.0144 C161ACP + 0.0543 C180ACP + 0.4261 C181ACP + 1.9754 C182ACP + 0.0744 C183ACP -> AGL3P + ACP	BLASTP
AO090003001084	Glycerol-3-phosphate acyltransferase	2.3.1.15	T3P2 + 0.5856 C160ACP + 0.0144 C161ACP + 0.0543 C180ACP + 0.4261 C181ACP + 1.9754 C182ACP + 0.0744 C183ACP -> AT3P2 + ACP	BLASTP
AO090701000835	Glycerol-3-phosphate acyltransferase	2.3.1.15	T3P2 + 0.5856 C160ACP + 0.0144 C161ACP + 0.0543 C180ACP + 0.4261 C181ACP + 1.9754 C182ACP + 0.0744 C183ACP -> AT3P2 + ACP	BLASTP
AO090023000308	Diacylglycerol pyrophosphate phosphatase	3.1.3.-/3.1.3.4	PA -> DAGLY + PI	BLASTP
AO090012000871	Diacylglycerol pyrophosphate phosphatase	3.1.3.-/3.1.3.4	PA -> DAGLY + PI	BLASTP
AO090103000232	Diacylglycerol pyrophosphate phosphatase	3.1.3.-/3.1.3.4	PA -> DAGLY + PI	BLASTP
AO090023000635	Diacylglycerol pyrophosphate phosphatase	3.1.3.-/3.1.3.4	PA -> DAGLY + PI	BLASTP
AO090023000308	Diacylglycerol pyrophosphate phosphatase	3.1.3.-/3.1.3.4	DGPP -> PA + PI	BLASTP
AO090023000635	Diacylglycerol pyrophosphate phosphatase	3.1.3.-/3.1.3.4	DGPP -> PA + PI	BLASTP

Gene name	Enzyme name	EC number	Biochemical reaction	Annotation method
GAP	Phosphatidylglycerol phosphate phosphatase	3.1.3.27	PGPm -> PIm + PGm	NONE
AO090701000187	CDP-diacylglycerol-glycerol-3-phosphate 3-phosphatidyltransferase	2.7.8.5	CDPDGm + GL3Pm -> CMPm + CLm	BLASTP
ZY135516	CDP-diacylglycerol-glycerol-3-phosphate 3-phosphatidyltransferase	2.7.8.5	CDPDGm + GL3Pm -> CMPm + CLm	EST
AO090020000334	Ethanolaminephosphotransferase.	2.7.8.1	CDPETN + DAGLY <-> CMP + PE	BLASTP
AO090020000334	Diacylglycerol cholinephosphotransferase	2.7.8.2	CDPCHO + DAGLY -> CMP + PC	BLASTP
AO090005000648	Diacylglycerol cholinephosphotransferase	2.7.8.2	CDPCHO + DAGLY -> CMP + PC	BLASTP
AO090011000843	1-acylglycerol-3-phosphate acyltransferase	2.3.1.51	AGL3P + 0.5856 C160ACP + 0.0144 C161ACP + 0.0543 C180ACP + 0.4261 C181ACP + 1.9754 C182ACP + 0.0744 C183ACP -> PA + ACP	BLASTP
AO090012000953	Phosphatidate cytidylyltransferase	2.7.7.41	PA + CTP <-> CDPDG + PPI	BLASTP
AO090023000648	Phosphatidate cytidylyltransferase	2.7.7.41	PA + CTP <-> CDPDG + PPI	BLASTP
AO090012000953	Phosphatidate cytidylyltransferase	2.7.7.41	PAm + CTPm <-> CDPDGm + PPIm	BLASTP
AO090023000648	Phosphatidate cytidylyltransferase	2.7.7.41	PAm + CTPm <-> CDPDGm + PPIm	BLASTP
AO090009000203	Phosphatidylserine synthase	2.7.8.8	CDPDG + SER <-> CMP + PS	BLASTP
AO090009000203	Phosphatidylserine synthase	2.7.8.8	CDPDGm + SERm <-> CMPm + PSm	BLASTP
AO090003001013	Phosphatidylinositol synthase	2.7.8.11	CDPDG + MYOI -> CMP + PINS	BLASTP
AO090005001121	Phosphatidylinositol synthase	2.7.8.11	CDPDG + MYOI -> CMP + PINS	BLASTP
AO090005001124	Phosphatidylserine decarboxylase	4.1.1.65	PS -> PE + CO2	BLASTP
AO090012000733	Phosphatidylserine decarboxylase	4.1.1.65	PS -> PE + CO2	BLASTP
AO090005001124	Phosphatidylserine decarboxylase	4.1.1.65	PSm -> PEm + CO2m	BLASTP
AO090012000733	Phosphatidylserine decarboxylase	4.1.1.65	PSm -> PEm + CO2m	BLASTP
ZY110723	Phosphatidylserine decarboxylase	4.1.1.65	PS -> PE + CO2	EST
AO090012000690	Acylglycerone-phosphate reductase	1.1.1.101	AT3P2 + NADPH -> AGL3P + NADP	BLASTP
AO090001000150	Acylglycerone-phosphate reductase	1.1.1.101	AT3P2 + NADPH -> AGL3P + NADP	BLASTP
AO090011000608	Phosphatidylinositol 3-kinase	2.7.1.137	ATP + PINS -> ADP + PINSP	GFAOP
AO090120000393	Phosphatidylinositol 3-kinase	2.7.1.137	ATP + PINS -> ADP + PINSP	GFAOP
AO090206000006	Phosphatidylinositol 3-kinase	2.7.1.137	ATP + PINS -> ADP + PINSP	GFAOP
AO090020000426	Phosphatidylinositol 3-kinase	2.7.1.137	ATP + PINS -> ADP + PINSP	GFAOP

Gene name	Enzyme name	EC number	Biochemical reaction	Annotation method
ZY006430	Phosphatidylinositol-3,4,5-trisphosphate 3-phosphatase	3.1.3.67	PINSP + H2O -> D45PI + PI	EST
AO090026000314	Phosphatidylinositol 4-kinase	2.7.1.67	ATP + PINS -> ADP + PINS4P	BLASTP
AO090026000840	Phosphatidylinositol 4-kinase	2.7.1.67	ATP + PINS -> ADP + PINS4P	BLASTP
AO090020000081	Phosphatidylinositol-4-phosphate 5-kinase	2.7.1.68	PINS4P + ATP -> D45PI + ADP	BLASTP
AO090012000557	Phosphoinositide phospholipase C	3.1.4.11	D45PI -> TPI + DAGLY	BLASTP
AO090005001493	Phosphoinositide phospholipase C	3.1.4.11	D45PI -> TPI + DAGLY	BLASTP
AO090005001492	Phosphoinositide phospholipase C	3.1.4.11	D45PI -> TPI + DAGLY	BLASTP
AO090003000530	Phosphoinositide phospholipase C	3.1.4.11	D45PI -> TPI + DAGLY	BLASTP
AO090012000277	Phospholipase A	3.1.1.4/3.1.1.32	PC -> LPC + 0.5856 C160ACP + 0.0144 C161ACP + 0.0543 C180ACP + 0.4261 C181ACP + 1.9754 C182ACP + 0.0744 C183ACP	BLASTP
AO090012000277	Phospholipase A	3.1.1.4/3.1.1.32	PE -> LPE + 0.5856 C160ACP + 0.0144 C161ACP + 0.0543 C180ACP + 0.4261 C181ACP + 1.9754 C182ACP + 0.0744 C183ACP	BLASTP
AO090005000433	Phospholipase D	3.1.4.4	PC + H2O -> CHO + PA	BLASTP
AO090102000204	Phospholipase D	3.1.4.4	PC + H2O -> CHO + PA	BLASTP
AO090020000034	Phospholipase D	3.1.4.4	PC + H2O -> CHO + PA	BLASTP
ZY111104	Phospholipase D	3.1.4.4	PC + H2O -> CHO + PA	EST
AO090012000680	Lysophospholipase	3.1.1.5	LPC + H2O -> GLYCEROCHO + CARBO	BLASTP
AO090023000685	Lysophospholipase	3.1.1.5	LPC + H2O -> GLYCEROCHO + CARBO	BLASTP
AO090120000172	Lysophospholipase	3.1.1.5	LPC + H2O -> GLYCEROCHO + CARBO	BLASTP
AO090701000473	Lysophospholipase	3.1.1.5	LPC + H2O -> GLYCEROCHO + CARBO	BLASTP
AO090701000473	Lysophospholipase	3.1.1.5	LPC + H2O -> GLYCEROCHO + CARBO	BLASTP
AO090012000680	Lysophospholipase	3.1.1.5	LPC + H2O -> GLYCEROCHO + CARBO	BLASTP
AO090023000685	Lysophospholipase	3.1.1.5	LPC + H2O -> GLYCEROCHO + CARBO	BLASTP
AO090102000492	Lysophospholipase	3.1.1.5	LPC + H2O -> GLYCEROCHO + CARBO	BLASTP
AO090012000204	Phosphatidylethanolamine N-methyltransferase	2.1.1.17	SAM + PE -> SAH + PMME	BLASTP
AO090005001620	Phospholipid methyltransferase	2.1.1.71	SAM + PMME -> SAH + PDME	BLASTP
AO090005001620	Phospholipid methyltransferase	2.1.1.71	PDME + SAM -> PC + SAH	BLASTP

Gene name	Enzyme name	EC number	Biochemical reaction	Annotation method
No_Gene	Chemical conversion	No_EC	PA + NH4OH -> PAA + H2O	BLASTP
**# Ethanolamine, choline, phosphocholine metabolism**				
GAP	Serine decarboxylase	4.1.1.-	SER -> ETHM + CO2	NONE
AO090038000268	Ethanolamine kinase	2.7.1.82	ATP + ETHM -> ADP + PETHM	BLASTP
AO090701000122	Phosphoethanolamine N-methyltransferase	2.1.1.103	PETHM + 3 SAM -> PCHO + 3 SAH	BLASTP
AO090005001098	Choline kinase	2.7.1.32	ATP + CHO -> ADP + PCHO	BLASTP
ZY029856	Choline kinase	2.7.1.32	ATP + CHO -> ADP + PCHO	EST
AO090005001594	Cholinephosphate cytidylyltransferase	2.7.7.15	PCHO + CTP -> CDPCHO + PPI	BLASTP
AO090023001003	Phosphoethanolamine transferase	2.7.7.14	PETHM + CTP -> CDPETN + PPI	BLASTP
**# Sphingoglycolipid metabolism**				
AO090001000375	Serine palmitoyltransferase	2.3.1.50	C160COA + SER -> COA + DHSPH + CO2	BLASTP
AO090009000484	Serine palmitoyltransferase	2.3.1.50	C160COA + SER -> COA + DHSPH + CO2	BLASTP
AO090038000292	3-ketosphinganine reductase	1.1.1.102	DHSPH + NADPH -> SPH + NADP	BLASTP
AO090038000306	Sphinganine kinase	2.7.1.91	SPH + ATP -> DHSP + ADP	BLASTP
AO090038000306	Sphinganine kinase	2.7.1.91	PSPH + ATP -> PHSP + ADP	BLASTP
AO090003001164	Sphingosine-1-phosphate lyase	4.1.2.27	DHSP -> PETHM + C16A	BLASTP
AO090010000721	Sphingosine-1-phosphate lyase	4.1.2.27	DHSP -> PETHM + C16A	BLASTP
AO090003001164	Sphingosine-1-phosphate lyase	4.1.2.27	PHSP -> PETHM + C16A	BLASTP
AO090010000721	Sphingosine-1-phosphate lyase	4.1.2.27	PHSP -> PETHM + C16A	BLASTP
AO090005000995	Sphingolipid hydroxylase	No_EC	SPH + O2 + NADPH -> PSPH + NADP	BLASTP
AO090023000242	Ceramide synthase	2.3.1.24	PSPH + ACOA -> CER2 + COA	BLASTP
AO090005001117	Ceramide hydroxylase	No_EC	CER2 + NADPH + O2 -> CER3 + NADP	BLASTP
AO090023000893	Fatty acid desaturase	1.14.-.-	CER2 -> CER1	BLASTP
AO090003000518	MIP2C synthase	2.4.1.-	CER3 + PINS -> IPC	BLASTP
AO090701000073	MIPC synthase	2.4.1.-	IPC + GDPMAN -> MIPC	BLASTP
AO090005001189	MIPC synthase	2.4.1.-	IPC + GDPMAN -> MIPC	BLASTP
AO090003000518	MIP2C synthase	2.4.1.-	MIPC + PINS -> MIP2C	BLASTP
AO090113000120	Sphingosine-1-phosphate phosphatase	3.1.3.-	DHSP -> SPH + PI	BLASTP
AO090020000582	Ceramide glucosyltransferase	2.4.1.80	UDPG + CER1 -> UDP + GCYLCR	BLASTP
AO090003000091	Sphingomyelin phosphodiesterase	3.1.4.12	SPMYLIN + H2O -> CER1 + PCHO	BLASTP
AO090701000789	Sphingomyelin phosphodiesterase	3.1.4.12	SPMYLIN + H2O -> CER1 + PCHO	BLASTP

Gene name	Enzyme name	EC number	Biochemical reaction	Annotation method
AO090023000919	Sphingomyelin phosphodiesterase	3.1.4.12	SPMYLIN + H2O -> CER1 + PCHO	BLASTP
AO090120000416	Arylsulfatase	3.1.6.1	SUFT + H2O -> GACER + SLF	BLASTP
**# Complex lipid metabolism**				
**# Glycerolipid Metabolism**				
AO090011000863	Diacylglycerol O-acyltransferase	2.3.1.20	DAGLY + 0.4710 C160ACP + 0.0242 C161ACP + 0.1535 C180ACP + 0.5328 C181ACP + 2.3362 C182ACP + 0.1499 C183ACP -> TAGLY + ACP	BLASTP
AO090011000353	Triacylglycerol lipase	3.1.1.3	TAGLY + H2O -> DAGLY + 0.4710 C160ACP + 0.0242 C161ACP + 0.1535 C180ACP + 0.5328 C181ACP + 2.3362 C182ACP + 0.1499 C183ACP	BLASTP
AO090701000542	Triacylglycerol lipase	3.1.1.3	TAGLY + H2O -> DAGLY + 0.4710 C160ACP + 0.0242 C161ACP + 0.1535 C180ACP + 0.5328 C181ACP + 2.3362 C182ACP + 0.1499 C183ACP	BLASTP
AO090012000766	Triacylglycerol lipase	3.1.1.3	TAGLY + H2O -> DAGLY + 0.4710 C160ACP + 0.0242 C161ACP + 0.1535 C180ACP + 0.5328 C181ACP + 2.3362 C182ACP + 0.1499 C183ACP	BLASTP
AO090102000588	Triacylglycerol lipase	3.1.1.3	TAGLY + H2O -> DAGLY + 0.4710 C160ACP + 0.0242 C161ACP + 0.1535 C180ACP + 0.5328 C181ACP + 2.3362 C182ACP + 0.1499 C183ACP	BLASTP
AO090005000837	Triacylglycerol lipase	3.1.1.3	TAGLY + H2O -> DAGLY + 0.4710 C160ACP + 0.0242 C161ACP + 0.1535 C180ACP + 0.5328 C181ACP + 2.3362 C182ACP + 0.1499 C183ACP	BLASTP
AO090026000395	Triacylglycerol lipase	3.1.1.3	TAGLY + H2O -> DAGLY + 0.4710 C160ACP + 0.0242 C161ACP + 0.1535 C180ACP + 0.5328 C181ACP + 2.3362 C182ACP + 0.1499 C183ACP	BLASTP

Gene name	Enzyme name	EC number	Biochemical reaction	Annotation method
AO090120000164	Triacylglycerol lipase	3.1.1.3	TAGLY + H2O -> DAGLY + 0.4710 C160ACP + 0.0242 C161ACP + 0.1535 C180ACP + 0.5328 C181ACP + 2.3362 C182ACP + 0.1499 C183ACP	BLASTP
AO090003001432	Triacylglycerol lipase	3.1.1.3	TAGLY + H2O -> DAGLY + 0.4710 C160ACP + 0.0242 C161ACP + 0.1535 C180ACP + 0.5328 C181ACP + 2.3362 C182ACP + 0.1499 C183ACP	BLASTP
AO090103000036	Triacylglycerol lipase	3.1.1.3	TAGLY + H2O -> DAGLY + 0.4710 C160ACP + 0.0242 C161ACP + 0.1535 C180ACP + 0.5328 C181ACP + 2.3362 C182ACP + 0.1499 C183ACP	BLASTP
AO090001000143	Triacylglycerol lipase	3.1.1.3	TAGLY + H2O -> DAGLY + 0.4710 C160ACP + 0.0242 C161ACP + 0.1535 C180ACP + 0.5328 C181ACP + 2.3362 C182ACP + 0.1499 C183ACP	BLASTP
AO090701000644	Triacylglycerol lipase	3.1.1.3	TAGLY + H2O -> DAGLY + 0.4710 C160ACP + 0.0242 C161ACP + 0.1535 C180ACP + 0.5328 C181ACP + 2.3362 C182ACP + 0.1499 C183ACP	BLASTP
ZY080376	Triacylglycerol lipase	3.1.1.3	TAGLY + H2O -> DAGLY + 0.4710 C160ACP + 0.0242 C161ACP + 0.1535 C180ACP + 0.5328 C181ACP + 2.3362 C182ACP + 0.1499 C183ACP	EST
ZY111292	Triacylglycerol lipase	3.1.1.3	TAGLY + H2O -> DAGLY + 0.4710 C160ACP + 0.0242 C161ACP + 0.1535 C180ACP + 0.5328 C181ACP + 2.3362 C182ACP + 0.1499 C183ACP	EST
AO090011000353	Triacylglycerol lipase	3.1.1.3	DAGLY + H2O -> MAGLY + 0.4710 C160ACP + 0.0242 C161ACP + 0.1535 C180ACP + 0.5328 C181ACP + 2.3362 C182ACP + 0.1499 C183ACP	BLASTP

Gene name	Enzyme name	EC number	Biochemical reaction	Annotation method
AO090701000542	Triacylglycerol lipase	3.1.1.3	DAGLY + H2O -> MAGLY + 0.4710 C160ACP + 0.0242 C161ACP + 0.1535 C180ACP + 0.5328 C181ACP + 2.3362 C182ACP + 0.1499 C183ACP	BLASTP
AO090012000766	Triacylglycerol lipase	3.1.1.3	DAGLY + H2O -> MAGLY + 0.4710 C160ACP + 0.0242 C161ACP + 0.1535 C180ACP + 0.5328 C181ACP + 2.3362 C182ACP + 0.1499 C183ACP	BLASTP
AO090102000588	Triacylglycerol lipase	3.1.1.3	DAGLY + H2O -> MAGLY + 0.4710 C160ACP + 0.0242 C161ACP + 0.1535 C180ACP + 0.5328 C181ACP + 2.3362 C182ACP + 0.1499 C183ACP	BLASTP
AO090005000837	Triacylglycerol lipase	3.1.1.3	DAGLY + H2O -> MAGLY + 0.4710 C160ACP + 0.0242 C161ACP + 0.1535 C180ACP + 0.5328 C181ACP + 2.3362 C182ACP + 0.1499 C183ACP	BLASTP
AO090026000395	Triacylglycerol lipase	3.1.1.3	DAGLY + H2O -> MAGLY + 0.4710 C160ACP + 0.0242 C161ACP + 0.1535 C180ACP + 0.5328 C181ACP + 2.3362 C182ACP + 0.1499 C183ACP	BLASTP
AO090120000164	Triacylglycerol lipase	3.1.1.3	DAGLY + H2O -> MAGLY + 0.4710 C160ACP + 0.0242 C161ACP + 0.1535 C180ACP + 0.5328 C181ACP + 2.3362 C182ACP + 0.1499 C183ACP	BLASTP
AO090003001432	Triacylglycerol lipase	3.1.1.3	DAGLY + H2O -> MAGLY + 0.4710 C160ACP + 0.0242 C161ACP + 0.1535 C180ACP + 0.5328 C181ACP + 2.3362 C182ACP + 0.1499 C183ACP	BLASTP
AO090103000036	Triacylglycerol lipase	3.1.1.3	DAGLY + H2O -> MAGLY + 0.4710 C160ACP + 0.0242 C161ACP + 0.1535 C180ACP + 0.5328 C181ACP + 2.3362 C182ACP + 0.1499 C183ACP	BLASTP

Gene name	Enzyme name	EC number	Biochemical reaction	Annotation method
AO090001000143	Triacylglycerol lipase	3.1.1.3	DAGLY + H2O -> MAGLY + 0.4710 C160ACP + 0.0242 C161ACP + 0.1535 C180ACP + 0.5328 C181ACP + 2.3362 C182ACP + 0.1499 C183ACP	BLASTP
AO090701000644	Triacylglycerol lipase	3.1.1.3	DAGLY + H2O -> MAGLY + 0.4710 C160ACP + 0.0242 C161ACP + 0.1535 C180ACP + 0.5328 C181ACP + 2.3362 C182ACP + 0.1499 C183ACP	BLASTP
ZY080376	Triacylglycerol lipase	3.1.1.3	DAGLY + H2O -> MAGLY + 0.4710 C160ACP + 0.0242 C161ACP + 0.1535 C180ACP + 0.5328 C181ACP + 2.3362 C182ACP + 0.1499 C183ACP	EST
ZY111292	Triacylglycerol lipase	3.1.1.3	DAGLY + H2O -> MAGLY + 0.4710 C160ACP + 0.0242 C161ACP + 0.1535 C180ACP + 0.5328 C181ACP + 2.3362 C182ACP + 0.1499 C183ACP	EST
GAP	UDPgalactose:1,2-diacylglycerol 3-beta-D-galactosyltransferase	2.4.1.46	UDPGAL + DAGLY <-> UDP + MGDG	NONE
GAP	Galactolipid galactosyltransferase	2.4.1.184	UDPGAL + MGDG <-> UDP + DGDG	NONE
AO090003000514	Phospholipid:diacylglycerol acyltransferase	2.3.1.158	PC + DAGLY -> LPC + TAGLY	BLASTP
AO090003000514	Phospholipid:diacylglycerol acyltransferase	2.3.1.158	PE + DAGLY -> LPE + TAGLY	BLASTP
AO090003000514	Phospholipid:diacylglycerol acyltransferase	2.3.1.158	PAA + DAGLY -> LPAA + TAGLY	BLASTP
AO090003000514	Phospholipid:diacylglycerol acyltransferase	2.3.1.158	PS + DAGLY -> LPS + TAGLY	BLASTP
AO090003000514	Phospholipid:diacylglycerol acyltransferase	2.3.1.158	PDME + DAGLY -> LPDME + TAGLY	BLASTP
AO090003000514	Phospholipid:diacylglycerol acyltransferase	2.3.1.158	PMME + DAGLY -> LPMME + TAGLY	BLASTP
AO090003000514	Phospholipid:diacylglycerol acyltransferase	2.3.1.158	PG + DAGLY -> LPG + TAGLY	BLASTP
AO090005001097	phospholipase C	3.1.4.3	PC -> DAGLY + PCHO	BLASTP
AO090103000073	phospholipase C	3.1.4.3	PC -> DAGLY + PCHO	BLASTP
AO090005001097	phospholipase C	3.1.4.3	PE -> DAGLY + PETHM	BLASTP
AO090103000073	phospholipase C	3.1.4.3	PE -> DAGLY + PETHM	BLASTP

Gene name	Enzyme name	EC number	Biochemical reaction	Annotation method
**# 6. Cofactor Pathway**				
**# Folate biosynthesis**				
AO090005000616	Methylenetetrahydrofolate dehydrogenase (NAD)	1.5.1.15	METTHF + NAD -> METHF + NADH	BLASTP
AO090009000139	Methylenetetrahydrofolate dehydrogenase (NAD)	1.5.1.15	METTHF + NAD -> METHF + NADH	BLASTP
AO090005001405	Methylenetetrahydrofolate dehydrogenase (NADP)	1.5.1.5	METTHFm + NADPm -> METHFm + NADPHm	BLASTP
AO090026000520	Methylenetetrahydrofolate reductase	1.5.1.20	METTHFm + NADPHm -> MTHFm + NADPm	BLASTP
AO090102000557	Methylenetetrahydrofolate reductase	1.5.1.20	METTHFm + NADPHm -> MTHFm + NADPm	BLASTP
AO090005001405	Methenyltetrahydrofolate cyclohydrolase	3.5.4.9	FTHFm -> METHFm + H2Om	BLASTP
AO090005001405	Formate-dihydrofolate ligase	6.3.4.3	ATPm + FORm + THFm -> ADPm + PIm + FTHFm	BLASTP
AO090005000463	5-formyltetrahydrofolate cyclo-ligase	6.3.3.2	ATPm + FTHFm -> ADPm + PIm + MTHFm	GFAOP
AO090005000463	5-formyltetrahydrofolate cyclo-ligase	6.3.3.2	ATP + FTHF -> ADP + PI + MTHF	BLASTP
AO090023000763	Folylpolyglutamate synthetase	6.3.2.17	THF + ATP + GLU <-> ADP + PI + THFG	BLASTP
AO090023000912	Folylpolyglutamate synthetase	6.3.2.17	THF + ATP + GLU <-> ADP + PI + THFG	BLASTP
AO090009000308	Folylpolyglutamate synthetase	6.3.2.17	THF + ATP + GLU <-> ADP + PI + THFG	BLASTP
AO090001000235	GTP cyclohydrolase I	3.5.4.16	GTP -> FOR + AHTD	GFAOP
AO090102000517	GTP cyclohydrolase I	3.5.4.16	GTP -> FOR + AHTD	GFAOP
AO090012000725	Alkaline phosphatase	3.1.3.1	AHTD -> DHP + 3 PI	BLASTP
AO090120000280	Alkaline phosphatase	3.1.3.1	AHTD -> DHP + 3 PI	BLASTP
AO090023000621	Alkaline phosphatase	3.1.3.1	AHTD -> DHP + 3 PI	BLASTP
AO090005000279	Alkaline phosphatase	3.1.3.1	AHTD -> DHP + 3 PI	BLASTP
AO090011000651	Dihydroneopterin aldolase	4.1.2.25	DHP -> AHHMP + GLAL	BLASTP
AO090011000651	6-Hydroxymethyl-7,8 dihydropterin pyrophosphokinase	2.7.6.3	AHHMP + ATP -> AMP + AHHMD	BLASTP
AO090011000651	Dihydropteroate synthase	2.5.1.15	PABA + AHHMD -> PPI + DHPT	BLASTP
AO090011000651	Dihydropteroate synthase	2.5.1.15	PABA + AHHMP -> DHPT	BLASTP
AO090023000912	Dihydrofolate synthase	6.3.2.12	DHPT + ATP + GLU -> ADP + PI + DHF	BLASTP
AO090011000807	Dihydrofolate reductase	1.5.1.3	DHFm + NADPHm -> NADPm + THFm	BLASTP
AO090020000337	Dihydrofolate reductase	1.5.1.3	DHF + NADPH -> NADP + THF	BLASTP
AO090701000057	Para-aminobenzoate synthase	6.3.5.8	CHOR + GLN -> ADCHOR + GLU	BLASTP
GAP	Aminodeoxychorismate lyase	4.1.3.38	ADCHOR -> PYR + PABA	NONE

Gene name	Enzyme name	EC number	Biochemical reaction	Annotation method
AO090023000412	Formyltetrahydrofolate deformylase	3.5.1.10	FTHF + H2O -> FOR + THF	BLASTP
AO090003000074	Methionyl-tRNA formyltransferase	2.1.2.9	FTHFm + LMETTRNAm + H2Om -> THFm + FOFMETm	BLASTP
AO090003000793	Methionyl-tRNA formyltransferase	2.1.2.9	FTHFm + LMETTRNAm + H2Om -> THFm + FOFMETm	BLASTP
AO090011000915	Glycine hydroxymethyltransferase	2.1.2.1	THFm + SERm <-> GLYm + METTHFm	BLASTP
AO090005001302	Glycine hydroxymethyltransferase	2.1.2.1	THF + SER <-> GLY + METTHF	BLASTP
AO090011000544	Glycinamide ribotide transformylase	2.1.2.2	GAR + FTHF -> THF + FGAR	GFAOP
**# Coenzyme A and pantothenate biosynthesis**				
AO090005000300	3-methyl-2-oxobutanoate hydroxymethyltransferase	2.1.2.11	OIVAL + METTHF -> AKP + THF	BLASTP
AO090001000594	3-methyl-2-oxobutanoate hydroxymethyltransferase	2.1.2.11	OIVALm + METTHFm -> AKPm + THFm	BLASTP
AO090009000600	2-dehydropantoate 2-reductase	1.1.1.169	AKP + NADPH -> NADP + PANT	BLASTP
AO090020000112	2-dehydropantoate 2-reductase	1.1.1.169	AKP + NADPH -> NADP + PANT	BLASTP
AO090012000235	2-dehydropantoate 2-reductase	1.1.1.169	AKP + NADPH -> NADP + PANT	BLASTP
AO090701000557	Ketol-acid reductoisomerase	1.1.1.169	AKPm + NADPHm -> NADPm + PANTm	BLASTP
GAP	Aspartate 1-decarboxylase	4.1.1.11	ASP -> CO2 + bALA	NONE
AO090026000745	Pantoate--beta-alanine ligase	6.3.2.1	PANT + bALA + ATP -> AMP + PPI + PNTO	BLASTP
AO090020000205	Pantothenate kinase	2.7.1.33	PNTO + ATP -> ADP + 4PPNTO	BLASTP
AO090020000202	Phosphopantothenate-cysteine ligase	6.3.2.5	4PPNTO + CTP + CYS -> CMP + PPI + 4PPNCYS	GFAOP
AO090020000202	Phosphopantothenoylcysteine decarboxylase	4.1.1.36	4PPNCYS -> CO2 + 4PPNTE	BLASTP
GAP	Pantetheine-phosphate adenylyltransferase	2.7.7.3	4PPNTE + ATP -> PPI + DPCOA	NONE
AO090003001319	Dephospho-CoA kinase	2.7.1.24	DPCOA + ATP -> ADP + COA	BLASTP
AO090206000106	4'-phosphopantetheinyl transferase	2.7.8.7	COAm -> PAPm + ACPm	GFAOP
AO090206000106	4'-phosphopantetheinyl transferase	2.7.8.7	COA -> PAP + ACP	BLASTP
**#Thiamine Metabolism**				
GAP	Thiamine biosynthesis protein (ThiC)	No_EC	AIR -> AHM	NONE
GAP	Hydroxymethylpyrimidine kinase	2.7.1.49	AHM + ATP -> AHMP + ADP	NONE
AO090011000561	Phosphomethylpyrimidine kinase	2.7.4.7	AHMP + ATP -> AHMPP + ADP	BLASTP
ZY087836	Phosphomethylpyrimidine kinase	2.7.4.7	AHMP + ATP -> AHMPP + ADP	EST
AO090011000799	Hydroxyethylthiazole kinase	2.7.1.50	THZ + ATP -> THZP + ADP	BLASTP

Gene name	Enzyme name	EC number	Biochemical reaction	Annotation method
AO090011000799	Thiamine-phosphate diphosphorylase	2.5.1.3	THZP + AHMPP -> THMP + PPI	BLASTP
GAP	Thiamine kinase	2.7.1.89	ATP + THME -> ADP + THMP	NONE
GAP	Thiamine-phosphate kinase	2.7.4.16	THMP + ATP <-> TPP + ADP	NONE
AO090012000697	Thiamine pyrophosphokinase	2.7.6.2	ATP + THME -> AMP + THDP	BLASTP
AO090026000733	Thiamine pyrophosphokinase	2.7.6.2	ATP + THME -> AMP + THDP	BLASTP
**# NAD AND NADP Conversion**				
AO090102000470/ AO090102000471/ AO090102000473	Nicotinamide nucleotide transhydrogenase	1.6.1.2	NADPHm + NADm <-> NADPm + NADHm	BLASTP
AO090009000675	NADH kinase	2.7.1.86	NADHm + ATPm -> ADPm + NADPHm	BLASTP
GAP	NAD nucleosidase	3.2.2.5	NADm + H2Om -> ADPRIBm + NICDm	NONE
AO090102000541	NADH pyrophosphatase	3.6.1.22	NADm + H2Om -> AMPm + NMNm	BLASTP
GAP	NMN nucleosidase	3.2.2.14	NMNm + H2Om -> R5Pm + NICDm	NONE
AO090001000095	Nicotinamidase	3.5.1.19	NICDm + H2Om -> NICNATEm + NH3m	BLASTP
AO090003001400	Nicotinamidase	3.5.1.19	NICD + H2O -> NICNATE + NH3	BLASTP
AO090005001244	Nicotinamidase	3.5.1.19	NICD + H2O -> NICNATE + NH3	BLASTP
AO090001000561	Nicotinamide mononucleotide adenylyl transferase	2.7.7.1	ATP + NICNUCLE -> PPI + DMNAD	BLASTP
AO090102000540	Glutamine dependent NAD synthetase	6.3.5.1	ATP + DMNAD + GLN + H2O -> AMP + PPI + NAD + GLU	GFAOP
No_Gene	Spontaneous conversion	No_EC	CMUSA -> H2O + QA	BLASTP
AO090020000041	Nicotinate-nucleotide pyrophosphorylase	2.4.2.19	QA + PRPP -> NICNUCLE + PPI + CO2	BLASTP
AO090120000404	NAD kinase	2.7.1.23	ATP + NAD -> ADP + NADP	GFAOP
AO090701000420	NAD kinase	2.7.1.23	ATP + NAD -> ADP + NADP	GFAOP
AO090038000565	Nicotinate phosphoribosyltransferase	2.4.2.11	NICNATE + PRPP + H2O -> PPI + NICNUCLE	BLASTP
**# Vitamin B6 Metabolism**				
AO090701000725	Pyridoxal 5'-phosphate synthase	4.-.-.-	RL5P -> T3P1 + PDXL5PI	BLASTP
AO090005000781	Pyridoxal kinase	2.7.1.35	PDXAM + ATP -> PDXAM5PI + ADP	BLASTP
AO090005000781	Pyridoxal kinase	2.7.1.35	PDXAL + ATP -> PDXL5PI + ADP	BLASTP
AO090010000473	Pyridoxamine phosphate oxidase	1.4.3.5	PDXAM5PI + H2O + O2 -> PDXL5PI + NH3 + H2O2	BLASTP
AO090020000331	Pyridoxamine phosphate oxidase	1.4.3.5	PDXAM5PI + H2O + O2 -> PDXL5PI + NH3 + H2O2	BLASTP

Gene name	Enzyme name	EC number	Biochemical reaction	Annotation method
AO090005000781	Pyridoxal kinase	2.7.1.35	PDXI + ATP -> PDXI5PI + ADP	BLASTP
AO090010000473	Pyridoxamine phosphate oxidase	1.4.3.5	PDXI5PI + H2O + O2 -> PDXL5PI + NH3 + H2O2	BLASTP
AO090020000331	Pyridoxamine phosphate oxidase	1.4.3.5	PDXI5PI + H2O + O2 -> PDXL5PI + NH3 + H2O2	BLASTP
AO090010000473	Pyridoxamine phosphate oxidase	1.4.3.5	PDXAL + H2O + O2 <-> PDXI + NH3 + H2O2	BLASTP
AO090020000331	Pyridoxamine phosphate oxidase	1.4.3.5	PDXAL + H2O + O2 <-> PDXI + NH3 + H2O2	BLASTP
AO090010000473	Pyridoxamine phosphate oxidase	1.4.3.5	PDXAL + H2O + O2 <-> PDXAM + NH3 + H2O2	BLASTP
AO090020000331	Pyridoxamine phosphate oxidase	1.4.3.5	PDXAL + H2O + O2 <-> PDXAM + NH3 + H2O2	BLASTP
**# Porphyrin and Chrophyll metabolism**				
GAP	Glutamyl-tRNA reductase	1.2.1.70	LGLUTRNA + NADPH -> GLU1SAL + TRNA + NADP	NONE
AO090020000375	Glutamate-1-semialdehyde 2,1-aminomutase	5.4.3.8	GLU1SAL -> AMIEVUL	BLASTP
AO090009000630	5-aminolevulinic acid synthase	2.3.1.37	SUCCOAm + GLYm -> AMIEVULm + COAm + CO2m	BLASTP
AO090005001652	Porphobilinogen synthase	4.2.1.24	2 AMIEVUL -> PPBG + H2O	BLASTP
AO090120000294	Hydroxymethylbilane synthase	2.5.1.61	4 PPBG + H2O -> HMTB + 4 NH3	BLASTP
AO090012000205	Hydroxymethylbilane synthase	2.5.1.61	4 PPBG + H2O -> HMTB + 4 NH3	BLASTP
AO090103000151	Hydroxymethylbilane synthase	2.5.1.61	4 PPBG + H2O -> HMTB + 4 NH3	BLASTP
AO090003000401	Uroporphyrinogen-III synthase	4.2.1.75	HMTB -> UPGIII + H2O	BLASTP
AO090003000803	Uroporphyrinogen decarboxylase	4.1.1.37	UPGIII -> CPGIII + 4 CO2	BLASTP
AO090012000998	Coproporphyrinogen III oxidase	1.3.3.3	CPGIIIm + O2m -> PPGIXm + 2 CO2m + 2 H2Om	BLASTP
ZY087286	Coproporphyrinogen III oxidase	1.3.3.3	CPGIIIm + O2m -> PPGIXm + 2 CO2m + 2 H2Om	EST
AO090001000500	Protoporphyrinogen oxidase	1.3.3.4	PPGIXm + O2m -> PPRIXm + H2Om	BLASTP
AO090012000855	Protoporphyrinogen oxidase	1.3.3.4	PPGIXm + O2m -> PPRIXm + H2Om	BLASTP
AO090701000682	Ferrochelatase	4.99.1.1	PPRIXm -> PHEMEm	BLASTP
AO090023000725	Heme A farnesyltransferase	2.5.1.-	PHEMEm -> HEME_Om	BLASTP
AO090003000180	cytochrome oxidase assembly factor COX15	COX15	HEME_Om -> HEME_Am	BLASTP
AO090023000863	Cytochrome c1 heme lyase	4.4.1.17	PHEMEm + APOCEm -> CYMECm	BLASTP
AO090206000030	Cytochrome c1 heme lyase	4.4.1.17	PHEMEm + APOCEm -> CYMECm	BLASTP
AO090020000339	Uroporphyrin-III C-methyltransferase	2.1.1.107	SAM + UPGIII -> SAH + PRECOR	BLASTP
AO090005000430	Precorrin-2 dehydrogenase	1.3.1.76	PRECOR + NAD -> SHCR + NADH	BLASTP
AO090005000430	Sirohydrochlorin ferrochelatase	4.99.1.4	SHCR -> SIHM	BLASTP

Gene name	Enzyme name	EC number	Biochemical reaction	Annotation method
**# Ubiquinone biosynthesis**				
GAP	Chorismate pyruvate lyase	4.1.3.-	CHOR -> 4HBZ + PYR	NONE
AO090023001001	4-hydroxybenzoate octaprenyltransferase	2.5.1.-	4HBZ + NPP -> N4HBZ + PPI	GFAOP
AO090001000093	3-octaprenyl-4-hydroxybenzoate carboxy-lyase	4.1.1.-	N4HBZ -> CO2 + 2NPPP	GFAOP
AO090003000424	3-octaprenyl-4-hydroxybenzoate carboxy-lyase	4.1.1.-	N4HBZ -> CO2 + 2NPPP	GFAOP
AO090003000423	3-octaprenyl-4-hydroxybenzoate carboxy-lyase	4.1.1.-	N4HBZ -> CO2 + 2NPPP	GFAOP
GAP	2-octaprenylphenol hydroxylase	1.13.14.-	2NPPP + O2 -> 2N6H	NONE
GAP	3-demethylubiquinone-9 3-O-methyltransferase	2.1.1.64	2N6H + SAM -> 2NPMP + SAH	NONE
AO090009000294	2-octaprenyl-6-methoxyphenol hydroxylase	1.14.13.-	2NPMPm + O2m -> 2NPMBm	NONE
AO090001000559	Ubiquinone biosynthesis methlytransferase	2.1.1.-	2NPMBm + SAMm -> 2NPMMBm + SAHm	BLASTP
AO090011000456	2-octaprenyl-3-methyl-6-methoxy-1,4-benzoquinone hydroxylase	1.14.13.-	2NPMMBm + O2m -> 2NMHMBm	NONE
GAP	3-demethylubiquinone-9 3-O-methyltransferase	2.1.1.64	2NMHMBm + SAMm -> Qm + SAHm	NONE
ZY080692	P-hydroxybenzoate hydroxylase	1.14.13.2	4HBZ + NADPH + O2 -> PCACE + NADP + H2O	EST
**# Riboflavin, FMN and FAD metabolism**				
AO090012000551	GTP cyclohydrolase II	3.5.4.25	GTP -> D6RP5P + FOR + PPI	BLASTP
AO090701000398	GTP cyclohydrolase II	3.5.4.25	GTP -> D6RP5P + FOR + PPI	BLASTP
GAP	Diaminohydroxyphosphoribosylaminopyrimidine deaminase	3.5.4.26	D6RP5P -> A6RP5P + NH3	NONE
AO090206000013	5-amino-6-(5-phosphoribosylamino)uracil reductase	1.1.1.193	A6RP5P + NADPH -> A6RP5P2 + NADP	NONE
GAP	Pyrimidine phosphatase	3.1.3.-	A6RP5P2 + H2O -> A6RP + PI	NONE
AO090003000439	3,4-dihydroxy-2-butanone 4-phosphate synthase	No_EC	RL5P -> DB4P + FOR	NONE
AO090003000004	6,7-dimethyl-8-ribityllumazine synthase	No_EC	DB4P + A6RP -> D8RL + PI + 2 H2O	BLASTP
AO090003000004	Riboflavin synthase	2.5.1.9	D8RL -> A6RP + RIBFLAV	GFAOP
AO090001000426	Riboflavin synthase	2.5.1.9	D8RL -> A6RP + RIBFLAV	GFAOP
AO090001000701	Riboflavin kinase	2.7.1.26	RIBFLAV + ATP -> FMN + ADP	BLASTP
AO090023000516	FMN adenylyltransferase	2.7.7.2	FMN + ATP -> FAD + PPI	BLASTP
AO090103000338	Acid phosphatase	3.1.3.2	FMN -> RIBFLAV + PI	BLASTP
AO090103000018	Acid phosphatase	3.1.3.2	FMN -> RIBFLAV + PI	BLASTP
AO090011000300	Acid phosphatase	3.1.3.2	FMNe -> RIBFLAVe + PIe	BLASTP
AO090011000115	Acid phosphatase	3.1.3.2	FMNe -> RIBFLAVe + PIe	BLASTP

Gene name	Enzyme name	EC number	Biochemical reaction	Annotation method
AO090009000416	Acid phosphatase	3.1.3.2	FMNe -> RIBFLAVe + PIe	BLASTP
**# Biotin Metabolism**				
GAP	6-carboxyhexanoate-CoA ligase	6.2.1.14	ATP + 6CARHEX + COA -> AMP + PPI + CHCOA	NONE
AO090009000589	8-amino-7-oxononanoate synthase	2.3.1.47	CHCOA + ALA -> AONA + COA + CO2	BLASTP
AO090038000549	8-amino-7-oxononanoate synthase	2.3.1.47	CHCOA + ALA -> AONA + COA + CO2	BLASTP
AO090010000518	Adenosylmethionine-8-amino-7-oxononanoate transaminase	2.6.1.62	SAMm + AONAm -> SAMOBm + DANNAm	BLASTP
AO090010000518	Adenosylmethionine-8-amino-7-oxononanoate transaminase	2.6.1.62	SAM + AONA -> SAMOB + DANNA	BLASTP
AO090009000590	Dethiobiotin synthase	6.3.3.3	CO2 + DANNA + ATP -> DTB + PI + ADP	BLASTP
AO090005001069	Biotin synthase	2.8.1.6	DTB + 2 SAM + S -> BT + 2 MET + DA	BLASTP
AO090011000708	Biotin holocarboxylase synthetase/biotin-protein ligase	6.3.4.9/6.3.4.10/ 6.3.4.11/ 6.3.4.15	BT -> BTAMP	BLASTP
AO090011000708	Biotin holocarboxylase synthetase/biotin-protein ligase	6.3.4.9/6.3.4.10/ 6.3.4.11/6.3.4.15	BTAMP -> HCXY	BLASTP
**# 7. Secondary metabolism**				
**# Penincillin biosynthesis**				
AO090003000661	Phenylpyruvate decarboxylase	4.1.1.43	PHPYR <-> PHACAL + CO2	BLASTP
AO090009000222	Aldehyde dehydrogenase (NADP)	1.2.1.5	PHACAL + NAD + H2O <-> PHAC + NADH	BLASTP
AO090011000620	Phenylacetyl-CoA ligase	6.2.1.30	ATP + PHAC + COA <-> AMP + PPI + PHAC-COA	BLASTP
AO090701000311	Phenylacetyl-CoA ligase	6.2.1.30	ATP + PHAC + COA <-> AMP + PPI + PHAC-COA	BLASTP
AO090001000200	Phenylacetyl-CoA ligase	6.2.1.30	ATP + PHAC + COA <-> AMP + PPI + PHAC-COA	BLASTP
AO090012000579	Phenylacetyl-CoA ligase	6.2.1.30	ATP + PHAC + COA <-> AMP + PPI + PHAC-COA	BLASTP
AO090102000062	Phenylacetyl-CoA ligase	6.2.1.12	ATP + PHAC + COA <-> AMP + PPI + PHAC-COA	BLASTP
AO090038000543	N-(5-Amino-5-carboxypentanoyl)-L-cysteinyl-D-valine synthase	6.3.2.26	AMA + CYS + VAL + 3 ATP -> LLDACV + 3 AMP + 3 PPI	BLASTP
AO090038000544	Isopenicillin-N synthase	1.21.3.1	LLDACV + O2 -> IPN + 2 H2O	BLASTP

Gene name	Enzyme name	EC number	Biochemical reaction	Annotation method
AO090038000545	Isopenicillin-N N-acyltransferase	2.3.1.164	IPN + PHAC-COA -> PEN + AMA + COA	BLASTP
AO090102000584	Isopenicillin N-CoA epimerase	5.1.1.17	IPN -> PENN	BLASTP
AO090005000338	Beta-lactamase	3.5.2.6	PEN -> PENACID	BLASTP
AO090010000330	Beta-lactamase	3.5.2.6	PEN -> PENACID	BLASTP
AO090010000427	Beta-lactamase	3.5.2.6	PEN -> PENACID	BLASTP
AO090012000033	Beta-lactamase	3.5.2.6	PEN -> PENACID	BLASTP
AO090701000879	Beta-lactamase	3.5.2.6	PEN -> PENACID	BLASTP
**# Sterigmatocystin/Aflatoxin biosynthesis**				
AO090026000012	Fatty acid synthase	No_EC	ACCOA + 9 MALCOA + O2 -> NOR	BLASTP, Gene Cluster
AO090026000013	Fatty acid synthase	No_EC	ACCOA + 9 MALCOA + O2 -> NOR	BLASTP, Gene Cluster
AO090026000009	Polyketide syntase	No_EC	ACCOA + 9 MALCOA + O2 -> NOR	BLASTP, Gene Cluster
AO090026000011	Norsolorinic acid reductase	1.1.1.-	NOR + NADPH -> NADP + AVN	BLASTP, Gene Cluster
AO090026000018	Norsolorinic acid reductase	1.1.1.-	NOR + NADPH -> NADP + AVN	BLASTP, Gene Cluster
AO090026000010	Norsolorinic acid reductase	1.1.1.-	NOR + NADPH -> NADP + AVN	BLASTP, Gene Cluster
AO090026000021	cytochrome P450 monooxygenase	1.14.-.-	AVN + NADPH + O2 -> NADP + H2O + HAVN	BLASTP, Gene Cluster
ZY006677	cytochrome P450 monooxygenase	1.14.-.-	AVN + NADPH + O2 -> NADP + H2O + HAVN	EST
AO090026000030	Sterigmatocystin biosynthesis monooxygenase	1.14.-.-	AVN + NADPH + O2 -> NADP + H2O + HAVN	BLASTP, Gene Cluster
AO090026000020	Sterigmatocystin biosynthesis P450 monooxygenase	1.14.-.-	AVN + NADPH + O2 -> NADP + H2O + HAVN	BLASTP, Gene Cluster
AO090026000016	Short-chain alcohol dehydrogenases	No_EC	HAVN + SAM + NAD -> NADH + AVF	BLASTP, Gene Cluster
AO090026000024	Sterigmatocystin biosynthesis protein stcO	No_EC	AVF + O2 -> H2O + VHA	BLASTP, Gene Cluster
AO090026000017	Sterigmatocystin biosynthesis lipase/esterase STCI	No_EC	VHA + H2O -> SAM + VERAL	BLASTP, Gene Cluster
AO090026000028	Versicolorin B synthase	No_EC	VERAL -> H2O + VERB	BLASTP, Gene Cluster
AO090026000029	Sterigmatocystin biosynthesis P450 monooxygenase	1.14.-	VERB + NAD -> NADH + VERA	BLASTP, Gene Cluster
AO090026000019	1,3,6,8-tetrahydroxynaphthalene reductase	1.1.-.-	VERA + NADPH -> NADP + H2O + DMST	BLASTP
AO090026000022	Sterigmatocystin biosynthesis P450 monooxygenase	1.14.-.-	VERA + NADPH -> NADP + H2O + DMST	BLASTP
AO090026000019	1,3,6,8-tetrahydroxynaphthalene reductase	1.1.-.-	VERB + NADPH -> NADP + H2O + DHDMST	BLASTP

Gene name	Enzyme name	EC number	Biochemical reaction	Annotation method
AO090026000022	Sterigmatocystin biosynthesis P450 monooxygenase	1.14.-.-	VERB + NADPH -> NADP + H2O + DHDMST	BLASTP
AO090001000024	Demethylsterigmatocystin 6-O-methyltransferase	2.1.1.109	DMST + SAM -> SAH + ST	BLASTP
AO090001000024	Demethylsterigmatocystin 6-O-methyltransferase	2.1.1.109	DHDMST + SAM -> SAH + DHST	BLASTP
AO090026000026	Demethylsterigmatocystin 6-O-methyltransferase	2.1.1.110	ST + SAM -> SAH + OMST	BLASTP, Gene Cluster
AO090026000025	Demethylsterigmatocystin 6-O-methyltransferase	2.1.1.110	ST + SAM -> SAH + OMST	BLASTP, Gene Cluster
AO090026000026	Demethylsterigmatocystin 6-O-methyltransferase	2.1.1.110	DHST + SAM -> SAH + DHOMST	BLASTP, Gene Cluster
AO090026000025	Demethylsterigmatocystin 6-O-methyltransferase	2.1.1.110	DHST + SAM -> SAH + DHOMST	BLASTP, Gene Cluster
AO090026000027	O-Methylsterigmatocystin oxidoreductase	1.14.1.-	OMST + NADPH + O2 -> NADP + H2O + AFB1	BLASTP, Gene Cluster
AO090026000014	O-Methylsterigmatocystin oxidoreductase	1.14.1.-	OMST + NADPH + O2 -> NADP + H2O + AFB1	BLASTP, Gene Cluster
AO090026000015	O-Methylsterigmatocystin oxidoreductase	1.14.1.-	OMST + NADPH + O2 -> NADP + H2O + AFB1	BLASTP, Gene Cluster
AO090026000023	O-Methylsterigmatocystin oxidoreductase	1.14.1.-	OMST + NADPH + O2 -> NADP + H2O + AFB1	BLASTP, Gene Cluster
AO090005000070	O-Methylsterigmatocystin oxidoreductase	1.14.1.-	OMST + NADPH + O2 -> NADP + H2O + AFB1	BLASTP
AO090026000027	O-Methylsterigmatocystin oxidoreductase	1.14.1.-	OMST + NADPH + O2 -> NADP + H2O + AFG1	BLASTP, Gene Cluster
AO090026000014	O-Methylsterigmatocystin oxidoreductase	1.14.1.-	OMST + NADPH + O2 -> NADP + H2O + AFG1	BLASTP, Gene Cluster
AO090026000015	O-Methylsterigmatocystin oxidoreductase	1.14.1.-	OMST + NADPH + O2 -> NADP + H2O + AFG1	BLASTP, Gene Cluster
AO090026000023	O-Methylsterigmatocystin oxidoreductase	1.14.1.-	OMST + NADPH + O2 -> NADP + H2O + AFG1	BLASTP, Gene Cluster
AO090005000070	O-Methylsterigmatocystin oxidoreductase	1.14.1.-	OMST + NADPH + O2 -> NADP + H2O + AFG1	BLASTP
AO090026000027	O-Methylsterigmatocystin oxidoreductase	1.14.1.-	DHOMST + NADPH + O2 -> NADP + H2O + AFB2	BLASTP, Gene Cluster
AO090026000014	O-Methylsterigmatocystin oxidoreductase	1.14.1.-	DHOMST + NADPH + O2 -> NADP + H2O + AFB2	BLASTP, Gene Cluster
AO090026000015	O-Methylsterigmatocystin oxidoreductase	1.14.1.-	DHOMST + NADPH + O2 -> NADP + H2O + AFB2	BLASTP, Gene Cluster
AO090026000023	O-Methylsterigmatocystin oxidoreductase	1.14.1.-	DHOMST + NADPH + O2 -> NADP + H2O + AFB2	BLASTP, Gene Cluster
AO090005000070	O-Methylsterigmatocystin oxidoreductase	1.14.1.-	DHOMST + NADPH + O2 -> NADP + H2O + AFB2	BLASTP
AO090026000027	O-Methylsterigmatocystin oxidoreductase	1.14.1.-	DHOMST + NADPH + O2 -> NADP + H2O + AFG2	BLASTP, Gene Cluster
AO090026000014	O-Methylsterigmatocystin oxidoreductase	1.14.1.-	DHOMST + NADPH + O2 -> NADP + H2O + AFG2	BLASTP, Gene Cluster
AO090026000015	O-Methylsterigmatocystin oxidoreductase	1.14.1.-	DHOMST + NADPH + O2 -> NADP + H2O + AFG2	BLASTP, Gene Cluster
AO090026000023	O-Methylsterigmatocystin oxidoreductase	1.14.1.-	DHOMST + NADPH + O2 -> NADP + H2O + AFG2	BLASTP, Gene Cluster
AO090005000070	O-Methylsterigmatocystin oxidoreductase	1.14.1.-	DHOMST + NADPH + O2 -> NADP + H2O + AFG2	BLASTP
**# Flavonoid Biosynthesis**				
GAP	Cinnamate 4-hydroxylase	1.14.13.11	CINNAM + NADPH + O2 -> 4CMRE + H2O + NADP	NONE
AO090005000532	Phenylalanine ammonia-lyase	4.3.1.5	TYR -> NH3 + 4CMRE	BLASTP
AO090011000788	Phenylalanine ammonia-lyase	4.3.1.5	TYR -> NH3 + 4CMRE	BLASTP

Gene name	Enzyme name	EC number	Biochemical reaction	Annotation method
AO090701000601	Phenylalanine ammonia-lyase	4.3.1.5	TYR -> NH3 + 4CMRE	BLASTP
AO090026000586	Phenylalanine ammonia-lyase	4.3.1.5	TYR -> NH3 + 4CMRE	BLASTP
AO090102000033	4-coumarate-CoA ligase	6.2.1.12	4CMRE + ATP + COA -> 4CMCOA + AMP + PPI	BLASTP
AO090102000165	4-coumarate-CoA ligase	6.2.1.12	4CMRE + ATP + COA -> 4CMCOA + AMP + PPI	BLASTP
AO090120000376	4-coumarate-CoA ligase	6.2.1.12	4CMRE + ATP + COA -> 4CMCOA + AMP + PPI	BLASTP
AO090124000040	Naringenin-chalcone synthase	2.3.1.74	4CMCOA + 3 MALCOA -> 4 COA + 3 CO2 + NCACE	BLASTP
AO090701000566	Naringenin-chalcone synthase	2.3.1.74	4CMCOA + 3 MALCOA -> 4 COA + 3 CO2 + NCACE	BLASTP
GAP	Chalcone isomerase	5.5.1.6	NCACE -> NGEN	NONE
AO090005001560	Flavonoid 3'-monooxygenase	1.14.13.21	NGEN + O2 + NADPH -> ERIDICOL + NADP + H2O	BLASTP
AO090166000121	2'-hydroxyisoflavone reductase	1.3.1.45	NGEN -> FERRIN	BLASTP
AO090166000121	2'-hydroxyisoflavone reductase	1.3.1.45	NGEN -> VESTINE	BLASTP
**# Melanin biosynthesis**				
AO090038000061	Tyrosinase	1.14.18.1	TYRe + DOPAe + O2e -> DOPAe + DOQUIe + H2Oe	GFAOP
AO090001000383	Tyrosinase	1.14.18.1	TYRe + DOPAe + O2e -> DOPAe + DOQUIe + H2Oe	GFAOP
AO090001000117	Tyrosinase	1.14.18.1	TYRe + DOPAe + O2e -> DOPAe + DOQUIe + H2Oe	GFAOP
AO090026000145	Tyrosinase	1.14.18.1	TYRe + DOPAe + O2e -> DOPAe + DOQUIe + H2Oe	GFAOP
AO090012000590	Tyrosinase	1.14.18.1	TYR + DOPA + O2 -> DOPA + DOQUI + H2O	GFAOP
AO090023000424	Tyrosinase	1.14.18.1	TYR + DOPA + O2 -> DOPA + DOQUI + H2O	GFAOP
AO090010000557	Tyrosinase	1.14.18.1	TYR + DOPA + O2 -> DOPA + DOQUI + H2O	GFAOP
AO090026000145	Tyrosinase	1.14.18.1	TYR + DOPA + O2 -> DOPA + DOQUI + H2O	GFAOP
AO090701000859	Tyrosinase	1.14.18.1	TYR + DOPA + O2 -> DOPA + DOQUI + H2O	GFAOP
AO090113000032	Tyrosinase	1.14.18.1	TYR + DOPA + O2 -> DOPA + DOQUI + H2O	GFAOP
No_Gene	Spontaneous conversion	No_EC	DOQUI -> DIHINDOLE	NONE
No_Gene	Oxidation reaction	No_EC	DIHINDOLE -> MENIN	NONE
**# Pentaketide melanin biosynthesis**				
GAP	Heptaketide synthase	No_EC	ACCOA + MALCOA -> 1368THN	NONE
GAP	Tetrahydroxynaphthalene reductase	1.1.1.252	1368THN + NADPH -> SCTLE + NADP	NONE
ZY111007	Scytalone dehydratase	4.2.1.94	SCTLE -> 138THN	EST
GAP	Tetrahydroxynaphthalene reductase	1.1.1.252	138THN + NADPH -> VEML + NADP	NONE
GAP	Dehydration and reduction	No_EC	VEML -> 18DHN	NONE
GAP	Polymerization reaction	No_EC	18DHN -> MENIN	NONE

Gene name	Enzyme name	EC number	Biochemical reaction	Annotation method
**# Transport reaction**				
**# Plasma membrane transport**				
**# Carbohydrates**				
PMT_DGLC	D-Glucose plasma membrane transport	No_EC	DGLCe -> DGLC	NONE
AO090020000259	MFS monosaccharide transporter	No_EC	GLCe -> GLC	BLASTP
AO090026000494	MFS monosaccharide transporter	No_EC	GLCe -> GLC	BLASTP
AO090010000126	MFS monosaccharide transporter	No_EC	GLCe -> GLC	BLASTP
AO090020000696	MFS monosaccharide transporter	No_EC	GLCe -> GLC	BLASTP
AO090038000167	MFS monosaccharide transporter	No_EC	GLCe -> GLC	BLASTP
AO090102000255	MFS monosaccharide transporter	No_EC	GLCe -> GLC	BLASTP
AO090010000470	MFS monosaccharide transporter	No_EC	GLCe -> GLC	BLASTP
AO090020000259	MFS monosaccharide transporter	No_EC	bDGLCe -> bDGLC	BLASTP
AO090026000494	MFS monosaccharide transporter	No_EC	bDGLCe -> bDGLC	BLASTP
AO090010000126	MFS monosaccharide transporter	No_EC	bDGLCe -> bDGLC	BLASTP
AO090020000696	MFS monosaccharide transporter	No_EC	bDGLCe -> bDGLC	BLASTP
AO090038000167	MFS monosaccharide transporter	No_EC	bDGLCe -> bDGLC	BLASTP
AO090102000255	MFS monosaccharide transporter	No_EC	bDGLCe -> bDGLC	BLASTP
AO090010000470	MFS monosaccharide transporter	No_EC	bDGLCe -> bDGLC	BLASTP
AO090020000259	MFS monosaccharide transporter	No_EC	GLACe -> GLAC	BLASTP
AO090026000494	MFS monosaccharide transporter	No_EC	GLACe -> GLAC	BLASTP
AO090010000126	MFS monosaccharide transporter	No_EC	GLACe -> GLAC	BLASTP
AO090020000696	MFS monosaccharide transporter	No_EC	GLACe -> GLAC	BLASTP
AO090038000167	MFS monosaccharide transporter	No_EC	GLACe -> GLAC	BLASTP
AO090102000255	MFS monosaccharide transporter	No_EC	GLACe -> GLAC	BLASTP
AO090010000470	MFS monosaccharide transporter	No_EC	GLACe -> GLAC	BLASTP
AO090020000259	MFS monosaccharide transporter	No_EC	FRUe -> FRU	BLASTP
AO090026000494	MFS monosaccharide transporter	No_EC	FRUe -> FRU	BLASTP
AO090010000126	MFS monosaccharide transporter	No_EC	FRUe -> FRU	BLASTP
AO090020000696	MFS monosaccharide transporter	No_EC	FRUe -> FRU	BLASTP
AO090038000167	MFS monosaccharide transporter	No_EC	FRUe -> FRU	BLASTP
AO090102000255	MFS monosaccharide transporter	No_EC	FRUe -> FRU	BLASTP

Gene name	Enzyme name	EC number	Biochemical reaction	Annotation method
AO090010000470	MFS monosaccharide transporter	No_EC	FRUe -> FRU	BLASTP
AO090020000259	MFS monosaccharide transporter	No_EC	MANe -> MAN	BLASTP
AO090026000494	MFS monosaccharide transporter	No_EC	MANe -> MAN	BLASTP
AO090010000126	MFS monosaccharide transporter	No_EC	MANe -> MAN	BLASTP
AO090020000696	MFS monosaccharide transporter	No_EC	MANe -> MAN	BLASTP
AO090038000167	MFS monosaccharide transporter	No_EC	MANe -> MAN	BLASTP
AO090102000255	MFS monosaccharide transporter	No_EC	MANe -> MAN	BLASTP
AO090010000470	MFS monosaccharide transporter	No_EC	MANe -> MAN	BLASTP
AO090038000233	Maltose permease	No_EC	MLTe -> MLT	BLASTP
AO090103000130	Maltose permease	No_EC	MLTe -> MLT	BLASTP
AO090011000538	Maltose permease	No_EC	MLTe -> MLT	BLASTP
AO090120000021	Myo-inositol transporter	No_EC	MYOIe -> MYOI	BLASTP
AO090023000988	Myo-inositol transporter	No_EC	MYOIe -> MYOI	BLASTP
PMT_MELI	Melibiose plasma membrane transport	No_EC	MELIe -> MELI	NONE
PMT_TRE	Trehalose plasma membrane transport	No_EC	TREe <-> TRE	NONE
PMT_RIB	Ribose plasma membrane transport	No_EC	RIBe -> RIB	NONE
PMT_MNT	Mannitol plasma membrane transport	No_EC	MNTe <-> MNT	NONE
PMT_SOT	Sorbitol plasma membrane transport	No_EC	SOTe <-> SOT	NONE
PMT_NAG	N-acetylglucosamine plasma membrane transport	No_EC	NAGe -> NAG	NONE
PMT_SOR	Sorbose plasma membrane transport	No_EC	SORe -> SOR	NONE
PMT_ARAB	Arabinose plasma membrane transport	No_EC	ARABe -> ARAB	NONE
PMT_LARAB	L-arabinose plasma membrane transport	No_EC	LARABe -> LARAB	NONE
PMT_GLY	Glycerol plasma membrane transport	No_EC	GLe <-> GL	NONE
PMT_RL	D-Ribulose plasma membrane transport	No_EC	RLe -> RL	NONE
PMT_XYL	D-Xylose plasma membrane transport	No_EC	XYLe -> XYL	NONE
PMT_XUL	D-Xylulose plasma membrane transport	No_EC	XULe -> XUL	NONE
PMT_LXUL	L-Xylulose plasma membrane transport	No_EC	LXULe -> LXUL	NONE
PMT_AOL	D-Arabitol plasma membrane transport	No_EC	AOLe <-> AOL	NONE
PMT_XOL	Xylitol plasma membrane transport	No_EC	XOLe <-> XOL	NONE
PMT_LACT	Lactose plasma membrane transport	No_EC	LACTe -> LACT	NONE
PMT_GLCNT	D-Gluconate plasma membrane transport	No_EC	GLCNTe <-> GLCNT	NONE

Gene name	Enzyme name	EC number	Biochemical reaction	Annotation method
PMT_TAR	Tartrate plasma membrane transport	No_EC	TARe <-> TAR	NONE
PMT_PROP	Propanoate plasma membrane transport	No_EC	PROPe <-> PROP	NONE
PMT_LRL	L-Ribulose plasma membrane transport	No_EC	LRLe -> LRL	NONE
PMT_TGE	Tagatose plasma membrane transport	No_EC	TGEe -> TGE	NONE
PMT_GALNT	D-Galactonate plasma membrane transport	No_EC	GALNTe <-> GALNT	NONE
PMT_GLCN15LAC	D-Glucono-1,5-lactone plasma membrane transport	No_EC	GLCN15LACe <-> GLCN15LAC	NONE
PMT_GALOL	Galactitol plasma membrane transport	No_EC	GALOLe <-> GALOL	NONE
PMT_FMN	FMN plasma membrane transport	No_EC	FMNe <-> FMN	NONE
PMT_RIBFLAV	Riboflavin plasma membrane transport	No_EC	RIBFLAVe <-> RIBFLAV	NONE
PMT_DOPA	L-Dopa plasma membrane transport	No_EC	DOPAe <-> DOPA	NONE
PMT_DOQUI	Dopaquinone plasma membrane transport	No_EC	DOQUIe <-> DOQUI	NONE
PMT_GALUNT	D-Galacturonate plasma membrane transport	No_EC	GALUNTe <-> GALUNT	NONE
PMT_H2O2	H2O2 plasma membrane transport	No_EC	H2O2e <-> H2O2	NONE
PMT_13GLUCAN	1,3-beta-D-Glucan	No_EC	13GLUCANe <-> 13GLUCAN	NONE
PMT_CHIB	Chitobiose plasma membrane transport	No_EC	CHIBe -> CHIB	NONE
PMT_CHITO	Chitosan plasma membrane transport	No_EC	CHITOe -> CHITO	NONE
PMT_CHIT	Chitin plasma membrane transport	No_EC	CHITe <-> CHIT	NONE
PMT_CELLOB	Cellobiose plasma membrane transport	No_EC	CELLOBe <-> CELLOB	NONE
PMT_GLYCOGEN	Glycogen plasma membrane transport	No_EC	GLYCOGENe -> GLYCOGEN	NONE
PMT_STAR	Starch plasma membrane transport	No_EC	STARe -> STAR	NONE
PMT_MANNAN	Mannan plasma membrane transport	No_EC	MANNANe -> MANNAN	NONE
PMT_XYLAN	Xylan plasma membrane transport	No_EC	XYLANe -> XYLAN	NONE
**# Amino acids**				NONE
AO090005000114	Amino acid permease	No_EC	ALAe -> ALA	BLASTP
AO090010000564	Amino acid permease	No_EC	ALAe -> ALA	BLASTP
AO090102000632	Amino acid permease	No_EC	ALAe -> ALA	BLASTP
AO090103000082	Amino acid permease	No_EC	ALAe -> ALA	BLASTP
AO090011000820	Dicarboxylic amino acid permease	No_EC	ALAe -> ALA	BLASTP
AO090005000114	Amino acid permease	No_EC	ARGe -> ARG	BLASTP
AO090010000564	Amino acid permease	No_EC	ARGe -> ARG	BLASTP
AO090102000632	Amino acid permease	No_EC	ARGe -> ARG	BLASTP

Gene name	Enzyme name	EC number	Biochemical reaction	Annotation method
AO090103000082	Amino acid permease	No_EC	ARGe -> ARG	BLASTP
AO090005000114	Amino acid permease	No_EC	ASNe -> ASN	BLASTP
AO090010000564	Amino acid permease	No_EC	ASNe -> ASN	BLASTP
AO090102000632	Amino acid permease	No_EC	ASNe -> ASN	BLASTP
AO090103000082	Amino acid permease	No_EC	ASNe -> ASN	BLASTP
AO090011000820	Dicarboxylic amino acid permease	No_EC	ASNe -> ASN	BLASTP
AO090005000114	Amino acid permease	No_EC	ASPe -> ASP	BLASTP
AO090010000564	Amino acid permease	No_EC	ASPe -> ASP	BLASTP
AO090102000632	Amino acid permease	No_EC	ASPe -> ASP	BLASTP
AO090103000082	Amino acid permease	No_EC	ASPe -> ASP	BLASTP
AO090011000116	Amino acid permease for serine, aspartate and glutamate	No_EC	ASPe -> ASP	BLASTP
AO090011000820	Dicarboxylic amino acid permease	No_EC	ASPe -> ASP	BLASTP
AO090005000114	Amino acid permease	No_EC	CYSe -> CYS	BLASTP
AO090010000564	Amino acid permease	No_EC	CYSe -> CYS	BLASTP
AO090102000632	Amino acid permease	No_EC	CYSe -> CYS	BLASTP
AO090103000082	Amino acid permease	No_EC	CYSe -> CYS	BLASTP
AO090005000114	Amino acid permease	No_EC	GLYe -> GLY	BLASTP
AO090010000564	Amino acid permease	No_EC	GLYe -> GLY	BLASTP
AO090102000632	Amino acid permease	No_EC	GLYe -> GLY	BLASTP
AO090103000082	Amino acid permease	No_EC	GLYe -> GLY	BLASTP
AO090011000820	Dicarboxylic amino acid permease	No_EC	GLYe -> GLY	BLASTP
AO090005000114	Amino acid permease	No_EC	GLUe -> GLU	BLASTP
AO090010000564	Amino acid permease	No_EC	GLUe -> GLU	BLASTP
AO090102000632	Amino acid permease	No_EC	GLUe -> GLU	BLASTP
AO090103000082	Amino acid permease	No_EC	GLUe -> GLU	BLASTP
AO090011000116	Amino acid permease for serine, aspartate and glutamate	No_EC	GLUe -> GLU	BLASTP

Gene name	Enzyme name	EC number	Biochemical reaction	Annotation method
AO090011000820	Dicarboxylic amino acid permease	No_EC	GLUe -> GLU	BLASTP
AO090005000114	Amino acid permease	No_EC	GLNe -> GLN	BLASTP
AO090010000564	Amino acid permease	No_EC	GLNe -> GLN	BLASTP
AO090102000632	Amino acid permease	No_EC	GLNe -> GLN	BLASTP
AO090103000082	Amino acid permease	No_EC	GLNe -> GLN	BLASTP
AO090011000820	Dicarboxylic amino acid permease	No_EC	GLNe -> GLN	BLASTP
AO090005000114	Amino acid permease	No_EC	HISe -> HIS	BLASTP
AO090010000564	Amino acid permease	No_EC	HISe -> HIS	BLASTP
AO090102000632	Amino acid permease	No_EC	HISe -> HIS	BLASTP
AO090103000082	Amino acid permease	No_EC	HISe -> HIS	BLASTP
AO090005000114	Amino acid permease	No_EC	ILEe -> ILE	BLASTP
AO090010000564	Amino acid permease	No_EC	ILEe -> ILE	BLASTP
AO090102000632	Amino acid permease	No_EC	ILEe -> ILE	BLASTP
AO090103000082	Amino acid permease	No_EC	ILEe -> ILE	BLASTP
AO090005000114	Amino acid permease	No_EC	LEUe -> LEU	BLASTP
AO090010000564	Amino acid permease	No_EC	LEUe -> LEU	BLASTP
AO090102000632	Amino acid permease	No_EC	LEUe -> LEU	BLASTP
AO090103000082	Amino acid permease	No_EC	LEUe -> LEU	BLASTP
AO090005000114	Amino acid permease	No_EC	METe -> MET	BLASTP
AO090010000564	Amino acid permease	No_EC	METe -> MET	BLASTP
AO090102000632	Amino acid permease	No_EC	METe -> MET	BLASTP
AO090103000082	Amino acid permease	No_EC	METe -> MET	BLASTP
AO090023000646	Methionine permease (High affinity)	No_EC	METe -> MET	BLASTP
AO090005000114	Amino acid permease	No_EC	PHEe -> PHE	BLASTP
AO090010000564	Amino acid permease	No_EC	PHEe -> PHE	BLASTP
AO090102000632	Amino acid permease	No_EC	PHEe -> PHE	BLASTP
AO090103000082	Amino acid permease	No_EC	PHEe -> PHE	BLASTP
AO090005000114	Amino acid permease	No_EC	PROe -> PRO	BLASTP
AO090010000564	Amino acid permease	No_EC	PROe -> PRO	BLASTP

Gene name	Enzyme name	EC number	Biochemical reaction	Annotation method
AO090102000632	Amino acid permease	No_EC	PROe -> PRO	BLASTP
AO090103000082	Amino acid permease	No_EC	PROe -> PRO	BLASTP
AO090010000119	Proline permease	No_EC	PROe -> PRO	BLASTP
AO090011000293	Proline permease	No_EC	PROe -> PRO	BLASTP
AO090011000034	Proline permease	No_EC	PROe -> PRO	BLASTP
AO090026000738	Proline permease	No_EC	PROe -> PRO	BLASTP
AO090005000114	Amino acid permease	No_EC	TRPe -> TRP	BLASTP
AO090010000564	Amino acid permease	No_EC	TRPe -> TRP	BLASTP
AO090102000632	Amino acid permease	No_EC	TRPe -> TRP	BLASTP
AO090103000082	Amino acid permease	No_EC	TRPe -> TRP	BLASTP
AO090005000114	Amino acid permease	No_EC	TYRe -> TYR	BLASTP
AO090010000564	Amino acid permease	No_EC	TYRe -> TYR	BLASTP
AO090102000632	Amino acid permease	No_EC	TYRe -> TYR	BLASTP
AO090103000082	Amino acid permease	No_EC	TYRe -> TYR	BLASTP
AO090005000114	Amino acid permease	No_EC	VALe -> VAL	BLASTP
AO090010000564	Amino acid permease	No_EC	VALe -> VAL	BLASTP
AO090102000632	Amino acid permease	No_EC	VALe -> VAL	BLASTP
AO090103000082	Amino acid permease	No_EC	VALe -> VAL	BLASTP
AO090005000114	Amino acid permease	No_EC	SERe -> SER	BLASTP
AO090010000564	Amino acid permease	No_EC	SERe -> SER	BLASTP
AO090102000632	Amino acid permease	No_EC	SERe -> SER	BLASTP
AO090103000082	Amino acid permease	No_EC	SERe -> SER	BLASTP
AO090011000116	Amino acid permease for serine, aspartate and glutamate	No_EC	SERe -> SER	BLASTP
AO090011000820	Dicarboxylic amino acid permease	No_EC	SERe -> SER	BLASTP
AO090005000114	Amino acid permease	No_EC	THRe -> THR	BLASTP
AO090010000564	Amino acid permease	No_EC	THRe -> THR	BLASTP
AO090102000632	Amino acid permease	No_EC	THRe -> THR	BLASTP
AO090103000082	Amino acid permease	No_EC	THRe -> THR	BLASTP
AO090005000114	Amino acid permease	No_EC	LYSe -> LYS	BLASTP
AO090010000564	Amino acid permease	No_EC	LYSe -> LYS	BLASTP

Gene name	Enzyme name	EC number	Biochemical reaction	Annotation method
AO090102000632	Amino acid permease	No_EC	LYSe -> LYS	BLASTP
AO090103000082	Amino acid permease	No_EC	LYSe -> LYS	BLASTP
AO090701000858	Lysine specific permease (High affinity)	No_EC	LYSe -> LYS	BLASTP
AO090011000204	Gaba specific permease	No_EC	GABAe -> GABA	BLASTP
AO090009000635	Gaba specific permease	No_EC	GABAe -> GABA	BLASTP
AO090009000552	Choline transport protein	No_EC	CHOe -> CHO	BLASTP
AO090102000493	Choline transport protein	No_EC	CHOe -> CHO	BLASTP
**# Nucleotides**				
AO090012000565	Uracil permease	No_EC	URAe -> URA	BLASTP
AO090005000019	Uracil permease	No_EC	URAe -> URA	BLASTP
AO090011000649	Purine-cytosine permease	No_EC	CYTSe -> CYTS	BLASTP
AO090011000649	Purine-cytosine permease	No_EC	ADe -> AD	BLASTP
AO090011000649	Purine-cytosine permease	No_EC	GNe -> GN	BLASTP
**# Other compounds**				
PMT_CO2	CO2 plasma membrane transport	No_EC	CO2e <-> CO2	NONE
PMT_H2O	H2O plasma membrane transport	No_EC	H2Oe <-> H2O	NONE
PMT_O2	O2 plasma membrane transport	No_EC	O2e -> O2	NONE
PMT_H3PO4	H3PO4 plasma membrane transport	No_EC	PIe -> PI	NONE
PMT_H2SO3	H2SO3 plasma membrane transport	No_EC	H2SO3e -> H2SO3	NONE
AO090003000798	Sulfate transporter	No_EC	SLFe -> SLF	BLASTP
AO090009000456	Sulfate transporter	No_EC	SLFe -> SLF	BLASTP
AO090038000314	Ammonium transporter	No_EC	NH3e -> NH3	BLASTP
AO090001000707	Ammonium transporter	No_EC	NH3e -> NH3	BLASTP
AO090026000749	Ammonium transporter	No_EC	NH3e -> NH3	BLASTP
AO090012000623	Nitrate permease	No_EC	HNO3e -> HNO3	BLASTP
AO090102000343	Potassium ion transporter	No_EC	Ke + He <-> K	BLASTP
AO090011000734	Potassium ion transporter	No_EC	Ke + He <-> K	BLASTP
AO090102000102	Na+/H+ exchanger	No_EC	Nae <-> Na + He	BLASTP
**# Alcohols and acids**				
PMT_ETH	Ethanol plasma membrane transport	No_EC	ETHe <-> ETH	NONE
PMT_METHOL	Methanol plasma membrane transport	No_EC	METHOLe <-> METHOL	NONE

Gene name	Enzyme name	EC number	Biochemical reaction	Annotation method
PMT_AC	Acetate plasma membrane transport	No_EC	ACe <-> AC	NONE
PMT_FOR	Formate plasma membrane transport	No_EC	FORe <-> FOR	NONE
PMT_LLAC	L-Lactate plasma membrane transport	No_EC	LLACe <-> LLAC	NONE
PMT_LAC	Lactate plasma membrane transport	No_EC	LACe <-> LAC	NONE
PMT_PYR	Pyruvate plasma membrane transport	No_EC	PYRe <-> PYR	NONE
PMT_SUCC	Succinate plasma membrane transport	No_EC	SUCCe <-> SUCC	NONE
PMT_MAL	Malate plasma membrane transport	No_EC	MALe <-> MAL	NONE
PMT_AKG	2-oxoglutarate plasma membrane transport	No_EC	AKGe <-> AKG	NONE
PMT_CIT	Citrate plasma membrane transport	No_EC	CITe <-> CIT	NONE
PMT_FUM	Fumarate plasma membrane transport	No_EC	FUMe <-> FUM	NONE
PMT_ICIT	Isocitrate plasma membrane transport	No_EC	ICITe <-> ICIT	NONE
PMT_OXAL	Oxalate plasma membrane transport	No_EC	OXALe <-> OXAL	NONE
PMT_OA	Oxaloacetate plasma membrane transport	No_EC	OAe <-> OA	NONE
**# Mitochondria membrane transport**				
MMT_CO2	CO2 mitochondrial membrane transport	No_EC	CO2 <-> CO2m	NONE
MMT_H2O	H2O mitochondrial membrane transport	No_EC	H2O <-> H2Om	NONE
MMT_O2	O2 mitochondrial membrane transport	No_EC	O2 <-> O2m	NONE
AO090005001192	Mitochondrial phosphate carrier	No_EC	PI <-> PIm	BLASTP
AO090120000053	Mitochondrial phosphate carrier	No_EC	PI <-> PIm	BLASTP
AO090005001430	Mitochondrial phosphate carrier	No_EC	PI <-> PIm	BLASTP
AO090026000437	Mitochondrial phosphate carrier	No_EC	PI <-> PIm	BLASTP
MMT_NH3	NH3 mitochondrial membrane transport	No_EC	NH3 <-> NH3m	NONE
MMT_ORN	Mitochondrial ornithine carrier	No_EC	ORN <-> ORNm	NONE
MMT_PA	Phosphatidate mitochondrial membrane transport	No_EC	PA -> PAm	NONE
MMT_OIVAL	Mitochondrial 2-Oxoisovalerate carrier	No_EC	OIVAL <-> OIVALm	NONE
MMT_IPPMAL	Mitochondrial 2-Isopropylmalate carrier	No_EC	IPPMAL <-> IPPMALm	NONE
MMT_OMVAL	Mitochondrial 2-keto-3-methyl-valerate carrier	No_EC	OMVAL <-> OMVALm	NONE
MMT_THR	Mitochondrial L-Threonine carrier	No_EC	THRm <-> THR	NONE
MMT_OICAP	3-Carboxy-4-methyl-2-oxopentanoate mitochondrial membrane transport	No_EC	OICAPm <-> OICAP	NONE

Gene name	Enzyme name	EC number	Biochemical reaction	Annotation method
MMT_CAP	Carbamoyl phosphate mitochondrial membrane transport	No_EC	CAPm <-> CAP	NONE
MMT_KYN	L-Kynurenine mitochondrial membrane transport	No_EC	KYNm <-> KYN	NONE
MMT_HKYN	3-Hydroxykynurenine mitochondrial membrane transport	No_EC	HKYNm <-> HKYN	NONE
MMT_DOROA	Dihydroorotate mitochondrial membrane transport	No_EC	DOROAm <-> DOROA	NONE
MMT_OROA	Orotate mitochondrial membrane transport	No_EC	OROAm <-> OROA	NONE
MMT_THF	Tetrahydrofolate mitochondrial membrane transport	No_EC	THF <-> THFm	
MMT_FTHF	Formyltetrahydrofolate mitochondrial membrane transport	No_EC	FTHF <-> FTHFm	
MMT_METTHF	5,10-Methylenetetrahydrofolate mitochondrial membrane transport	No_EC	METTHF <-> METTHFm	NONE
MMT_METHF	5,10-Methenyltetrahydrofolate mitochondrial membrane transport	No_EC	METHFm <-> METHF	NONE
MMT_ETH	Ethanol mitochondrial membrane transport	No_EC	ETH <-> ETHm	NONE
MMT_AC	Acetate mitochondrial membrane transport	No_EC	AC <-> ACm	NONE
MMT_ACAL	Acetaldehyde mitochondrial membrane transport	No_EC	ACAL <-> ACALm	NONE
MMT_FOR	Formate mitochondrial membrane transport	No_EC	FOR <-> FORm	NONE
MMT_PYR	Mitochondrial pyruvate carrier	No_EC	PYR -> PYRm	NONE
MMT_ICITMAL	Isocitrate/malate mitochondrial membrane transport	No_EC	ICITm + MAL -> ICIT + MALm	NONE
MMT_CITMAL	Citrate/malate mitochondrial membrane transport	No_EC	CITm + MAL -> CIT + MALm	NONE
MMT_AKG	2-oxoglutarate mitochondrial membrane transport	No_EC	AKGm <-> AKG	NONE
MMT_SUCC	Succinate mitochondrial membrane transport	No_EC	SUCCm <-> SUCC	NONE
MMT_FUM	Fumarate mitochondrial membrane transport	No_EC	FUMm <-> FUM	NONE
MMT_MAL	Malate mitochondrial membrane transport	No_EC	MALm <-> MAL	NONE
MMT_CIT	Citrate mitochondrial membrane transport	No_EC	CITm <-> CIT	NONE
MMT_ICIT	Isocitrate mitochondrial membrane transport	No_EC	ICITm <-> ICIT	NONE

Gene name	Enzyme name	EC number	Biochemical reaction	Annotation method
AO090102000125	Succinate/fumarate antiporter	No_EC	SUCC + FUMm -> SUCCm + FUM	BLASTP
AO090120000354	Mitochondrial oxaloacetate carrier	No_EC	OA <-> OAm	BLASTP
AO090026000334	Dicarboxylate carrier	No_EC	SUCC + PIm -> SUCCm + PI	BLASTP
AO090026000334	Dicarboxylate carrier	No_EC	MAL + SUCCm <-> MALm + SUCC	BLASTP
AO090026000334	Dicarboxylate carrier	No_EC	MAL + PIm <-> MALm + PI	BLASTP
AO090020000012	Citrate transport protein	No_EC	CIT + MALm <-> CITm + MAL	BLASTP
AO090020000012	Citrate transport protein	No_EC	CIT + ICITm <-> CITm + ICIT	BLASTP
AO090020000012	Citrate transport protein	No_EC	CIT + PEPm <-> CITm + PEP	BLASTP
MMT_GLU	Glutamate carrier	No_EC	GLU <-> GLUm	NONE
AO090009000405	ADP/ATP carrier protein (MCF)	No_EC	ADP + ATPm + PI -> Hm + ADPm + ATP + PIm	BLASTP
MMT_SAM	S-Adenosyl-L-methionine mitochondrial membrane transport	No_EC	SAM <-> SAMm	NONE
MMT_SAH	S-Adenosyl-L-homocysteine mitochondrial membrane transport	No_EC	SAH <-> SAHm	NONE
MMT_GLY	Glycine mitochondrial membrane transport	No_EC	GLYm <-> GLY	NONE
MMT_SER	Serine mitochondrial membrane transport	No_EC	SERm <-> SER	NONE
MMT_ILE	Isoleucine mitochondrial membrane transport	No_EC	ILEm <-> ILE	NONE
MMT_VAL	Valine mitochondrial membrane transport	No_EC	VALm <-> VAL	NONE
MMT_LEU	Leucine mitochondrial membrane transport	No_EC	LEUm <-> LEU	NONE
MMT_CITR	Citrulline mitochondrial membrane transport	No_EC	CITRm <-> CITR	NONE
MMT_FAD	FAD transport protein	No_EC	FAD + FMNm -> FADm + FMN	NONE
MMT_H3MCOA	(S)-3-Hydroxy-3-methylglutaryl-CoA mitochondrial membrane transport	No_EC	H3MCOA <-> H3MCOAm	NONE
MMT_DHF	Dihydrofolate mitochondrial membrane transport	No_EC	DHF <-> DHFm	NONE
MMT_PROP	Propanoate mitochondria membrane transporter	No_EC	PROP <-> PROPm	NONE
**# Shuttle**				
**# Glycerol phosphate shuttle**				
SHUTM_GL3P	Glycerol phosphate shuttle	No_EC	GL3P -> GL3Pm	NONE
**# Malate-Aspartate shuttle**				
SHUTM_ASPGLU	Aspartate shuttle	No_EC	ASPm + GLU <-> ASP + GLUm	NONE
SHUTM_MALAKG	Malate shuttle	No_EC	MAL + AKGm <-> MALm + AKG	NONE

Gene name	Enzyme name	EC number	Biochemical reaction	Annotation method
**# Carnitine shuttle**				
AO090103000383	Carnitine carrier	No_EC	CARm + ACAR -> CAR + ACARm	NONE
**# Peroxisomal membrane transport**				
PxMT_CO2	CO2 peroxisomal membrane transport	No_EC	CO2p <-> CO2	NONE
PxMT_H2O	H2O peroxisomal membrane transport	No_EC	H2Op <-> H2O	NONE
PxMT_O2	O2 peroxisomal membrane transport	No_EC	O2p <-> O2	NONE
PxMT_ICITMAL	Isocitrate/malate peroxisomal membrane transport	No_EC	ICIT + MALp -> ICITp + MAL	NONE
PxMT_SUCC	Succinate peroxisomal membrane transport	No_EC	SUCCp <-> SUCC	NONE
AO090005000361	Malic acid transport protein	No_EC	MALp -> MAL	BLASTP
PxMT_HIURTE	3-hydroxy-isobutyrate peroxisomal membrane transport	No_EC	HIURTEp <-> HIURTE	NONE
PxMT_AKG	2-oxoglutarate peroxisomal membrane transport	No_EC	AKGp -> AKG	NONE
AO090003000496	Allantoate transporter	No_EC	ATTp <-> ATT	BLASTP
AO090009000507	Allantoate transporter	No_EC	ATTp <-> ATT	BLASTP
AO090003000854	Urea active transporter	No_EC	UREAp <-> UREA	BLASTP
AO090003001423	Urea active transporter	No_EC	UREAp <-> UREA	BLASTP
PxMT_PYR	Pyruvate peroxisomal membrane transport	No_EC	PYRp -> PYR	NONE
PxMT_PROPCOA	Propanoyl-CoA peroxisomal membrane transport	No_EC	PROPCOAp <-> PROPCOA	NONE
PxMT_GLAL	Glycoaldehyde peroxisomal membrane transport	No_EC	GLALp <-> GLAL	NONE
PxMT_OA	Oxaloacetate peroxisomal membrane transport	No_EC	OAp <-> OA	NONE
PxMT_XAN	Xanthine peroxisomal membrane transport	No_EC	XANp <-> XAN	NONE
PxMT_UGC	Ureidoglycolate peroxisomal membrane transport	No_EC	UGC -> UGCp	NONE
PxMT_H2O2	H2O2 peroxisomal membrane transport	No_EC	H2O2p <-> H2O2	NONE

Suppl. Table 2: List of the abbreviations for metabolite names used in the reaction list of *A. oryzae* (Suppl. Table 1) and their full name. An “m” denotes that the metabolite is found in the mitochondria. A “p” indicates that the metabolite is found in the peroxisome whereas an “e” is used to mark an extracellular metabolite.

Abbreviation	Full name of metabolite
1368THN	1,3,6,8-THN
138THN	1,3,8-THN
13GLUCAN	1,3-beta-D-Glucan
13GLUCANe	1,3-beta-D-Glucan (Extracellular)
13PDG	1,3-Bisphospho-D-glycerate
14MNAN	1,4 beta Mannan
18DHN	1,8-DHN
2D3DGALT	2-Dehydro-3-deoxy-D-galactonate
2MACOp	2-Methyl-cis-aconitate (Peroxisome)
2MCITp	2-Methylcitrate (Peroxisome)
2MICITp	2-Methylisocitrate (Peroxisome)
2N6H	2-octaprenyl-6-hydroxyphenol
2NMHMBm	3-demethylubiquinone-8 (Mitochondria)
2NPMBm	2-octaprenyl-6-methoxy-1,4-benzoquinone (Mitochondria)
2NPMMBm	2-octaprenyl-3-methyl-6-methoxy-1,4-benzoquinone (Mitochondria)
2NPMP	2-octaprenyl-6-methoxyphenol
2NPMPm	2-octaprenyl-6-methoxyphenol (Mitochondria)
2NPPP	2-octaprenylphenol
2PG	2-Phospho-D-glycerate
3DDAH7P	2-Dehydro-3-deoxy-D-arabino-heptonate 7-phosphate
3PG	3-Phospho-D-glycerate
3PSER	3-Phosphoserine
3PSME	5-O-(1-Carboxyvinyl)-3-phosphoshikimate
3SULALA	3-sulfinoalanine
4CMCOA	4-coumaroyl-COA
4CMRE	4-hydroxycinnamate
4HBZ	4-hydroxybenzoate
4HPP	4-Hydroxyphenyl pyruvate
4PPNCYS	4-Phosphopantothenoyl-L-cysteine
4PPNTE	Pantetheine 4'-phosphate
4PPNTO	D-4'-Phosphopantothenate
5MTA	5'-Methylthioadenosine
5OXOPRO	5-oxo-L-proline
5THR1P	S-methyl-5-thio-α-D-ribose 1-phosphate
6CARHEX	6-carboxyhexanoate
7DECHORES	Cholesta-5,7-dien-3-beta-ol
A6RP	5-amino-6-ribityluracil
A6RP5P	5-amino-6-(5-phosphoribosylamino) uracil
A6RP5P2	5-amino-6-(5-phosphoribitylamino) uracil
AACACP	3-oxoacyl-[acyl-carrier protein]
AACCOA	Acetoacetyl coenzyme A
AACCOAm	Acetoacetyl coenzyme A (Mitochondria)
AACCOAp	Acetoacetyl coenzyme A (Peroxisome)
ABUTm	2-Aceto-2-hydroxy butyrate (Mitochondria)
AC	Acetate
ACACP	Acyl-[acyl-carrier protein]
ACAL	Acetaldehyde
ACALm	Acetaldehyde (Mitochondria)

Abbreviation	Full name of metabolite
ACAR	O-Acetylcarnitine
ACARm	O-Acetylcarnitine (Mitochondria)
ACCOA	Acetyl coenzyme A
ACCOAm	Acetyl coenzyme A (Mitochondria)
ACCOAp	Acetyl coenzyme A (Peroxisome)
ACe	Acetate (Extracellular)
ACET	Acetone
ACETm	Acetone (Mitochondria)
ACLACm	2-Acetolactate (Mitochondria)
ACm	Acetate (Mitochondria)
ACOA	Acyl coenzyme A
ACOm	Cis-Aconitate (Mitochondria)
ACP	Acetate (Peroxisome)
ACPC	1-aminocyclopropane-1-carboxylate
ACPm	Acyl-carrier protein (Mitochondria)
ACTAC	Acetoacetate
ACTACm	Acetoacetate (Mitochondria)
ACTP	Acetyl phosphate
ACTPm	Acetyl phosphate (Mitochondria)
ACYBUT	Gamma-Amino-gamma-cyanobutanoate
AD	Adenine
ADCHOR	4-Amino-4-deoxychorismate
ADe	Adenine (Extracellular)
ADN	Adenosine
ADP	ADP
ADPm	ADP (Mitochondria)
ADPRIB	ADP-ribose
ADPRIBm	ADP-ribose (Mitochondria)
AFB1	Aflatoxin B1
AFB2	Aflatoxin B2
AFG1	Aflatoxin G1
AFG2	Aflatoxin G2
AGL3P	Acyl-sn-glycerol 3-phosphate
AGMT	Agmatine
AHHMD	2-Amino-7,8-dihydro-4-hydroxy-6-(diphosphooxymethyl)pteridine
AHHMP	2-Amino-4-hydroxy-6-hydroxymethyl-7,8-dihydropteridine
AHM	4-amino-5-hydroxymethyl-2-methylpyrimidine
AHMP	4-amino-5-phosphomethyl-2-methylpyrimidine
AHMPP	4-amino-2-methyl-5-diphosphomethylpyrimidine
AHTD	2-Amino-4-hydroxy-6-(erythro-1,2,3-trihydroxypropyl)-dihydropteridine triphosphate
AICAR	AICAR
AIR	Aminoimidazole ribotide
AKA	2-Oxoadipate
AKG	2-Oxoglutarate
AKGe	2-Oxoglutarate (Extracellular)
AKGm	2-Oxoglutarate (Mitochondria)
AKGp	2-Oxoglutarate (Peroxisome)
AKP	2-Dehydropantoate
AKPm	2-Dehydropantoate (Mitochondria)
ALA	L-Alanine
ALAe	L-Alanine (Extracellular)

Abbreviation	Full name of metabolite
ALAGLY	R-S-Alanylglycine
ALAm	L-Alanine (Mitochondria)
ALTRNA	L-Arginyl-tRNA
AM6SA	2-Aminomuconate 6-semialdehyde
AMA	L-2-Aminoadipate
AMAC	Aminoacetone
AMACm	Aminoacetone (Mitochondria)
AMASA	L-2-Aminoadipate 6-semialdehyde
AMIACE	Aminoacetaldehyde
AMIEVUL	5-aminolevulinate
AMIEVULm	5-aminolevulinate (Mitochondria)
AMOXOBU	2-amino-3oxobutanoate
AMP	AMP
AMPm	AMP (Mitochondria)
AMPp	AMP (Peroxisome)
AMUCO	2-Aminomuconate
AN	Anthranilate
AOL	D-Arabitol
AOLe	D-Arabitol (Extracellular)
AONA	8-Amino-7-oxononanoate
AONAm	8-Amino-7-oxononanoate (Mitochondria)
APEBU	4-(2-aminophenyl)-2,4-dioxobutanoate
APOCEm	Apocytochrome C (Mitochondria)
APROA	3-Aminopropanal
APROP	Alpha-amino-propiononitrile
APRUT	N-Acetylputrescine
APS	Adenylylsulfate
ARAB	D-Arabinose
ARABe	D-Arabinose (Extracellular)
ARABINe	Arabinan (Extracellular)
ARABLAC	D-Arabinono-1,4-lactone
ARG	L-Arginine
ARGe	L-Arginine (Extracellular)
ARGSUCC	N-(L-Arginino)succinate
ASER	O-Acetyl-L-serine
ASERm	O-Acetyl-L-serine (Mitochondria)
ASN	L-Asparagine
ASNe	L-Asparagine (Extracellular)
ASNm	L-Asparagine (Mitochondria)
ASNTRNA	L-Asparaginyl-tRNA
ASNTRNAm	L-Asparaginyl-tRNA (Mitochondria)
ASP	L-Aspartate
ASPe	L-Aspartate (Extracellular)
ASPERMD	N1-Acetylspermidine
ASPm	L-Aspartate (Mitochondria)
ASPSA	L-Aspartate 4-semialdehyde
ASPTRNA	L-Aspartyl-tRNA
ASPTRNAm	L-Aspartyl-tRNA (Mitochondria)
ASUC	N6-(1,2-Dicarboxyethyl)-AMP
AT3P2	Acyldihydroxyacetone phosphate
ATN	Allantoin
ATP	ATP
ATPm	ATP (Mitochondria)

Abbreviation	Full name of metabolite
ATPp	ATP (Peroxisome)
ATT	Allantoate
ATTp	Allantoate (Peroxisome)
AVF	Averufin
AVN	Averantin
bALA	Beta-alanine
BASP	4-Phospho-L-aspartate
BCCP	Biotin carboxyl-carrier protein
bDG6P	Beta-D-Glucose 6-phosphate
bDGLC	Beta-D-Glucose
bDGLCe	Beta-D-Glucose (Extracellular)
BETALD	Betaine aldehyde
BT	Biotin
BTAMP	Biotinyl-5-AMP
C100	Decanoic acid
C100ACP	Decanoyl-[Acyl carrier protein]
C100COA	Decanoyl-Coenzyme A
C100COAm	Decanoyl-Coenzyme A (Mitochondria)
C10DACP	Decanoyl-dehydro-[acyl-carrier protein]
C10DCOAm	Decanoyl-dehydro-Coenzyme A (Mitochondria)
C10HACP	Decanoyl-Hydroxy-[acyl-carrier protein]
C10HCOAm	Decanoyl-Hydroxy-Coenzyme A (Mitochondria)
C10OACP	Decanoyl-oxo-[acyl-carrier protein]
C10OCOAm	Decanoyl-oxo-Coenzyme A (Mitochondria)
C120	Dodecanoic acid
C120ACP	Dodecanoyl-[acyl-carrier protein]
C120COA	Dodecanoyl-Coenzyme A
C120COAm	Dodecanoyl-Coenzyme A (Mitochondria)
C12DACP	Dodecanoyl-dehydro-[acyl-carrier protein]
C12DCOAm	Dodecanoyl-dehydro-Coenzyme A (Mitochondria)
C12HACP	Dodecanoyl-Hydroxy-[acyl-carrier protein]
C12HCOAm	Dodecanoyl-Hydroxy-Coenzyme A (Mitochondria)
C12OACP	Dodecanoyl-oxo-[acyl-carrier protein]
C12OCOAm	Dodecanoyl-oxo-Coenzyme A (Mitochondria)
C140	Myristic acid
C140ACP	Myristoyl-[acyl-carrier protein]
C140COA	Myristoyl-Coenzyme A
C140COAm	Myristoyl-Coenzyme A (Mitochondria)
C14DACP	Myristoyl-dehydro-[acyl-carrier protein]
C14DCOAm	Myristoyl-dehydro-Coenzyme A (Mitochondria)
C14HACP	Myristoyl-Hydroxy-[acyl-carrier protein]
C14HCOAm	Myristoyl-Hydroxy-Coenzyme A (Mitochondria)
C14OACP	Myristoyl-oxo-[acyl-carrier protein]
C14OCOAm	Myristoyl-oxo-Coenzyme A (Mitochondria)
C160	Palmitate
C160ACP	Hexadecanoyl-[acyl-carrier protein]
C160COA	Hexadecanoyl-Coenzyme A
C160COAm	Hexadecanoyl-Coenzyme A (Mitochondria)
C161	Palmitoic acid
C161ACP	Palmitoyl-[acyl-carrier protein]
C161COA	Palmitoyl-Coenzyme A
C162	Hexadecadienoic acid
C162ACP	Hexadecadienoic acid-[acyl-carrier protein]

Abbreviation	Full name of metabolite
C162COA	Hexadecadienoyl-Coenzyme A
C16A	Palmitate_aldehydes
C16DACP	Hexadecanoyl-dehydro-[acyl-carrier protein]
C16DCOAm	Hexadecanoyl-dehydro-Coenzyme A (Mitochondria)
C16HACP	Hexadecanoyl-Hydroxy-[acyl-carrier protein]
C16HCOAm	Hexadecanoyl-Hydroxy-Coenzyme A (Mitochondria)
C16OACP	Hexadecanoyl-oxo-[acyl-carrier protein]
C16OCOAm	Hexadecanoyl-oxo-Coenzyme A (Mitochondria)
C180	Stearate
C180ACP	Stearoyl-[acyl-carrier protein]
C180COA	Stearoyl-Coenzyme A
C180COAm	Stearoyl-Coenzyme A (Mitochondria)
C181	Oleic acid
C181ACP	Oleoyl-[acyl-carrier protein]
C181COA	Oleoyl-Coenzyme A
C182	Linoleic acid
C182ACP	Linolenoyl-[acyl-carrier protein]
C182COA	Linolenoyl-Coenzyme A
C183	Octadecatrienoic acid
C183ACP	Octadecatrienoic acid-[acyl-carrier protein]
C183COA	Octadecatrienoic acid-Coenzyme A
C18DACP	Stearoyl-dehydro-[acyl-carrier protein]
C18DCOAm	Stearoyl-dehydro-Coenzyme A (Mitochondria)
C18HACP	Stearoyl-Hydroxy-[acyl-carrier protein]
C18HCOAm	Stearoyl-Hydroxy-Coenzyme A (Mitochondria)
C18OACP	Stearoyl-oxo-[acyl-carrier protein]
C18OCOAm	Stearoyl-oxo-Coenzyme A (Mitochondria)
C40	Butyric acid
C40ACP	Butyryl-[acyl-carrier protein]
C40COA	Butyryl-Coenzyme A
C40COAm	Butyryl-Coenzyme A (Mitochondria)
C4DACP	Butyryl-dehydro-[acyl-carrier protein]
C4DCOAm	Butyryl-dehydro-Coenzyme A (Mitochondria)
C4HACP	Butyryl-Hydroxy-[acyl-carrier protein]
C4HCOA	Butyryl-Hydroxy-Coenzyme A
C4HCOAm	Butyryl-Hydroxy-Coenzyme A (Mitochondria)
C60	Hexanoic acid
C60ACP	Hexanoyl-[acyl-carrier protein]
C60COA	Hexanoyl-Coenzyme A
C60COAm	Hexanoyl-Coenzyme A (Mitochondria)
C6DACP	Hexanoyl-dehydro-[acyl-carrier protein]
C6DCOAm	Hexanoyl-dehydro-Coenzyme A (Mitochondria)
C6HACP	Hexanoyl-Hydroxy-[acyl-carrier protein]
C6HCOAm	Hexanoyl-Hydroxy-Coenzyme A (Mitochondria)
C6OACP	Hexanoyl-oxo-[acyl-carrier protein]
C6OCOAm	Hexanoyl-oxo-Coenzyme A (Mitochondria)
C80	Octanoic acid
C80ACP	Octanoyl-[acyl-carrier protein]
C80COA	Octanoyl-Coenzyme A
C80COAm	Octanoyl-Coenzyme A (Mitochondria)
C8DACP	Octanoyl-dehydro-[acyl-carrier protein]
C8DCOAm	Octanoyl-dehydro-Coenzyme A (Mitochondria)
C8HACP	Octanoyl-Hydroxy-[acyl-carrier protein]

Abbreviation	Full name of metabolite
C8HCOAm	Octanoyl-Hydroxy-Coenzyme A (Mitochondria)
C8OACP	Octanoyl-oxo-[acyl-carrier protein]
C8OCOAm	Octanoyl-oxo-Coenzyme A (Mitochondria)
Ca	Calcium
CAASP	N-Carbamoyl-L-aspartate
CABM	Carbamate
CAIR	1-(5-Phospho-D-ribosyl)-5-amino-4-imidazolecarboxylate
CALH	2-(3-Carboxy-3-aminopropyl)-L-histidine
Cam	Calcium (Mitochondria)
cAMP	3',5'-Cyclic AMP
CAP	Carbamoyl phosphate
CAPm	Carbamoyl phosphate (Mitochondria)
CAR	Carnitine
CARBO	carboxylate
CARm	Carnitine (Mitochondria)
CB15LCT	Cellobiono-1,5-lactone
CB15LCTe	Cellobiono-1,5-lactone (Extracellular)
CBCCP	Carboxybiotin-carboxyl-carrier-protein
CBHCAP	3-Isopropylmalate
cCMP	3',5'-Cyclic CMP
cdAMP	3',5'-Cyclic dAMP
CDP	CDP
CDPCHO	CDPcholine
CDPDG	CDPdiacylglycerol
CDPDGm	CDPdiacylglycerol (Mitochondria)
CDPETN	CDPethanolamine
CELLOB	Cellobiose
CELLOBe	Cellobiose (Extracellular)
CELLOTe	Cellotriose (Extracellular)
CELLUe	Cellulose (Extracellular)
CER1	Ceramide
CER2	Dihydroceramide
CER3	Phytoceramide
CGLY	Cys-Gly
cGMP	3',5'-Cyclic GMP
CHCOA	6-Carboxyhexanoyl-CoA
CHIB	Chitobiose
CHIBe	Chitobiose (Extracellular)
CHIT	Chitin
CHITe	Chitin (Extracellular)
CHITO	Chitosan
CHITOe	Chitosan (Extracellular)
CHO	Choline
CHOe	Choline (Extracellular)
CHOR	Chorismate
CHOREOL	Cholesterol
CHOREOLESTR	Cholesterol Ester
CHORESTA	Cholesta-7,24-dien-3β-ol
cIMP	3',5'-Cyclic IMP
CINNAM	Cinnamate
CIT	Citrate
CITe	Citrate (Extracellular)
CITm	Citrate (Mitochondria)

Abbreviation	Full name of metabolite
CITR	L-Citrulline
CITRm	L-Citrulline (Mitochondria)
CLm	Cardiolipin (Mitochondria)
CML	Citramalate
CMP	CMP
CMPm	CMP (Mitochondria)
CMUSA	2-Amino-3-carboxymuconate semialdehyde
CO2	Carbon dioxide
CO2e	Carbon dioxide (Extracellular)
CO2m	Carbon dioxide (Mitochondria)
CO2p	Carbon dioxide (Peroxisome)
COA	Coenzyme A
COAm	Coenzyme A (Mitochondria)
COAp	Coenzyme A (Peroxisome)
CPAD5P	1-(2-Carboxyphenylamino)-1-deoxy-D-ribulose 5-phosphate
CPGIII	Coproporphyrinogen III
CPGIIIm	Coproporphyrinogen III (Mitochondria)
CRONYLCOA	crotonyl coA
CRONYLCOAm	Crotonyl CoA (Mitochondria)
CTP	CTP
CTPm	CTP (Mitochondria)
Cu	Copper
Cum	Copper (Mitochondria)
CYMECm	Cytochrome C (Mitochondria)
CYNE	Cyanate
CYS	L-Cysteine
CYSE	L-Cysteate
CYSm	L-Cysteine (Mitochondria)
CYST	L-Cystine
CYTD	Cytidine
CYTS	Cytosine
CYTSe	Cytosine (Extracellular)
D45PI	1-Phosphatidyl-D-myo-inositol 4,5-bisphosphate
D6PGC	6-Phospho-D-gluconate
D6PGL	D-Glucono-1,5-lactone 6-phosphate
D6RP5P	2,5-diamino-6-hydroxy-4-(5-phosphoribosylamino)pyrimidine
D8RL	6,7-dimethyl-8-(1-D-ribityl)lumazine
DA	Deoxyadenosine
DADP	dADP
DAGLY	Diacylglycerol
DALA	D-Alanine
DAMP	dAMP
DANNA	7,8-diaminononanoate
DANNAm	7,8-diaminononanoate (Mitochondria)
DAPRP	1,3-Diaminopropane
DASP	D-aspartate
DATP	dATP
DB4P	3,4 dihydroxy-2-butanone-4-P
DC	Deoxycytidine
DCDP	dCDP
DCMP	dCMP
DCTP	dCTP
DEHXG	3-dehydro-2-deoxy-D-gluconate

Abbreviation	Full name of metabolite
DEORIPI	2-deoxy-D-ribose 5-phosphate
DEXG	2-deoxy-D-gluconate
DG	Deoxyguanosine
DGDG	Digalactosyl diglyceride
DGDP	dGDP
DGLC	D-Glucose
DGLCe	D-Glucose (Extracellular)
DGLYCODEX	Debranched glycogen phosphorylase-limited dextrin
DGMP	dGMP
DGPP	Diacylglycerol pyrophosphate
DGTP	dGTP
DHDMST	Dihydrodemethylsterigmatocystin
DHF	Dihydrofolate
DHFm	Dihydrofolate (Mitochondria)
DHMVAm	(R)-2,3-dihydroxy-3-methylbutanoate (Mitochondria)
DHOMST	Dihydro-O-methylsterigmatocystin
DHP	2-Amino-4-hydroxy-6-(D-erythro-1,2,3-trihydroxypropyl)-7,8-dihydropteridine
DHPT	Dihydropteroate
DHSK	3-Dehydroshikimate
DHSP	Sphinganine 1-phosphate
DHSPH	3-Dehydrosphinganine
DHST	Dihydrosterigmatocystin
DHVALm	(R)-3-Hydroxy-3-methyl-2-oxobutanoate (Mitochondria)
DIDIPC	(S)-dihydrodipicolinate
DIHINDOLE	Dihydroxyindole
DIHURA	5,6-dihydrouracil
DIMEGLY	N,N-dimethylglycine
DIMEGLYm	N,N-dimethylglycine (Mitochondria)
DIMGP	D-erythro-1-(Imidazol-4-yl)glycerol 3-phosphate
DIN	Deoxyinosine
DMNAD	Deamido-NAD
DMPP	Dimethylallyl diphosphate
DMST	Demethylsterigmatocystin
DMZYMST	4,4-Dimethylzymosterol
DOPA	L-Dopa
DOPAe	L-Dopa (Extracellular)
DOQUI	Dopaquinone
DOQUIe	Dopaquinone (Extracellular)
DOROA	(S)-Dihydroorotate
DOROAm	(S)-Dihydroorotate (Mitochondria)
DPCOA	Dephospho-CoA
DPRO	D-proline
DPTH	2-[3-Carboxy-3-(methylammonio)propyl]-L-histidine
DQT	3-Dehydroquinate
DR1P	Deoxy-ribose 1-phosphate
DSAM	S-Adenosylmethioninamine
DT	Thymidine
DTB	Dethiobiotin
DTDP	dTDP
DTMP	dTMP
DTMPm	dTMP (Mitochondria)

Abbreviation	Full name of metabolite
DTTP	dTTP
DU	Deoxyuridine
DUDP	dUDP
DUMP	dUMP
DUMPm	dUMP (Mitochondria)
DUTP	dUTP
E4P	D-Erythrose 4-phosphate
ECYSm	[Enzyme]-cysteine (Mitochondria)
EPST	Episterol
ERGOSE	Sterol ester
ERGOST	Ergosterol
ERIDICOL	Eriodictyol
ERTEOL	Ergosta-5,7,22,24(28)-tetraenol
ERTROL	Ergosta-5,7,24(28)-trienol
ESULFCYSm	[Enzyme]-S-sulfanylcysteine (Mitochondria)
ETH	Ethanol
ETHe	Ethanol (Extracellular)
ETHm	Ethanol (Mitochondria)
F26P	D-Fructose 2,6-bisphosphate
F6P	Beta-D-Fructose 6-phosphate
FAD	FAD
FADH2m	FADH2 (Mitochondria)
FADm	FAD (Mitochondria)
FALD	Formaldehyde
FALDm	Formaldehyde (Mitochondria)
FDP	Beta-D-Fructose 1,6-bisphosphate
FERIm	Ferricytochrome C (Mitochondria)
FEROm	Ferrocytochrome C (Mitochondria)
FERRIN	Ferreirin
FEST	Fecosterol
FGAM	2-(Formamido)-N1-(5'-phosphoribosyl)acetamidine
FGAR	5'-Phosphoribosyl-N-formylglycinamide
FGT	S-Formylglutathione
FKYN	L-Formylkynurenine
FMN	FMN
FMNe	FMN (Extracellular)
FMNm	FMN (Mitochondria)
FOFMETm	N-formylmethionyl-tRNAfMet (Mitochondria)
FOR	Formate
FORe	Formate (Extracellular)
FORGLU	N-formimidoyl-L-glutamate
FORm	Formate (Mitochondria)
FORMIE	Formamide
FPP	Trans,trans-Farnesyl diphosphate
FRU	D-Fructose
FRUe	D-Fructose (Extracellular)
FRUTN	D-fructuronate
FTHF	10-Formyltetrahydrofolate
FTHFm	10-Formyltetrahydrofolate (Mitochondria)
FUACAC	4-Fumarylacetoacetate
FUM	Fumarate
FUMe	Fumarate (Extracellular)
FUMm	Fumarate (Mitochondria)

Abbreviation	Full name of metabolite
G	D-Glycerate
G1P	Alpha-D-Glucose 1-phosphate
G6P	Alpha-D-Glucose 6-phosphate
GA6P	D-Glucosamine 6-phosphate
GABA	4-Aminobutanoate
GABAe	4-Aminobutanoate (Extracellular)
GABAL	4-Aminobutyraldehyde
GABALm	4-Aminobutyraldehyde (Mitochondria)
GABAm	4-Aminobutanoate (Mitochondria)
GACER	Galactocyl ceremide
GAL1P	Alpha-D-Galactose 1-phosphate
GALN14LAC	D-Galactono-1,4-lactone
GALNT	D-Galactonate
GALOL	Galactitol
GALUNT	D-Galacturonate
GALUNTe	D-Galacturonate (Extracellular)
GAR	5'-Phosphoribosylglycinamide
GC	Gamma-L-Glutamyl-L-cysteine
GCYLCR	D-glucosyl-ceramide
GDP	GDP
GDPm	GDP (Mitochondria)
GDPMAN	GDPmannose
GGPP	Geranylgeranyl diphosphate
GL	Glycerol
GL3P	Sn-Glycerol 3-phosphate
GL3Pm	Sn-Glycerol 3-phosphate (Mitochondria)
GLAC	D-Galactose
GLACe	D-Galactose (Extracellular)
GLAL	Glycoaldehyde
GLALp	Glycoaldehyde (Peroxisome)
GLC	Alpha-D-Glucose
GLCe	Alpha-D-Glucose (Extracellular)
GLCN	D-Glucosamine
GLCN15LAC	D-Glucono-1,5-lactone
GLCN15LACe	D-Glucono-1,5-lactone (Extracellular)
GLCNe	D-Glucosamine (Extracellular)
GLCNT	D-Gluconate
GLCNTe	D-Gluconate (Extracellular)
GLe	Glycerol (Extracellular)
GLN	L-Glutamine
GLNe	L-Glutamine (Extracellular)
GLNm	L-Glutamine (Mitochondria)
GLTCOA	Glutaryl CoA
GLU	L-Glutamate
GLU1SAL	Glutamate-1-semialdehyde
GLUCN	D-glucuronate
GLUCRE	D-glucurononate
GLUe	L-Glutamate (Extracellular)
GLUGSAL	L-Glutamate 5-semialdehyde
GLUGSALm	L-Glutamate 5-semialdehyde (Mitochondria)
GLUm	L-Glutamate (Mitochondria)
GLUP	Alpha-D-Glutamyl phosphate
GLX	Glyoxylate

Abbreviation	Full name of metabolite
GLXm	Glyoxylate (Mitochondria)
GLXp	Glyoxylate (Peroxisome)
GLY	Glycine
GLYAL	D-Glyceraldehyde
GLYBET	Glycine betaine
GLYCEROCHO	Glycerophosphocholine
GLYCODEX	Glycogen phosphorylase-limited dextrin
GLYCOGEN	Glycogen
GLYCOGENe	Glycogen (Extracellular)
GLYCOLAp	Glycolate (Peroxisome)
GLYe	Glycine (Extracellular)
GLYm	Glycine (Mitochondria)
GLYN	Glycerone
GLYNIN	Glycogenin
GLYTRNA	Glycyl-tRNA
GMP	GMP
GN	Guanine
GNe	Guanine (Extracellular)
Gp	D-Glycerate (Peroxisome)
GPP	Geranyl diphosphate
GSN	Guanosine
GTP	GTP
GTPm	GTP (Mitochondria)
H	Hydrogen
H_PO	Proton
H_PO_m	Proton (Mitochondria)
H2O	Water
H2O2	Hydrogen peroxide
H2O2e	Hydrogen peroxide (Extracellular)
H2O2m	Hydrogen peroxide (Mitochondria)
H2O2p	Hydrogen peroxide (Peroxysome)
H2Oe	Water (Extracellular)
H2Om	Water (Mitochondria)
H2Op	Water (Peroxisome)
H2S	Hydrogen sulfide
H2Sm	Hydrogen sulfide (Mitochondria)
H2SO3	Sulfite
H2SO3e	Sulfite (Extracellular)
H3MCOA	(S)-3-Hydroxy-3-methylglutaryl-CoA
H3MCOAm	(S)-3-Hydroxy-3-methylglutaryl-CoA (Mitochondria)
HACN	But-1-ene-1,2,4-tricarboxylate
HACNm	But-1-ene-1,2,4-tricarboxylate (Mitochondria)
HAN	3-Hydroxyanthranilate
HAVN	5-Hydroxyaverantin
HCIT	2-Hydroxybutane-1,2,4-tricarboxylate
HCITm	2-Hydroxybutane-1,2,4-tricarboxylate (Mitochondria)
HCO3	HCO3
HCO3m	HCO3 (Mitochondria)
HCXY	Holo-carboxylase
HCYS	L-Homocysteine
He	Hydrogen (Extracellular)
HEME_Am	Heme A (Mitochondria)
HEME_Om	Heme O (Mitochondria)

Abbreviation	Full name of metabolite
HHTRNA	L-Histidyl-tRNA
HICIT	Homoisocitrate
HICITm	Homoisocitrate (Mitochondria)
HIS	L-Histidine
HISe	L-Histidine (Extracellular)
HISOL	L-Histidinol
HISOLP	L-Histidinol phosphate
HIURTE	3-hydroxy-isobutyrate
HIURTEp	3-hydroxy-isobutyrate (Peroxisome)
HKYN	3-Hydroxykynurenine
HKYNm	3-Hydroxykynurenine (Mitochondria)
Hm	Hydrogen (Mitochondria)
HMTB	Hydroxymethylbilane
HNO2	Nitrite
HNO2m	Nitrite (Mitochondria)
HNO3	Nitrate
HNO3e	Nitrate (Extracellular)
HOMOGEN	Homogentisate
HPRO	Trans-4-Hydroxy-L-proline
HPROm	Trans-4-Hydroxy-L-proline (Mitochondria)
HPYRp	Hydroxypyruvate (Peroxisome)
HSER	L-Homoserine
HX	HX
HYGTA	S-(hydroxymethyl)glutathione
HYISOCOA	3-hydroxy-isobutyryl COA
HYISOCOAm	3-hydroxy-isobutyryl COA (Mitochondria)
HYISORATEm	3-hydroxy-isobutyrate (Mitochondria)
HYXN	Hypoxanthine
IAC	Indole-3-acetate
IAD	Indole-3-acetamide
ICIT	Isocitrate
ICITe	Isocitrate (Extracellular)
ICITm	Isocitrate (Mitochondria)
ICITp	Isocitrate (Peroxisome)
IDP	IDP
IDPm	IDP (Mitochondria)
IGP	Indoleglycerol phosphate
IGST	4,4-Dimethylcholesta-8,14,24-trienol
IIMZYMST	Intermediate_Methylzymosterol_II
IIZYMST	Intermediate_Zymosterol_II
ILE	L-Isoleucine
ILEe	L-Isoleucine (Extracellular)
ILEm	L-Isoleucine (Mitochondria)
IMACP	3-(Imidazol-4-yl)-2-oxopropyl phosphate
IMIPRO	(S)-3-(5-oxo-4,5-dihydro-3H-imidazol-4-yl)propanoate
IMP	IMP
IMZYMST	Intermediate_Methylzymosterol_I
INS	Inosine
IPC	Inositol phosphorylceramide
IPN	Isopenicillin N
IPPMAL	2-Isopropylmalate
IPPMALm	2-Isopropylmalate (Mitochondria)
IPPP	Isopentenyl diphosphate

Abbreviation	Full name of metabolite
ISOBUCOA	Isobutyryl coA
ISOVACOA	Isovaleryl-CoA
ISOVACOAm	Isovaleryl-CoA (Mitochondria)
ITP	ITP
ITPm	ITP (Mitochondria)
IZYMST	Intermediate_Zymosterol_I
K	Potassium
Ke	Potassium (Extracellular)
KEMYOI	2-keto-myo-inositol
Km	Potassium (Mitochondria)
KYN	L-Kynurenine
KYNm	L-Kynurenine (Mitochondria)
LAC	D-Lactate
LACAL	D-Lactaldehyde
LACALm	(S)-Lactaldehyde (Mitochondria)
LACe	D-Lactate (Extracellular)
LACm	(R)-Lactate (Mitochondria)
LACT	Lactose
LACTe	Lactose (Extracellular)
LAlaTRNA	L-alanyl-tRNA
LAOL	L-Arabitol
LARAB	L-Arabinose
LARABe	L-Arabinose (Extracellular)
LCysTRNA	L-cysteinyl-tRNA
LEU	L-Leucine
LEUe	L-Leucine (Extracellular)
LEUm	L-Leucine (Mitochondria)
LGALNT	L-Galactonate
LGLNTRNA	L-glutaminyl-tRNA
LGLNTRNAm	L-glutaminyl-tRNA (Mitochondria)
LGLUTRNA	L-glutamyl-tRNA
LGLUTRNAm	L-glutamyl-tRNA (Mitochondria)
LGLYAL	L-Glyceraldehyde
LGT	(R)-S-Lactoylglutathione
LILEUTRNA	L-isoleucine-tRNA
LILEUTRNAm	L-isoleucine-tRNA (Mitochondria)
LIPOm	Lipoamide (Mitochondria)
LLAC	L-Lactate
LLACe	L-Lactate (Extracellular)
LLACm	(S)-Lactate (Mitochondria)
LLCT	L-Cystathionine
LLDACV	N-[5-amino-5-carboxypentanoyl]-L-cysteinyl-D-valine
LLeuTRNA	L-leucyl-tRNA
LLTRNA	L-Lysyl-tRNA
LMETTRNA	L-methionyl-tRNA
LMETTRNAm	L-methionyl-tRNA (Mitochondria)
LNST	Lanosterol
LPAA	Lysophosphatidylamine
LPC	Lysophosphatidylcholine
LPDME	Lysophosphatidyl-N-dimethylethanolamine
LPE	Lysophosphatidylethanolamine
LPG	Lysophosphatidylglycerol
LPheTRNA	L-phenylalanyl-tRNA

Abbreviation	Full name of metabolite
LPMME	Lysophosphatidyl-N-methylethanolamine
LPROTRNA	L-prolyl-tRNA
LPS	Lysophosphatidylserine
LRL	L-Ribulose
LRL5P	L-ribulose 5-phosphate
LRLe	L-Ribulose (Extracellular)
LSECTRNA	L-selenocysteinyl-tRNA
LSERTRNA	L-seryl-tRNA
LTHETRNA	L-threonyl-tRNA
LTHETRNAm	L-threonyl-tRNA (Mitochondria)
LTST	Lathosterol
LTyrTRNA	L-tyrosyl-tRNA
LTyrTRNAm	L-tyrosyl-tRNA (Mitochondria)
LValTRNA	L-valyl-tRNAVal
LXUL	L-Xylulose
LXULe	L-Xylulose (Extracellular)
LYS	L-Lysine
LYSe	L-Lysine (Extracellular)
MACAC	Maleylacetoacetate
MAGLY	Monoacylglycerol
MAL	(S)-Malate
MALACP	Malonyl-[acyl-carrier protein]
MALCOA	Malonyl Coenzyme A
MALe	(S)-Malate (Extracellular)
MALm	(S)-Malate (Mitochondria)
MALp	(S)-Malate (Peroxisome)
MAN	D-Mannose
MAN1P	Alpha-D-Mannose 1-phosphate
MAN6P	D-Mannose 6-phosphate
MANe	D-Mannose (Extracellular)
MANNAN	Mannan
MANNANe	Mannan (Extracellular)
MANOE	D-mannonate
MCECOA	2-methylaceto-acetyl CoA
MCRCOA	2-methylbut-2-enoyl-CoA
MCRCOAm	2-methylbut-2-enoyl-CoA (Mitochondria)
MELI	Melibiose
MELIe	Melibiose (Extracellular)
MENIN	Melanin
MESC	Mesaconate
MET	L-Methionine
METBUCOA	2-methylbutyryl coA
METBYCOA	2-methyl-2-hydroxybutyryl coA
METe	L-Methionine (Extracellular)
METHF	5,10-Methenyltetrahydrofolate
METHFm	5,10-Methenyltetrahydrofolate (Mitochondria)
METHOL	Methanol
METHOLe	Methanol (Extracellular)
METTHF	5,10-Methylenetetrahydrofolate
METTHFm	5,10-Methylenetetrahydrofolate (Mitochondria)
MGCOA	3-methylglutaconyl-CoA
MGCOAm	3-methylglutaconyl-CoA (Mitochondria)
MGDG	Monogalactosyl diglyceride

Abbreviation	Full name of metabolite
MHIS	N(pai)-Methyl-L-histidine
MI1P	1L-myo-Inositol 1-phosphate
MIP2C	Inositol-mannose-P-inositol-P-ceramide
MIPC	Mannose-inositol-P-ceramide
MLT	Maltose
MLTe	Maltose (Extracellular)
MLTIOSE	Maltotriose
MLTOSE	Maltotetraose
MMCOA	(S)-methylmalonyl-CoA
MMSHYm	2-methyl-3-oxopropanoate (Mitochondria)
MNT	D-Mannitol
MNT1P	D-Mannitol 1-phosphate
MNTe	D-Mannitol (Extracellular)
MTHF	5-Methyltetrahydrofolate
MTHFm	5-Methyltetrahydrofolate (Mitochondria)
MTHGXL	Methylglyoxal
MTHGXLm	Methylglyoxal (Mitochondria)
MTHPTGLU	5-Methyltetrahydropteroyltri-L-glutamate
MVL	(R)-Mevalonate
MYOBISPI	Myo-inositol 1,4-bisphosphate
MYOCYPI	1D-myo-inositol 1,2-cyclic phosphate
MYOI	Myo-Inositol
MYOIe	Myo-Inositol (Extracellular)
MZYMST	4-Methylzymsterol
N4HBZ	3-octaprenyl-4-hydroxybenzoate
Na	Sodium
NAD	NAD+
NADH	NADH
NADHm	NADH (Mitochondria)
NADHp	NADH (Peroxisome)
NADm	NAD+ (Mitochondria)
NADP	NAD+ (Peroxisome)
NADPH	NADPH
NADPHm	NADPH (Mitochondria)
NADPHp	NADPH (Peroxisome)
NADPm	NADP+ (Mitochondria)
NADPp	NADP+ (Peroxisome)
Nae	Sodium (Extracellular)
NAG	N-Acetyl-D-glucosamine
NAGA1P	N-Acetyl-D-glucosamine 1-phosphate
NAGA6P	N-Acetyl-D-glucosamine 6-phosphate
NAGe	N-Acetyl-D-glucosamine (Extracellular)
NAGLUm	N-Acetyl-L-glutamate (Mitochondria)
NAGLUPm	N-Acetyl-L-glutamate 5-phosphate (Mitochondria)
NAGLUSm	N-Acetyl-L-glutamate 5-semialdehyde (Mitochondria)
Nam	Sodium (Mitochondria)
NAORN	N2-Acetyl-L-ornithine
NAORNm	N2-Acetyl-L-ornithine (Mitochondria)
NCACE	Naringenin chalcone
NGEN	Naringenin
NH3	Ammonia
NH3e	Ammonia (Extracellular)
NH3m	Ammonia (Mitochondria)

Abbreviation	Full name of metabolite
NH4OH	Ammonium hydroxide
NICD	Nicotinamide
NICDm	Nicotinamide (Mitochondria)
NICNATE	Nicotinate
NICNATEm	Nicotinate (Mitochondria)
NICNUCLE	Nicotinate D-ribonucleotide
NITE	Nitriles
NITROPRO	2-nitropropane
NITROPROm	2-nitropropane (Mitochondria)
NMNm	Nicotinamide mononucleotide (Mitochondria)
NO	Nitric oxide
NOR	Norsolorinic acid
NPP	Octaprenyl diphosphate
NPRAN	N-(5-Phospho-D-ribosyl)anthranilate
O2	Oxygen
O2e	Oxygen (Extracellular)
O2m	Oxygen (Mitochondria)
O2p	Oxygen (Peroxisome)
OA	Oxaloacetate
OAe	Oxaloacetate (Extracellular)
OAHSER	O-Acetyl-L-homoserine
OAm	Oxaloacetate (Mitochondria)
OAp	Oxaloacetate (Peroxisome)
OBUT	2-Oxobutanoate
OBUTm	2-Oxobutanoate (Mitochondria)
OGT	Oxidized glutathione
OICAP	3-Carboxy-4-methyl-2-oxopentanoate
OICAPm	3-Carboxy-4-methyl-2-oxopentanoate (Mitochondria)
OIVAL	(R)-2-Oxoisovalerate
OIVALm	(R)-2-Oxoisovalerate (Mitochondria)
OMP	Orotidine 5'-phosphate
OMST	O-methylsterigmatocystin
OMVAL	2-keto-3-methyl-valerate
OMVALm	2-keto-3-methyl-valerate (Mitochondria)
ORN	L-Ornithine
ORNm	L-Ornithine (Mitochondria)
OROA	Orotate
OROAm	Orotate (Mitochondria)
OSLHSER	O-Succinyl-L-homoserine
OTHIO	Oxidized thioredoxin
OTHIOm	Oxidized thioredoxin (Mitochondria)
OXAL	Oxalate
OXALe	Oxalate (Extracellular)
OXGLY	Oxaloglycolate
P5C	(S)-1-Pyrroline-5-carboxylate
P5Cm	(S)-1-Pyrroline-5-carboxylate (Mitochondria)
PA	Phosphatidate
PAA	Phosphatidylamine
PABA	4-Aminobenzoate
PAC	Phenylacetic acid
PAD	2-Phenylacetamide

Abbreviation	Full name of metabolite
PADm	2-Phenylacetamide (Mitochondria)
PAm	Phosphatidate (Mitochondria)
PANT	(R)-Pantoate
PANTm	(R)-Pantoate (Mitochondria)
PAP	Adenosine 3',5'-bisphosphate
PAPm	Adenosine 3',5'-bisphosphate (Mitochondria)
PAPS	3'-Phosphoadenylylsulfate
PC	Phosphatidylcholine
PCACE	Protocatechuate
PCHO	Choline phosphate
PDME	Phosphatidyl-N-dimethylethanolamine
PDXAL	Pyridoxal
PDXAM	Pyridoxamine
PDXAM5PI	Pyridoxamine-5-phosphate
PDXI	Pyridoxine
PDXI5PI	Pyridoxine-5-phosphate
PDXL5PI	Pyridoxal-5-phosphate
PE	Phosphatidylethanolamine
PEm	Phosphatidylethanolamine (Mitochondria)
PEN	Penicillin
PENACID	Pennicillic acid
PENN	Penicillin N
PEP	Phosphoenolpyruvate
PEPm	Phosphoenolpyruvate (Mitochondria)
PETHM	Ethanolamine phosphate
PG	Phosphatidylglycerol
PGm	Phosphatidylglycerol (Mitochondria)
PGPm	Phosphatidylglycerophosphate (Mitochondria)
PHAC	Phenylacetate
PHACAL	Phenylacetaldehyde
PHACALm	Phenylacetaldehyde (Mitochondria)
PHAC-COA	Phenylacetyl-CoA
PHC	L-1-Pyrroline-3-hydroxy-5-carboxylate
PHCm	L-1-Pyrroline-3-hydroxy-5-carboxylate (Mitochondria)
PHE	L-Phenylalanine
PHEe	L-Phenylalanine (Extracellular)
PHEETHAL	Phenylethylalcohol
PHEETHALm	Phenylethylalcohol (Mitochondria)
PHEm	L-Phenylalanine (Mitochondria)
PHEMEm	Protoheme (Mitochondria)
PHEN	Prephenate
PHP	3-Phosphonooxypyruvate
PHPYR	Phenylpyruvate
PHSER	O-Phospho-L-homoserine
PHSP	Phytosphingosine 1-phosphate
PI	Orthophosphate
PIe	Orthophosphate (Extracellular)
PIm	Orthophosphate (Mitochondria)
PINS	1-Phosphatidyl-D-myo-inositol
PINS4P	1-Phosphatidyl-1D-myo-inositol 4-phosphate
PINSP	1-Phosphatidyl-1D-myo-inositol 3-phosphate
PMME	Phosphatidyl-N-methylethanolamine
PMVL	(R)-5-Phosphomevalonate

Abbreviation	Full name of metabolite
PNTO	(R)-Pantothenate
PPBG	Porphobilinogen
PPGIXm	Protoporphyrinogen IX
ppGpp	ppGpp
PPI	Pyrophosphate
PPIm	Pyrophosphate (Mitochondria)
PPIp	Pyrophosphate (Peroxixome)
PPMAL	2-Isopropylmaleate
PPMVL	(R)-5-Diphosphomevalonate
pppGpp	pppGpp
PPRIXm	Protoporphyrin IX (Mitochondria)
PRAM	5-Phosphoribosylamine
PRBAMP	N1-(5-Phospho-D-ribosyl)-AMP
PRBATP	N1-(5-Phospho-D-ribosyl)-ATP
PRECOR	Precorrin
PRFICA	1-(5'-Phosphoribosyl)-5-formamido-4-imidazolecarboxamide
PRFP	5-(5-Phospho-D-ribosylaminoformimino)-1-(5-phosphoribosyl)-imidazole-4-carboxamide
PRLP	N-(5'-Phospho-D-1'-ribulosylformimino)-5-amino-1-(5"-phospho-D-ribosyl)-4-imidazolecarboxamide
PRO	L-Proline
PROe	L-Proline (Extracellular)
PROm	L-Proline (Mitochondria)
PROP	Propanoate
PROPCOA	Propanoyl-CoA
PROPCOAm	Propanoyl-CoA (Mitochondria)
PROPCOAp	Propanoyl-CoA (Peroxisome)
PROPe	Propanoate (Extracellular)
PROPm	Propanoate (Mitochondria)
PRPP	5-Phospho-alpha-D-ribose 1-diphosphate
PS	Phosphatidylserine
PSm	Phosphatidylserine (Mitochondria)
PSPH	Phytosphingosine
PTATEe	Pectate (Extracellular)
PTRSC	Putrescine
PTRSCm	Putrescine (Mitochondria)
PURI5P	Pseudouridine 5'-phosphate
PYR	Pyruvate
PYRe	Pyruvate (Extracellular)
PYRm	Pyruvate (Mitochondria)
PYRp	Pyruvate (Peroxisome)
PYTE	Phytate
QA	Quinolinate
QH2m	Ubiquinol (Mitochondria)
Qm	Ubiquinone (Mitochondria)
QT	Quinate
R1P	D-Ribose 1-phosphate
R5P	D-Ribose 5-phosphate
R5Pm	D-Ribose 5-phosphate (Mitochondria)
RAFe	Raffinose (Extracellular)
RGT	Glutathione
RIB	D-Ribose
RIBe	D-Ribose (Extracellular)

Abbreviation	Full name of metabolite
RIBFLAV	Riboflavin
RIBFLAVe	Riboflavin (Extracellular)
RL	D-Ribulose
RL5P	D-Ribulose 5-phosphate
RLe	D-Ribulose (Extracellular)
RTHIO	Reduced thioredoxin
RTHIOm	Reduced thioredoxin (Mitochondria)
RX	RX
S	Sulfur
S23E	(S)-2,3-Epoxysqualene
S7P	Sedoheptulose 7-phosphate
SACP	N6-(L-1,3-Dicarboxypropyl)-L-lysine
SAH	S-Adenosyl-L-homocysteine
SAHm	S-Adenosyl-L-homocysteine (Mitochondria)
SAICAR	1-(5'-Phosphoribosyl)-5-amino-4-(N-succinocarboxamide)-imidazole
SAM	S-Adenosyl-L-methionine
SAMm	S-Adenosyl-L-methionine (Mitochondria)
SAMOB	S-adenosyl-4-methylthio-2-oxobutanoate
SAMOBm	S-adenosyl-4-methylthio-2-oxobutanoate (Mitochondria)
SAPm	S-Aminomethyldihydrolipoylprotein (Mitochondria)
SARC	Sarcosine
SARCm	Sarcosine (Mitochondria)
SCTLE	Scytalone
SER	L-Serine
SERe	L-Serine (Extracellular)
SERm	L-Serine (Mitochondria)
SHCR	Sirohydrochlorin
SIHM	Siroheme
SLF	Sulfate
SLFe	Sulfate (Extracellular)
SME	Shikimate
SME3P	Shikimate-3-phosphate
SNPI	Selenophosphate
SOR	L-Sorbose
SORe	L-Sorbose (Extracellular)
SOT	D-Sorbitol
SOTe	D-Sorbitol (Extracellular)
SPH	Sphinganine
SPMYLIN	Sphingomyelin
SPRM	Spermine
SPRMD	Spermidine
SQL	Squalene
ST	Sterigmatocystin
STAR	Starch
STARe	Starch (Extracellular)
SUCC	Succinate
SUCCe	Succinate (Extracellular)
SUCCm	Succinate (Mitochondria)
SUCCOAm	Succinyl coenzyme A (Mitochondria)
SUCCp	Succinate (Peroxisome)
SUCCSAL	Succinate semialdehyde
SUCCSALm	Succinate semialdehyde (Mitochondria)
SUCe	Sucrose (Extracellular)

Abbreviation	Full name of metabolite
SUFT	Sulfatide
T3P1	D-Glyceraldehyde 3-phosphate
T3P2	Glycerone phosphate
T6P	Tagatose-6-phosphate
TAGLY	Triacylglycerol
TAR	Tartrate
TARE	Tartrate
TDP	D-tagatose 1,6-bisphosphate
TGE	Tagatose
TGEe	Tagatose (Extracellular)
TGLCOA	Tigly CoA
THDP	Thiamine diphosphate
THF	Tetrahydrofolate
THFG	Tetrahydrofolyl-[Glu]
THFm	Tetrahydrofolate (Mitochondria)
THME	Thiamine
THMP	Thiamine monophosphate
THPTGLU	Tetrahydropteroyltri-L-glutamate
THR	L-Threonine
THRe	L-Threonine (Extracellular)
THRm	L-Threonine (Mitochondria)
THY	Thymine
THZ	4-methyl-5-(2-hydroxyethyl)thiazole
THZP	4-methyl-5-(2-phosphonooxyethyl)thiazole
TPI	D-myo-inositol 1,4,5-trisphosphate
TPP	Thiamine diphosphate
TR3DHT	L-threo-3-deoxy-hexulosonate
TRE	Alpha,alpha-Trehalose
TRE6P	Alpha,alpha-Trehalose 6-phosphate
TREe	Alpha,alpha-Trehalose (Extracellular)
TRMAS	L-threo-3-methylaspartate
TRNA	TRNA
TRNAm	TRNA (Mitochondria)
TRP	L-Tryptophan
TRPe	L-Tryptophan (Extracellular)
TRPM	Tryptamine
TRPTRNA	L-Tryptophanyl-tRNA
TTGGP	Trans, trans cis-geranyl geranyl diphosphate
TYR	L-Tyrosine
TYRe	L-Tyrosine (Extracellular)
TYRm	L-Tyrosine (Mitochondria)
UDP	UDP
UDPG	UDPglucose
UDPGAL	UDPgalactose
UDPGE	UDP-glucuronate
UDPNAG	UDP-N-acetyl-D-glucosamine
UDPNAGA	UDP-N-acetyl-D-galactosamine
UGC	Ureidoglycolate
UGCp	Ureidoglycolate (Peroxisome)
UMP	UMP
UPGIII	Uroporphyrinogen III
URA	Uracil
URAe	Uracil (Extracellular)

Abbreviation	Full name of metabolite
URATEp	Urate (Peroxisome)
UREA	Urea
UREAC	Urea-1-carboxylate
UREAp	Urea (Peroxisome)
UREIPRO	N-carbamoyl-β-alanine
URI	Uridine
UROCA	Urocanate
UTP	UTP
VAL	L-Valine
VALe	L-Valine (Extracellular)
VALm	L-Valine (Mitochondria)
VEML	Vermelone
VERA	Versicolorin A
VERAL	Versiconal
VERB	Versicolorin B
VESTINE	Vestine
VHA	Versiconal hemiacetal acetate
XAN	Xanthine
XANp	Xanthine (Peroxixome)
XMP	Xanthosine 5'-phosphate
XOL	Xylitol
XOLe	Xylitol (Extracellular)
XTSINE	Xanthosine
XUL	D-Xylulose
XUL5P	D-Xylulose 5-phosphate
XULe	D-Xylulose (Extracellular)
XYL	D-Xylose
XYLAN	Xylan
XYLANe	Xylan (Extracellular)
XYLe	D-Xylose (Extracellular)
ZYMST	Zymosterol

Suppl. Table 3: Macromolecular composition (Biomass formation) of *A. oryzae*. The measured values are from Pedersen et al. (1999) where *A. oryzae* (A1560, wild type strain) grown on glucose and ammonia for a growth rate of 0.10 1/h and a specific glucose uptake rate of 1.12 mmol glucose/(g DW.h). For Glycogen, the measured value is from Debois et al. (1995). For Glucan and Chitin, the measured values are from Pedersen et al. (1996).

Biomass component	%[g/g]	%[mol/mol]	Average MW [g/mol of monomers in polymer]	Stoichiometric coefficient [mmol/ g DW]
**Proteins**	40.0	71.24	134.58	3.50075
**Carbohydrates**	28.0			
Glycogen	0.1	0.04	666.6	0.00212
Chitin	7.0	8.29	203.2	0.40759
Glucan	20.8	30.82	162.1	1.51453
**RNA**	5.3	3.72	341.9	0.18259
**DNA**	0.8	0.58	332.3	0.02836
**Lipids**	6.8	2.09	780.43	
Neutral Lipids				
Triacylglycerol	2.12	0.53	954.96	0.02617
Free fatty acids	0.35	0.28	301.31	0.01365
Phospholipids				
Phosphatidylethanolamine	0.97	0.30	782.50	0.01468
Phosphatidylcholine	2.38	0.68	834.80	0.03356
Phosphatidylserine	0.40	0.11	827.32	0.00564
Phosphatidylamine	0.58	0.18	755.24	0.00903
**D-Mannitol**	3.3	4.34	182.2	0.21333
**Glycerol**	0.7	1.82	92.1	0.08952
**Ash**	15.1	-	-	-

Average MW of biomass (g/mol) = 239.68
Elemental biomass composition = $CH_{1.5}O_{0.53}N_{0.19}P_{0.005}S_{0.01}$
MW on C-mole (g/C-mol) = 25.1

Suppl. Table 4: Protein composition of *A. oryzae*. The measured values are from Pedersen et al. (1999).

Amino acid type	MW [g/mol]	Composition [%mol]	Composition [% (g/g)]
Alanine	89.1	9.5	6.6
Arginine	174.2	4.4	6.0
Asparagine	132.1	4.6	4.7
Aspartate	133.1	4.6	4.8
Cysteine	121.2	1.1	1.0
Glutamate	147.1	8	9.2
Glutamine	146.1	8	9.1
Glycine	75.1	9.4	5.5
Histidine	155.2	2	2.4
Isoleucine	131.2	4.5	4.6
Leucine	131.2	6.9	7.1
Lysine	146.2	5.7	6.5
Methionine	149.2	1.4	1.6
Phenylalanine	165.2	3.1	4.0
Proline	115.1	4.7	4.2
Serine	105.1	6.6	5.4
Threonine	119.1	4.8	4.5
Tryptophan	204.2	1.8	2.9
Tyrosine	181.2	2.8	4.0
Valine	117.1	6.4	5.9
Average MW of protein (g/mol) = 134.58			

Suppl. Table 5: DNA composition of *A. oryzae*. The measured values are from Pedersen et al. (1999).

dNTP type	MW [g/mol]	Composition [% (mol)]	Composition [% (g/g)]
dAMP	349.2	24.2	25.51
dTMP	322.2	24.2	23.53
dGMP	347.2	25.8	27.04
dCMP	307.2	25.8	23.92
Average MW of DNA (g/mol) = 332.3			
**Remark:** Assuming the same composition in NTP and NMP			

Suppl. Table 6: RNA composition of *A. oryzae*. The measured values are from Pedersen et al. (1999).

NTP type	MW [g/mol]	Composition [% (mol)]	Composition [% (g/g)]
AMP	347.2	25.6	26.1
UMP	324.2	26.2	24.9
GMP	363.2	28.6	30.5
CMP	323.2	19.6	18.6

Average MW of RNA (g/mol) = 341.9

Remark Assuming the same composition in NTP and NMP

Suppl. Table 7: Lipid composition of *A. oryzae*. The measured values are from Sakuradani et. al (1999).

Lipid type	Average MW [g/mol]	Composition [%mol]	Composition [% (g/g)]
Triacylglycerol	954.96	25.50	31.20
Free fatty acids	301.31	13.3	5.13
Phosphatidylethanolamine	782.50	14.3	14.34
Phosphatidylcholine	834.80	32.7	34.98
Phosphatidylserine	827.32	5.5	5.83
Phosphatidylamine	755.24	8.8	8.52
Average MW of total lipids (g/mol) = 780.43			

Suppl. Table 8: Triacylglycerol composition of *A. oryzae*. The measured values are from Sakuradani et. al (1999).

Fatty acids	MW [g/mol]	Composition [%mol]	% [mol/100 mol of total fatty acids in lipid]
C16:0	239.2	47.10	11.8
C16:1	237.2	2.42	0.6
C18:0	267.5	15.35	4.3
C18:1	265.3	53.28	14.8
C18:2	263.2	233.62	64.4
C18:3	261.2	14.99	4.1

Suppl. Table 9: Phospholipid composition of *A. oryzae*. The measured values are from Sakuradani et. al (1999).

**Fatty acids**	**MW [g/mol]**	**Composition [%mol]**	**% [mol/100 mol of total fatty acids in lipid]**
C16:0	239.2	58.56	17.3
C16:1	237.2	1.44	0.4
C18:0	267.5	5.43	1.8
C18:1	265.3	42.61	13.9
C18:2	263.2	197.54	64.2
C18:3	261.2	7.44	2.4

Suppl. Table 10: Free fatty acid composition of *A. oryzae*. The measured values are from Sakuradani et al. (1999).

Fatty acids	MW [g/mol]	Composition [%mol]	% [mol/100 mol of total fatty acids in lipid]
C16:0	239.2	27.20	21.6
C16:1	237.2	0.51	0.4
C18:0	267.5	3.04	2.7
C18:1	265.3	15.34	13.5
C18:2	263.2	66.73	58.3
C18:3	261.2	4.04	3.5

References:

Books, paper publications and databases are applied for genome-scale reconstruction

Brown, D.W., Yu, J.H., Kelkar, H.S., Fernandes, M., Nesbitt, T.C., Keller, N.P., Adams, T.H., and Leonard, T.J: **Twenty-five coregulated transcripts define a sterigmatocystin gene cluster in *Aspergillus nidulans***. *PNAS* 1996, **93**: 1418-1422.

Carlsen M., Nielsen J., Villadsen J: **Growth and alpha-amylase production by *Aspergillus oryzae* during continuous cultivations.** *Journal of Biotechnology* 1996, 81-93.

David H, Hofmann G, Oliveira A.P, Jarmer H, Nielsen J: **Metabolic network driven analysis of genome-wide transcription data from *Aspergillus nidulans***. *Genome Biology* 2006, **7** (11).

David H, Akesson M, Nielsen J: **Reconstruction of the central carbon metabolism of *Aspergillus niger***. *European Journal of Biochemistry* 2003, **270**: 4243-4253.

Debois, M., Heydorn, A., Obel, N: **Macromolecule analysis of *Aspergillus oryzae***. *Master Thesis* 1995, Technical University of Denmark.

Forster J, Famili I, Fu P, Palsson B.O, Nielsen J: **Genome-scale reconstruction of the *Saccharomyces cerevisiae* metabolic network**. *Genome Research* 2003, **13**:244-253.

Machida M, Asai K, Sano M, Tanaka T, Kumagai T, Terai G, Kusumoto KI, Arima T, Akita O, Kashiwagi Y *et al*: **Genome sequencing and analysis of *Aspergillus oryzae***. *Nature* 2005, **438**:1157-1161.

McMurry, J. and Begley, T: **The Organic Chemistry of Biological Pathways**. *Englewood* 2005, CO: Roberts and Co. Publishers.

Pedersen H, Carlsen M, Nielsen J: **Identification of enzymes and quantification of metabolic fluxes in the wild type and in a recombinant *Aspergillus oryzae* strain**. *Applied and Environmental Microbiology* 1999, **65**:11-19.

Pedersen, H: **Metabolic flux in *Aspergillus oryzae***. *Master Thesis* 1996, Technical University of Denmark.

Sakuradani, E, Kobayashi M, Shimizu S: **Delta 9-fatty acid desaturase from arachidonic acid producing fungus**. *European Journal of Biochemistry* 1999, **260**: 208-216.

Hilditch, S, Berghäll, S, Kalkkinen, N, Penttilä, M, Richard, P: T**he missing link in the fungal D-galacturonate pathway; Identification of the L-threo-3-deoxy-hexulosonate aldolase**. *Journal of Biological Chemistry* 2007, 1-16.

Tsai, H.F., Fujii, I., Watanabe, A., Wheeler, A.H., Chang, Y.C., Yasuoka, Y., Ebizuka, Y. and Kwon-Chung, K.J: **Pentaketide melanin biosynthesis in *Aspergillus fumigatus* requires chain-length shortening of a heptaketide precursor**. *Journal of Biological Chemistry* 2001, **276**: 29292-29298.

***Aspergillus* oryzae genome database**
[http://www.bio.nite.go.jp/dogan/MicroTop?GENOME_ID=ao]

BioCyc pathway database
[http://biocyc.org/server.html]

Comparative genome analysis database:
[http://ergo.integratedgenomics.com/ERGO]

KEGG pathway database:
[http://www.kegg.com]
